# Jianpi‐Huogu Prescription Repairs Nontraumatic Osteonecrosis of the Femoral Head by Inhibiting NAMPT/STK11/HMGCR/ACAT1 Axis‐Mediated Lipid Production

**DOI:** 10.1111/jcmm.70858

**Published:** 2025-09-23

**Authors:** Tao Li, Zhaochen Ma, Shuangrong Gao, Chu Zhang, Yan Jia, Huang Feng, Na Lin, Weiheng Chen, Yanqiong Zhang

**Affiliations:** ^1^ Institute of Chinese Materia Medica China Academy of Chinese Medical Sciences Beijing China; ^2^ Third Affiliated Hospital of Beijing University of Chinese Medicine; Engineering Research Center of Chinese Orthopaedics and Sports Rehabilitation Artificial Intelligent, Ministry of Education Beijing China; ^3^ School of Chinese Materia Medica Yunnan University of Chinese Medicine Kunming China

**Keywords:** Jianpi‐Huogu Prescription, lipid metabolism, NAMPT/STK11/HMGCR/ACAT1 signal axis, nontraumatic osteonecrosis of the femoral head, transcriptome‐based network investigation

## Abstract

Jianpi‐Huogu Prescription (JHP), integrating the classic Chinese herbal formulas Linggui‐Zhugan Decoction and Si‐Wu Decoction, has shown clinical efficacy in treating early nontraumatic osteonecrosis of the femoral head (NONFH). In this study, 299 chemical constituents of JHP were identified using ultra‐performance liquid chromatography–quadrupole‐time of flight mass spectrometry. Pharmacological evaluations in rats with early NONFH demonstrated that JHP attenuated femoral head pathological changes, improved gait parameters, increased mechanical pain thresholds, reduced serum levels of triglycerides, total cholesterol, low‐density lipoprotein, very‐low‐density lipoprotein, and the TXB₂/6‐keto‐PGF₁α ratio while elevating high‐density lipoprotein, decreased serum inflammatory cytokines (IL‐1β, IL‐6, TNF‐α) and the RANKL/OPG ratio, and restored bone trabecular morphology and reduced the number of bone marrow adipocytes. Mechanistically, through transcriptomic profiling, network calculations and experimental validation, JHP downregulated NMNAT1, NMNAT3, and NAMPT in the femoral head, thereby reducing NAD^+^ synthesis, increased ATP production (evidenced by a decreased ADP/ATP ratio) and upregulated STK11 expression, activated p‐HMGCR while suppressing ACAT1 thus inhibiting cholesterol synthesis and lipid accumulation, and surface plasmon resonance confirmed direct binding of its active components (ligustilide, 5,6,4′‐trihydroxy‐7,3′‐dimethoxyflavone, (Z)‐3‐butylidenephthalide, senkyunolide H, ferulic acid, guanosine) to NMNAT1. In conclusion, JHP ameliorates early NONFH by regulating lipid metabolism, improving hypercoagulability, and restoring bone homeostasis through the NMNAT1/NMNAT3/NAMPT/STK11/HMGCR/ACAT1 axis.

## 1 Introduction

Non‐traumatic osteonecrosis of the femoral head (NONFH) is a disease characterised by local bone cell or bone marrow constituent death, typically owing to venous stasis, arterial blood supply injury, or bone interruption [1]. It usually affects young people aged 20–40 years and continuously leads to the loss of hip joint function. Excessive corticosteroid use is a common cause, with NONFH prevalence reaching 9%–40% in patients receiving long‐term and overdosed hormone therapy [1]. Existing therapeutic drugs, such as anticoagulants [2], fibrinolysis enhancers [3], vasodilators [4], and lipid‐lowering drugs [5], alleviate pathological changes and pain associated with osteonecrosis of the femoral head but do not address hip joint inflammation or prevent femoral head collapse. Therefore, identifying novel therapeutic targets and developing effective strategies for NONFH management are clinically important.

Jianpi‐Huogu Prescription (JHP), originated from the two classic Chinese herbal formulas Linggui‐Zhugan Decoction and Si‐wu Decoction [6, 7], comprises 11 Chinese herbs, including *Citrus reticulata* Blanco (CP), *Paeonia lactiflora* Pall. (CS), *Codonopsis pilosula* (branch.) Nannf. (DS) and *Rehmannia glutinosa* Libosch. (SDH), *Cinnamomum cassia* Presl. (GZ), *Angelica sinensis* (Oliv.) Diels (DG), *Cyathula officinalis* Kuan (CNX), *Ligusticum chuanxiong* Hort. (CX), and *Atractylodes macrocephala* Koidz. (CBZ) and *Poria* cocos (Schw.) Wolf (FL), and deerhorn glue (LJJ), has been extensively used for the treatment of NONFH with a satisfying clinical efficacy. Our previous clinical study indicated that the collapse rate of the femoral head in 53 NONFH patients treated with JHP was only 26.14% [8], significantly lower than the average collapse rate of 49% reported by Mont [9]. This suggests that JHP may effectively prevent femoral head collapse and delay the time of joint replacement. Mechanically, recent studies have revealed that JHP ethyl acetate extract significantly inhibited bone marrow mesenchymal stem cell adipogenesis during NONFH progression by regulating BMP and Wnt pathways [10]. Additionally, it has been reported that JHP activates the VEGF/VEGFR2/PI3K/Akt pathway and Akt/JNK/p38 MAPK pathway to repair vascular injury in alcohol‐induced or hormone‐induced femoral head necrosis [11, 12]. However, the underlying pharmacological mechanisms of JHP remain completely elucidated.

To address this aspect, this study aimed to systematically identify the chemical constituents of JHP using ultra‐performance liquid chromatography‐quadrupole‐time of flight mass spectrometry (UPLC‐Q‐TOF‐MS), and putative targets were collected from the ETCM 2.0 [13] database. Subsequently, transcriptomic profiling was conducted based on clinical whole blood samples from early NONFH patients and controls, as well as clinical whole blood samples before and after treatment with JHP, to identify the disease‐related genes and the effective targets of this prescription, respectively. Following the construction of the ‘disease‐related gene‐drug putative target’ interaction network, the calculation of topological features, and the functional enrichment analysis, candidate targets of JHP against early NONFH were screened and further validated through a series of in vivo experiments using a rat model of NONFH established by methylprednisolone sodium succinate.

## 2 Materials and Methods

### 2.1 Ethics Statement

This study was approved by the Research Ethics Committee of the Institute of Chinese Materia Media, Wangjing Hospital, China (approval number: 81473695) and the Third Affiliated Hospital of Beijing University of Chinese Medicine (approval number: BZYSY‐2021KYKTPJ‐01). Informed consent was obtained from all the patients.

All animal experiments were approved by the Experimental Animal Ethics Committee of the Institute of Chinese Materia Medica, China Academy of Chinese Medical Sciences, Beijing, China (Ethics Approval Number: 2022B086), and were performed in accordance with international guidelines on the ethical use of animals.

### 2.2 Chemicals and Materials

The chemicals and materials are described in detail in Appendix S2: Section 1. All enzyme‐linked immunosorbent assay (ELISA) kits and antibodies are listed in Table S2.

### 2.3 Clinical Cohorts

Data were collected from four early NONFH patients treated with JHP at the Third Affiliated Hospital of Beijing University of Traditional Chinese Medicine. All enrolled patients completed the corresponding Case Report Form (CFR) stage, collecting whole blood samples before and after JHP treatment and storing them in a −80°C refrigerator. The inclusion criteria are described in Appendix S2: Section 2. The patient clinical information is shown in Table S3.

### 2.4 Preparation of JHP

The detailed preparation method for JHP is available in Appendix S2: Section 3 according to our previous study [8, 10, 14, 15].

### 2.5 Analysis of Chemical Constituents of JHP

Qualitative identification of chemical constituents in JHP decoction using UPLC‐Q‐TOF‐MS. Detailed sample pretreatment methods, chromatographic elution conditions, and mass spectrometry collection conditions are provided in Appendix S2: Section 4.

### 2.6 Animals

Fifty male Sprague–Dawley rats (weighing 230–250 g) were purchased from Beijing Vitonglihua Biotechnology Co. Ltd. (Certificate of Conformity: SYXK2021‐0017). The rats were acclimated to laboratory conditions (23°C, 12/12 h light/dark, 50% humidity, ad libitum access to food and water) for 1 week before the experiments.

### 2.7 Establishment of Early NONFH Rat Model

Each rat was injected with lipopolysaccharide (20 μg/kg) through the tail vein from Day 1 to Day 2, once daily. Subsequently, methylprednisolone sodium succinate (40 mg/kg) was alternately injected into both buttocks once daily from Days 3 to 5. A proportional volume of physiological saline was injected at the same time as the control group for model administration. In addition, 80,000 units of penicillin sodium were injected into the gluteal muscles of each group of rats to prevent infection. Information regarding animal grouping and administration dose is provided in Appendix S2: Section 5.

### 2.8 Mechanical Pain Detection and Gait Analysis

From the first week of administration, we tested the pain threshold of each group of rats on the hind limbs and soles once a week using the Aesthesio von Frey acupuncture pain test kit. Gait analysis was performed using the CatWalk XT Version 10.6 instrument one day before rat execution.

### 2.9 Prediction of JHP Putative Targets and Collection of Early Nonfh‐Related Genes

Putative JHP targets were collected from ETCM 2.0 (http://www.tcmip.cn/ETCM2/front/#/), and early NONFH‐related genes were collected from the GEO and HPO databases. Detailed information on the collection protocols and genes is provided in Appendix S2: Section 6 and Table S4.

### 2.10 Identification of JHP Effective Targets Based on Transcriptomics Profiling

Whole blood samples collected from the clinical cohorts of different groups were used for gene expression profiling using RNA‐seq (GSE254972). Differentially expressed genes (DEGs) among different groups were identified using the edge R package with a *p* value < 0.05 and fold change > 1.5/< 0.67. Among these, JHP effective targets (DEGs of the JHP pretreatment group vs. the JHP posttreatment group) were identified. Details of the effective targets of JHP are provided in Table S5.

### 2.11 Network Construction and Analysis

The ‘disease‐related gene‐drug target’ interaction network was constructed using the links among the early NONFH‐related genes, drug putative, and effective targets collected from the String 10.0 database (http://string‐db.org/) and visualised by the Cytoscape 3.9.0 platform (https://cytoscape.org/). The original data of the interaction network is presented in Table S6.

### 2.12 Enzyme‐Linked Immunosorbent Assay

According to the routine scheme [16], the blood biochemical indices, expression levels of NMNAT3, and contents of ATP and ADP in the femoral head tissues were detected by enzyme‐linked immunosorbent assay.

### 2.13 Histopathological Detection

According to the conventional protocol [17], histological examination was performed using haematoxylin and eosin (H&E) and oil red O.

### 2.14 Immunohistochemistry

Based on our previous study [18], we examined the subcellular localization and expression levels of p‐HMGCR protein in femoral head tissues using immunohistochemistry with a DAB kit and a rabbit/mouse two‐step detection kit. Immunoreactive data were quantitatively analysed using ImageJ 1.42q (Image Progression and Analysis in Java, https://imagej.nih.gov/ij/) according to the user's guide.

### 2.15 Western Blot Analysis

In our previous study [16], the expression levels of NMNAT1, NAMPT, STK11, and ACAT1 proteins in femoral head tissues were detected via western blot analysis, with β‐actin as the internal control.

### 2.16 WST‐8 Detection

The NAD^+^/NADH ratio in the femoral head of rats was determined using the WST‐8 method [19].

### 2.17 Molecular Docking Simulation

Molecular docking of small molecules with protein NMNAT1 (PDB ID: 8Q8E) was performed using AutoDock Vina (Version 1.2.3) software. Then, PyMOL software (Version 3.0.3) and LigPlot+ software (Version 2.2.8) were used for result analysis and plotting operations.

### 2.18 Surface Plasmon Resonance (SPR)

SPR assay was performed using the Biacore T200 (Cytiva, USA). Recombinant human NMNAT1 protein (Mabnus, M507064) was immobilised on a Biacore CM5 sensor chip via the primary amine groups. The compounds were superfused at a rate of 30 μL·min^−1^ for 60 s to allow for association, followed by 150 s for dissociation over immobilised protein in PBS, 5% DMSO running buffer (1.05 × PBS, 0.5% P20 surfactant [Cytiva, BR100054], 5% DMSO, pH 7.4). Compounds were tested for binding at 1.56 to 50 μM. Normalisation of the data involved transformation of the y − axis such that the theoretical maximum amount of binding for a 1:1 interaction with the protein surface corresponded to a sensor response of 100 relative units (RU). Small molecule compounds information is provided in in Appendix S2: Section 7.

### 2.19 Statistical Analysis

GraphPad Prism 8.0 software (San Diego, CA, USA) was used for the statistical analysis. The data were expressed as mean ± SD and analysed with one‐way ANOVA, followed by Bonferroni's or Dunnett's post hoc tests for multiple comparisons. Statistical significance was set at *p* < 0.05.

## 3 Results

### 3.1 JHP Attenuates the Pathological Changes of the Femoral Head in Early NONFH Rats

**FIGURE 1:**
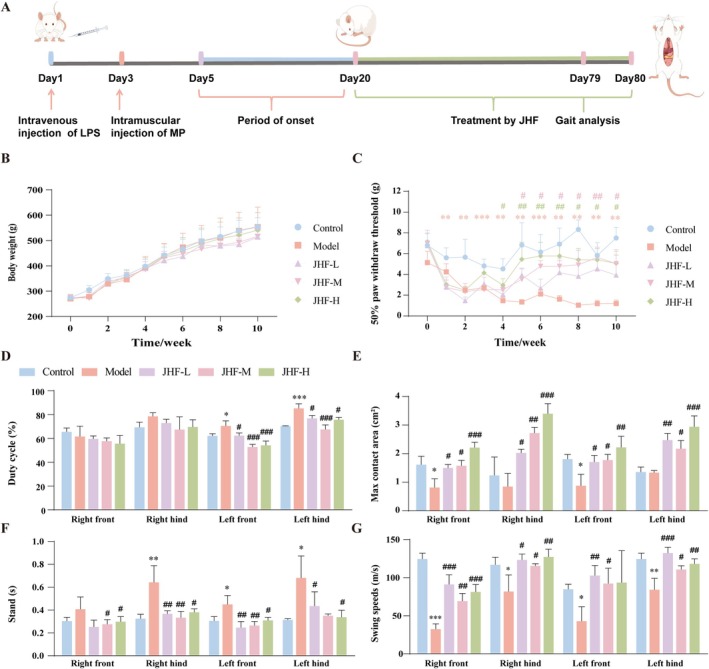
JHP attenuates the pathological changes of the femoral head in early NONFH rats. (A) The time axis of the establishment of NONFH rat model induced by methylprednisolone sodium succinate and the administration of JHP; (B) The changes of rat body weight in different groups; (C) The changes of mechanical pain threshold of rats in different groups; (D–G) The results of gait analysis of rats in different groups; D, E, F and G are the changes of standing time, swing speed, duty cycle and maximum contact area of the limbs, respectively. *p < 0.05, **p < 0.01, ***p < 0.001, vs. control group; ^#^ *p* < 0.05, ^##^ *p* < 0.01, ^###^ *p* < 0.001, vs. model group.

Figure 1A,B display the construction and administration time axes of the early NONFH rat model and changes in rat body weight, respectively. In addition, JHP significantly increased the mechanical pain threshold in early NONFH rats (*p < 0.05*, Figure 1C). In gait analysis, early NONFH rats had varying degrees of walking abnormalities owing to pathological changes and pain in the femoral head, including significantly increased standing time and duty cycle, decreased limb swing speed, and maximum contact area between the limbs and the ground (all *p < 0.05*, Figure 1D–G). Subsequent treatment with JHP at various dosages improved all the abnormal indices (all *p < 0.05*, Figure 1C–G).

### 3.2 JHP Improves the Abnormal Bone and Lipid Metabolisms in Early NONFH Rats

**FIGURE 2:**
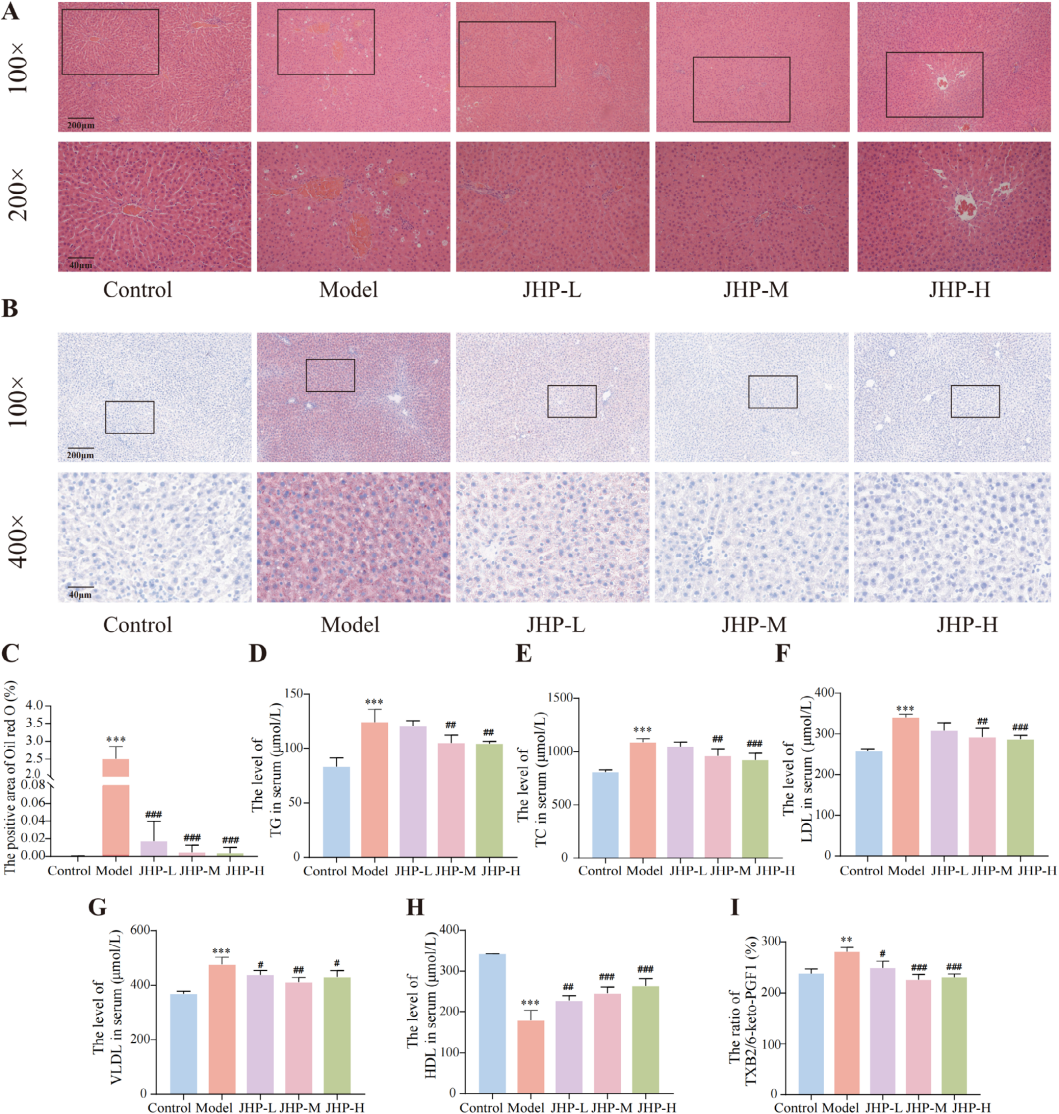
JHP improves the abnormal lipid metabolism and blood hypercoagulable status in early NONFH rats (*n* = 3). (A) Liver H&E staining; (B) Liver oil red O staining; (C) Statistics of positive expression area based on liver oil red O staining; (D–H) Serum content of TG, TC, LDL, VLDL, and HDL in different groups; (I) The ratio of platelet‐activating factor TXB2/6‐keto‐PGF1α in different groups. *p < 0.05, **p < 0.01, ***p < 0.001, vs. control group; ^#^ *p* < 0.05, ^##^ *p* < 0.01, ^###^ *p* < 0.001, vs. model group. 6‐K‐PGF1α, 6‐keto prostaglandin F1α; H&E staining, haematoxylin–eosin staining; HDL, high‐density lipoprotein; LDL, low‐density lipoprotein cholesterol; TC, total cholesterol; TG, triglyceride; TXB2, thromboxane B2; VLDL, very low‐density lipoprotein cholesterol.

H&E staining revealed a disordered arrangement of sinusoidal cells, abundant fat vacuoles in lobular cells, and evident inflammatory infiltration in liver tissues of the model group, suggesting increased lipid droplets and hepatocyte steatosis. Conversely, hepatic lobular cells in the JHP groups were neatly arranged, with no obvious fat vacuoles or inflammatory infiltration observed (Figure 2A). Liver Oil Red O staining results demonstrated severe fatty degeneration in the liver of the model group, whereas no significant abnormal changes were observed in the JHP groups (Figure 2B,C). Compared to the control group, early NONFH rats exhibited significantly increased levels of TG, TC, LDL, VLDL, and the ratio of blood clotting marker TXB2/6‐keto‐PGF1α in sera, alongside decreased HDL (all *p < 0.05*, Figure 2D–I). Abnormal lipid metabolism and hypercoagulable blood status improved after 8 weeks of JHP treatment (all *p < 0.05*; Figure 2).

**FIGURE 3:**
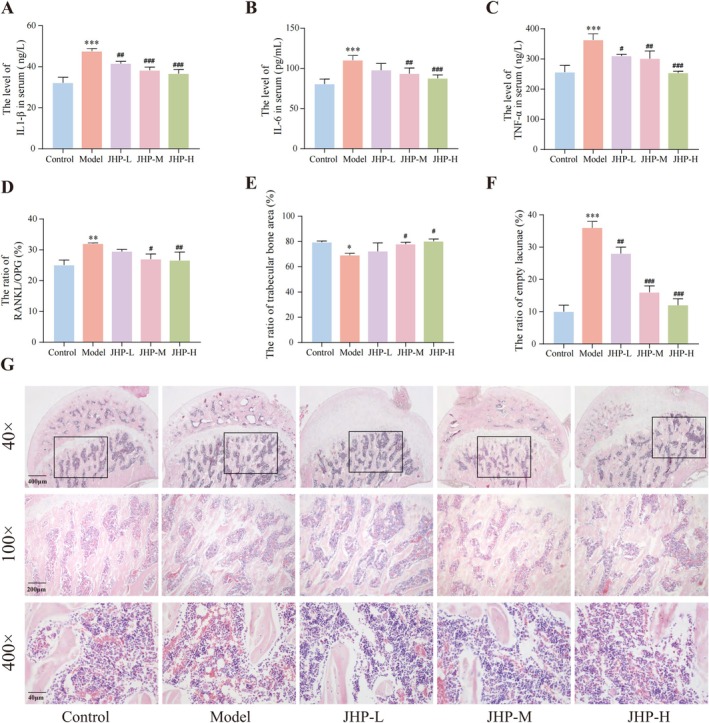
JHP improves the abnormal bone metabolism in early NONFH rats (*n* = 3). (A–C) Serum levels of IL‐1β, IL‐6 and TNF‐α in different groups; (D) Ratio of RANKL/OPG in rats' sera of different groups; (E–F) Statistics on trabecular area and empty bone lacuna rate in different groups; (G) Representative H&E photograph of femoral head tissues collected from different groups. *p < 0.05, **p < 0.01, ***p < 0.001, vs. control group; ^#^ *p* < 0.05, ^##^ *p* < 0.01, ^###^ *p* < 0.001, vs. model group. IL‐1β, interleukin‐1β; IL‐6, interleukin‐6; OPG, osteoprotegerin; RANK, nuclear factor‐κ B ligand; RANKL, receptor activator for nuclear factor‐κ B ligand; TNF‐α, tumour necrosis factor‐α.

Additionally, JHP treatment effectively reduced serum levels of inflammatory factors IL‐1β, IL‐6, and TNF‐α, as well as the ratio of osteoclast differentiation marker RANKL/OPG in early NONFH rats (all *p < 0.05*, Figure 3A–D). The results of the H&E staining indicated a decrease in the proportion of calcification in femoral head tissues in the model group. Furthermore, the bone trabeculae exhibited rough surfaces, a loose arrangement, and uneven texture. The osteocytes were not full, while the empty bone lacunae increased significantly. Additionally, adipocytes in the bone marrow of the femoral head increased, enlarged, and even fused. After 8 weeks of JHP treatment, the trabecular morphology of the femoral head in the JHP‐M and JHP‐H groups was more complete. Although a certain number of empty bone lacunae were observed, they were fewer than those in the model group, and the morphology and number of adipocytes in the bone marrow cavity of the femoral head were significantly improved (Figure 3E–G).

### 3.3 Chemical Profiling of JHP

**FIGURE 4:**
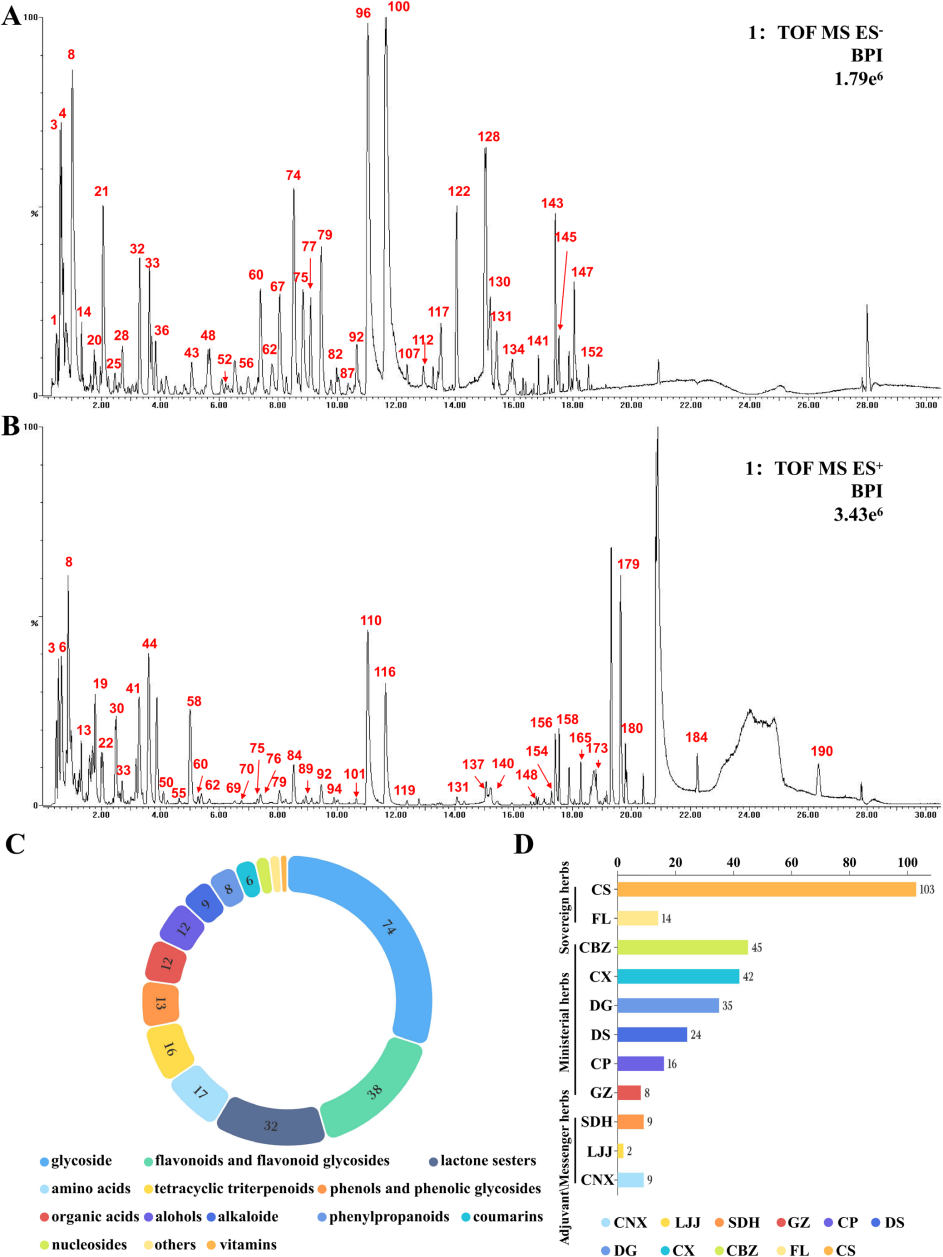
Chemical profiling of JHP. (A) The chemical base peak ion (BPI) chromatogram of JHP in the negative ion mode. (B) The BPI chromatogram of JHP in the positive ion mode. (C) Structural classification of compounds contained in JHP. (D) The number of chemical components for each herb. UPLC‐Q‐TOF‐MS, ultra‐performance liquid chromatography‐quadrupole‐time of flight‐mass spectrometry.

Overall, 299 chemical constituents in JHP were identified using Masslynx 4.2 and UNIFI 1.9 software, comprising 153 constituents in negative ion mode (Figure 4A) and 146 constituents in positive ion mode (Figure 4B). These chemical constituents included 74 glycosides, 38 flavonoids and flavonoid glycosides, 32 lactones and sesters, 17 amino acids, 16 tetracyclic triterpenoids, 13 phenols and phenolic glycosides, 12 organic acids, 12 alcohols, nine alkaloids, eight phenylpropanoids, six coumarins, four nucleosides, two vitamins, and three others, identified or tentatively characterised by their retention times and fragmentation patterns (Figure 4C). The herbs and their component numbers are summarised in Figure 4D, with CS having the largest number of components (103), followed by CBZ, CX, and DG, containing 45, 42, and 35 components, respectively. Detailed chemical information on the components and their pharmacological effects is shown in Table S1 and Figure S1, respectively.

### 3.4 NAMPT/STK11/HMGCR/ACAT1 Signal Axis‐Mediated Lipid Metabolism Was Screened as One of the Candidate Network Targets of JHP Against Early NONFH

Overall, 1172 putative targets hitting 299 chemical constituents in JHP were collected from ETCM 2.0. Differentially expressed gene analysis based on our clinical transcriptomic sequencing data identified 594 JHP‐effective targets, including 227 upregulated genes and 367 downregulated genes, in blood cell samples from patients with NONFH before and after JHP treatment. Our previous gene expression microarray data from GSE123568 identified 1748 early NONFH‐related genes [20]. We also identified 909 early NONFH‐related genes in the HPO database. Based on the links among the early NONFH‐related genes, JHP putative and effective targets, the ‘disease‐related gene‐drug putative target’ interaction network was constructed, and 734 network targets with Degree ≥ 22, Maximal Clique Centrality ≥ 45,156.6 were screened. Functionally, the network targets were mainly involved in early NONFH‐related lipid metabolism disorders, immune‐inflammatory imbalance, abnormal bone metabolism, and obstruction of blood circulation and were associated with clinical symptoms such as obesity, tongue edema, hip pain, episodic pain, joint swelling, and tongue ecchymosis. Notably, glycerolipid metabolism, glycerophospholipid metabolism, fatty acid metabolism, and thermogenesis, significantly enriched by the network targets of the ‘disease‐related gene‐drug putative target’ interaction network, were also associated with the disturbance of lipid metabolism during NONFH progression (Figure S2). Results of the association of omics data with clinical efficacy and animal efficacy are shown in Figure S3, respectively.

Particularly, the candidate network target Nicotinamide Phosphoribosyl Transferase (NAMPT), which mainly participates in lipid metabolism, was one of the early NONFH‐related genes and the effective targets of JHP. Its expression was upregulated in the early NONFH group but downregulated after treatment with JHP. In addition, the NAMPT‐related genes Nicotinamide Nucleotide Adenylyl Transferase 1 (NMNAT1), Nicotinamide Nucleotide Adenylyl Transferase 3 (NMNAT3), and 3‐Hydroxy‐3‐Methylglutaryl‐CoA Reductase (HMGCR) were all JHP effective targets. Downstream genes of NAMPT [21], Serine/Threonine Kinase 11 (STK11) [22], and Acetyl‐CoA Acetyltransferase 1 (ACAT1) [23] are early NONFH‐related genes. Therefore, we hypothesised that the NAMPT/STK11/HMGCR/ACAT1 axis, involved in lipid metabolism disturbance, might be a candidate target of JHP against early NONFH.

### 3.5 JHP Promotes the Repair of Early NONFH by Regulating NAMPT/STK11/HMGCR/ACAT1 Signal Axis

**FIGURE 5:**
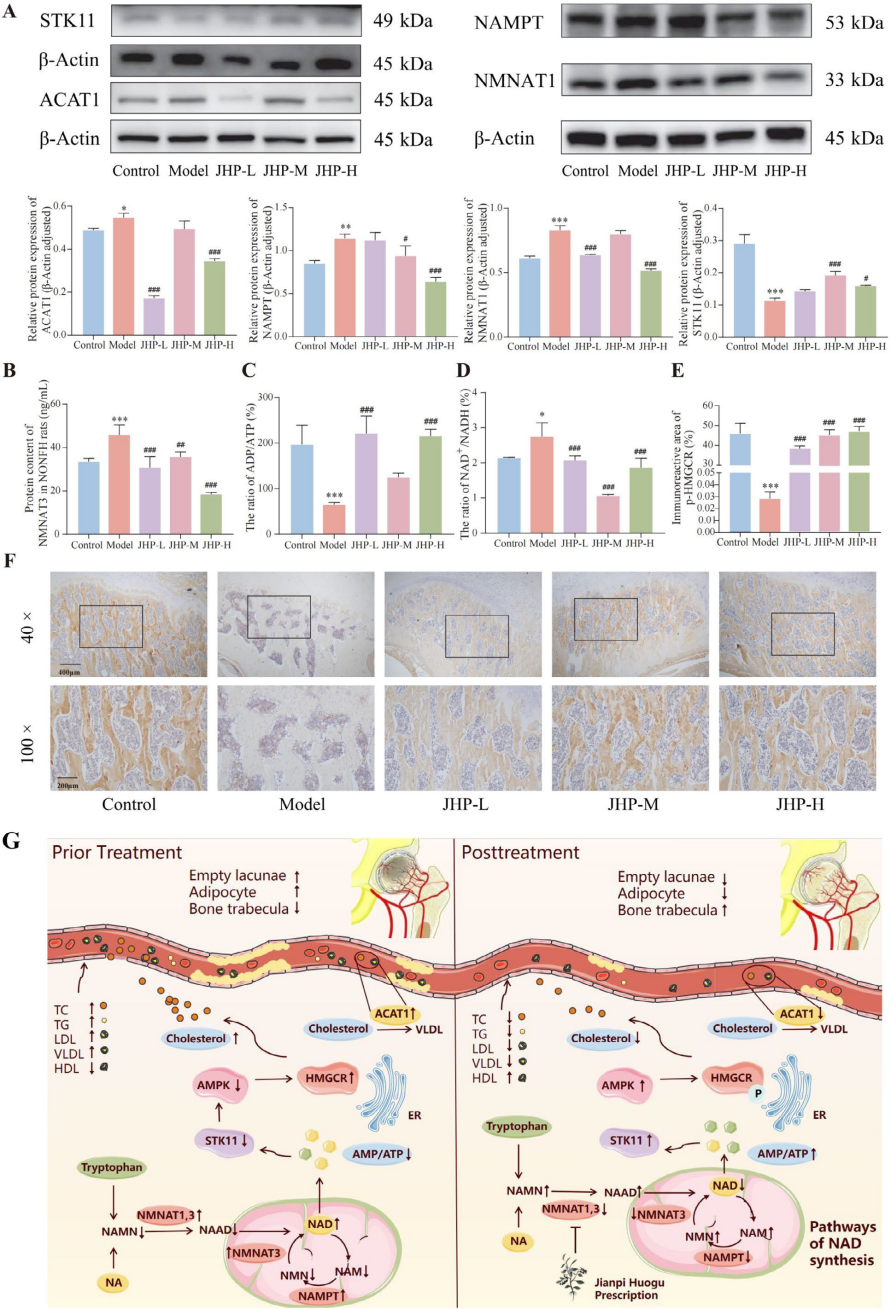
JHP promotes the repair of early NONFH through regulating NAMPT/STK11/HMGCR/ACAT1 signal axis (*n* = 3). (A) Expression levels of STK11, ACAT1, NAMPT and NMNAT1 proteins in femoral head tissues of different groups detected by western blot; (B) NMNAT3 content, (C) ADP/ATP ratio and (D) NAD+/NADH ratio in femoral head tissues of different groups detected by ELISA; (E) and (F) Statistical results and representative images on immunoreactive area of p‐HMGCR protein in femoral head tissues of different groups detected by immunohistochemistry. (G) Illustration of the pharmacological mechanisms of JHP against early NONFH. *p < 0.05, **p < 0.01, ***p < 0.001, vs. control group; ^#^ *p* < 0.05, ^##^ *p* < 0.01, ^###^ *p* < 0.001, vs. model group. ADP, adenosine diphosphate; AMPK, protein kinase AMP‐activated catalytic subunit; ATP, adenosine triphosphate; NAD+, nicotinamide adenine dinucleotide; NADH, nicotinamide adenine dinucleotide; p‐HMGCR, Phospho‐HMGCR; WST‐8, 2‐(2‐Methoxy‐4‐nitrophenyl)‐3‐(4‐nitrophenyl)‐5‐(2,4‐disulfophenyl)‐2H‐tetrazolium Sodium Salt.

Compared with those in normal rats, the expression levels of NMNAT1, NAMPT, NMNAT3, and ACAT1 proteins in the femoral head of early NONFH rats were all significantly increased. In contrast, those of STK11 protein and the ADP/ATP ratio were dramatically decreased, all of which were effectively reversed by JHP treatment (all *p* < 0.05, Figure 5A–C). Immunohistochemical staining revealed minimal positive expression of p‐HMGCR protein in the femoral heads of rats in the model group. In contrast, the positive expression area of p‐HMGCR protein in each JHP dose group was significantly increased (*p < 0.05*, Figure 5E,F). The WST‐8 assay results indicated that the NAD^+^/NADH ratio in the Model group was significantly higher than that in the control group, whereas it was significantly lower in each dose group than in the control (*p < 0.05*; Figure 5D). The ELISA detection results showed that the serum levels of NAMN, NAAD, NMN, and NAM in each dose group were significantly higher than those in the model group (*p < 0.05*; Figure S4). These findings demonstrate that JHP may reduce the level of NAD+ in the mitochondrial electron transport chain by inhibiting the key enzymes NMNAT1 and NMNAT3 and the rate‐limiting enzyme NAMPT of NAD+ synthesis, leading to increased production of ATP during cell respiration. This promotes the combination of STK11 and AMPK, subsequently reducing HMGCR‐mediated cholesterol synthesis, ACAT1‐mediated cholesterol lipid accumulation, and blood lipids production, potentially promoting the repair of early NONFH (Figure 5G).

### 3.6 Active Components of JHP Directly Target NMNAT1 to Regulate the Core Signalling Axis

**FIGURE 6:**
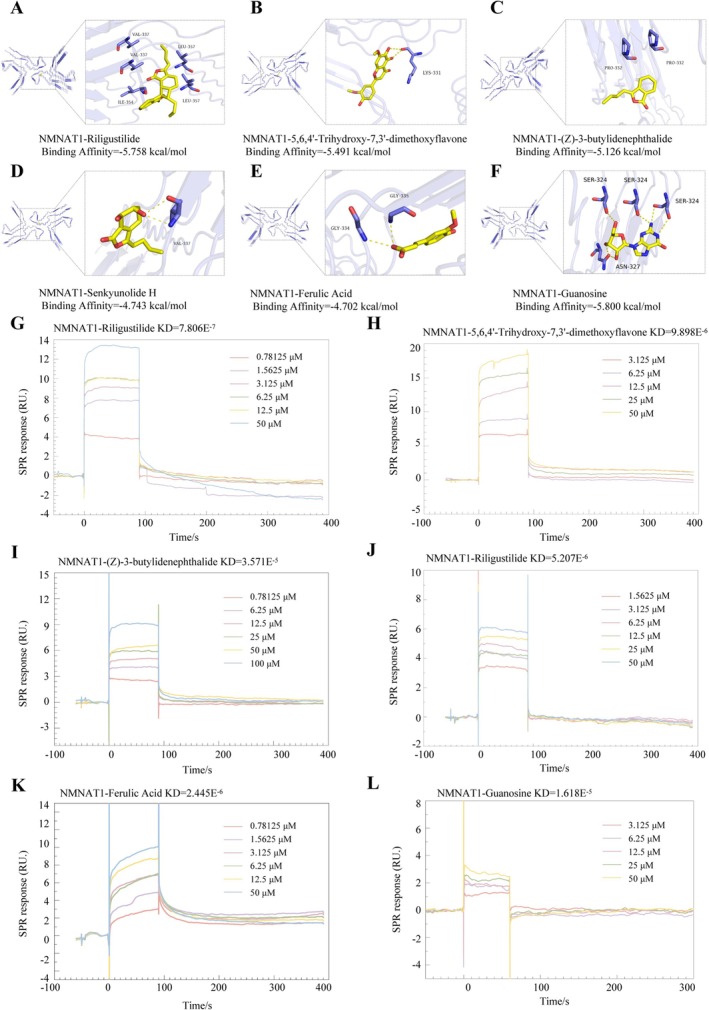
Active components of JHP directly target NMNAT1. (A–F) Molecular docking simulations predicted strong binding affinity between candidate compounds and NMNAT1. Ligustilide, 5,6,4′‐trihydroxy‐7,3′‐dimethoxyflavone, (Z)‐3‐butylidenephthalide, senkyunolide H, ferulic acid, and guanosine exhibited significant binding potential (docking scores < −4.5 kcal/mol). Protein NMNAT1 is shown in purple; compounds are in yellow. Predicted hydrogen bonds are indicated by yellow dashed lines. (G–L) Surface plasmon resonance (SPR) analysis confirmed direct binding of these compounds to NMNAT1.

Molecular docking simulations predicted strong binding affinity between key JHP constituents and NMNAT1, the upstream regulator of the NAMPT/STK11/HMGCR/ACAT1 signalling axis. Six compounds—ligustilide, 5,6,4′‐trihydroxy‐7,3′‐dimethoxyflavone, (Z)‐3‐butylidenephthalide, senkyunolide H, ferulic acid, and guanosine—exhibited significant binding potential (docking scores < −4.5 kcal/mol; Figure 6A–F). SPR analysis confirmed direct and specific binding of these compounds to NMNAT1 (Figure 6G–L). These findings identify NMNAT1 as a critical target for JHP's active components, demonstrating their role in mechanistically driving the regulation of the NMNAT1/NMNAT3/NAMPT/STK11/HMGCR/ACAT1 axis in early NONFH treatment.

## 4 Discussion

Accumulating clinical and basic research evidence shows that lipid metabolism disorders and blood hypercoagulability are vital pathological events of early NONFH and therapeutic targets of the disease [24]. As a modified version of Linggui‐Zhugan Decoction and Si‐wu Decoction, JHP has demonstrated satisfactory efficacy in NONFH therapy at an early stage [25]. However, the material basis and pharmacological mechanisms of JHP remain unclear, potentially affecting its clinical application. We identified terpenoids, glycosides, flavonoids, lipids, and phenolic acids as the main chemical components of JHP. Additionally, JHP effectively ameliorated histopathological abnormalities, inflammatory responses, and structural damage to the femoral head. Mechanistically, the therapeutic effects were associated with JHP's role in suppressing the enzymatic activities of NAMPT, NMNAT1, and NMNAT3, the reduction of NAD+ synthesis and its mediated ATP synthesis, and the activation of STK11. This inhibits the phosphorylation of HMGCR‐mediated cholesterol synthesis and ACAT1‐mediated cholesterol ester synthesis. To our knowledge, this is the first study to identify the chemical profile of JHP and highlight its major pharmacological properties in treating early NONFH by reversing blood hypercoagulability mediated by lipid metabolism disorders during disease occurrence and progression.

Based on our chemical profiling data, JHP mainly consists of terpenoids, glycosides, flavonoids, lipids, phenolic acids, and other components. Modern pharmacological studies reveal that tetracyclic triterpenes from FL, such as ganoderic acid B (compound 141) and tumulosic acid (compound 263), may mediate cholesterol efflux and inhibit macrophage inflammation [26, 27]. Glycosides in CS, such as paeoniflorin (compound 179), may reduce the production of inflammatory mediators, alleviate bone and joint damage [28], and mediate neurotransmitter release, thereby regulating nerve signal transduction [29]. Flavonoid glycosides, such as luteolin‐7‐O‐rutinoside (compound 168) and eriocitrin (compound 199), in CBZ, may scavenge oxygen free radicals [30] and improve dyslipidemia [31]. Lactone compounds, such as senkyunolide Q (compound 293), senkyunolide G (compound 272), and senkyunolide J (compound 213), in CX may relieve neuroinflammation and inhibit osteoarthritis [32]. Phenolic acids, including chlorogenic acid (compound 139), neochlorogenic acid (compound 96), and crypto‐chlorogenic acid (compound 147), in DG may reduce lipid accumulation [33] and inhibit microglial inflammation [34, 35]. These findings suggest that JHP regulates lipid metabolism, inflammation, and abnormal bone metabolism, potentially contributing to the pathogenesis of early NONFH. Critically, our SPR analysis confirmed that six key active components—ligustilide, 5,6,4′‐trihydroxy‐7,3′‐dimethoxyflavone, (Z)‐3‐butylidenephthalide, senkyunolide H, ferulic acid, and guanosine—directly bind to NMNAT1, the upstream regulator of the identified signalling axis. This provides molecular evidence that JHP's chemical constituents mechanistically drive the regulation of the NMNAT1/NMNAT3/NAMPT/STK11/HMGCR/ACAT1 axis.

Notably, our previous 2–3‐year follow‐up study based on clinical cohorts treated with JHP or core decompression combined with bone grafting (the common surgical method for international treatment of NONFH) revealed that the image stabilisation rate of the JHP treatment group was 74.47%, like that of the operation treatment group (75.00%) [25, 36]. In addition, the JHP treatment group displayed a higher Harris score for the hip joints (95.74%) than the operation treatment group (79.17%), indicating that JHP may improve hip joint function in four aspects: pain degree, joint deformity, range of motion, and overall function. Importantly, patients in the JHP treatment group reported better quality of life than those in the operation treatment group in terms of physiological function, body pain, social function, and emotional well‐being.

Consistently, our data in this study indicated that the ratio of RANKL to OPG, which determines the activity of osteoclasts, was increased in early NONFH rats, suggesting the differentiation of osteoclasts and enhancement of bone resorption capacity [37]. In addition, H&E staining showed a decreased proportion of calcification of the femoral head tissues, rough bone trabeculae, and increased empty bone lacunae in the model group, indicating obvious abnormal bone metabolism in the early NONFH rats, aligning with a previous study [38]. Following treatment with JHP, disease severity in NONFH rats effectively improved.

The pathogenesis of early NONFH is complex, with vascular stenosis caused by hypercoagulable blood being one of its important factors in NONFH [39]. The ratio of platelet movement related index TXB2/6‐keto‐PGF1α can directly reflect the blood viscosity [40]. Consistently, our data also indicated that JHP reduced the ratio of TXB2/6‐keto‐PGF1α in early NONFH rats and improved their blood hypercoagulable status. Lipid metabolism disorder is the initial factor in the blood hypercoagulable state, and lipid deposition forms a fat thrombus in blood vessels, leading to intraosseous vascular microcirculation disturbance, reducing the blood supply to the femoral head and subsequently resulting in femoral head necrosis [20]. Accordingly, we found that serum TC, TG, LDL, and VLDL levels were markedly increased, whereas HDL levels were distinctly decreased in early NONFH rats. Histopathological examination revealed an increase in the number of adipocytes in the bone marrow of rats with early NONFH, accompanied by severe steatosis in the liver tissue. These findings indicate significant abnormalities in lipid metabolism in rats with early NONFH. After treatment with JHP, the indices tended to normalise, indicating that JHP effectively improved lipid metabolism disorders in early NONFH rats. Previous studies have reported the inseparable relationship between joint pain and inflammatory factors during the progression of early NONFH [2]. Accordingly, our data showed that JHP effectively expanded the mechanical pain area and restored the normal gait in rats by reducing serum levels of inflammatory factors such as IL‐1β, IL‐6, and TNF‐α.

Under physiological conditions, the transformation of NAD^+^ to NADH plays a crucial role in ATP production during cellular respiration [41]. NAMPT and NMNAT are indispensable components of NAD^+^ synthesis. NAMPT serves as the rate‐limiting enzyme of NAD^+^ synthesis, while NMNAT, which comprises NMNAT1 and NMNAT3 subtypes, is responsible for NAD^+^ synthesis [42]. Additionally, NAMPT has been identified as a new biomarker for assessing the clinical severity of NONFH disease [43]. This study data showed that NAMPT was an early NONFH‐related disease gene and a drug target for JHP treatment, along with its associated genes NMNAT1, NMNAT3, and HMGCR. Experimentally, JHP treatment effectively reduced intracellular NAD^+^ levels and ATP production but increased the proportion of ADP/ATP in early NONFH rats. The direct binding of JHP components to NMNAT1 (as validated by SPR) underpins this suppression of NAD^+^ synthesis. Subsequently, the downstream kinase STK11 inhibits HMGCR‐mediated cholesterol synthesis and reduces the cholesterol content in the body upon sensing an increase in the ADP/ATP ratio. This reduction in cholesterol content positively regulates cholesterol esters synthesis mediated by ACAT1, consequently reducing cholesterol esters accumulation in the liver and blood and alleviating the blood hypercoagulable state and ameliorating symptoms of early NONFH.

## 5 Conclusion

This study provides convincing evidence that JHP may partially ameliorate early NONFH by restoring the balance of lipid metabolism disorder and reversing pathological events during early NONFH progression by regulating the NAMPT/STK11/HMGCR/ACAT1 axis. These findings provide an experimental basis for the scientific understanding and clinical application of JHP in early‐stage NONFH.

## Author Contributions

**Tao Li:** methodology (equal), validation (equal), writing – original draft (equal). **Zhaochen Ma:** methodology (equal), supervision (equal), writing – original draft (equal). **Shuangrong Gao:** data curation (equal), validation (equal), visualization (equal). **Chu Zhang:** data curation (equal), validation (equal), visualization (equal). **Yan Jia:** data curation (equal), validation (equal), visualization (equal). **Huang Feng:** data curation (equal), supervision (equal), visualization (equal). **Na Lin:** funding acquisition (equal), methodology (equal), writing – review and editing (equal). **Weiheng Chen:** funding acquisition (equal), project administration (equal), writing – review and editing (equal). **Yanqiong Zhang:** funding acquisition (equal), methodology (equal), writing – review and editing (equal).

## Ethics Statement

This study was approved by the Research Ethics Committee of the Institute of Chinese Materia Media, Wangjing Hospital, China (Approval number: 81473695) and the Third Affiliated Hospital of Beijing University of Chinese Medicine (approval number: BZYSY‐2021KYKTPJ‐01). Informed consent was obtained from all the patients. All animal experiments were approved by the Experimental Animal Ethics Committee of the Institute of Chinese Materia Medica, China Academy of Chinese Medical Sciences, Beijing, China (Ethics Approval Number: 2022B086), and were performed in accordance with international guidelines on the ethical use of animals.

## Consent

All enrolled patients completed the corresponding Case Report Form (CFR) stage and consent for publication.

## Conflicts of Interest

The authors declare no conflicts of interest.

## Supporting information


**Figure S1:** Representative chemical constituents identified from JHP and their corresponding biological activities.
**Figure S2:** Multi‐level interaction network of herbs and chemical constituents containing in JHP, drug putative and effective targets, enriched pathways, and symptoms of early NONFH. In the Herb and Compound plates, the compounds are corresponding to the colours of the herbs from which it originated. In the Target plate, orange nodes represent early NONFH differential genes, in which triangles and arrows represent upregulated and down‐regulated genes, blue nodes represent early NONFH symptom‐related genes, pink nodes represent predictive targets of JHP, mixed colour nodes represent common genes, and green nodes represent JHP efficacy‐related genes. In the pathway and symptom plates, the colour of the pathway is consistent with the colour of the related symptoms, and in the symptom plate, the mixed colour node indicates that the node corresponds to multiple pathways at the same time. ACAT1, Acetyl‐CoA Acetyltransferase 1; HMGCR, 3‐Hydroxy‐3‐Methylglutaryl‐CoA Reductase; HPO, The Human Phenotype Ontology; mRNA‐Seq, Messenger Ribonucleic Acid sequencing; NMNAT1, Nicotinamide Nucleotide Adenylyl transferase 1; NMNAT3, Nicotinamide Nucleotide Adenylyl transferase 3; NAMPT, Nicotinamide Phosphoribosyl transferase; SFDA, State Food and Drug Administration; STK11, Serine/Threonine Kinase 11; ETCM 2.0.
**Figure S3:** Results of association of omics data with clinical efficacy and animal efficacy.
**Figure S4:** JHP increases the content of NAMN, NAAD, NMN and NAM in the NAD+ remediation pathway. (A‐D) The contents of serum NAMN, NAAD, NMN and NAM in rats. NAMN, Nicotinate mononucleotide; NAAD, Deamido nad; NMN, β‐Nicotinamide Mononucleotide; NAM, Nicotinamide.

**Appendix S1:** Supplementary Tables .

**Appendix S2:** Supplementary Methods

## Data Availability

The original contributions proposed in the study are stored in articles and Supporting Information, and further inquiries can be made directly to the corresponding author. The data that support the findings of this study are available in NCBI – GEO database at [https://www.ncbi.nlm.nih.gov/geo/query/acc.cgi?acc=GSE254972], reference number [GSE254972].
