# Supplementary material for: Jianpi‐Huogu Prescription Repairs Nontraumatic Osteonecrosis of the Femoral Head by Inhibiting NAMPT/STK11/HMGCR/ACAT1 Axis‐Mediated Lipid Production
Source: J Cell Mol Med. 2025 Sep 23;29(18):e70858. doi: 10.1111/jcmm.70858 (PMC12457209; doi:10.1111/jcmm.70858)
Supplement: Supplementary file 1 — Figure S1: Representative chemical constituents identified from JHP and their corresponding biological activities. Figure S2: Multi‐level interaction network of herbs and chemical constituents containing in JHP, drug putative and effective targets, enriched pathways, and symptoms of early NONFH. In the Herb and Compound plates, the compounds are corresponding to the colours of the herbs from which it originated. In the Target plate, orange nodes represent early NONFH differential genes, in which triangles and arrows represent upregulated and down‐regulated genes, blue nodes represent early NONFH symptom‐related genes, pink nodes represent predictive targets of JHP, mixed colour nodes represent common genes, and green nodes represent JHP efficacy‐related genes. In the pathway and symptom plates, the colour of the pathway is consistent with the colour of the related symptoms, and in the symptom plate, the mixed colour node indicates that the node corresponds to multiple pathways at the same time. ACAT1, Acetyl‐CoA Acetyltransferase 1; HMGCR, 3‐Hydroxy‐3‐Methylglutaryl‐CoA Reductase; HPO, The Human Phenotype Ontology; mRNA‐Seq, Messenger Ribonucleic Acid sequencing; NMNAT1, Nicotinamide Nucleotide Adenylyl transferase 1; NMNAT3, Nicotinamide Nucleotide Adenylyl transferase 3; NAMPT, Nicotinamide Phosphoribosyl transferase; SFDA, State Food and Drug Administration; STK11, Serine/Threonine Kinase 11; ETCM 2.0. Figure S3: Results of association of omics data with clinical efficacy and animal efficacy. Figure S4: JHP increases the content of NAMN, NAAD, NMN and NAM in the NAD+ remediation pathway. (A‐D) The contents of serum NAMN, NAAD, NMN and NAM in rats. NAMN, Nicotinate mononucleotide; NAAD, Deamido nad; NMN, β‐Nicotinamide Mononucleotide; NAM, Nicotinamide. [file JCMM-29-e70858-s003.docx]

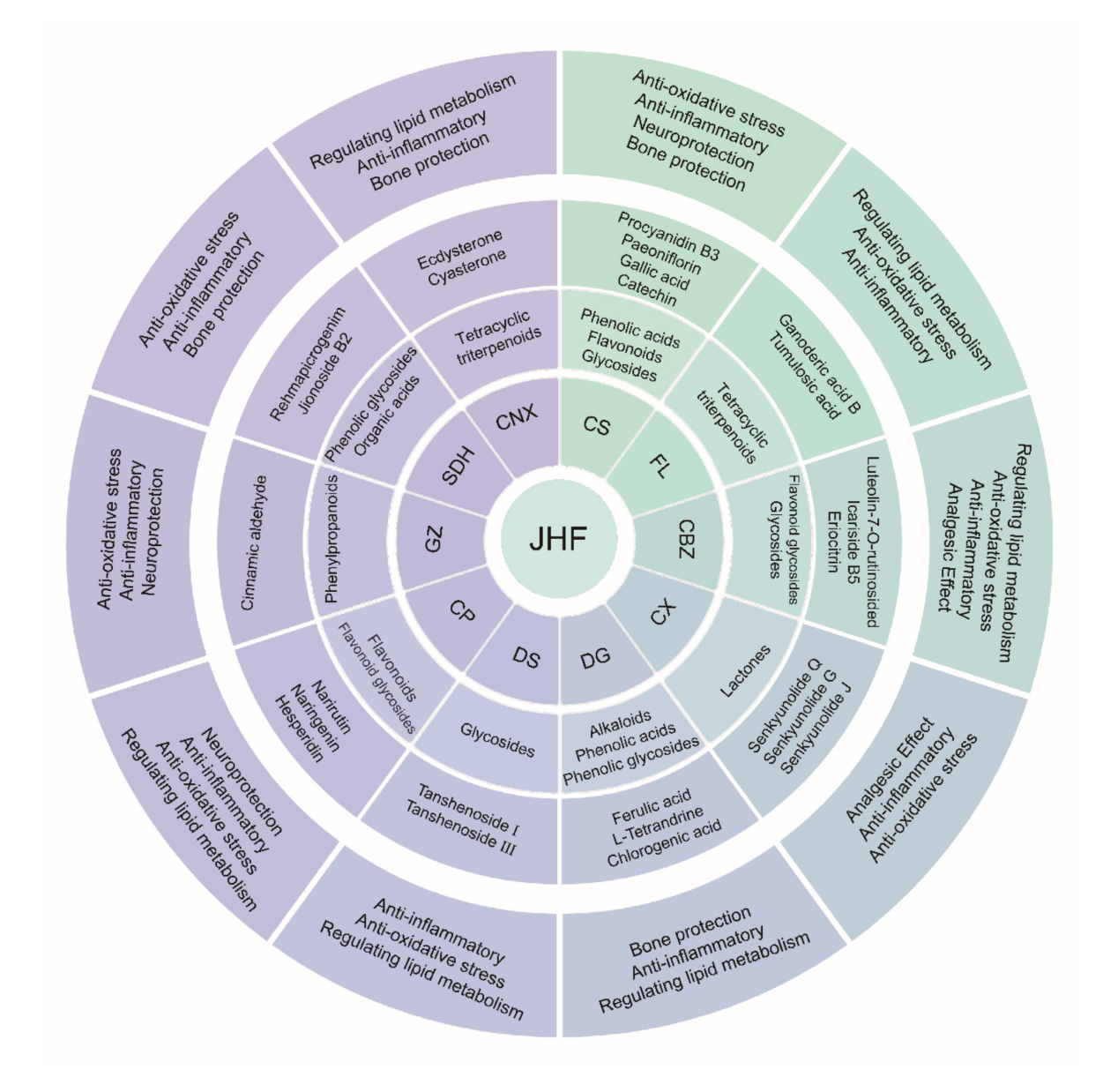


**Figure S1. Representative chemical constituents identified from JHP and their corresponding biological activities.**

**
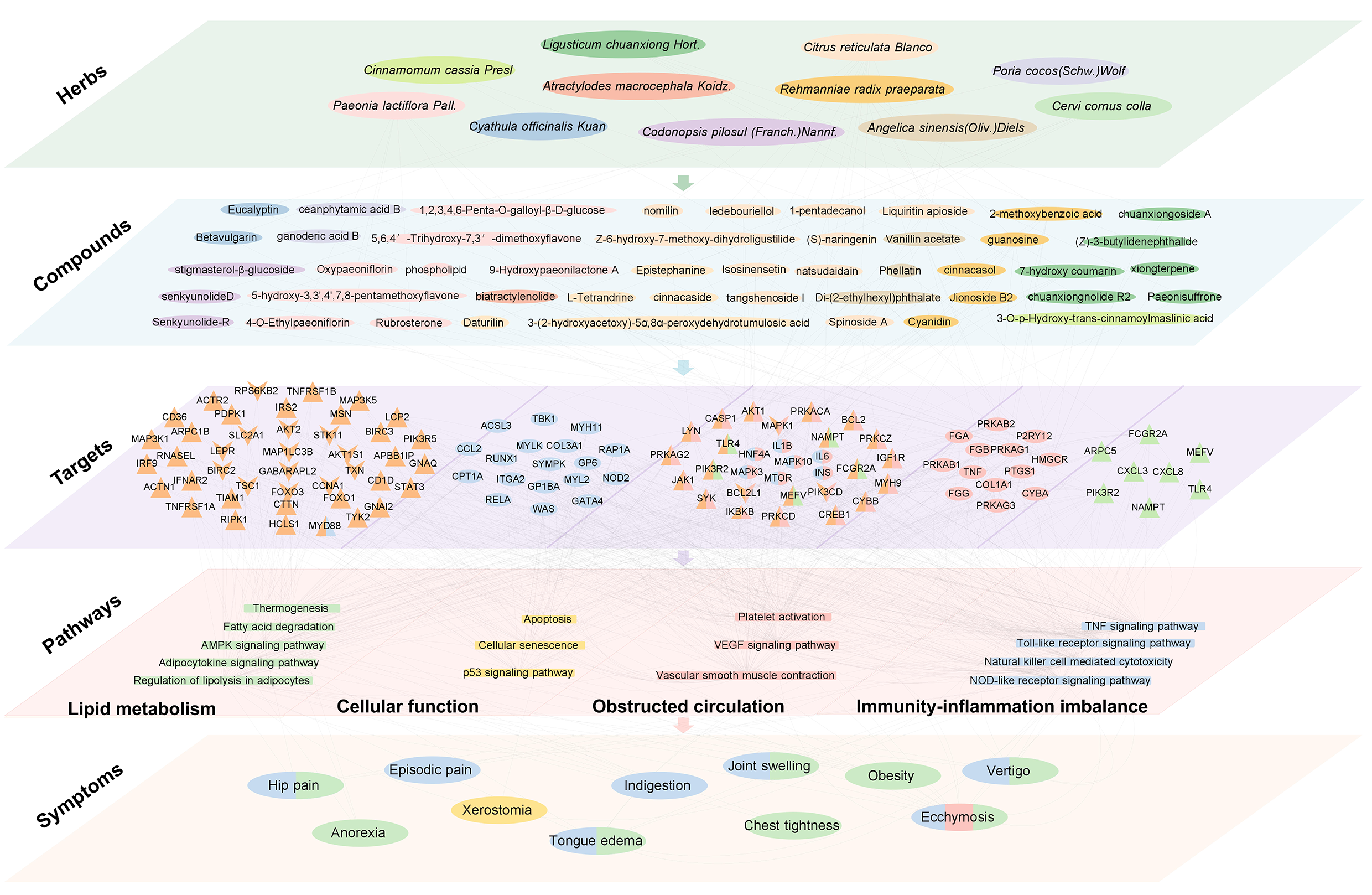
**

**Figure S2. Multi-level interaction network of herbs and chemical constituents containing in JHP, drug putative and effective targets, enriched pathways, and symptoms of early NONFH.** In the Herb and Compound plates, the compounds are corresponding to the colors of the herbs from which it originated. In the Target plate, orange nodes represent early NONFH differential genes, in which triangles and arrows represent upregulated and down-regulated genes, blue nodes represent early NONFH symptom-related genes, pink nodes represent predictive targets of JHP, mixed color nodes represent common genes, and green nodes represent JHP efficacy-related genes. In the pathway and symptom plates, the color of the pathway is consistent with the color of the related symptoms, and in the symptom plate, the mixed color node indicates that the node corresponds to multiple pathways at the same time. ACAT1, Acetyl-CoA Acetyltransferase 1; HMGCR, 3-Hydroxy-3-Methylglutaryl-CoA Reductase; HPO, The Human Phenotype Ontology; mRNA-Seq, Messenger Ribonucleic Acid sequencing; NMNAT1, Nicotinamide Nucleotide Adenylyl transferase 1; NMNAT3, Nicotinamide Nucleotide Adenylyl transferase 3; NAMPT, Nicotinamide Phosphoribosyl transferase; SFDA, State Food and Drug Administration; STK11, Serine/Threonine Kinase 11; ETCM 2.0.


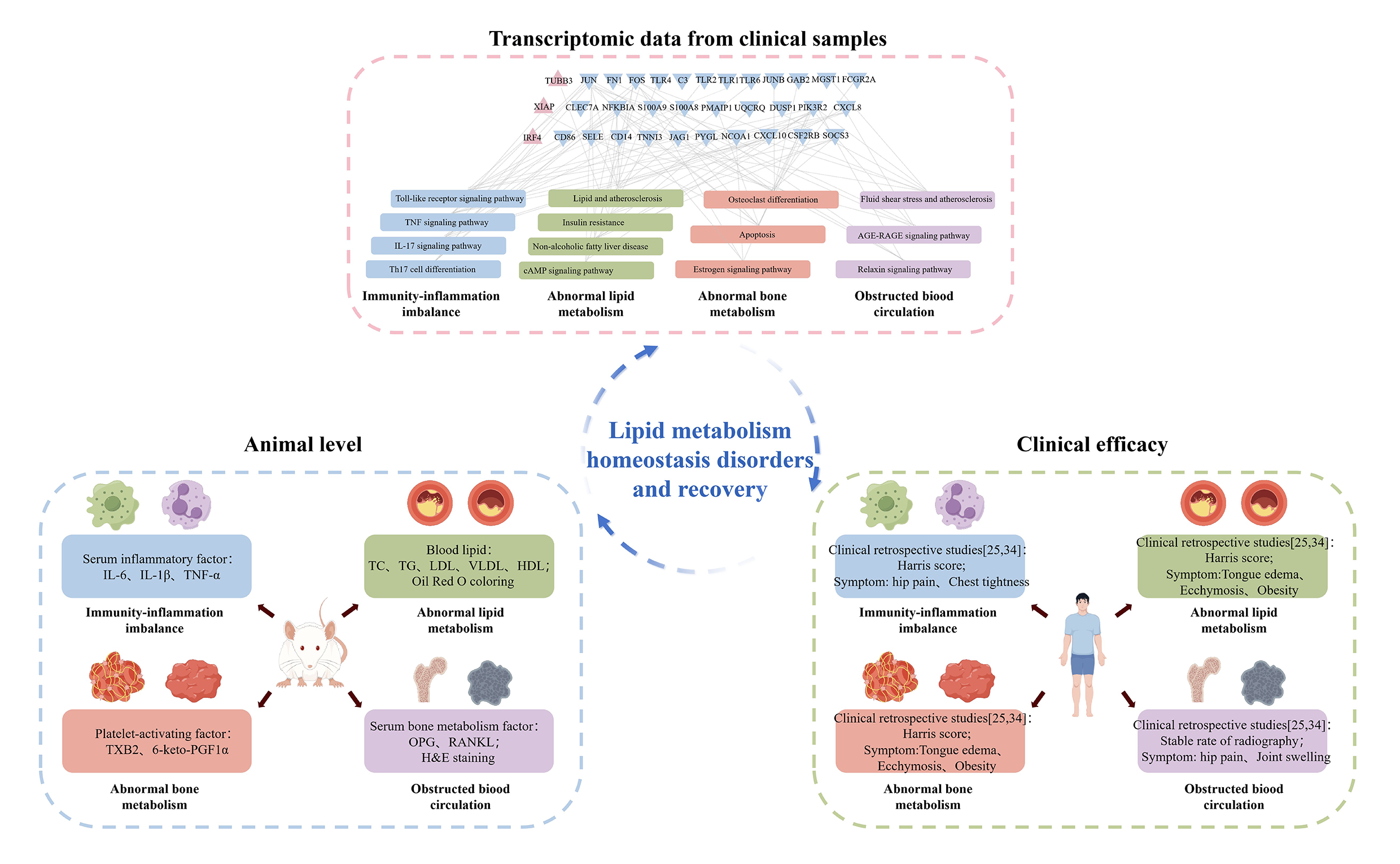


**Figure S3. Results of association of omics data with clinical efficacy and animal efficacy.**

**
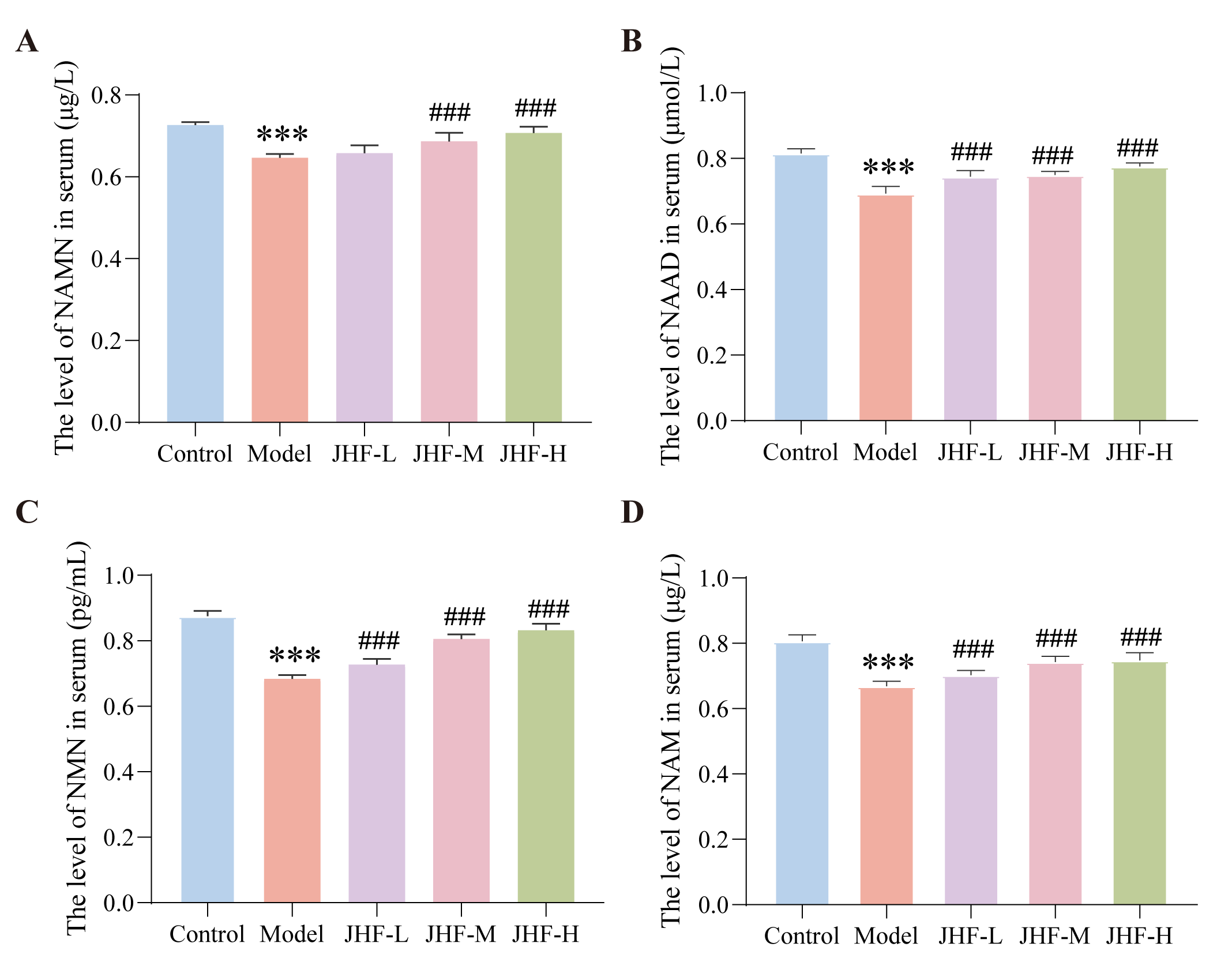
**

**Figure S4. JHP increases the content of NAMN, NAAD, NMN and NAM in the NAD+ remediation pathway.** (A-D) The contents of serum NAMN, NAAD, NMN and NAM in rats. NAMN, Nicotinate mononucleotide; NAAD, Deamido nad; NMN, β-Nicotinamide Mononucleotide; NAM, Nicotinamide.


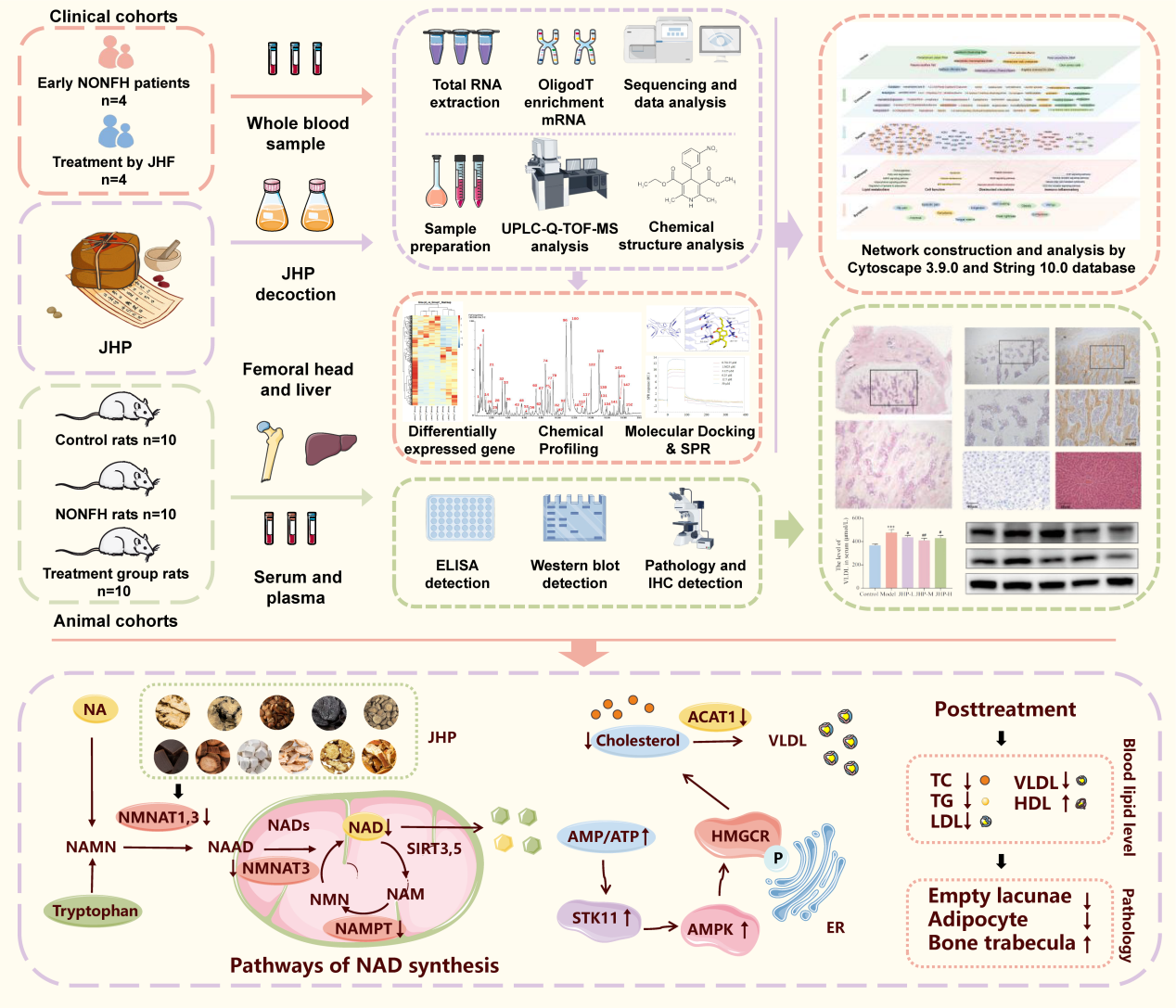


**Graphical abstract**

The main compound types of JHP were terpenoids, glycosides, flavonoids, lipids, phenolic acids. JHP was demonstrated to effectively ameliorate histopathological abnormalities, inflammation, and structural damage of the femoral head, through the inhibition of NAMPT, NMNAT1 and NMNAT3 enzyme activities, the reduction of NAD+ synthesis and its mediated ATP synthesis, and the activation of STK11, thus phosphorylation inhibition of HMGCR-mediated cholesterol synthesis and ACAT1-mediated cholesterol ester synthesis.
