# Supplementary material for: Jianpi‐Huogu Prescription Repairs Nontraumatic Osteonecrosis of the Femoral Head by Inhibiting NAMPT/STK11/HMGCR/ACAT1 Axis‐Mediated Lipid Production
Source: J Cell Mol Med. 2025 Sep 23;29(18):e70858. doi: 10.1111/jcmm.70858 (PMC12457209; doi:10.1111/jcmm.70858)
Supplement: Supplementary file 2 — Appendix S1: Supplementary Tables . [file JCMM-29-e70858-s002.pdf]

Table S1 Detailed information on chemical compounds contained in JPHGP using UPLC-Q-TOF-MS/MS system.

NO.	Component name	Observed m/z	Formula	Mass error (mDa)	Observed RT (min)	Response	Adduct	Fragment ions
1	1-pentadecanol	251.2364	$C_{15}H_{32}O$	1.9	0.48	9716	$[M+Na]^{+}$	251.2364
2	Vanillin acetate	251.0315	$C_{10}H_{12}O_5$	-0.2	0.53	38350	$[M+K]^{+}$	175.11955, 235.05623
3	arginine	173.1061	$C_6H_{14}N_4O_2$	1.7	0.54	37409	$[M-H]^{-}$	131.08453
4	polysaccharide	539.1396	$C_{18}H_{32}O_{16}$	1.2	0.58	116128	$[M+Cl]^{-}$	179.05800, 221.06861, 383.12055, 443.14260
5	n-Butyl allophanate	195.0526	$C_6H_{12}N_2O_3$	-1.6	0.59	288376	$[M+Cl]^{-}$	71.01510, 131.04764, 143.03700
6	L-valine	118.0869	$C_5H_{11}NO_2$	0.6	0.59	33493	$[M+H]^{+}$	84.04504, 87.04434, 102.05597
7	D-proline	116.0714	$C_5H_9NO_2$	0.8	0.63	42093	$[M+H]^{+}$	73.02933, 90.05582
8	trehalose	404.1059	$C_{12}H_{22}O_{11}$	1.4	0.65	722427	$[M+NO_3]^{-}$	127.04222, 191.05773
9	Paeoniflorin B	781.2167	$C_{36}H_{42}O_{17}$	5.1	0.66	22753	$[M+Cl]^{-}$	191.05773, 341.11008, 404.10595, 533.17287, 604.17336, 683.22583
10	tokinolide,b	377.1753	$C_{24}H_{26}O_4$	-0.6	0.66	9196	$[M-H]^{-}$	262.05885
11	melibiose	341.1099	$C_{12}H_{22}O_{11}$	1	0.69	452111	$[M-H]^{-}$	113.02613, 161.0474, 191.05773, 323.09999
12	Campheoside I	653.2148	$C_{30}H_{38}O_{16}$	6.1	0.77	14110	$[M-H]^{-}$	311.09973, 421.07163, 475.13305, 637.18433
13	Cinnamic aldehyde	150.092	C_9H_8O	0.7	0.89	717996	$[M+NH_4]^{+}$	77.03940, 91.05545, 103.05607
14	icariside I	575.1814	$C_{27}H_{30}O_{11}$	4.4	0.93	9986	$[M+HCOO]^{-}$	179.05778, 323.09955, 404.10601, 457.12160
15	senkyunolide D	240.123	$C_{12}H_{14}O_4$	0	1.03	60698	$[M+NH_4]^{+}$	77.03911, 124.99181, 196.07097
16	tangshenoside	581.1841	$C_{28}H_{34}O_{11}$	4.6	1.11	19635	$[M+Cl]^{-}$	330.08207, 499.13092, 530.22142

	IV deglycosylation							
17	Pyroglutamic acid N-fructoside	290.0895	$C_{11}H_{17}NO_8$	1.3	1.12	359735	$[M-H]^-$	84.04726, 128.03705, 200.05793, 254.06976, 272.08112
18	Paeonidanins F	470.1523	$C_{46}H_{54}O_{21}$	1.8	1.15	37879	$[M-2H]^-$	109.03118, 452.14204, 647.20413, 839.23309
19	Suffruticosol A	783.24	$C_{47}H_{40}O_9$	3.4	1.2	30205	$[M+Cl]^-$	251.07891, 281.08941, 383.12060
20	1-O-(4-hydroxy benzoyl)-β-D-ga lactopyranoside	332.1321	$C_{14}H_{18}O_8$	-1.9	1.22	129613	$[M+NH_4]^+$	97.02897, 109.02907, 127.03961, 145.05040, 163.06057, 181.05005, 223.06000, 253.07072
21	Pelargonidin 3-glucoside	596.1707	$C_{27}H_{31}O_{15}$	-2.9	1.23	16331	$[M+H]^+$	233.06000, 365.10224, 434.11821, 527.15311
22	4-methylcinnam ic acid	180.1019	$C_{10}H_{10}O_2$	0	1.32	187508	$[M+NH_4]^+$	105.07053, 117.07042, 133.06486
23	icariside D2	345.1195	$C_{14}H_{20}O_7$	0.4	1.33	27346	$[M+HCOO]^-$	117.02095, 165.05745
24	4-O-Methyl-4″-hydroxy-3″-met hoxy-paeoniflori n or its isomer	558.2169	$C_{25}H_{32}O_{13}$	-1.2	1.35	29760	$[M+NH_4]^+$	355.07315, 487.16540
25	Tyrosine or its isomer	182.0816	$C_9H_{11}NO_3$	0.5	1.36	47872	$[M+H]^+$	95.05012, 119.04981, 136.07591, 147.04576, 165.05557
26	L-Uridine	243.0636	$C_9H_{12}N_2O_6$	1.3	1.38	39213	$[M-H]^-$	99.01038, 117.02095, 128.03706, 169.01599, 180.06326, 200.05810, 230.06866
27	4-O-Galloylquin	343.0678	$C_{14}H_{16}O_{10}$	0.7	1.39	31303	$[M-H]^-$	125.02639, 147.03173, 169.01599,

	ic acid							175.02593, 191.05764, 239.06561, 271.01571, 299.03735
28	3-(4-Hydroxyphenyl)-trans-propenoic acid-2,3-dihydroxypropyl ester	326.1222	$C_{15}H_{16}O_7$	-1.2	1.4	78088	$[M+NH_4]^+$	130.05058, 185.03879, 245.05141
29	2,3-[(S)-Hexahydroxydiphenoyl]-D-glucose	481.0632	$C_{20}H_{18}O_{14}$	0.8	1.53	9511	$[M-H]^-$	125.02605, 169.01579, 257.01089, 275.02116, 301.00056
30	9-Hydroxypaeonilactone A	215.0925	$C_{10}H_{14}O_5$	1.1	1.64	16893	$[M+H]^+$	97.02892, 151.07665, 165.05744, 197.08166
31	Senkyunolide-R	258.1333	$C_{12}H_{16}O_5$	-0.3	1.72	39352	$[M+NH_4]^+$	99.04473, 127.03962, 135.04491
32	N-methyl phenylalanine	241.0841	$C_{10}H_{13}NO_2$	1.1	1.79	17370	$[M+NO_3]^-$	71.01486
33	Adenine nucleoside	312.0957	$C_{10}H_{13}N_5O_4$	0.7	1.8	8330	$[M+HCOO]^-$	97.03117, 125.02612, 155.05252, 212.05806, 241.08451
34	1-O-Galloyl-β-D-glucose	331.0674	$C_{13}H_{16}O_{10}$	0.3	1.8	109780	$[M-H]^-$	125.02612
35	guanosine	300.1198	$C_{10}H_{12}N_5O_5$	2.1	1.88	11936	$[M+NH_4]^+$	85.02876, 223.05992, 253.07080
36	Trimethyl citrate	252.1083	$C_9H_{14}O_7$	0.5	1.95	8657	$[M+NH_4]^+$	99.04467, 153.01856
37	7-oxo-15-hydroxydehydroabietic acid	353.1695	$C_{20}H_{26}O_4$	-2.9	2	23347	$[M+Na]^+$	110.03453, 166.09601, 254.16209, 284.09934

38	Butyl β-D-fructopyranoside or its isomer	254.1608	$C_{10}H_{20}O_6$	0.9	2	208781	$[M+NH_4]^+$	110.03453(C6H6O2),160.07630(C7H12O4)
39	2-Methyl-1-phenylpropene	155.082	$C_{10}H_{12}$	-1.1	2.05	130415	$[M+Na]^+$	155.08202
40	Gallic acid	169.0157	$C_7H_6O_5$	1.4	2.06	385278	$[M-H]^-$	125.02604
41	glucogallin or its isomer	331.0678	$C_{13}H_{16}O_{10}$	0.7	2.14	108376	$[M-H]^-$	143.03699, 179.05763, 271.04749
42	xiongterpene	613.3301	$C_{37}H_{50}O_5$	1.1	2.2	9013	$[M+K]^+$	165.05795
43	8-Debenzoylpaeoniflorin	438.1258	$C_{16}H_{24}O_{10}$	0.5	2.24	77170	$[M+NO_3]^-$	109.03152, 155.03668, 164.07376, 309.12011, 329.08871
44	Paeonisuffral	215.0917	$C_{10}H_{14}O_5$	0.3	2.24	10202	$[M+H]^+$	123.04427, 151.07634, 165.05795, 182.08574
45	L-Valine-L-valine anhydride	217.1547	$C_{10}H_{20}N_2O_3$	0.1	2.34	11504	$[M+H]^+$	100.07695, 115.04000
46	2-(hydroxymethyl)-4-oxo-4H-pyran-3-yl-6-O-α-L-rhamnopyranosyl-β-D-glucopyranoside	512.1259	$C_{18}H_{26}O_{13}$	0.2	2.45	85110	$[M+NO_3]^-$	111.01055, 125.02607, 169.01569, 173.00816, 279.07271, 325.10920, 331.06791, 405.14104
47	1-O-β-D-Glucopyranosyl-paeonisuffrone or its isomer	422.1308	$C_{16}H_{24}O_9$	0.4	2.46	77668	$[M+NO_3]^-$	113.02630, 125.02607, 299.07787

48	Paeonidanin B	664.2286	$C_{31}H_{34}O_{15}$	5	2.46	30615	$[M+NH_4]^+$	109.02923, 127.03970, 296.09429, 421.14323
49	Butyl β-D-fructopyranoside or its isomer	254.161	$C_{10}H_{20}O_6$	1.2	2.48	356021	$[M+NH_4]^+$	117.05819, 132.08142, 152.05943, 195.11364
50	Butyl β-D-fructopyranoside	281.1267	$C_{10}H_{20}O_6$	2.5	2.53	27049	$[M+HCOO]^-$	144.04687, 191.05745
51	14(alpha-Methylbutyryl)-2E,8E,10E-atractylentriol	339.156	$C_{19}H_{24}O_4$	-0.7	2.57	10571	$[M+Na]^+$	127.03986, 167.07287
52	glucogallin or its isomer	331.0681	$C_{13}H_{16}O_{10}$	1	2.61	47736	$[M-H]^-$	125.02635, 169.01603, 180.06821, 241.03733
53	3β-acetoxy-atractylone	297.1449	$C_{17}H_{22}O_3$	-1.2	2.61	16929	$[M+Na]^+$	123.04585, 202.09359
54	3-O-p-Hydroxy-trans-cinnamoyl maslinic acid	657.3571	$C_{39}H_{54}O_6$	1.9	2.71	10049	$[M+K]^+$	273.1452
55	1-O-β-D-Glucopyranosyl-paeonisuffrone or its isomer	422.1314	$C_{16}H_{24}O_9$	1	2.72	161515	$[M+NO_3]^-$	164.07383, 262.05646
56	Trichoderic acid	273.1449	$C_{15}H_{22}O_3$	-1.3	2.72	17256	$[M+Na]^+$	149.05854
57	sec-O-glucosylh	456.1867	$C_{21}H_{26}O_{10}$	0.3	2.74	8068	$[M+NH_4]^+$	145.05064, 172.07758, 200.07277,

	amaudol							383.13654
58	Cyanidin	305.0882	$C_{15}H_{11}O_6$	-1.2	2.79	3512	$[M+NH_4]^+$	109.02951
59	3,5-dihydroxyphenyl beta-D-glucopyranoside	333.0835	$C_{12}H_{16}O_8$	0.8	2.82	12562	$[M+HCOO]^-$	140.01370, 157.01163, 198.00320, 254.08497, 295.10525, 303.07363
60	Tyrosine or its isomer	199.1081	$C_9H_{11}NO_3$	0.4	3.01	43655	$[M+NH_4]^+$	89.03947, 147.04655
61	penicitrinol C	326.1259	$C_{15}H_{20}O_4$	1.4	3.05	16312	$[M+NO_3]^-$	236.09649
62	Epoxydihydrolinalool	211.1081	$C_{10}H_{20}O_2$	-1.4	3.18	180901	$[M+K]^+$	211.10806
63	5-O-Galloylquinic acid	343.0683	$C_{14}H_{16}O_{10}$	1.2	3.19	49547	$[M-H]^-$	93.03639, 191.05815, 297.06458
64	6'-O-Galloylsucrose	493.1209	$C_{19}H_{26}O_{15}$	1	3.3	361356	$[M-H]^-$	107.01557, 125.02624, 169.01607, 283.04766, 313.05800, 446.15229
65	(E)-cinnamyl acetate	194.118	$C_{11}H_{12}O_2$	0.4	3.4	48709	$[M+NH_4]^+$	117.06884
66	verbasoside	480.2074	$C_{20}H_{30}O_{12}$	-0.1	3.53	2635	$[M+NH_4]^+$	455.12824, 365.10566, 325.11398, 311.07625, 164.07603
67	Codonopsine	268.1547	$C_{14}H_{21}NO_4$	0.4	3.61	1126807	$[M+H]^+$	115.05598, 146.03641, 161.06054
68	1'-O-Galloylsucrose	493.1214	$C_{19}H_{26}O_{15}$	1.5	3.63	338730	$[M-H]^-$	107.01572, 125.02634, 169.01625, 271.04799, 313.05845, 331.06912, 402.10696
69	Protocatechuic acid-3-glucoside or its isomer	315.0736	$C_{13}H_{16}O_9$	1.5	3.71	150931	$[M-H]^-$	108.02339, 131.03791, 152.01343, 164.07527, 249.08135

70	3-O-β-D-glucopyranosyl-4-methoxyacetophenone	373.1152	$C_{15}H_{20}O_8$	1.2	3.78	13376	$[M+HCOO]^-$	143.04364, 160.00981, 254.07980, 272.09165
71	ethyl-β-D-fructofuranoside	359.1812	$C_{16}H_{32}O_6$	-1.8	3.8	22032	$[M+K]^+$	134.09805, 174.09250, 212.10733
72	6-O-Galloylsucrose	493.1212	$C_{19}H_{26}O_{15}$	1.3	3.84	162203	$[M-H]^-$	125.02638, 169.01621, 221.06847, 313.05834, 445.09560
73	12(alpha-Methylbutyryl)-14-acetyl-2E,8E,10E-atractylentriol	359.182	$C_{21}H_{26}O_5$	-3.3	3.97	41486	$[M+H]^+$	103.05526, 184.07695, 224.10496
74	Syringic acid	243.0529	$C_9H_{10}O_5$	1.9	4.02	18594	$[M+HCOO]^-$	123.04722, 135.04758, 151.04328, 167.03844, 179.03700
75	4-O-Ethylpaeoniflorin	547.1631	$C_{25}H_{32}O_{11}$	5.4	4.03	25989	$[M+K]^+$	147.04533, 361.10252, 495.21668
76	5-hydroxy-6-methyl-1H-indole-3-carbaldehyde	176.0711	$C_{10}H_9NO_2$	0.5	4.11	46281	$[M+H]^+$	130.06690, 133.05388, 147.06889
77	L-valyl-L-valinc-achydride	253.1298	$C_{11}H_{22}N_2O_2$	-1.5	4.11	92776	$[M+K]^+$	253.12978
78	2,3,3-trimethyl-2-[(Z)-3-methyl-1,3-butadien-1-yl]cyclohexanone	229.1553	$C_{14}H_{22}O$	-1	4.16	9972	$[M+Na]^+$	229.15533
79	2-methoxybenzo	170.082	$C_8H_8O_3$	0.8	4.25	30750	$[M+NH_4]^+$	170.08196

	ic acid							
80	(1R)-2,3,4,9-tetrahydro-1H-$b-carboline-1-carboxylic acid	238.0718	$C_{12}H_{11}N_2O_2$	0.5	4.31	10933	$[M+Na]^+$	170.05971
81	Paeonin B	403.1256	$C_{16}H_{22}O_9$	1.1	4.4	9182	$[M+HCOO]^-$	271.06408
82	4-buta-1,3-dienyl-3,5,5-trimethylcyclohex-2-en-1-one	213.1239	$C_{13}H_{18}O$	-1.1	4.64	44169	$[M+Na]^+$	175.12088
83	Pyridylpaeoniflorin	543.1473	$C_{22}H_{27}NO_{11}$	0.6	4.7	15850	$[M+NO_3]^-$	122.02712, 323.08468, 373.27597
84	tangshenoside V or its isomer	515.1409	$C_{21}H_{26}O_{12}$	0.3	4.76	9057	$[M+HCOO]^-$	305.07277
85	Paeonisuffrone	199.0971	$C_{10}H_{14}O_4$	0.6	4.82	45682	$[M+H]^+$	94.04067, 119.05070, 147.04537
86	Glucopaeonol	373.1148	$C_{15}H_{20}O_8$	0.8	4.87	29022	$[M+HCOO]^-$	134.04357, 135.04700, 161.02606, 179.03692, 217.01937, 247.02573, 261.03826, 264.09922
87	Protocatechuic acid-3-glucoside or its isomer	315.0733	$C_{13}H_{16}O_9$	1.2	4.95	11297	$[M-H]^-$	109.03150, 153.02166
88	2-Methyl-N-phenylmaleimide	188.0712	$C_{11}H_9NO_2$	0.6	5.02	521631	$[M+H]^+$	146.06094
89	Senkyunolide C	203.0839	$C_{10}H_{16}O_2$	-0.5	5.07	100186	$[M-H]^-$	185.01079, 171.00085, 160.04223, 146.96781, 142.03805, 130.06773, 116.05231, 101.02598, 80.96658,

								71.05113, 61.98935
90	Cocaine	365.1364	$C_{17}H_{21}NO_4$	1	5.1	13858	$[M+NO_3]^-$	130.06773, 142.06805, 157.07834, 177.00085
91	1-O-β-D-glucopyranosyl-8-O-benzoylpaeonisuffrone	487.1552	$C_{23}H_{28}O_{10}$	-2.3	5.26	5271	$[M+Na]^+$	151.03939, 425.18371
92	Citronellol	195.1132	$C_{1}0H_{20}O$	-1.4	5.29	62078	$[M+K]^+$	74.06093, 98.06158, 111.09253, 139.08746
93	Paeonicluside	461.1309	$C_{18}H_{24}O_{11}$	0.8	5.38	8559	$[M+HCOO]^-$	108.02305, 236.05961
94	6′-O-Galloyldesbenzoylpaeoniflorin or its isomer	527.141	$C_{23}H_{28}O_{14}$	0.4	5.51	12080	$[M-H]^-$	125.02622, 169.01611, 313.05955
95	1-beta-ethylacrylate-7-aldehyde-beta-carboline	355.0797	$C_{17}H_{13}N_2O_3$	-1.3	5.56	23357	$[M+NO_3]^-$	180.07249
96	neochlorogenic acid	353.0884	$C_{16}H_{18}O_9$	0.6	5.62	150727	$[M-H]^-$	135.0468, 161.02595, 179.03674, 191.05764, 264.10101, 307.08404
97	mudanpioside G	406.1361	$C_{16}H_{24}O_8$	0.6	5.63	20561	$[M+NO_3]^-$	135.04680, 191.05764
98	biatractylenolide	501.2451	$C_{30}H_{38}O_4$	4.9	5.64	11310	$[M+K]^+$	202.09670, 276.17262
99	6-O-β-D-Glucopyranosyllactinolide	424.1466	$C_{16}H_{26}O_9$	0.6	5.69	40813	$[M+NO_3]^-$	385.07830, 367.06790, 313.05783, 291.07329, 243.03503, 209.03240, 191.02148, 169.01404, 147.03194, 129.02086
100	α-cubebol	231.1705	$C_{14}H_{24}O$	-1.4	5.8	17533	$[M+Na]^+$	121.03789

101	tangshenoside V or its isomer	515.1409	$C_{21}H_{26}O_{12}$	0.3	5.9	14004	$[M+HCOO]^-$	173.03655, 265.07538, 341.09350
102	Vitamin B15 or its isomer	475.2455	$C_{20}H_{40}N_2O_8$	3.9	6.11	9562	$[M+K]^+$	115.05515, 177.05808, 238.08385, 279.09241, 337.19241
103	L-Tetrandrine	623.3138	$C_{38}H_{42}N_2O_6$	2.2	6.17	7264	$[M+H]^+$	548.28141
104	tangshenoside V or its isomer	515.1408	$C_{21}H_{26}O_{12}$	0.2	6.22	64757	$[M+HCOO]^-$	179.03707, 191.05763, 375.06953
105	Foliosidine	308.1475	$C_{16}H_{21}NO_5$	-1.8	6.3	11413	$[M+H]^+$	244.12925
106	Mudanpioside F	406.1363	$C_{16}H_{24}O_8$	0.8	6.31	51989	$[M+NO_3]^-$	136.05425
107	1,8-dioxo-1,8-dihydropyrano[3,4-c]pyran-3,6-dicarboxyl acid, diethyl ester or its isomer	355.0681	$C_{14}H_{14}O_8$	1	6.36	24295	$[M+HCOO]^-$	282.11506, 209.03368
108	Vitamin B15 or its isomer	475.2449	$C_{20}H_{40}N_2O_8$	3.3	6.44	27045	$[M+K]^+$	70.06622, 143.08230, 215.1142, 351.17390
109	chuanxiongoside A	389.1777	$C_{18}H_{28}O_9$	-2.9	6.48	8780	$[M+H]^+$	132.04499, 174.09277, 231.12577, 273.10116
110	6-methoxycoumarin or its isomer	177.0553	$C_{10}H_8O_3$	0.6	6.53	49090	$[M+H]^+$	145.02947
111	2-phenylethyl vicianoside	461.1663	$C_{19}H_{28}O_{10}$	-0.1	6.63	15860	$[M+HCOO]^-$	226.12271
112	8,8'-diferulic acid	404.1355	$C_20H_{18}O_8$	1.5	6.77	5237	$[M+NH_4]^+$	107.05043, 179.07589

113	5,6,4′-Trihydroxy-7,3′-dimethoxyflavone	348.1065	$C_{17}H_{14}O_7$	-1.3	6.82	8516	$[M+NH_4]^+$	139.0411
114	[(2R,3S,4S,5R,6R)-3,4,5-Trihydroxy-6-phenylmethoxyoxan-2-yl]methyl hydrogen sulfate	349.0605	$C_{13}H_{18}O_9S$	0.7	6.98	109476	$[M-H]^-$	96.96181, 137.02660, 185.06089, 241.00466
115	(+)-Catechin-3-O-β-D-glucopyranoside	451.1257	$C_{21}H_{24}O_{11}$	1.1	7.02	12326	$[M-H]^-$	124.01840, 244.03076 ,289.07310, 305.07170
116	6′-O-Galloyldesbenzoylpaeoniflorin or its isomer	527.1407	$C_{23}H_{28}O_{14}$	0	7.05	15410	$[M-H]^-$	169.01612, 313.05869, 399.09437, 479.11932, 497.13098
117	3-(2-hydroxyacetoxy)-5α,8α-peroxydehydrotumulosic acid	611.3008	$C_{33}H_{48}O_8$	2.7	7.17	20700	$[M+K]^+$	144.08174, 353.18311
118	Paeonisothujone	183.1023	$C_{10}H_{14}O_3$	0.7	7.3	53270	$[M+H]^+$	109.06610, 151.04061, 165.09268
119	(E)-3-(2-methoxyphenyl)-2-propen-1-ol	165.0918	$C_{10}H_{12}O_2$	0.8	7.3	24946	$[M+H]^+$	109.06610, 119.08628, 147.08263
120	4-O-Methyldebenzoylpaeoniflori	389.1456	$C_{17}H_{26}O_{10}$	0.2	7.31	84616	$[M-H]^-$	135.04693, 167.03709, 179.03669, 237.03554

	n							
121	codonopyrrolidiums A or its isomer	385.1673	$C_{19}H_{28}NO_5$	1.2	7.4	14366	$[M+Cl]^-$	134.03913
122	Paeoniostin A	485.1276	$C_{19}H_{26}O_{13}$	1	7.41	5751	$[M+Na]^+$	105.03430, 177.05547, 209.09314, 369.07780
123	Procyanidin B7	577.1351	$C_{30}H_{26}O_{12}$	0	7.56	21785	$[M-H]^-$	125.02644, 161.02611, 289.07326, 407.07837, 451.10644
124	(S)-naringenin	290.1031	$C_{15}H_{12}O_5$	0.8	7.71	13158	$[M+NH_4]^+$	103.05531, 131.05081
125	3-Hydroxy-4-methoxybenzoic acid	167.0365	$C_8H_8O_4$	1.5	7.79	111652	$[M-H]^-$	93.03616, 121.03151, 123.04698, 134.03926
126	1,8-dioxo-1,8-dihydropyrano[3,4-c]pyran-3,6-dicarboxyl acid, diethyl ester or its isomer	355.0677	$C_{14}H_{14}O_8$	0.7	7.9	27085	$[M+HCOO]^-$	135.04685, 179.03690, 311.04379
127	Caffeic acid	179.0366	$C_9H_8O_4$	1.6	7.92	15954	$[M-H]^-$	135.04685
128	Paeonidanins G	861.2797	$C_{39}H_{50}O_{20}$	1	8.02	7532	$[M+Na]^+$	121.02943, 145.02950, 357.09534, 519.14754
129	tangshenoside V or its isomer	515.1404	$C_{21}H_{26}O_{12}$	-0.2	8.03	96328	$[M+HCOO]^-$	161.02601, 281.06877, 323.07826, 375.06693
130	Oxypaeoniflora	558.14627	$C_{23}H_{28}O_{12}$	0	8.04	595200	$[M+NO_3]^-$	135.04667, 161.02601, 323.07826, 385.07804
131	Sachaloside	385.1671	$C_{19}H_{28}NO_5$	0.9	8.05	13733	$[M+Cl]^-$	93.03602, 135.04667, 147.03187,

								179.03656, 195.06750
132	codonopyrrolidiums A or its isomer	385.1671	$C_{19}H_{28}NO_5$	0.9	8.05	13733	$[M+Cl]^-$	191.05738
133	Catechin	289.0729	$C_{15}H_{14}O_6$	1.2	8.11	108596	$[M-H]^-$	109.03111, 125.02625, 151.04206, 271.05148
134	6′-O-Galloyldesbenzoylpaeoniflorin or its isomer	527.1406	$C_{23}H_{28}O_{14}$	0	8.13	11695	$[M-H]^-$	125.02625, 169.01598, 251.05813, 295.05079, 345.11987
135	atractyloside A or its isomer	493.2299	$C_{21}H_{36}O_{10}$	0.8	8.15	38956	$[M+HCOO]^-$	108.02350, 125.02625, 213.15183
136	1'-O-benzoylsucrose or its isomer	491.1419	$C_{19}H_{26}O_{12}$	1.3	8.24	30080	$[M+HCOO]^-$	179.05883, 341.10939, 379.10818
137	Paeoniflorol	467.189	$C_{23}H_{30}O_{10}$	-2.2	8.27	23098	$[M+H]^+$	117.03444, 234.09164, 347.13676
138	4-O-methylgalloyloxypaeoniflorin	707.1831	$C_{31}H_{34}O_{16}$	0.3	8.53	97041	$[M+HCOO]^-$	135.04690, 191.05757, 353.08857, 567.12782
139	Chlorogenic acid	353.0886	$C_{16}H_{18}O_9$	0.8	8.53	743619	$[M-H]^-$	135.04690, 191.05757
140	7-hydroxy coumarin	163.0397	$C_9H_6O_3$	0.7	8.53	225223	$[M+H]^+$	145.02962
141	ganoderic acid B	555.274	$C_{30}H_{44}O_7$	2.1	8.65	3464	$[M+K]^+$	112.08701, 178.09179, 232.13640
142	Z-6-hydroxy-7-	239.1285	$C_{13}H_{18}O_4$	0.7	8.69	24309	$[M+H]^+$	145.02913, 177.05647

	methoxy-dihydroligustilide							
143	Salicylpaeoniflorin	558.1463	$C_{23}H_{28}O_{12}$	0.0	8.85	183516	$[M+NO_3]^-$	137.02619, 229.01627, 339.05213, 465.14149
144	Oxypaeoniflorin	514.192	$C_{23}H_{28}O_{12}$	0.1	8.85	94721	$[M+NH_4]^+$	151.07663, 317.09711, 335.11160
145	tangshenoside V or its isomer	515.1412	$C_{21}H_{26}O_{12}$	0.5	8.91	19662	$[M+HCOO]^-$	179.03669, 341.09252
146	Isotrilobine-N-2-oxide	610.2883	$C_{36}H_{36}N_2O_6$	-2.9	8.95	9324	$[M+NH_4]^+$	308.13200, 455.15577, 463.16199
147	Cryptochlorogenic acid	353.0883	$C_{16}H_{18}O_9$	0.5	9.1	266875	$[M-H]^-$	71.01491, 93.03607, 135.04682, 173.04748, 191.05776, 307.08340
148	Paeonin A	417.1405	$C_{17}H_{24}O_9$	0.3	9.15	14649	$[M+HCOO]^-$	150.03392, 167.04134
149	Isomaltopaeoniflorin	660.251	$C_{29}H_{38}O_{16}$	1.2	9.31	13948	$[M+NH_4]^+$	151.08218, 295.10146, 419.13720, 503.15619
150	Paeonin D	771.1626	$C_{37}H_{36}O_{16}$	-7.1	9.46	48394	$[M+Cl]^-$	147.03192, 367.06866, 385.07842, 511.14614
151	6-methoxycoumarin or its isomer	177.0551	$C_{10}H_8O_3$	0.5	9.47	125185	$[M+H]^+$	145.02945
152	Scopolin	399.0938	$C_{16}H_{18}O_9$	0.5	9.74	36150	$[M+HCOO]^-$	161.04846, 259.06247
153	natsudaidain	419.1345	$C_{21}H_{22}O_9$	0.9	9.79	4068	$[M+H]^+$	137.06069, 266.04043
154	Phenylglucoside	255.089	$C_{12}H_{16}O_6$	1.6	9.87	13008	$[M-H]^-$	124.01832
155	Epistephanine	624.3109	$C_{37}H_{38}N_2O_6$	4.1	9.89	23192	$[M+NH_4]^+$	120.08164, 283.09846, 473.24074
156	11α,12α-Epoxy-3β,23-dihydroxy-30-norolean-20	509.2683	$C_{29}H_{42}O_5$	1.9	9.89	38125	$[M+K]^+$	389.18418, 475.18495

	(29)-en-28,13β-olide							
157	Proanthocyanidin B2	577.1355	$C_{30}H_{26}O_{12}$	0.3	9.97	22862	$[M-H]^-$	289.07311, 407.07852, 425.08968, 451.10400
158	4-O-Methyl-4″-hydroxy-3″-methoxy-paeoniflorin or its isomer	579.1494	$C_{25}H_{32}O_{13}$	2	9.98	15619	$[M+K]^+$	127.04028, 151.07740, 179.07290, 379.10435, 425.14247, 441.11530
159	4″-Hydroxy-3″-methoxyalbiflorin	588.1569	$C_{24}H_{30}O_{10}$	-0.1	9.99	128406	$[M+NO_3]^-$	125.02642, 167.03701, 175.04300, 289.07311, 385.07856, 425.08968, 495.15299
160	Albiflorin R1	525.1615	$C_{23}H_{28}O_{11}$	0.1	9.99	90836	$[M+HCOO]^-$	91.02055, 108.02335, 123.04709, 125.02642, 167.03701, 175.04300, 289.07311, 305.06953, 367.06759, 385.07856
161	Daturilin	459.2518	$C_{28}H_{36}O_4$	1.2	10.01	5169	$[M+Na]^+$	79.05463, 123.04613, 133.06519, 179.07290
162	undulatoside A	399.0938	$C_{16}H_{18}O_9$	0.5	10.05	45735	$[M+HCOO]^-$	129.02093, 326.09736
163	(6S,9S)-roseoside	448.1832	$C_{19}H_{30}O_8$	0.7	10.21	11285	$[M+NO_3]^-$	135.04751, 265.07602
164	1'-O-benzoylsucrose or its isomer	508.132	$C_{19}H_{26}O_{12}$	1.2	10.38	96404	$[M+NO_3]^-$	121.03147, 193.05263, 429.14429
165	dihydrocinnacasside	327.1097	$C_{15}H_{20}O_8$	1.1	10.53	17008	$[M-H]^-$	119.05193, 191.05832, 205.05236, 233.04329, 355.14953
166	Paeonolide	522.1473	$C_{20}H_{28}O_{12}$	0.9	10.58	28184	$[M+NO_3]^-$	122.03878, 150.03417, 165.05792,

								167.04092, 191.05832, 205.04447, 245.08330, 272.09217, 289.07315, 337.09410
167	epicatechin	289.0731	$C_{15}H_{14}O_6$	1.3	10.58	34946	$[M-H]^-$	109.02908, 165.05792, 181.05783
168	luteolin-7-O-rutinoside	609.1453	$C_{27}H_{30}O_{16}$	-0.8	10.64	16436	$[M-H]^-$	191.05770, 339.05213, 558.14669
169	Poacynose	415.1248	$C_{18}H_{24}O_{11}$	0.2	10.66	56483	$[M-H]^-$	125.02655, 169.01593, 215.07337, 239.06333
170	2,5-dimethyl-phenyl butyric acid methyl ester	207.1376	$C_{13}H_{18}O_2$	-0.4	10.66	42481	$[M+H]^+$	103.05480, 115.05453
171	tangshenoside III deglycosylation	425.1573	$C_{22}H_{26}O_7$	0.2	10.66	15005	$[M+Na]^+$	189.08318, 341.06604
172	Galloyloxypaeoniflorin	323.0782	$C_{30}H_{32}O_{16}$	0.4	10.74	14890	$[M-2H]^-$	261.07792, 323.07809, 367.06799, 390.13123, 481.10054, 515.11646
173	penicitrinol M	366.1906	$C_{19}H_{24}O_6$	-0.5	10.76	9070	$[M+NH_4]^+$	145.02716, 177.05933, 223.06491
174	cinnacaside	562.2906	$C_{26}H_{40}O_{12}$	4.8	10.79	8468	$[M+NH_4]^+$	177.05933, 312.12679, 385.14857, 497.20351, 505.23344
175	folinic acid	508.1321	$C_{20}H_{23}N_7O_7$	-3.2	10.84	17295	$[M+Cl]^-$	121.03146, 133.01601, 194.05889, 209.08418
176	atractyloside F	827.3918	$C_{37}H_{62}O_{20}$	1.1	10.86	6196	$[M+H]^+$	144.08253, 244.13414, 354.17663, 749.36088
177	Paeonovicinoside	469.1308	$C_{19}H_{26}O_{12}$	-0.9	10.93	13134	$[M+Na]^+$	208.10808, 224.10489, 284.13296
178	β-Gentiobiosylp	687.2138	$C_{29}H_{38}O_{16}$	-0.4	10.95	11233	$[M+HCOO]^-$	263.06177, 383.06177, 558.14756,

	aeoniflorin							628.20900
179	Paeoniflorin	525.1615	$C_{23}H_{28}O_{11}$	0.2	11.05	2515943	$[M+HCOO]^-$	121.03113, 283.08392, 327.1114, 357.13067, 479.15647
180	Tryptophol	206.0833	$C_{10}H_{11}NO$	1.1	11.18	15880	$[M+HCOO]^-$	142.0665
181	6,8-di-c-glucosylapigenin or its isomer	593.1492	$C_{27}H_{30}O_{15}$	-2	11.37	121105	$[M-H]^-$	353.06688, 383.07754, 503.11940, 575.13627
182	phospholipid	695.3652	$C_{31}H_{55}N_5O_9P$	2.3	11.42	7750	$[M+Na]^+$	267.06786, 353.15471, 397.18195, 519.22531, 544.24777
183	Astragaloside IV	823.4246	$C_{41}H_{68}O_{14}$	0.5	11.44	14499	$[M+K]^+$	348.16161, 397.18195, 518.25900
184	UNII-A9ZZD585U6	879.4424	$C_{42}H_{68}N_2O_{16}$	-3.7	11.47	8014	$[M+Na]^+$	252.13742, 382.19097, 606.27553, 742.35324
185	Paeoveitols B	183.1039	$C_{10}H_{16}O_3$	1.2	11.54	42429	$[M-H]^-$	78.01235, 93.03611, 134.03884, 167.07334
186	Suffruticosol A	787.2345	$C_{47}H_{40}O_9$	4.1	11.56	7298	$[M+K]^+$	415.19264, 549.19530
187	Albiflorin	525.1609	$C_{23}H_{28}O_{11}$	-0.5	11.65	3323681	$[M+HCOO]^-$	77.04068, 121.03090, 165.05707, 195.06762, 289.07266, 327.10864, 345.11951, 375.13035, 449.14490
188	Apigenin 7-neohesperidoside	623.1604	$C_{27}H_{30}O_{14}$	-1.4	11.81	23661	$[M+HCOO]^-$	146.96812, 312.06545, 383.07874, 413.08968, 484.02290, 503.12174, 524.19919
189	narirutin-4'-O-glucoside	787.2283	$C_{33}H_{42}O_{19}$	-2	11.85	127545	$[M+HCOO]^-$	151.00510, 263.06066, 271.06182, 433.11384, 495.15034, 579.17098, 647.16045
190	Tangshenoside	765.2314	$C_{34}H_{46}O_{17}$	-5.2	11.86	8696	$[M+K]^+$	273.07501, 365.11023, 497.21010,

	III						581.18575, 655.25266
191	3-hydroxy-4,5,6,7-tetrahydro-6,7-dihydroxy-3-butylphthalide	241.1091	$C_{12}H_{18}O_5$	1	11.89	8272	$[M-H]^-$	151.04195, 181.05520, 196.07477, 205.08757
192	Paeonins C	495.1503	$C_{23}H_{28}O_{12}$	-0.5	12.07	39444	$[M-H]^-$	93.03594, 137.02607, 219.03014, 353.06726, 383.07713, 413.08831, 425.08385
193	icariside DC	478.1566	$C_{19}H_{28}O_{10}$	0	12.13	15889	$[M+NO_3]^-$	124.01842, 167.03816, 227.07258
194	icariside B5	450.198	$C_{19}H_{32}O_8$	-0.1	12.26	9700	$[M+NO_3]^-$	165.05777, 222.12595
195	FERULIC ACID (CIS)	193.0517	$C_{10}H_{10}O_4$	1.1	12.37	78456	$[M-H]^-$	134.03889, 161.02535
196	Hexyl-beta-D-glucopyranosyl-(1-2)-beta-D-glucopyranoside or its isomer	471.2083	$C_{18}H_{34}O_{11}$	-0.1	12.42	35946	$[M+HCOO]^-$	99.04661, 191.05812, 261.09900, 389.18228
197	9-O-butylpaeonidanin	581.223	$C_{27}H_{36}O_{11}$	-0.9	12.51	4130	$[M+HCOO]^-$	121.03120, 245.09357, 367.06753, 433.15352, 477.14510
198	Hexyl-beta-D-glucopyranosyl-(1-2)-beta-D-glucopyranoside or its isomer	488.1986	$C_{18}H_{34}O_{11}$	0.1	12.76	25124	$[M+NO_3]^-$	109.02949, 165.05746, 239.07346, 364.1680
199	eriocitrin	595.1643	$C_{27}H_{32}O_{15}$	-2.5	12.91	26756	$[M-H]^-$	107.01531, 121.03100, 151.00521, 271.06160, 287.05707, 315.05227,

								433.11407, 542.15102, 563.13969
200	(2R)-(-)-naringenin-5-O-β-D-glucopyranoside	433.1138	$C_{21}H_{22}O_{10}$	-0.2	12.92	11166	$[M-H]^-$	177.02105, 271.06160, 287.05707, 315.05227
201	Albiflorin R2	503.1535	$C_{23}H_{28}O_{11}$	1.1	12.94	30086	$[M+Na]^+$	77.04014, 283.06232
202	4'-O-methylellagic acid 4-O-β-D-glucopyranos	477.0673	$C_{21}H_{18}O_{13}$	-0.1	12.98	8553	$[M-H]^-$	125.02621, 315.01299, 413.08784
203	chuanxiongoside B or its isomer	566.2074	$C_{23}H_{36}O_{12}$	-1.6	13.06	34583	$[M+NO_3]^-$	99.00917, 145.03094, 191.05576, 240.13643, 403.19883
204	Kaempferol-3-O-β-D-glucopyranosyl-7-O-β-D-glucopyranoside	609.144	$C_{27}H_{30}O_{16}$	-2.1	13.1	33482	$[M-H]^-$	133.03073, 151.00517, 243.03085, 271.02570, 300.02797, 367.07587, 469.13428
205	icariside B8	450.1981	$C_{19}H_{32}O_8$	0	13.18	18118	$[M+NO_3]^-$	124.01800, 169.01518
206	chuanxiongnolide R2	249.1109	$C_{12}H_{18}O_4$	1.1	13.25	18092	$[M+Na]^+$	133.06529
207	Alpha-Asarone	209.1161	$C_{12}H_{16}O_3$	-1.1	13.41	28494	$[M+H]^+$	145.03091
208	Rubrosterone or its isomer	352.2112	$C_{19}H_{26}O_5$	-0.6	13.47	39613	$[M+NH_4]^+$	91.05533, 133..6587, 161.06026
209	Paeonilactone C	319.1201	$C_{17}H_{18}O_6$	2.5	13.49	16628	$[M+H]^+$	105.03405, 161.06026
210	Galloylalbiflorin	631.1648	$C_{30}H_{32}O_{15}$	-2	13.52	519078	$[M-H]^-$	121.03094, 169.01547, 313.05674, 399.09282, 491.11899, 613.15449
211	chuanxiongnolideA	458.1801	$C_{24}H_{28}O_5$	-1.9	13.62	11701	$[M+NO_3]^-$	133.03102, 161.02587, 263.03573

212	forsythiaside or its isomer	623.1964	$C_{29}H_{36}O_{15}$	-1.8	13.65	34860	$[M-H]^-$	461.16767
213	Senkyunolide J	249.1103	$C_{12}H_{18}O_4$	0.6	13.71	21451	$[M+Na]^+$	77.03960, 135.04553, 149.02351
214	Foeniculin	469.0508	$C_{20}H_{18}O_{11}$	-3.5	13.81	46636	$[M+Cl]^-$	95.01495, 125.02587, 169.01565, 277.03711, 330.03900
215	1,2,3,4,6-Penta-O-galloyl-β-D-glucose	958.1543	$C_{41}H_{32}O_{26}$	2.3	13.84	14670	$[M+NH_4]^+$	125.02412, 153.01893, 305.02931, 601.08293, 771.10248
216	Paeoniflorone	335.1137	$C_{17}H_{20}O_7$	0.1	13.92	38717	$[M-H]^-$	124.01806, 301.07260
217	D-galactitol 3′R,4′S-acetal	319.1164	$C_{15}H_{20}O_6$	1.2	13.93	11469	$[M+Na]^+$	120.08149
218	Dipotassium glycyrrhizinate	937.2697	$C_{42}H_{60}K_2O_{16}$	-9	13.96	5418	$[M+K]^+$	325.15282, 457.21668, 581.22260
219	Coumarin	147.0441	$C_9H_6O_2$	0	13.97	13851	$[M+H]^+$	147.04406
220	narirutin	579.1704	$C_{27}H_{32}O_{14}$	-1.6	14.05	765224	$[M-H]^-$	107.01526, 119.05160, 125.02587, 145.03104, 151.00507, 165.02071, 177.02070, 191.05763, 211.02639, 219.03125, 229.05141, 271.0617, 313.06042, 399.09348, 459.11345, 533.16594
221	Engeletin	435.1306	$C_{21}H_{22}O_{10}$	2	14.07	21940	$[M+H]^+$	153.01812, 177.05713, 273.07581, 357.05825, 419.13987
222	Jionoside B2	832.321	$C_{37}H_{50}O_{20}$	-2.4	14.09	13901	$[M+NH_4]^+$	603.16995
223	Sengosterone	598.2864	$C_{29}H_{44}O_9$	-0.5	14.11	14338	$[M+NO_3]^-$	373.19775, 391.20968, 425.20299, 467.21537
224	Rubrosterone or	352.2106	$C_{19}H_{26}O_5$	-1.2	14.13	52853	$[M+NH_4]^+$	121.06576, 159.08127, 303.11925

	its isomer							
225	4'-O-galloylalbiflorin	631.166	$C_{30}H_{32}O_{15}$	-0.8	14.28	53453	$[M-H]^-$	107.01565, 121.03131, 125.02605, 169.01591, 211.02770, 313.05802, 399.09315, 477.10523, 509.12965, 542.15250
226	Rubrosterone or its isomer	352.2098	$C_{19}H_{26}O_5$	-2.1	14.32	71464	$[M+NH_4]^+$	91.05471, 136.07896
227	forsythiaside or its isomer	623.1974	$C_{29}H_{36}O_{15}$	-0.7	14.33	19132	$[M-H]^-$	461.16714
228	Azelaic acid	187.0991	$C_9H_{16}O_4$	1.5	14.45	26781	$[M-H]^-$	125.09884, 151.08290
229	4-O-galloylalbiflorin	631.1663	$C_{30}H_{32}O_{15}$	-0.5	14.67	95623	$[M-H]^-$	121.03132, 125.02614, 329.03122, 330.03866, 345.06155, 360.04883, 374.06526, 492.09331, 507.11484, 537.12549
230	hesperidin	609.1826	$C_{28}H_{34}O_{15}$	0.1	15.03	1409817	$[M-H]^-$	83.01522, 93.03629, 107.01560, 136.01850, 164.01331, 286.04941, 301.07302, 441.14132, 593.14919
231	ligustilide I	247.0944	$C_{12}H_{16}O_4$	0.3	15.12	19428	$[M+Na]^+$	149.02168, 189.09191
232	Rehmapicrogenin	207.0997	$C_{10}H_{16}O_3$	0.5	15.12	97272	$[M+Na]^+$	98.97759
233	9-epi-Oxypaeonidanin	509.1677	$C_{24}H_{30}O_{12}$	1.2	15.16	33645	$[M-H]^-$	77.04108, 121.03136, 135.04692, 353.08944, 479.15737
234	3,5,6,7,8,3',4'-heptamethoxyflavone	479.157	$C_{22}H_{26}O_9$	1.1	15.19	286106	$[M+HCOO]^-$	121.03136, 195.06881, 271.06506, 331.12073
235	4-epi-albiflorin	481.1709	$C_{23}H_{28}O_{11}$	0.5	15.22	46530	$[M+H]^+$	127.04148, 377.08927

236	Cyasterone	565.3023	$C_{29}H_{44}O_8$	0.5	15.41	372604	$[M+HCOO]^-$	167.03744, 249.15344, 273.15135, 301.18276, 319.19291, 373.20169, 391.22229, 439.28734, 501.28671
237	Ecdysterone	503.2988	$C_{27}H_{44}O_7$	0.8	15.44	142377	$[M+Na]^+$	91.05450, 129.06814, 211.10812, 283.17069, 301.17999, 373.20416, 449.26295
238	Precyasterone	521.3107	$C_{29}H_{44}O_8$	-0.2	15.45	112644	$[M+H]^+$	91.05450, 129.06814, 171.07680, 211.10812, 283.17069, 301.17999, 373.20416, 409.21303, 485.28963
239	24-hydroxycyasterone	598.2875	$C_{29}H_{44}O_9$	0.6	15.5	64709	$[M+NO_3]^-$	229.12571, 335.18820
240	Lactiflorin	507.151	$C_{23}H_{26}O_{10}$	0.2	15.76	40293	$[M+HCOO]^-$	125.02632, 169.01584, 202.02872
241	paeonibenzofuran	237.0779	$C_{24}H_{28}O_{10}$	0.5	15.94	135954	$[M-2H]^-$	91.02035, 108.02344, 237.07832
242	chuanxiongoside B or its isomer	566.2084	$C_{23}H_{36}O_{12}$	-0.7	16.03	76752	$[M+NO_3]^-$	101.02565, 125.02594, 169.01559, 311.09709, 437.20640
243	galloylpaeoniflorin	631.1663	$C_{30}H_{32}O_{15}$	-0.6	16.2	25847	$[M-H]^-$	301.07425, 313.05756, 314.05040, 399.09373, 407.13431, 465.14105, 507.11582
244	nomilin	515.2246	$C_{28}H_{34}O_9$	-3	16.33	5404	$[M+H]^+$	161.06060, 375.09780
245	trans-ε-Viniferin	500.2037	$C_{30}H_{26}O_6$	-3	16.49	2971	$[M+NH_4]^+$	163.07625, 187.07559, 360.13736
246	penicitrinol I	263.1302	$C_{15}H_{20}O_4$	1.3	16.57	10667	$[M-H]^-$	173.06220, 189.05964, 220.10942
247	benzenamine,3-chloro-N-(2-pyridinylmethylene)-	217.0517	$C_{12}H_{11}C_lN_2$	-2.1	16.59	10351	$[M-H]^-$	193.05244

248	atractyloside A or its isomer	510.2189	$C_{21}H_{36}O_{10}$	-0.3	16.6	20812	$[M+NO_3]^-$	119.05192, 220.10942, 317.12741, 367.17894
249	(Z)-(1S,5R)-β-Pinen-10-yl β-vicianoside	491.2138	$C_{21}H_{34}O_{10}$	0.4	16.67	28178	$[M+HCOO]^-$	137.02653, 257.12978
250	Eucalyptin	344.1469	$C_{19}H_{18}O_5$	-2.4	16.77	68592	$[M+NH_4]^+$	77.03966, 201.04737
251	6′-Hemiglutaryl-paeoniflorin	656.1822	$C_{28}H_{34}O_{14}$	-1.1	16.83	111972	$[M+NO_3]^-$	164.01315, 285.07782, 479.12014, 508.20429(
252	Liquiritin apioside	551.1745	$C_{26}H_{30}O_{13}$	-1.4	16.91	6547	$[M+H]^+$	221.08083, 405.11766
253	11-angeloylsenkyunolide F	333.1348	$C_{17}H_{20}O_4$	0.4	17.01	8080	$[M+HCOO]^-$	125.02622, 174.10478, 203.11039
254	FA	463.1179	$C_{19}H_{18}N_7O_6$	-3.1	17.03	5802	$[M+Na]^+$	404.11142
255	tangshenoside I	717.1984	$C_{29}H_{42}O_{18}$	-1.9	17.06	13835	$[M+K]^+$	127.04149, 287.07866, 419.13100
256	tamoxifen	389.2549	$C_{26}H_{29}NO$	-3.8	17.12	8341	$[M+NH_4]^+$	95.05130, 131.05080
257	Spinoside A	734.4125	$C_{39}H_{56}O_{12}$	1.5	17.29	9430	$[M+NH_4]^+$	242.11636, 282.14821, 377.18629, 474.23795, 542.31715, 587.31745, 663.35981
258	(Z)-3-butylidene phthalide	189.0917	$C_{12}H_{12}O_2$	0.7	17.34	24616	$[M+H]^+$	147.0421
259	2'-O-benzoylpaeoniflorin	629.1869	$C_{30}H_{32}O_{12}$	-0.7	17.41	427849	$[M+HCOO]^-$	121.03113, 193.05244, 431.13579, 553.17084
260	Procyanidin B3	577.1347	$C_{30}H_{26}O_{12}$	-0.5	17.43	22341	$[M-H]^-$	134.03914, 165.05750, 271.06263, 392.05173, 407.07816
261	sedanoic-acid	211.1329	$C_{12}H_{18}O_3$	0	17.46	105772	$[M+H]^+$	147.04532
262	Benzoylwurdin	629.1869	$C_{30}H_{32}O_{12}$	-0.6	17.52	112431	$[M-H]^-$	125.02608, 417.11952

263	tumulosic acid	242.1771	$C_{31}H_{50}O_4$	-3.8	17.87	98655	$[M-2H]^-$	123.04692, 165.09281, 181.16048, 225.15115, 242.17746, 455.35445
264	5,6,9-trihydroxy-octadec-7-enoic acid	329.2335	$C_{18}H_{34}O_5$	0.2	18.05	281166	$[M-H]^-$	139.11417, 171.10463
265	Phellatin	535.1768	$C_{26}H_{30}O_{12}$	-4.2	18.08	3509	$[M+H]^+$	1953.02946, 303.08461, 341.02831, 449.14424
266	coriacoic acid C	549.3501	$C_{33}H_{50}O_5$	-5	18.11	6590	$[M+Na]^+$	141.07054, 257.11384, 271.13458
267	calycosin-7-O-beta-D-glucoside	491.12	$C_{22}H_{22}O_{10}$	0.5	18.14	14885	$[M+HCOO]^-$	255.02009, 307.07767
268	Malvoside	673.222	$C_{29}H_{35}O_{17}$	0.8	18.14	12304	$[M+NH_4]^+$	121.03056, 165.06973, 453.13408
269	Neohancoside C	469.1152	$C_{19}H_{26}O_{11}$	4.6	18.16	20306	$[M+K]^+$	149.02473, 193.05253, 237.07518
270	(Z)-3-butylidene phthalide	189.0924	$C_{12}H_{12}O_2$	1.4	18.22	11404	$[M+H]^+$	189.09237
271	Isosinensetin or its isomer	373.1286	$C_{20}H_{20}O_7$	0.5	18.28	251828	$[M+H]^+$	105.03493, 193.05230, 327.05149
272	Senkyunolide G	207.104	$C_{12}H_{16}O_3$	1.3	18.31	10807	$[M-H]^-$	159.04512
273	5-hydroxy-3,3',4',7,8-pentamethoxyflavone	389.1251	$C_{20}H_{20}O_8$	2	18.38	19279	$[M+H]^+$	375.10498
274	Betavulgarin	313.0722	$C_{17}H_{12}O_6$	1.5	18.44	27591	$[M+H]^+$	295.06505
275	n-Valerophenone-O-carboxylic acid	205.0883	$C_{12}H_{14}O_3$	1.3	18.53	54488	$[M-H]^-$	131.05139
276	cinnamomumolide	329.1049	$C_{18}H_{16}O_6$	3	18.59	12367	$[M+H]^+$	165.05736, 267.07108

277	5-methoxy-7-[(3-methyl-2-buten-1-yl)oxy]-2H-1-benzopyran-2-one	261.1143	$C_{15}H_{16}O_4$	2.1	18.65	11026	$[M+H]^+$	229.04702
278	2-Methoxy-4-(3-methoxy-1-propenyl)-phenol	193.0886	$C_{11}H_{14}O_3$	1.6	18.66	18149	$[M-H]^-$	147.04710, 177.05748
279	Atractylenolide II	250.1788	$C_{15}H_{20}O_2$	-1.3	18.73	172010	$[M+NH_4]^+$	94.04253, 117.03488, 122.03729, 131.05018, 160.05170, 175.07615
280	dihydroxy-β-ionone	228.1965	$C_{13}H_{22}O_2$	0.7	18.73	533025	$[M+NH_4]^+$	114.95439, 133.02025, 191.00224, 210.11993
281	tetramethyl-O-scutellarin	373.1292	$C_{20}H_{20}O_7$	1.1	18.8	216222	$[M+H]^+$	313.07405
282	limonin	515.1929	$C_{26}H_{30}O_8$	0.6	18.81	28764	$[M+HCOO]^-$	92.99739, 187.00049, 357.13790
283	ledebouriellol	375.1463	$C_{20}H_{22}O_7$	2.5	18.93	15888	$[M+H]^+$	272.06634
284	(Z)-3-butenyl-5-hydroxyphthalide	203.0727	$C_{12}H_{12}O_3$	1.3	18.96	16952	$[M-H]^-$	117.03675, 145.03137, 173.02709
285	1-tert-butyl-4-(2-methylprop-2-enyl)benzene	206.1917	$C_{14}H_{20}$	1.4	19.09	79424	$[M+NH_4]^+$	117.06003, 146.09931, 160.11476
286	Atractylenolide I	231.1401	$C_{15}H_{18}O_2$	2.1	19.61	22811	$[M+H]^+$	121.03031, 162.06953, 173.06206
287	3(S)-3-Butyl-4,5-dihydrophthalid	193.1238	$C_{12}H_{16}O_2$	1.5	19.62	291465	$[M+H]^+$	77.04062, 99.04561, 121.03031 ,137.06173, 162.03953

	e							
288	Heptamethoxyfl avone	433.1517	$C_{22}H_{24}O_9$	2.4	19.64	1202421	$[M+H]^+$	137.06173, 373.05891, 387.07470
289	Isosinensetin or its isomer	373.1304	$C_{20}H_{20}O_7$	2.2	19.8	300223	$[M+H]^+$	328.0592
290	2β-hydroxytrich oacorenol	256.2294	$C_{15}H_{26}O_2$	2.3	20.07	50183	$[M+NH_4]^+$	91.05514, 105.07063, 145.10189, 171.11767
291	pq-2	307.1907	$C_{18}H_{26}O_4$	0.3	20.57	83448	$[M+H]^+$	115.05591, 188.09239, 259.16732
292	cinnacasol	383.2052	$C_{20}H_{30}O_7$	-1.2	22.21	14198	$[M+H]^+$	235.06601, 285.16843, 301.14150
293	Senkyunolide Q	301.1412	$C_{16}H_{22}O_4$	0.2	22.24	372502	$[M+Na]^+$	149.02466, 160.99250, 184.07434
294	riligustilide_1	381.2064	$C_{24}H_{28}O_4$	0.4	22.57	10994	$[M+H]^+$	79.05588, 135.04647, 149.06309, 213.09336
295	Di-(2-ethylhexyl)phthalate	413.2678	$C_{24}H_{38}O_4$	1.6	25.27	17824	$[M+Na]^+$	121.02968, 128.06322, 149.02326
296	3β-acetoxylanos ta-7,9(11),24-tri en-21-oic acid	497.362	$C_{32}H_{48}O_4$	-0.6	25.58	13473	$[M+H]^+$	378.32048
297	ceanphytamic acid B	569.3815	$C_{33}H_{54}O_6$	0.2	25.62	123574	$[M+Na]^+$	265.25615, 378.32048, 497.36175
298	stigmasterol-β-g lucoside	592.4591	$C_{35}H_{58}O_6$	2	25.62	10287	$[M+NH_4]^+$	305.13940, 457.25751
299	Z-6,7-epoxyligu stilide-SG3	536.1651	$C_{22}H_{31}N_3O_9S$	-2.2	26.35	780777	$[M+Na]^+$	131.03565, 147.06653, 149.04641, 207.03289, 265.02046, 281.05169, 299.06211

Table S2 ELISA Kit and Antibody List

Product name	Article number	Corporate name
Rat Triglyceride ELISA Kit	YJ639131	Shanghai Enzyme-linked Biotechnology Co., Ltd
Rat Total Cholesterol ELISA Kit	YJ634090	Shanghai Enzyme-linked Biotechnology Co., Ltd
Rat Low Density Lipoprotein ELISA Kit	YJ003390	Shanghai Enzyme-linked Biotechnology Co., Ltd
Rat high-density lipoprotein ELISA kit	YJ003387	Shanghai Enzyme-linked Biotechnology Co., Ltd
Rat Very Low Density Lipoprotein ELISA Kit	YJ290034	Shanghai Enzyme-linked Biotechnology Co., Ltd
Rat IL-1β ELISA kit	YJ003057	Shanghai Enzyme-linked Biotechnology Co., Ltd
Rat IL-6 ELISA kit	YJ102828	Shanghai Enzyme-linked Biotechnology Co., Ltd
Rat TNF-α ELISA kit	YJ002859	Shanghai Enzyme-linked Biotechnology Co., Ltd
Rat nuclear factor κB receptor activating factor ligand EL	YJ003065	Shanghai Enzyme-linked Biotechnology Co., Ltd
Rat osteoprotegerin ELISA kit	YJ280841	Shanghai Enzyme-linked Biotechnology Co., Ltd
Rat 6-Ketoprostaglandin F1a ELISA Kit	YJ003311	Shanghai Enzyme-linked Biotechnology Co., Ltd
Rat Thromboxane B2 ELISA Kit	YJ003311	Shanghai Enzyme-linked Biotechnology Co., Ltd
Human Nicotinamide mononucleotide adenylytransferase	YJ402208	Shanghai Enzyme-linked Biotechnology Co., Ltd
Rat adenosine triphosphate (ATP) ELISA Kit	YJ327235	Shanghai Enzyme-linked Biotechnology Co., Ltd
Rat adenosine triphosphate (ADP) ELISA Kit	YJ327280	Shanghai Enzyme-linked Biotechnology Co., Ltd
NAD+/NADH Assay Kit with WST-8	S0175	Beyotime
DAB kit	AR1027	Boster Biotechnology Co., Ltd.
rabbit two-step detection kit	SV0002	Boster Biotechnology Co., Ltd.
mouse two-step detection kit	SV0001	Boster Biotechnology Co., Ltd.
β-Actin (13E5) Rabbit mAb	4970S	Cell Signaling TECHNOLOGY™
STK11/LKB1 Monoclonal antibody	68016-1-Ig	proteintech
ACAT1 Polyclonal antibody	16215-1-AP	proteintech
NAMPT/PBEF Polyclonal antibody	11776-1-AP	proteintech
NMNAT1 Polyclonal antibody	28493-1-AP	proteintech
Rabbit Anti-phospho-HMGCR (Ser872) antibody	bs-4063R	Bioss ANTIBODIES

Table S3 Clinical information of study subjects

Baseline variables	NONFH group
Gender	
Male	2
Female	2
Age (year)	
Median (range)	34.5 (20-67)
Mean	39.00±19.92
Body mass index, (BMIkg/m^2)	
Median (range)	21.59 (20.62-24.22)
Mean	22.00±1.68
Total cholesterol (μmol/L)	
Median (range)	3.84 (2.89-4.49)
Mean	3.76±0.76
Triglyceride (μmol/L)	
Median (range)	0.9 (0.62-3.25)
Mean	1.42±1.23
Clinical information of study subjects	
Low density lipoprotein (μmol/L)	
Median (range)	2.11 (1.42-2.80)
Mean	2.11±0.62
High density lipoprotein (μmol/L)	
Median (range)	1.09 (1.01-1.16)
Mean	1.09±0.06
Steroid treatment	
Drug species	triamcinolone acetonide; prednisone; methylprednisolone; metacortandracin
Duration of steroid treatment (month)	
Median (range)	23 (8-120)
Mean	43.5±51.49
ARCO stage	
ARCOII	4
Visual analog scale	
Median (range)	4 (3-5)
Mean	4±0.82
Harris hip score (Left)	
Median (range)	83 (79-98)
Mean	85.75±8.38
Harris hip score (Right)	
Median (range)	75 (64-96)
Mean	77.50±13.75

Table S4 Details of early NONFH-related genes

NO.	GeneSymbol	Gene_Title	pvalues	foldchange	Regulatio
1	1-Mar	membrane-associated ring finge	0.028126159	1.67605362	up
2	8-Mar	membrane-associated ring finge	0.020132283	0.35595377	down
3	9-Sep	septin 9	0.001319144	1.72948244	up
4	AAK1	AP2 associated kinase 1	0.004567623	1.54418601	up
5	ABAT	4-aminobutyrate aminotransfera	0.00505535	1.56829495	up
6	ABCA5	ATP-binding cassette, sub-fami	0.001932156	1.60962938	up
7	ABCB10	ATP-binding cassette, sub-fami	0.000596212	0.52080747	down
8	ABCB6	ATP-binding cassette, sub-fami	0.032562169	0.47717827	down
9	ABCC13	ATP-binding cassette, sub-fami	0.008246475	0.22371079	down
10	ABCC3	ATP-binding cassette, sub-fami	0.011697057	1.57508833	up
11	ABCC4	ATP-binding cassette, sub-fami	0.027857313	0.49457633	down
12	ABCC5	ATP-binding cassette, sub-fami	0.010767863	1.57236167	up
13	ABCG1	ATP-binding cassette, sub-fami	0.014232622	1.79078097	up
14	ABCG2	ATP-binding cassette, sub-fami	0.002789713	0.19492955	down
15	ABHD2	abhydrolase domain containing	0.000479149	2.04859705	up
16	ABHD3	abhydrolase domain containing	0.018618695	1.64827599	up
17	ABHD5	abhydrolase domain containing	0.010673195	1.79366273	up
18	ABLIM1	actin binding LIM protein 1	0.000396588	1.99740895	up
19	ABLIM3	actin binding LIM protein famil	0.033482913	1.65102651	up
20	ABR	active BCR-related	0.003955927	1.71758875	up
21	ACAD9	acyl-CoA dehydrogenase family	0.014827658	1.51478404	up
22	ACAP1	ArfGAP with coiled-coil, ankyr	0.00553119	1.6557528	up
23	ACOX1	acyl-CoA oxidase 1, palmitoyl	0.001533465	1.75389175	up
24	ACP1	acid phosphatase 1, soluble	0.001252126	0.36623188	down
25	ACP5	acid phosphatase 5, tartrate resi	0.037830106	0.3827715	down
26	ACPP	acid phosphatase, prostate	0.007138149	1.72179567	up
27	ACSL6	acyl-CoA synthetase long-chain	0.004731472	0.18140884	down
28	ACSM3	acyl-CoA synthetase medium-c	0.005731529	0.18713662	down
29	ACTG1P4	actin, gamma 1 pseudogene 4 //	0.035409259	1.59566798	up
30	ACTN1	actinin, alpha 1	0.001291851	1.98474182	up
31	ACTR2	ARP2 actin-related protein 2 hc	0.028483629	1.50462562	up
32	ACVR1	activin A receptor, type I	0.027470919	1.50362691	up
33	ACVR1C	activin A receptor, type IC	0.000741876	1.65236059	up
34	ADAM19	ADAM metallopeptidase doma	0.004906401	1.60567867	up
35	ADAM28	ADAM metallopeptidase doma	0.013274078	1.58249469	up
36	ADAM8	ADAM metallopeptidase doma	0.004155665	1.95727846	up
37	ADCY7	adenylate cyclase 7	0.010764488	1.63460579	up
38	ADD2	adducin 2 (beta)	0.01625738	0.34738718	down
39	ADRBK2	adrenergic, beta, receptor kinas	0.045491538	1.70676843	up
40	ADTRP	androgen-dependent TFPI-regul	0.012735382	1.612622	up
41	AGBL5	ATP/GTP binding protein-like	0.02858677	0.57034886	down
42	AGER	advanced glycosylation end pro	0.005310926	1.50597186	up
43	AGO2	argonaute RISC catalytic comp	0.002155108	0.47208776	down
44	AGO4	argonaute RISC catalytic comp	0.002236198	1.92112837	up
45	AHCTF1	AT hook containing transcriptic	0.003899594	1.69968373	up
46	AHNAK	AHNAK nucleoprotein	0.014050374	1.51011277	up
47	AHSP	alpha hemoglobin stabilizing pr	0.003865203	0.2736777	down
48	AIDA	axin interactor, dorsalization as	0.000917613	0.46815694	down
49	AIF1	allograft inflammatory factor 1	0.02950167	1.57134314	up
50	AKAP10	A kinase (PRKA) anchor protei	0.000517354	1.71122266	up
51	AKAP13	A kinase (PRKA) anchor protei	0.002103903	1.5742303	up
52	AKAP9	A kinase (PRKA) anchor protei	0.005844207	1.71586831	up
53	AKIP1	A kinase (PRKA) interacting pr	0.000315912	2.00172327	up
54	AKNA	AT-hook transcription factor	0.001441774	1.69406137	up
55	AKR1C1	aldo-keto reductase family 1, m	0.013293665	0.39560101	down
56	AKT1	v-akt murine thymoma viral onc	0.000966921	1.7306602	up
57	AKT1S1	AKT1 substrate 1 (proline-rich)	0.012991231	0.60218825	down
58	AKT2	v-akt murine thymoma viral onc	0.005024719	0.45201022	down
59	AKTIP	AKT interacting protein	0.003233282	1.63418236	up
60	ALAD	aminolevulinate dehydratase	0.049235065	0.6564849	down
61	ALAS2	aminolevulinate, delta-, synthas	0.004880395	0.43783422	down
62	ALDH2	aldehyde dehydrogenase 2 fami	0.018180721	1.62017253	up
63	ALDH5A1	aldehyde dehydrogenase 5 fami	0.002234896	0.28459829	down
64	ALDH9A1	aldehyde dehydrogenase 9 fami	0.000840098	1.51611413	up
65	ALG13	ALG13, UDP-N-acetylglucosar	0.021735182	0.63736427	down
66	ALPK1	alpha-kinase 1	0.041368949	1.50355643	up
67	AMFR	autocrine motility factor recept	0.037937614	0.54258438	down
68	AMICA1	adhesion molecule, interacts wi	0.002440575	2.02092505	up
69	AMPD2	adenosine monophosphate dean	9.14E-05	1.98793743	up
70	ANK1	ankyrin 1, erythrocytic	0.00617151	0.28072389	down
71	ANK3	ankyrin 3, node of Ranvier (ank	0.011740374	1.54025876	up
72	ANKFY1	ankyrin repeat and FYVE doma	0.011793914	1.88486456	up
73	ANKH	ankylosis, progressive homolog	0.021043647	0.47318311	down
74	ANKHD1	ankyrin repeat and KH domain	0.002894458	1.73222223	up
75	ANKLE1	ankyrin repeat and LEM domai	0.044554255	0.43094525	down
76	ANKRD12	ankyrin repeat domain 12	0.013037369	1.53692795	up
77	ANKRD22	ankyrin repeat domain 22	0.011818152	0.65338777	down
78	ANKRD44	ankyrin repeat domain 44	0.001067684	1.65203843	up
79	ANKRD9	ankyrin repeat domain 9	0.007104475	0.36226002	down
80	ANKZF1	ankyrin repeat and zinc finger d	0.005119305	1.5113881	up
81	ANO6	anoctamin 6	0.005182837	1.79804889	up
82	ANP32A	acidic (leucine-rich) nuclear ph	0.018104536	1.60694169	up
83	ANPEP	alanyl (membrane) aminopeptid	0.006057952	1.91264206	up
84	ANTXR2	anthrax toxin receptor 2	0.003158669	1.61155147	up
85	ANXA11	annexin A11	0.003124323	1.79767845	up
86	ANXA5	annexin A5	0.010451473	1.76497777	up
87	AOAH	acyloxyacyl hydrolase (neutrop	0.01873521	1.73104472	up
88	AOC3	amine oxidase, copper containi	0.001279512	1.57057413	up
89	AP2B1	adaptor-related protein complex	0.000359215	0.3129241	down
90	AP2S1	adaptor-related protein complex	0.025790954	0.60624187	down
91	APBA2	amyloid beta (A4) precursor pro	0.000990745	2.18719144	up
92	APBB1IP	amyloid beta (A4) precursor pro	0.006803325	1.58620057	up
93	APLP2	amyloid beta (A4) precursor-lik	0.002233106	1.90327505	up
94	APOBEC2	apolipoprotein B mRNA editing	0.037253298	0.60131478	down
95	APOO	apolipoprotein O	0.005334628	0.64133993	down
96	APPBP2	amyloid beta precursor protein (	0.041330013	1.51425009	up
97	AQP1	aquaporin 1 (Colton blood grou	0.042218028	0.39088653	down
98	AQP3	aquaporin 3 (Gill blood group)	0.001988254	1.67644267	up
99	AQP9	aquaporin 9	0.025939327	1.59332555	up
100	ARAP1	ArfGAP with RhoGAP domain	6.58E-05	1.92325335	up
101	ARAP3	ArfGAP with RhoGAP domain	0.008299569	1.60843375	up
102	AREL1	apoptosis resistant E3 ubiquitin	0.002144794	1.58869108	up
103	ARF3	ADP-ribosylation factor 3	0.001976577	1.50515265	up
104	ARG1	arginase, liver	0.008563046	0.32060193	down
105	ARG2	arginase 2	0.000635699	0.22885942	down
106	ARHGAP1	Rho GTPase activating protein	0.000947041	1.59035432	up
107	ARHGAP23	Rho GTPase activating protein	0.014816982	0.56345414	down
108	ARHGAP25	Rho GTPase activating protein	0.000337217	2.05007914	up
109	ARHGAP27	Rho GTPase activating protein	0.01405332	1.57286929	up
110	ARHGAP30	Rho GTPase activating protein	0.005858886	1.56058091	up
111	ARHGAP9	Rho GTPase activating protein	0.004363885	1.76315964	up
112	ARHGDIA	Rho GDP dissociation inhibitor	0.0048667	1.52194383	up
113	ARHGDIB	Rho GDP dissociation inhibitor	0.007819349	1.63939322	up
114	ARHGEF12	Rho guanine nucleotide exchan	0.00817891	0.2971883	down
115	ARHGEF18	Rho/Rac guanine nucleotide ex	0.000530242	1.63101122	up
116	ARHGEF3	Rho guanine nucleotide exchan	0.008367495	1.54133588	up
117	ARHGEF37	Rho guanine nucleotide exchan	0.010546514	0.44839479	down
118	ARID1A	AT rich interactive domain 1A (	0.003836819	1.82011398	up
119	ARL2-SNX15	ARL2-SNX15 readthrough /// s	0.004810097	0.59033502	down
120	ARL4A	ADP-ribosylation factor-like 4A	0.000426656	0.1404554	down
121	ARMC8	armadillo repeat containing 8	0.000893459	0.62459311	down
122	ARNTL	aryl hydrocarbon receptor nucle	0.00086685	1.73733124	up
123	ARPC1B	actin related protein 2/3 comple	0.01184869	1.5323823	up
124	ARRDC3	arrestin domain containing 3	0.003904495	1.76880762	up
125	ARRDC4	arrestin domain containing 4	0.010210426	1.52850008	up
126	ARTN	artemin	0.003730037	0.53921958	down
127	ASAP1	ArfGAP with SH3 domain, ank	0.001507655	1.6906555	up
128	ASXL1	additional sex combs like 1 (Dr	0.009386558	0.43415799	down
129	ASXL2	additional sex combs like 2 (Dr	0.001786021	1.586546	up
130	ATG16L2	autophagy related 16-like 2 (S.	0.001105304	2.07742213	up
131	ATG4D	autophagy related 4D, cysteine	0.004005683	0.64691314	down
132	ATG9A	autophagy related 9A	0.022116714	0.64466261	down
133	ATM	ataxia telangiectasia mutated	0.017372744	1.76892247	up
134	ATP11A	ATPase, class VI, type 11A	0.018937942	1.65605887	up
135	ATP13A5	ATPase type 13A5 /// chromoso	0.003087298	1.75067051	up
136	ATP2B1	ATPase, Ca++ transporting, pla	0.012475522	1.80061574	up
137	ATP5E	ATP synthase, H+ transporting,	0.001560009	0.63660346	down
138	ATP6	ATP synthase F0 subunit 6 /// c	0.00441394	1.62536931	up
139	ATP6V1A	ATPase, H+ transporting, lysos	0.000967853	1.83383056	up
140	ATP6V1B2	ATPase, H+ transporting, lysos	0.003121955	1.86640131	up
141	ATP6V1D	ATPase, H+ transporting, lysos	4.36E-05	0.44925676	down
142	ATP8B2	ATPase, aminophospholipid tra	0.000552324	1.70933058	up
143	ATRX	alpha thalassemia/mental retard	0.006642266	1.58196508	up
144	ATXN1	ataxin 1	0.004272972	1.61527817	up
145	ATXN3	ataxin 3	0.007322886	0.64359325	down
146	ATXN7	ataxin 7	0.008333933	1.65480754	up
147	B4GALNT1	beta-1,4-N-acetyl-galactosamin	0.026769017	0.63171233	down
148	B4GALT2	UDP-Gal:betaGlcNAc beta 1,4-	0.034261764	0.5917783	down
149	B4GALT3	UDP-Gal:betaGlcNAc beta 1,4-	0.027141319	0.60235564	down
150	BABAM1	BRISC and BRCA1 A complex	0.013919128	0.60234255	down
151	BACH1	BTB and CNC homology 1, bas	0.021593884	1.62996616	up
152	BAHCC1	BAH domain and coiled-coil co	0.006712579	0.64835486	down
153	BANP	BTG3 associated nuclear protei	0.00040387	1.72575793	up
154	BASP1	brain abundant, membrane attac	0.022676157	1.72274882	up
155	BAZ2A	bromodomain adjacent to zinc f	0.004040234	1.63941971	up
156	BCAT1	branched chain amino-acid tran	0.012030421	0.51502387	down
157	BCL11B	B-cell CLL/lymphoma 11B (zin	0.000838603	1.88905479	up
158	BCL2	B-cell CLL/lymphoma 2	0.012508963	1.51468202	up
159	BCL2L1	BCL2-like 1	0.004920997	0.42123596	down
160	BCL2L11	BCL2-like 11 (apoptosis facilit	0.009255933	1.78350105	up
161	BCL2L13	BCL2-like 13 (apoptosis facilit	0.035770234	0.63520654	down
162	BCL7A	B-cell CLL/lymphoma 7A	0.031194004	0.66046093	down
163	BCLAF1	BCL2-associated transcription f	0.011117094	1.6206274	up
164	BEND2	BEN domain containing 2	0.010031015	1.86605979	up
165	BEST1	bestrophin 1	0.0149734	1.75469965	up
166	BEX1	brain expressed, X-linked 1	0.039642626	0.463751	down
167	BEX2	brain expressed X-linked 2	0.030459227	1.58187036	up
168	BICD2	bicaudal D homolog 2 (Drosop	0.001717314	1.68186166	up
169	BID	BH3 interacting domain death a	0.005098478	1.73261351	up
170	BIN2	bridging integrator 2	0.000848595	1.74058735	up
171	BIRC2	baculoviral IAP repeat containi	0.004350112	0.53117875	down
172	BIRC3	baculoviral IAP repeat containi	0.00596566	1.87037432	up
173	BIVM-ERCC5	BIVM-ERCC5 readthrough ///	0.028823844	1.50681494	up
174	BLOC1S5-TXNI	BLOC1S5-TXNDC5 readthrou	0.031532702	2.45779766	up
175	BMP2K	BMP2 inducible kinase	0.010956443	0.44868453	down
176	BNIP3L	BCL2/adenovirus E1B 19kDa i	0.000122021	0.275587	down
177	BOD1L1	biorientation of chromosomes i	0.000535046	1.83858267	up
178	BOLA3	bolA homolog 3 (E. coli)	0.004353119	0.52593082	down
179	BPGM	2,3-bisphosphoglycerate mutas	8.54E-05	0.09950665	down
180	BPTF	bromodomain PHD finger trans	0.01579964	1.5199689	up
181	BRAF	v-raf murine sarcoma viral onc	0.019487657	0.62034292	down
182	BRI3BP	BRI3 binding protein	0.030278743	1.53489498	up
183	BSG	basigin (Ok blood group)	0.002878489	0.43623893	down
184	BSPRY	B-box and SPRY domain conta	0.037763795	0.52021819	down
185	BTG1	B-cell translocation gene 1, anti	0.019399357	1.63122596	up
186	BTG2	BTG family, member 2	0.000426049	1.7003228	up
187	BTLA	B and T lymphocyte associated	0.019517715	1.74300405	up
188	BTN2A1	butyrophilin, subfamily 2, mem	0.025981545	1.50100509	up
189	BTN3A1	butyrophilin, subfamily 3, mem	0.03016672	1.56826084	up
190	BTN3A3	butyrophilin, subfamily 3, mem	0.023908362	1.55851264	up
191	C10orf10	chromosome 10 open reading fi	0.022970914	0.3834599	down
192	C10orf25	chromosome 10 open reading fi	0.027791673	0.6024589	down
193	C10orf54	chromosome 10 open reading fi	0.009048407	1.7516264	up
194	C10orf99	chromosome 10 open reading fi	0.005674153	1.71911359	up
195	C11orf58	chromosome 11 open reading fi	0.009958666	0.64060123	down
196	C11orf96	chromosome 11 open reading fi	0.014442848	0.50563937	down
197	C12orf10	chromosome 12 open reading fi	0.001064995	0.63661773	down
198	C12orf29	chromosome 12 open reading fi	0.005986398	0.54789588	down
199	C14orf159	chromosome 14 open reading fi	0.010665177	1.80572284	up
200	C15orf39	chromosome 15 open reading fi	0.001470192	1.96044737	up
201	C16orf54	chromosome 16 open reading fi	0.001147094	1.64801857	up
202	C19orf59	chromosome 19 open reading fi	0.028502375	0.38437687	down
203	C19orf77	chromosome 19 open reading fi	0.002427815	0.42809048	down
204	C1orf116	chromosome 1 open reading fra	0.018330027	0.34348372	down
205	C20orf112	chromosome 20 open reading fi	0.00101372	1.61112424	up
206	C20orf194	chromosome 20 open reading fi	0.001198842	0.34719667	down
207	C2CD2	C2 calcium-dependent domain	0.019645121	1.51377024	up
208	C2CD2	C2 calcium-dependent domain	0.00906029	0.63849718	down
209	C2orf68	chromosome 2 open reading fra	0.007084183	1.64657397	up
210	C5	complement component 5	0.007794989	0.53967313	down
211	C5AR1	complement component 5a rece	0.007248949	1.94022995	up
212	C6orf106	chromosome 6 open reading fra	0.005431427	0.40338503	down
213	C7orf31	chromosome 7 open reading fra	0.000387184	1.61356049	up
214	C9orf40	chromosome 9 open reading fra	0.000829327	0.2562862	down
215	C9orf64	chromosome 9 open reading fra	0.003710558	1.56165645	up
216	C9orf72	chromosome 9 open reading fra	0.043502079	1.50527706	up
217	C9orf78	chromosome 9 open reading fra	0.000654353	0.42940026	down
218	CA1	carbonic anhydrase I	0.000520147	0.13035908	down
219	CA2	carbonic anhydrase II	0.001187794	0.21323172	down
220	CA3	carbonic anhydrase III, muscle s	0.02600569	0.62369413	down
221	CACNA2D3	calcium channel, voltage-depen	0.000369742	2.4155158	up
222	CACUL1	CDK2-associated, cullin domai	0.002155862	1.53406457	up
223	CAMK2G	calcium/calmodulin-dependent	0.001775413	2.10460336	up
224	CAP1	CAP, adenylate cyclase-associa	0.006633564	1.64173082	up
225	CAPN12	calpain 12	0.018966603	0.58178166	down
226	CAPN2	calpain 2, (m/II) large subunit	0.035194857	1.53112407	up
227	CARD16	caspase recruitment domain fan	0.032976873	1.54777694	up
228	CARD17	caspase recruitment domain fan	0.012852931	0.6278212	down
229	CARD8	caspase recruitment domain fan	0.007281421	1.86325107	up
230	CARHSP1	calcium regulated heat stable pr	0.000167011	0.59317789	down
231	CASP1	caspase 1, apoptosis-related cys	0.037726895	1.60557068	up
232	CASP4	caspase 4, apoptosis-related cys	0.04454682	1.66823279	up
233	CASS4	Cas scaffolding protein family r	0.003406544	1.6698755	up
234	CAST	calpastatin	0.015018025	1.54501872	up
235	CAT	catalase	0.030709775	0.60887926	down
236	CBL	Cbl proto-oncogene, E3 ubiquit	0.000274153	1.90281632	up
237	CBWD1	COBW domain containing 1 ///	0.014290015	1.89803162	up
238	CBX1	chromobox homolog 1	0.019247372	1.50392081	up
239	CBX7	chromobox homolog 7	8.14E-05	1.79865321	up
240	CCDC109B	coiled-coil domain containing 1	0.040437459	1.76164377	up
241	CCDC176	coiled-coil domain containing 1	0.001022503	0.17759691	down
242	CCDC23	coiled-coil domain containing 2	0.001821987	0.55022229	down
243	CCDC34	coiled-coil domain containing 3	0.007825104	0.63357121	down
244	CCDC88C	coiled-coil domain containing 8	0.001401281	1.70993293	up
245	CCL4L1	chemokine (C-C motif) ligand 4	0.000161026	2.3572719	up
246	CCM2	cerebral cavernous malformatio	0.000874355	1.51628425	up
247	CCNA1	cyclin A1	0.016833046	0.63213683	down
248	CCNDBP1	cyclin D-type binding-protein 1	0.000679112	0.47495895	down
249	CCNG2	cyclin G2	0.016146281	1.59170033	up
250	CCNJL	cyclin J-like	0.008507621	2.25556935	up
251	CCNL1	cyclin L1	0.043921486	1.58662806	up
252	CCNY	cyclin Y	0.004234976	1.64428114	up
253	CCR2	chemokine (C-C motif) recepto	0.012674856	1.77252427	up
254	CCR3	chemokine (C-C motif) recepto	0.003878122	2.77938503	up
255	CCR5	chemokine (C-C motif) recepto	0.003259196	1.69985966	up
256	CCR7	chemokine (C-C motif) recepto	0.000400465	2.24464972	up
257	CCRL2	chemokine (C-C motif) recepto	0.006169301	0.64274034	down
258	CD14	CD14 molecule	0.021575433	1.64028236	up
259	CD177	CD177 molecule	0.040620523	0.18522532	down
260	CD19	CD19 molecule	0.005606377	0.6293567	down
261	CD1D	CD1d molecule	0.009828603	1.85219298	up
262	CD1E	CD1e molecule	0.001298615	1.57879293	up
263	CD2	CD2 molecule	0.004155444	1.61586971	up
264	CD200R1	CD200 receptor 1	0.009869875	1.66263043	up
265	CD244	CD244 molecule, natural killer	0.01847709	1.55558277	up
266	CD27	CD27 molecule	0.000483572	1.78189576	up
267	CD28	CD28 molecule	0.000482012	1.57078413	up
268	CD2AP	CD2-associated protein	0.011285015	0.59462619	down
269	CD300E	CD300e molecule	0.003648335	2.32624014	up
270	CD300LF	CD300 molecule-like family m	0.009004344	1.73740862	up
271	CD302	CD302 molecule /// LY75-CD3	0.017339433	1.79213108	up
272	CD36	CD36 molecule (thrombospond	0.021800752	1.75407287	up
273	CD37	CD37 molecule	0.005437186	1.50556589	up
274	CD3D	CD3d molecule, delta (CD3-TC	0.005185972	1.63694904	up
275	CD3E	CD3e molecule, epsilon (CD3-	0.00145129	1.6300645	up
276	CD4	CD4 molecule	0.001016186	1.59510282	up
277	CD44	CD44 molecule (Indian blood g	0.003079149	1.54061754	up
278	CD46	CD46 molecule, complement re	0.007090658	1.84684165	up
279	CD5	CD5 molecule	0.000447259	1.94404729	up
280	CD53	CD53 molecule	0.005132527	1.57703576	up
281	CD55	CD55 molecule, decay accelera	0.042700918	0.44509291	down
282	CD59	CD59 molecule, complement re	0.002215635	0.57029992	down
283	CD6	CD6 molecule	0.000739263	1.93460422	up
284	CD68	CD68 molecule	0.024588083	1.52130673	up
285	CD80	CD80 molecule	0.012391919	0.64777138	down
286	CD8B	CD8b molecule /// putative T-c	0.013150987	1.6130121	up
287	CD93	CD93 molecule	0.001956669	2.21378153	up
288	CD97	CD97 molecule	0.024349586	1.58849753	up
289	CDA	cytidine deaminase	0.028039529	1.62385863	up
290	CDC14B	cell division cycle 14B	0.000201326	2.01421443	up
291	CDC25A	cell division cycle 25A	0.030356087	0.59016664	down
292	CDC25B	cell division cycle 25B	0.000398625	1.70685521	up
293	CDC27	cell division cycle 27	0.001049254	0.66323724	down
294	CDC34	cell division cycle 34	0.022126105	0.58833063	down
295	CDC42EP3	CDC42 effector protein (Rho G	0.004587407	1.69735124	up
296	CDC42SE1	CDC42 small effector 1	0.005117415	1.58080468	up
297	CDIPT	CDP-diacylglycerol--inositol 3-	0.00567019	0.62836288	down
298	CDK1	cyclin-dependent kinase 1	0.037206184	0.41000458	down
299	CDK11A	cyclin-dependent kinase 11A ///	0.016435246	1.59637975	up
300	CDK13	cyclin-dependent kinase 13	0.013682384	1.70602996	up
301	CDK19	cyclin-dependent kinase 19	0.001263533	1.66689543	up
302	CDK5RAP3	CDK5 regulatory subunit assoc	0.005812308	1.55794556	up
303	CDK8	cyclin-dependent kinase 8	0.003779333	0.47830836	down
304	CDKL1	cyclin-dependent kinase-like 1 (	0.006405249	0.45275544	down
305	CDKN1B	cyclin-dependent kinase inhibit	0.00882971	1.56059887	up
306	CDKN2C	cyclin-dependent kinase inhibit	0.014934253	0.38387632	down
307	CDKN3	cyclin-dependent kinase inhibit	0.007963944	0.45389653	down
308	CDT1	chromatin licensing and DNA r	0.017474776	0.49192092	down
309	CEACAM4	carcinoembryonic antigen-relat	0.029484877	1.64387329	up
310	CEACAM6	carcinoembryonic antigen-relat	0.008801996	0.57585789	down
311	CEBPB	CCAAT/enhancer binding prote	0.019661136	1.62343289	up
312	CEBPD	CCAAT/enhancer binding prote	0.019741953	1.67155941	up
313	CECR1	cat eye syndrome chromosome	0.002568253	1.85698166	up
314	CELF1	CUGBP, Elav-like family mem	0.011500168	1.5662247	up
315	CELF2	CUGBP, Elav-like family mem	0.005924573	1.64902169	up
316	CEP19	centrosomal protein 19kDa	0.013538833	1.69304962	up
317	CEP350	centrosomal protein 350kDa	0.007616991	1.50855594	up
318	CEP63	centrosomal protein 63kDa	0.000594447	1.88749996	up
319	CERS4	ceramide synthase 4	0.006268854	1.55640815	up
320	CETN2	centrin, EF-hand protein, 2	0.001111793	0.46270324	down
321	CFD	complement factor D (adipsin)	0.008168079	1.89613177	up
322	CFLAR	CASP8 and FADD-like apopto	0.023482583	1.57799776	up
323	CFP	complement factor properdin	0.036971659	1.50238656	up
324	CHD2	chromodomain helicase DNA b	0.005078536	0.66698542	down
325	CHEK1	checkpoint kinase 1	0.02897348	0.57570395	down
326	CHI3L1	chitinase 3-like 1 (cartilage gly	0.017323935	2.50657421	up
327	CHKB	choline kinase beta /// CHKB-C	0.002345908	1.64612562	up
328	CHMP7	charged multivesicular body pro	0.000721369	1.67427273	up
329	CHP1	calcineurin-like EF-hand protei	0.002784971	0.49928885	down
330	CHPT1	choline phosphotransferase 1	0.004217872	0.41997238	down
331	CHRAC1	chromatin accessibility comple	0.009401892	1.60799363	up
332	CHST11	carbohydrate (chondroitin 4) su	0.006715988	1.54177127	up
333	CHST15	carbohydrate (N-acetylgalactos	0.01294828	1.83681427	up
334	CHST2	carbohydrate (N-acetylglucosan	0.045347794	0.63446169	down
335	CHST7	carbohydrate (N-acetylglucosan	0.000629711	1.75170247	up
336	CHURC1-FNTB	CHURC1-FNTB readthrough //	0.01386159	1.77066135	up
337	CISD2	CDGSH iron sulfur domain 2	7.51E-05	0.2024053	down
338	CITED2	Cbp/p300-interacting transactiv	0.008476376	0.23532915	down
339	CIZ1	CDKN1A interacting zinc finge	0.019134129	0.63182445	down
340	CKS1B	CDC28 protein kinase regulato	0.045566251	0.58429597	down
341	CLEC10A	C-type lectin domain family 10,	0.005856416	2.01904968	up
342	CLEC11A	C-type lectin domain family 11,	0.003719518	0.60554642	down
343	CLEC12A	C-type lectin domain family 12,	0.038935184	1.94102218	up
344	CLEC4A	C-type lectin domain family 4,	0.044945496	1.50340957	up
345	CLEC7A	C-type lectin domain family 7,	0.019397048	1.70395252	up
346	CLIC2	chloride intracellular channel 2	0.000442224	0.22954784	down
347	CLK2	CDC-like kinase 2	0.031938164	1.55493793	up
348	CLPB	ClpB caseinolytic peptidase B h	0.001717438	0.57988456	down
349	CLPX	ClpX caseinolytic peptidase X h	0.015653185	0.48200091	down
350	CLU	clusterin	0.017777161	1.77501687	up
351	CMAHP	cytidine monophospho-N-acety	0.015082168	0.60521724	down
352	CMAS	cytidine monophosphate N-acet	0.00024803	0.41134691	down
353	CMBL	carboxymethylenebutenolidase	0.003594216	0.41059041	down
354	CMIP	c-Maf inducing protein	0.000934353	1.83890291	up
355	CMTM2	CKLF-like MARVEL transmen	0.02079683	1.59939985	up
356	CNN2	calponin 2	0.003610102	1.55254314	up
357	CNOT1	CCR4-NOT transcription comp	0.004472584	1.5435594	up
358	CNOT2	CCR4-NOT transcription comp	0.03723739	1.54011821	up
359	CNPPD1	cyclin Pas1/PHO80 domain con	0.008536283	0.53308474	down
360	CNTNAP3	contactin associated protein-lik	0.00192998	2.15435345	up
361	COL17A1	collagen, type XVII, alpha 1	0.045707781	0.51880108	down
362	COL18A1	collagen, type XVIII, alpha 1	0.000320248	1.78911766	up
363	COL6A1	collagen, type VI, alpha 1	0.009868499	1.51132161	up
364	COPS2	COP9 signalosome subunit 2	0.019238365	0.55167435	down
365	CORO1A	coronin, actin binding protein, 1	0.015466967	1.5884234	up
366	CORO7	coronin 7	0.001433164	1.51119652	up

367	COTL1	coactosin-like 1 (Dictyostelium	0.006986936	1.79235553	up
368	CPD	carboxypeptidase D	0.015124675	1.59755611	up
369	CPEB4	cytoplasmic polyadenylation el	0.022419354	0.54155187	down
370	CPOX	coproporphyrinogen oxidase	0.030406342	0.65244284	down
371	CPPED1	calcineurin-like phosphoesteras	0.000276927	2.30374032	up
372	CPQ	carboxypeptidase Q	0.002536277	1.92394827	up
373	CPVL	carboxypeptidase, vitellogenic-l	0.019300973	1.87254223	up
374	CPXM1	carboxypeptidase X (M14 famil	0.029076421	0.52705629	down
375	CRB1	crumbs homolog 1 (Drosophila	0.017721617	0.60551773	down
376	CREB1	cAMP responsive element bind	0.01038017	1.58275804	up
377	CREB5	cAMP responsive element bind	0.031861062	1.65853583	up
378	CREBBP	CREB binding protein	0.000775281	1.85795789	up
379	CREBRF	CREB3 regulatory factor	0.00805002	1.51182108	up
380	CREG1	cellular repressor of E1A-stimu	5.58E-05	0.35526598	down
381	CRIPAK	cysteine-rich PAK1 inhibitor	0.000856719	1.90380518	up
382	CRIPT	cysteine-rich PDZ-binding prot	0.001141407	0.57276051	down
383	CRISP2	cysteine-rich secretory protein 2	0.03037749	0.52166407	down
384	CRISPLD1	cysteine-rich secretory protein L	0.036231612	0.64393851	down
385	CRISPLD2	cysteine-rich secretory protein L	0.007503471	1.84452167	up
386	CRTAM	cytotoxic and regulatory T cell	0.023985926	1.62791736	up
387	CRYBA2	crystallin, beta A2	0.002854347	0.66384741	down
388	CSAD	cysteine sulfinic acid decarboxy	0.01159115	1.63360004	up
389	CSAG2	CSAG family, member 2 /// CS	0.024247375	0.59774626	down
390	CSF1R	colony stimulating factor 1 rece	0.004537717	1.67864455	up
391	CSF2RA	colony stimulating factor 2 rece	0.018186659	1.62358231	up
392	CSF2RB	colony stimulating factor 2 rece	0.005667924	1.86697876	up
393	CSF3R	colony stimulating factor 3 rece	0.004872233	1.93478979	up
394	CSGALNACT1	chondroitin sulfate N-acetylgala	0.004784866	1.62748016	up
395	CSK	c-src tyrosine kinase	0.000513478	1.77625044	up
396	CSNK1G2	casein kinase 1, gamma 2	0.004040312	1.5431258	up
397	CST3	cystatin C	0.005083286	2.02493344	up
398	CTAGE5	CTAGE family, member 5	0.015362867	1.57639082	up
399	CTBS	chitobiase, di-N-acetyl-	0.009236532	1.70803797	up
400	CTLA4	cytotoxic T-lymphocyte-associa	0.005049836	1.831089	up
401	CTNNAL1	catenin (cadherin-associated pr	3.63E-05	0.14265129	down
402	CTSC	cathepsin C	0.003035206	1.57302575	up
403	CTSG	cathepsin G	0.040421019	0.13611569	down
404	CTSH	cathepsin H	0.014802399	1.65652414	up
405	CTSS	cathepsin S	0.007721784	1.83332336	up
406	CTTN	cortactin	0.039431214	1.58231867	up
407	CUL4A	cullin 4A	0.002502738	0.6251161	down
408	CUL9	cullin 9	0.00210251	1.65294765	up
409	CUX1	cut-like homeobox 1	0.019825398	1.62652062	up
410	CX3CR1	chemokine (C-X3-C motif) rece	0.019790838	1.70408177	up
411	CXCL5	chemokine (C-X-C motif) ligan	0.002248782	2.27819365	up
412	CXCR1	chemokine (C-X-C motif) recep	0.005311043	1.96701462	up
413	CXCR2	chemokine (C-X-C motif) recep	0.001331561	2.38651301	up
414	CXCR5	chemokine (C-X-C motif) recep	0.001462714	1.69270231	up
415	CYB561D1	cytochrome b561 family, memb	0.000769146	1.51792549	up
416	CYB5A	cytochrome b5 type A (microso	0.001711034	0.42718095	down
417	CYBB	cytochrome b-245, beta polype	0.049626384	1.75781136	up
418	CYBRD1	cytochrome b reductase 1	0.014576696	0.27861422	down
419	CYP2U1	cytochrome P450, family 2, sub	0.003782303	1.66316148	up
420	CYSTM1	cysteine-rich transmembrane m	0.005377993	0.36070392	down
421	CYTH4	cytohesin 4	0.001222939	1.98388945	up
422	CYTL1	cytokine-like 1	0.012997885	0.59982384	down
423	DAP	death-associated protein	0.001238146	0.59479672	down
424	DAPK2	death-associated protein kinase	0.005641715	1.55535002	up
425	DARC	Duffy blood group, chemokine	0.047584633	0.55074399	down
426	DAZAP2	DAZ associated protein 2	0.001906739	1.63532756	up
427	DBNDD2	dysbindin (dystrobrevin binding	0.001706176	1.5750278	up
428	DBNL	drebrin-like	0.003618542	1.52231005	up
429	DCAF10	DDB1 and CUL4 associated fa	0.016371604	0.4765696	down
430	DCAF11	DDB1 and CUL4 associated fa	0.004886601	0.58130236	down
431	DCAF16	DDB1 and CUL4 associated fa	0.044638633	1.54993731	up
432	DCAF6	DDB1 and CUL4 associated fa	0.036261067	0.64273601	down
433	DCK	deoxycytidine kinase	0.000789605	0.53972104	down
434	DCTN4	dynactin 4 (p62)	0.014805481	0.41800309	down
435	DCUN1D1	DCN1, defective in cullin nedd	0.006616772	0.30838461	down
436	DDAH2	dimethylarginine dimethylamin	0.042773716	0.59738764	down
437	DDI2	DNA-damage inducible 1 homo	0.00745071	0.51023859	down
438	DDX24	DEAD (Asp-Glu-Ala-Asp) box	0.019755628	1.58860685	up
439	DDX5	DEAD (Asp-Glu-Ala-Asp) box	0.033016527	1.54173394	up
440	DEF6	differentially expressed in FDC	0.00108992	1.63431487	up
441	DEF8	differentially expressed in FDC	0.002804111	1.95891073	up
442	DENND1A	DENN/MADD domain contain	0.020414979	0.59477793	down
443	DENND3	DENN/MADD domain contain	0.01912976	1.7485678	up
444	DENND4A	DENN/MADD domain contain	0.000713334	0.55345268	down
445	DENND4B	DENN/MADD domain contain	0.000455611	1.50744486	up
446	DENND5A	DENN/MADD domain contain	0.012916728	1.52024994	up
447	DGAT2	diacylglycerol O-acyltransfera	0.010786874	1.7838423	up
448	DGCR2	DiGeorge syndrome critical reg	0.001695983	1.55923246	up
449	DGKA	diacylglycerol kinase, alpha 80	0.000846931	1.82539849	up
450	DHRS7	dehydrogenase/reductase (SDR	0.004490032	1.5923386	up
451	DHX29	DEAH (Asp-Glu-Ala-His) box	0.00411817	0.58804418	down
452	DIP2B	DIP2 disco-interacting protein 2	0.016049246	1.58863972	up
453	DMTN	dematin actin binding protein	0.027499275	0.45300607	down
454	DNAJA4	DnaJ (Hsp40) homolog, subfam	0.041127678	0.50451712	down
455	DNAJB4	DnaJ (Hsp40) homolog, subfam	0.005360561	0.52564482	down
456	DNAJC3	DnaJ (Hsp40) homolog, subfam	0.002413723	1.73270486	up
457	DNAJC6	DnaJ (Hsp40) homolog, subfam	0.000536444	0.11368834	down
458	DOCK2	dedicator of cytokinesis 2	0.002228573	1.65065214	up
459	DOCK5	dedicator of cytokinesis 5	0.003854481	2.14239316	up
460	DOCK8	dedicator of cytokinesis 8	0.002377979	1.88246226	up
461	DOCK9	dedicator of cytokinesis 9	0.014514243	1.60982249	up
462	DOHH	deoxyhypusine hydroxylase/mo	0.002622133	0.51456314	down
463	DOPEY1	dopey family member 1	0.016719071	1.53441956	up
464	DPCD	deleted in primary ciliary dyski	0.004653819	0.3135898	down
465	DPEP2	dipeptidase 2	0.000188386	2.57969082	up
466	DPEP3	dipeptidase 3	0.003181071	1.60580837	up
467	DPP4	dipeptidyl-peptidase 4	0.007486561	1.68253067	up
468	DUSP1	dual specificity phosphatase 1	0.033738382	1.66995122	up
469	DUSP6	dual specificity phosphatase 6	0.014829896	1.88619558	up
470	DYNLL1	dynein, light chain, LC8-type 1	8.36E-05	0.49216899	down
471	DYRK3	dual-specificity tyrosine-(Y)-ph	0.000575746	0.17357637	down
472	E2F1	E2F transcription factor 1	0.046277146	0.45563014	down
473	E2F2	E2F transcription factor 2	0.016831895	0.47416597	down
474	ECHDC2	enoyl CoA hydratase domain c	0.008352562	1.54030811	up
475	EFCAB2	EF-hand calcium binding doma	0.02090514	0.59657642	down
476	EFHD2	EF-hand domain family, memb	0.005501989	1.80288079	up
477	EGFL7	EGF-like-domain, multiple 7	0.048536942	0.43899905	down
478	EGFL8	EGF-like-domain, multiple 8 ///	0.033673018	0.59151268	down
479	EGLN1	egl nine homolog 1 (C. elegans	0.018662693	1.67790504	up
480	EIF1B	eukaryotic translation initiation	0.001513134	0.51374726	down
481	EIF2AK1	eukaryotic translation initiation	0.031744304	0.45816653	down
482	EIF3J	eukaryotic translation initiation	0.003359251	0.51194455	down
483	EIF4A1	eukaryotic translation initiation	0.013371458	1.53343009	up
484	EIF5	eukaryotic translation initiation	0.00498178	0.57183798	down
485	EIF5A2	eukaryotic translation initiation	0.020445822	0.44492407	down
486	ELF2	E74-like factor 2 (ets domain tr	0.006512345	1.76987673	up
487	ELL2	elongation factor, RNA polyme	0.012852759	0.29576004	down
488	ELMSAN1	ELM2 and Myb/SANT-like do	0.000430634	1.72312209	up
489	ELOF1	elongation factor 1 homolog (S.	0.008588905	0.49318519	down
490	ELOVL6	ELOVL fatty acid elongase 6	0.003086637	0.25815978	down
491	EML3	echinoderm microtubule associ	0.003997374	1.50830603	up
492	EML4	echinoderm microtubule associ	0.001211669	1.9232712	up
493	EMP2	epithelial membrane protein 2	0.030533125	0.62467581	down
494	EMR1	egf-like module containing, mu	0.009250858	1.75372821	up
495	EMR2	egf-like module containing, mu	0.021356445	1.75193345	up
496	EMR3	egf-like module containing, mu	0.000735107	2.5642342	up
497	ENDOD1	endonuclease domain containin	0.007311268	0.55195392	down
498	ENSA	endosulfine alpha	0.001708607	0.44501979	down
499	ENTPD1	ectonucleoside triphosphate dip	0.026801965	1.5247851	up
500	EOMES	eomesodermin	0.044921972	1.61293759	up
501	EPB41	erythrocyte membrane protein b	0.01036146	0.31686783	down
502	EPB41L3	erythrocyte membrane protein b	0.003658884	2.31397678	up
503	EPB42	erythrocyte membrane protein b	0.029010888	0.49370188	down
504	EPDR1	ependymin related protein 1 (ze	0.037555095	0.60803401	down
505	EPHB1	EPH receptor B1	0.00534481	1.69169853	up
506	EPHX2	epoxide hydrolase 2, cytoplasm	0.002488771	2.43188853	up
507	EPM2A	epilepsy, progressive myoclonu	0.012780328	0.50786856	down
508	ERBB2IP	erbb2 interacting protein	0.032688064	1.52254082	up
509	ERG	v-ets erythroblastosis virus E26	0.049360282	0.41498919	down
510	ERMAP	erythroblast membrane-associat	0.025788352	0.4809788	down
511	ERMN	ermin, ERM-like protein	0.014896585	1.59155689	up
512	ETS1	v-ets erythroblastosis virus E26	0.009265687	1.81350901	up
513	EVI2B	ecotropic viral integration site 2	0.009467253	1.58215492	up
514	EVL	Enah/Vasp-like	0.001668561	1.62016716	up
515	EXT2	exostosin glycosyltransferase 2	0.001098114	0.64557392	down
516	EZH1	enhancer of zeste homolog 1 (D	0.000531362	1.6111478	up
517	F11R	F11 receptor	0.004928409	1.62251298	up
518	F13A1	coagulation factor XIII, A1 poly	0.001484728	2.14310252	up
519	F2RL1	coagulation factor II (thrombin)	0.002598822	2.07469177	up
520	FAH	fumarylacetoacetate hydrolase (	0.005966511	0.59244412	down
521	FAHD1	fumarylacetoacetate hydrolase	0.004449641	0.54245711	down
522	FAIM3	Fas apoptotic inhibitory molecu	0.001359463	1.80118429	up
523	FAM101B	family with sequence similarity	0.016805729	1.52199883	up
524	FAM102A	family with sequence similarity	0.000355848	1.98350349	up
525	FAM104A	family with sequence similarity	0.006552952	0.43813052	down
526	FAM104B	family with sequence similarity	0.000677687	0.33470458	down
527	FAM117A	family with sequence similarity	0.000542697	0.57789392	down
528	FAM117B	family with sequence similarity	0.018825258	1.62128853	up
529	FAM122A	family with sequence similarity	0.001782263	0.58195052	down
530	FAM129A	family with sequence similarity	0.002630503	1.91855531	up
531	FAM134A	family with sequence similarity	0.040397137	0.61617303	down
532	FAM168B	family with sequence similarity	0.004706239	1.63736386	up
533	FAM169A	family with sequence similarity	0.016857668	1.52938446	up
534	FAM174A	family with sequence similarity	0.000605256	2.13260176	up
535	FAM193B	family with sequence similarity	0.018670337	1.57993454	up
536	FAM198B	family with sequence similarity	0.016366146	1.76101045	up
537	FAM20B	family with sequence similarity	0.004590602	0.47690776	down
538	FAM210B	family with sequence similarity	0.041050313	0.49552625	down
539	FAM211A	family with sequence similarity	0.016543318	0.48062971	down
540	FAM212A	family with sequence similarity	0.025360697	0.55500041	down
541	FAM212B	family with sequence similarity	0.004288707	1.62891731	up
542	FAM45A	family with sequence similarity	0.008816959	1.71809687	up
543	FAM46A	family with sequence similarity	0.041411869	1.55511135	up
544	FAM46C	family with sequence similarity	0.001409641	0.20240865	down
545	FAM49A	family with sequence similarity	0.009786969	1.79588453	up
546	FAM49B	family with sequence similarity	0.023833347	1.57553845	up
547	FAM53B	family with sequence similarity	0.004797574	1.59453742	up
548	FAM53C	family with sequence similarity	0.005842284	1.56424087	up
549	FAM64A	family with sequence similarity	0.027553292	0.60914828	down
550	FAM65B	family with sequence similarity	0.001010121	2.03222131	up
551	FAM65C	family with sequence similarity	0.00602574	0.54070467	down
552	FAM78A	family with sequence similarity	0.00316682	1.54603669	up
553	FAM83A	family with sequence similarity	0.008657151	0.40511063	down
554	FAM83H	family with sequence similarity	0.000272028	1.56149192	up
555	FBXL20	F-box and leucine-rich repeat p	0.003644657	1.60057531	up
556	FBXL4	F-box and leucine-rich repeat p	0.000160568	0.43687513	down
557	FBXO38	F-box protein 38	0.04508917	1.50834498	up
558	FCGBP	Fc fragment of IgG binding pro	0.015930337	1.81158583	up
559	FCGR2A	Fc fragment of IgG, low affinity	0.019649828	1.6111011	up
560	FCGR2B	Fc fragment of IgG, low affinity	0.011216188	1.63182072	up
561	FCGR3A	Fc fragment of IgG, low affinity	0.035350355	1.56754097	up
562	FCGRT	Fc fragment of IgG, receptor, tr	0.002023907	1.96651317	up
563	FCHO2	FCH domain only 2	0.011840414	1.58047822	up
564	FCN1	ficolin (collagen/fibrinogen dom	0.04038514	1.58577479	up
565	FCRL2	Fc receptor-like 2	0.035749703	1.51013896	up
566	FECH	ferrochelatase	0.005659396	0.18573301	down
567	FEM1A	fem-1 homolog a (C. elegans)	0.038660571	0.60544236	down
568	FFAR2	free fatty acid receptor 2	0.016541999	1.733372	up
569	FGD3	FYVE, RhoGEF and PH domai	0.00032224	1.94136129	up
570	FGFR1OP2	FGFR1 oncogene partner 2	0.018591028	0.37065555	down
571	FGL2	fibrinogen-like 2	0.043006345	1.5985635	up
572	FGR	Gardner-Rasheed feline sarcom	0.019039613	1.60718438	up
573	FHDC1	FH2 domain containing 1	0.00393767	0.11540256	down
574	FHL2	four and a half LIM domains 2	0.012961101	0.42690464	down
575	FIS1	fission 1 (mitochondrial outer m	0.003758998	0.49190406	down
576	FKBP1B	FK506 binding protein 1B, 12.6	0.009303434	0.47150292	down
577	FKBP8	FK506 binding protein 8, 38kD	0.016106449	0.4375996	down
578	FLII	flightless I homolog (Drosophil	0.000637167	1.58776394	up
579	FLJ39632	uncharacterized LOC642477 ///	0.023486424	0.64112698	down
580	FLNA	filamin A, alpha	0.001186528	1.63132483	up
581	FLVCR1	feline leukemia virus subgroup	0.002956749	1.59002564	up
582	FMNL1	formin-like 1	0.001623526	1.6417065	up
583	FNBP1	formin binding protein 1	0.005360107	1.5601511	up
584	FNBP1L	formin binding protein 1-like	0.003640593	0.59245289	down
585	FNBP4	formin binding protein 4	0.03251389	1.50012814	up
586	FNIP1	folliculin interacting protein 1	0.002449371	1.89307229	up
587	FOSL2	FOS-like antigen 2	0.010852279	1.60454843	up
588	FOXO1	forkhead box O1	0.001995654	1.59949127	up
589	FOXO3	forkhead box O3 /// forkhead b	0.00496871	0.43239459	down
590	FOXO4	forkhead box O4	0.009258383	0.45689736	down
591	FOXP1	forkhead box P1	0.013707284	1.53170237	up
592	FPR1	formyl peptide receptor 1	0.012981153	1.67734126	up
593	FPR2	formyl peptide receptor 2	0.013539933	1.69242488	up
594	FRAT1	frequently rearranged in advanc	0.015722321	1.53310793	up
595	FRAT2	frequently rearranged in advanc	0.010359451	1.74380098	up
596	FRMD4A	FERM domain containing 4A	0.039162842	0.65166181	down
597	FRMD4B	FERM domain containing 4B	0.03099333	0.5452421	down
598	FRY	furry homolog (Drosophila)	0.001009329	2.25509364	up
599	FUCA2	fucosidase, alpha-L- 2, plasma	0.04465293	1.58673079	up
600	FUNDC2	FUN14 domain containing 2	0.004866897	0.48619768	down
601	FURIN	furin (paired basic amino acid c	0.016787419	0.55996601	down
602	FXYD5	FXYD domain containing ion t	0.006916443	1.51335394	up
603	FYB	FYN binding protein	0.005153575	1.7870795	up
604	FZD5	frizzled family receptor 5	1.06E-05	0.62206083	down
605	FZR1	fizzy/cell division cycle 20 rela	0.021231553	0.57518645	down
606	GAA	glucosidase, alpha; acid	0.00139791	1.75671309	up
607	GABARAPL2	GABA(A) receptor-associated p	6.97E-05	0.36621349	down
608	GADD45A	growth arrest and DNA-damage	0.000857514	0.19534029	down
609	GAPT	GRB2-binding adaptor protein,	0.027280311	1.51654459	up
610	GAPVD1	GTPase activating protein and V	0.0021593	0.66143472	down
611	GATA3	GATA binding protein 3	0.000131717	1.85807932	up
612	GCA	grancalcin, EF-hand calcium bi	0.031250346	1.57473697	up
613	GCAT	glycine C-acetyltransferase	0.003574032	0.58869755	down
614	GCC2	GRIP and coiled-coil domain c	0.033154228	2.22883383	up
615	GCLC	glutamate-cysteine ligase, catal	0.000498327	0.33478093	down
616	GCNT2	glucosaminyl (N-acetyl) transfe	0.003954071	0.61882712	down
617	GDF15	growth differentiation factor 15	0.038715612	0.25574296	down
618	GGA3	golgi-associated, gamma adapti	0.005871501	1.62128134	up
619	GGTA1P	glycoprotein, alpha-galactosyltr	0.006203695	1.73232759	up
620	GHITM	growth hormone inducible trans	0.001700558	0.56437224	down
621	GHRL	ghrelin/obestatin prepropeptide	0.006830052	1.62891796	up
622	GID4	GID complex subunit 4, VID24	0.000697904	0.39182	down
623	GID8	GID complex subunit 8 homolo	0.006456352	0.56580777	down
624	GIGYF2	GRB10 interacting GYF protein	0.001878872	1.51024634	up
625	GIMAP1-GIMAI	GIMAP1-GIMAP5 readthrough	0.001254711	1.53102129	up
626	GIMAP4	GTPase, IMAP family member	0.004662888	1.70359585	up
627	GIMAP6	GTPase, IMAP family member	0.013513229	1.54250325	up
628	GIMAP8	GTPase, IMAP family member	0.001196004	1.92414674	up
629	GLB1	galactosidase, beta 1	0.001413197	1.62541491	up
630	GLCCI1	glucocorticoid induced transcri	0.006515929	1.65734719	up
631	GLIPR1	GLI pathogenesis-related 1	0.024724744	1.58145397	up
632	GLRX5	glutaredoxin 5	0.001194973	0.39656943	down
633	GLT1D1	glycosyltransferase 1 domain c	0.020459579	1.5921178	up
634	GLUL	glutamate-ammonia ligase	0.020026129	0.52298649	down
635	GMCL1	germ cell-less, spermatogenesis	0.005895762	1.64861468	up
636	GMIP	GEM interacting protein	0.022617984	1.54326569	up
637	GMPR	guanosine monophosphate redu	0.018788284	0.39884288	down
638	GNAI2	guanine nucleotide binding prot	0.008691703	1.51032398	up
639	GNAQ	guanine nucleotide binding prot	0.030051592	1.52549121	up
640	GNS	glucosamine (N-acetyl)-6-sulfat	0.013776398	1.54354678	up
641	GOLPH3L	golgi phosphoprotein 3-like	0.001845602	0.60909158	down
642	GORASP1	golgi reassembly stacking prote	0.014935416	1.53559815	up
643	GPR146	G protein-coupled receptor 146	0.012967292	0.45528148	down
644	GPR155	G protein-coupled receptor 155	0.003189699	2.13194512	up
645	GPR183	G protein-coupled receptor 183	0.006454357	1.78724368	up
646	GPR34	G protein-coupled receptor 34	0.010813122	1.69374393	up
647	GPRASP1	G protein-coupled receptor ass	0.001658574	1.99904644	up
648	GPSM1	G-protein signaling modulator 1	0.006136538	0.63442797	down
649	GPSM3	G-protein signaling modulator 3	0.001895457	1.6390907	up
650	GRB10	growth factor receptor-bound p	0.034664193	0.58434865	down
651	GRB2	growth factor receptor-bound p	0.001971268	1.58692818	up
652	GRK5	G protein-coupled receptor kina	0.000959228	1.85641486	up
653	GSK3B	glycogen synthase kinase 3 beta	0.005087423	1.53864572	up
654	GSPT1	G1 to S phase transition 1	0.001736304	0.35408855	down
655	GYPA	glycophorin A (MNS blood gro	7.92E-05	0.12754109	down
656	GYPB	glycophorin B (MNS blood gro	0.002256076	0.12960513	down
657	GYPE	glycophorin E (MNS blood gro	0.001741885	0.21264836	down
658	H1F0	H1 histone family, member 0	0.049348669	0.24320338	down
659	H3F3A	H3 histone, family 3A /// H3 hi	0.015348381	1.58679668	up
660	HAL	histidine ammonia-lyase	0.001602258	2.43457499	up
661	HARS2	histidyl-tRNA synthetase 2, mit	0.004490926	1.54907804	up
662	HBD	hemoglobin, delta	0.001173883	0.28521636	down
663	HBE1	hemoglobin, epsilon 1	0.034568908	0.57225792	down
664	HBM	hemoglobin, mu	0.018246555	0.54311214	down
665	HBP1	HMG-box transcription factor 1	0.008908107	1.5034654	up
666	HBS1L	HBS1-like (S. cerevisiae)	0.019995969	0.24934141	down
667	HCAR2	hydroxycarboxylic acid recepto	0.036515589	1.58894621	up
668	HCK	hemopoietic cell kinase	0.006174495	1.86538945	up
669	HCLS1	hematopoietic cell-specific Lyn	0.000690399	1.78262394	up
670	HDGF	hepatoma-derived growth facto	2.24E-05	0.46646874	down
671	HEATR5A	HEAT repeat containing 5A	0.01870142	1.65388616	up
672	HECTD3	HECT domain containing E3 u	0.006922503	0.65747163	down
673	HEMGN	hemogen	0.000551871	0.16087699	down
674	HEPACAM2	HEPACAM family member 2	7.42E-05	0.16234074	down
675	HERC3	HECT and RLD domain contai	0.002810743	1.50264552	up
676	HERPUD1	homocysteine-inducible, endop	0.005789665	1.53171901	up
677	HERPUD2	HERPUD family member 2	0.004350064	1.59239359	up
678	HES6	hairy and enhancer of split 6 (D	0.004793433	0.24423931	down
679	HEXIM1	hexamethylene bis-acetamide i	0.00329079	0.5014506	down
680	HIP1	huntingtin interacting protein 1	0.010516209	1.89230664	up
681	HIPK1	homeodomain interacting prote	0.004216144	0.58413179	down
682	HIPK2	homeodomain interacting prote	0.00197685	1.74217918	up
683	HIST1H1C	histone cluster 1, H1c	0.000446614	0.34126107	down
684	HIST1H2AG	histone cluster 1, H2ag /// histo	0.001788649	0.64974793	down
685	HIST1H3F	histone cluster 1, H3f /// histon	0.010469992	0.6331342	down
686	HIVEP1	human immunodeficiency virus	0.007187768	1.60569795	up
687	HJURP	Holliday junction recognition p	0.001332067	0.65955124	down
688	HK1	hexokinase 1	0.015905894	0.39159698	down
689	HK2	hexokinase 2	0.011836942	1.50369775	up
690	HLA-A	major histocompatibility compl	0.049028998	1.70284847	up
691	HLA-E	major histocompatibility compl	0.014114273	1.58688709	up
692	HLA-F	major histocompatibility compl	0.022583989	1.52147523	up
693	HMBS	hydroxymethylbilane synthase	0.006208084	0.39420606	down
694	HMHA1	histocompatibility (minor) HA-	0.004295073	1.72916583	up
695	HNRNPA2B1	heterogeneous nuclear ribonucl	0.04986554	1.54067379	up
696	HNRNPD	heterogeneous nuclear ribonucl	0.025306823	1.80281087	up
697	HNRNPH3	heterogeneous nuclear ribonucl	0.037076833	1.51484687	up
698	HNRNPM	heterogeneous nuclear ribonucl	0.000246405	1.91271213	up
699	HP	haptoglobin	0.019068283	0.24454626	down
700	HPCAL4	hippocalcin like 4	0.011631811	1.50181228	up
701	HPS1	Hermansky-Pudlak syndrome 1	0.010631868	0.30773813	down
702	HPS3	Hermansky-Pudlak syndrome 3	0.01587447	1.52463734	up
703	HRSP12	heat-responsive protein 12	0.001121567	0.30117248	down
704	HSD17B11	hydroxysteroid (17-beta) dehyd	0.022246589	1.52476568	up
705	HSDL2	hydroxysteroid dehydrogenase l	0.0065466	1.59735118	up
706	HSP90B1	heat shock protein 90kDa beta (	0.028893585	1.84552312	up
707	HSPA12B	heat shock 70kD protein 12B	0.04722012	0.65128041	down
708	HSPA1A	heat shock 70kDa protein 1A //	0.008873297	1.69270081	up
709	HSPA5	heat shock 70kDa protein 5 (gl	0.030001392	1.65637427	up
710	HSPA6	heat shock 70kDa protein 6 (H	0.020769281	1.71833881	up
711	HVCN1	hydrogen voltage-gated channel	0.006992892	1.81256813	up
712	ICAM1	intercellular adhesion molecule	0.027215101	1.50706717	up
713	ICAM3	intercellular adhesion molecule	0.001473684	1.94318821	up
714	ICAM4	intercellular adhesion molecule	0.023382152	0.63015485	down
715	ICOS	inducible T-cell co-stimulator	0.000501029	1.56505934	up
716	IDS	iduronate 2-sulfatase	0.000132705	1.85274827	up
717	IFFO2	intermediate filament family or	0.000493538	1.51253469	up
718	IFI27	interferon, alpha-inducible prot	0.0019344	0.06505611	down
719	IFI30	interferon, gamma-inducible pr	0.04918749	1.50397368	up
720	IFIT1B	interferon-induced protein with	0.000342612	0.13333076	down
721	IFNAR2	interferon (alpha, beta and ome	0.003483703	1.65670468	up
722	IFNGR1	interferon gamma receptor 1	0.023147298	1.54371508	up
723	IFNGR2	interferon gamma receptor 2 (in	0.012936525	1.59522398	up
724	IFRD2	interferon-related development	3.24E-05	0.47402459	down
725	IFT20	intraflagellar transport 20 homo	0.012177272	0.66270217	down
726	IFT52	intraflagellar transport 52 homo	0.002101694	0.56153542	down
727	IGF1R	insulin-like growth factor 1 rece	0.000143785	2.22171985	up
728	IGF2	insulin-like growth factor 2 (so	0.009639666	0.05651719	down
729	IGF2BP2	insulin-like growth factor 2 mR	0.024048605	0.44629614	down
730	IGF2R	insulin-like growth factor 2 rece	0.000941531	2.17669576	up
731	IGH	immunoglobulin heavy locus ///	0.012631187	3.20462765	up
732	IGHA1	immunoglobulin heavy constan	0.012006202	3.13837606	up
733	IGHG1	immunoglobulin heavy constan	0.039995701	4.23005367	up
734	IGK	immunoglobulin kappa locus ///	0.017256774	2.312012	up

735	IGKV1-39	immunoglobulin kappa variable	0.049848444	2.31870862	up
736	IGLL3P	immunoglobulin lambda-like p	0.031320492	1.86041171	up
737	IKBIP	IKBKB interacting protein	0.005424626	1.64541322	up
738	IKBKB	inhibitor of kappa light polypep	0.005165394	1.50530006	up
739	IL10RA	interleukin 10 receptor, alpha	0.007313371	1.59120101	up
740	IL11RA	interleukin 11 receptor, alpha	0.002635468	1.68799845	up
741	IL13RA1	interleukin 13 receptor, alpha 1	0.001856239	2.32245293	up
742	IL16	interleukin 16	4.29E-05	1.86050721	up
743	IL17RA	interleukin 17 receptor A	0.006720197	1.80140685	up
744	IL23A	interleukin 23, alpha subunit p1	0.002334401	1.88049938	up
745	IL32	interleukin 32	0.012968193	1.55504051	up
746	IL6R	interleukin 6 receptor	0.000633707	2.29968964	up
747	IL7R	interleukin 7 receptor	0.012994954	1.59254793	up
748	IMPA2	inositol(myo)-1(or 4)-monopho	0.001859534	1.91599992	up
749	IMPDH1	IMP (inosine 5'-monophosphate	0.002234219	1.75466333	up
750	INADL	InaD-like (Drosophila)	0.002786375	1.92672464	up
751	INPP4A	inositol polyphosphate-4-phosp	0.011199651	1.57717205	up
752	IPCEF1	interaction protein for cytohesir	0.002578569	1.56294782	up
753	IQGAP1	IQ motif containing GTPase act	0.001207211	1.75904169	up
754	IQGAP2	IQ motif containing GTPase act	0.010871951	1.56349858	up
755	IQSEC1	IQ motif and Sec7 domain 1	0.005263063	1.54183355	up
756	IRF2	interferon regulatory factor 2	0.009489318	1.51073409	up
757	IRF2BPL	interferon regulatory factor 2 bi	0.001635993	1.63194855	up
758	IRF5	interferon regulatory factor 5	0.023756132	1.88554519	up
759	IRF8	interferon regulatory factor 8	0.009593531	1.66052719	up
760	IRF9	interferon regulatory factor 9	0.043700988	1.51909809	up
761	IRS2	insulin receptor substrate 2	0.004438192	1.64201048	up
762	ISCA1	iron-sulfur cluster assembly 1 h	0.000884392	0.19485016	down
763	ISCU	iron-sulfur cluster scaffold hom	0.00276751	0.6534767	down
764	ISY1-RAB43	ISY1-RAB43 readthrough /// R	0.008753401	1.50177867	up
765	ITGAL	integrin, alpha L (antigen CD11	0.000589244	2.03441132	up
766	ITGAX	integrin, alpha X (complement	0.005733909	1.88136117	up
767	ITGB2	integrin, beta 2 (complement co	0.001609235	1.70975681	up
768	ITGB7	integrin, beta 7	0.001685922	1.67404912	up
769	ITK	IL2-inducible T-cell kinase	0.003429659	1.64327474	up
770	ITM2B	integral membrane protein 2B	0.004466177	1.54450556	up
771	ITPKB	inositol-trisphosphate 3-kinase	7.48E-05	1.68953433	up
772	ITPR2	inositol 1,4,5-trisphosphate rece	0.021061376	1.55391922	up
773	ITPRIPL2	inositol 1,4,5-trisphosphate rece	0.031428121	1.53879171	up
774	ITSN1	intersectin 1 (SH3 domain prote	0.046263441	0.65367405	down
775	JAK1	Janus kinase 1	0.000142181	1.74531636	up
776	JAK3	Janus kinase 3	0.003508443	1.66022922	up
777	JAM3	junctional adhesion molecule 3	0.004797451	1.72510793	up
778	JARID2	jumonji, AT rich interactive do	0.00256751	1.64024499	up
779	JAZF1	JAZF zinc finger 1	0.005522858	0.33603212	down
780	JHDM1D	jumonji C domain containing hi	0.018472767	0.58385611	down
781	JMJD1C	jumonji domain containing 1C	0.021763799	1.55830292	up
782	JPH1	junctophilin 1	0.029386461	0.59622606	down
783	KANK2	KN motif and ankyrin repeat do	0.015147845	0.59784591	down
784	KANSL1	KAT8 regulatory NSL complex	0.007685488	1.52318496	up
785	KAT2B	K(lysine) acetyltransferase 2B	0.005063038	0.38406816	down
786	KAT6A	K(lysine) acetyltransferase 6A	0.003660574	1.65860516	up
787	KAT8	K(lysine) acetyltransferase 8	0.001100519	1.55741004	up
788	KATNBL1	katanin p80 subunit B-like 1	0.000134741	1.90845682	up
789	KBTBD11	kelch repeat and BTB (POZ) do	0.001327038	1.92788748	up
790	KCNE3	potassium voltage-gated channe	0.038171641	1.50699913	up
791	KCNH2	potassium voltage-gated channe	0.049357494	0.29038359	down
792	KCNJ2	potassium inwardly-rectifying c	0.018821939	1.63047693	up
793	KCTD12	potassium channel tetramerisati	0.011655412	2.09221636	up
794	KDELR3	KDEL (Lys-Asp-Glu-Leu) endo	0.015767644	0.61828564	down
795	KDM2A	lysine (K)-specific demethylase	0.001409603	1.64611437	up
796	KDM3A	lysine (K)-specific demethylase	0.032725757	1.52080708	up
797	KIAA0040	KIAA0040	0.029033238	1.61372545	up
798	KIAA0101	KIAA0101	0.017079986	0.6574869	down
799	KIAA0226L	KIAA0226-like	0.01472003	1.59568311	up
800	KIAA0247	KIAA0247	0.000814458	1.87663688	up
801	KIAA0319L	KIAA0319-like	0.013574533	1.51896147	up
802	KIAA0513	KIAA0513	0.003164125	1.73666768	up
803	KIAA0754	KIAA0754	0.02761139	1.55541419	up
804	KIAA1147	KIAA1147	0.003956745	1.55349963	up
805	KIAA1551	KIAA1551	0.00365845	1.67389757	up
806	KIAA1598	KIAA1598	0.019895024	1.87390663	up
807	KIAA1671	KIAA1671 /// uncharacterized I	0.000527716	1.81934824	up
808	KIF14	kinesin family member 14	0.038122805	0.65351875	down
809	KIF26A	kinesin family member 26A	0.026625939	0.27345681	down
810	KIF2C	kinesin family member 2C	0.023274347	0.64071442	down
811	KLF1	Kruppel-like factor 1 (erythroid	0.010605089	0.35837049	down
812	KLF11	Kruppel-like factor 11	0.004894734	1.73489251	up
813	KLF2	Kruppel-like factor 2 (lung)	0.005621096	1.56894538	up
814	KLF3	Kruppel-like factor 3 (basic)	0.030671957	0.23855238	down
815	KLF7	Kruppel-like factor 7 (ubiquitou	0.0085301	1.58560566	up
816	KLHL3	kelch-like family member 3	0.000285844	1.54808349	up
817	KLRF1	killer cell lectin-like receptor su	0.02645184	1.58737914	up
818	KMT2C	lysine (K)-specific methyltransf	0.00055717	1.76451511	up
819	KMT2E	lysine (K)-specific methyltransf	0.008656047	1.60244878	up
820	KPNA6	karyopherin alpha 6 (importin a	0.000613193	0.65786317	down
821	KPNB1	karyopherin (importin) beta 1	0.02994315	1.55231816	up
822	KRT1	keratin 1	0.007063347	0.27011769	down
823	KRT23	keratin 23 (histone deacetylase	0.008647098	2.19836616	up
824	LAMP2	lysosomal-associated membran	0.010689944	1.72139483	up
825	LANCL3	LanC lantibiotic synthetase com	0.01512499	0.58434158	down
826	LAPTM4B	lysosomal protein transmembra	0.007866596	0.61919202	down
827	LASP1	LIM and SH3 protein 1	0.002619818	1.62609322	up
828	LAT	linker for activation of T cells	0.001409622	1.56524498	up
829	LAX1	lymphocyte transmembrane ada	0.008277859	1.70500914	up
830	LBR	lamin B receptor	0.006875555	1.57854778	up
831	LCK	lymphocyte-specific protein tyr	0.000857913	1.73038557	up
832	LCP1	lymphocyte cytosolic protein 1	0.001395706	1.70303534	up
833	LCP2	lymphocyte cytosolic protein 2	0.006196002	1.67167612	up
834	LDLRAD2	low density lipoprotein receptor	0.021958314	0.623343	down
835	LENG8	leukocyte receptor cluster (LRC	0.000163786	1.52334915	up
836	LEPR	leptin receptor	0.008292023	0.49246638	down
837	LEPROTL1	leptin receptor overlapping tran	0.012807321	1.61106234	up
838	LFNG	LFNG O-fucosylpeptide 3-beta-	0.006087185	1.55422915	up
839	LGALS2	lectin, galactoside-binding, solu	0.018615885	1.63109463	up
840	LGALS3	lectin, galactoside-binding, solu	0.034449031	0.57018437	down
841	LGALSL	lectin, galactoside-binding-like	0.012103322	1.73069198	up
842	LHFPL2	lipoma HMGIC fusion partner-l	0.010565329	0.57149402	down
843	LHPP	phospholysine phosphohistidine	0.034266487	0.6504952	down
844	LILRA1	leukocyte immunoglobulin-like	0.002257422	2.27124863	up
845	LILRA2	leukocyte immunoglobulin-like	0.025625354	1.57760643	up
846	LILRA6	leukocyte immunoglobulin-like	0.034714901	1.53197384	up
847	LILRB1	leukocyte immunoglobulin-like	0.023972347	1.94418948	up
848	LILRB2	leukocyte immunoglobulin-like	0.02039468	1.90201819	up
849	LIPA	lipase A, lysosomal acid, choles	0.014615489	1.73495722	up
850	LITAF	lipopolysaccharide-induced TN	0.002372297	1.81523294	up
851	LNX2	ligand of numb-protein X 2	0.002346872	0.55658569	down
852	LOC100128751	INM04	0.009002625	1.51554476	up
853	LOC100129518	uncharacterized LOC10012951	0.006867319	1.72714504	up
854	LOC100130872	uncharacterized LOC10013087	0.031737896	1.70130192	up
855	LOC100131541	uncharacterized LOC10013154	0.007660101	2.34102156	up
856	LOC100288142	neuroblastoma breakpoint famil	0.006254244	1.95922925	up
857	LOC100507117	uncharacterized LOC10050711	0.009730639	1.54548298	up
858	LOC100507412	uncharacterized LOC10050741	0.012391733	0.57988493	down
859	LOC100653194	signal-regulatory protein beta-1	0.02797472	1.57118619	up
860	LOC100996732	uncharacterized LOC10099673	0.007068904	1.70402086	up
861	LOC100996752	uncharacterized LOC10099675	8.17E-05	0.22409728	down
862	LOC101059961	neuroblastoma breakpoint famil	0.004874817	1.69801832	up
863	LOC101060226	neuroblastoma breakpoint famil	0.000397758	1.77028446	up
864	LOC101060281	vesicular, overexpressed in can	0.000889208	1.58418434	up
865	LOC101060503	thioredoxin-interacting protein-	0.002677281	1.78214299	up
866	LOC101060545	ribose-5-phosphate isomerase-li	0.003750696	0.35554342	down
867	LOC101060692	neuroblastoma breakpoint famil	0.001250357	1.54334865	up
868	LOC101060714	sulfotransferase 1A3/1A4-like /	0.0019122	2.11159889	up
869	LOC128322	nuclear transport factor 2-like //	0.002400411	0.65163066	down
870	LOC254896	uncharacterized LOC254896 ///	0.00753214	1.78891221	up
871	LOC286052	uncharacterized LOC286052	0.02395486	1.65732664	up
872	LOC392288	microtubule-associated proteins	0.001253654	0.37140338	down
873	LOC728392	uncharacterized LOC728392 ///	4.69E-05	1.93219645	up
874	LOC81691	exonuclease NEF-sp	0.011775767	0.4923383	down
875	LPAR1	lysophosphatidic acid receptor	0.015665541	1.54417002	up
876	LPAR2	lysophosphatidic acid receptor	0.000483814	2.02240733	up
877	LPCAT2	lysophosphatidylcholine acyltra	0.014715715	1.6196584	up
878	LPGAT1	lysophosphatidylglycerol acyltr	0.00080903	1.7286578	up
879	LPXN	leupaxin	0.003472258	1.51365456	up
880	LRFN4	leucine rich repeat and fibronec	0.001313399	0.66427877	down
881	LRRC2	leucine rich repeat containing 2	0.013373914	0.33888612	down
882	LRRC28	leucine rich repeat containing 2	0.032030916	0.52027692	down
883	LRRC4	leucine rich repeat containing 4	0.021861824	1.52473427	up
884	LRRC8A	leucine rich repeat containing 8	0.047377705	0.64832914	down
885	LRRFIP1	leucine rich repeat (in FLII) inte	0.017446322	1.80930458	up
886	LRRK2	leucine-rich repeat kinase 2	0.030991479	1.62066514	up
887	LRRN3	leucine rich repeat neuronal 3	0.004202955	3.28307572	up
888	LSM12	LSM12 homolog (S. cerevisiae)	5.52E-05	0.57959565	down
889	LSP1	lymphocyte-specific protein 1	0.000753233	2.05712821	up
890	LST1	leukocyte specific transcript 1	0.008767369	1.82487726	up
891	LTB	lymphotoxin beta (TNF superfa	0.004304901	1.66076621	up
892	LTBP3	latent transforming growth fact	0.007933853	1.50801037	up
893	LY75	lymphocyte antigen 75	0.005634772	1.64715063	up
894	LYN	v-yes-1 Yamaguchi sarcoma vir	0.002522669	1.85356377	up
895	LYST	lysosomal trafficking regulator	0.017348237	1.64570871	up
896	LYZ	lysozyme	0.012787057	2.41129687	up
897	LZTFL1	leucine zipper transcription fact	0.014864331	0.45455078	down
898	MAF1	MAF1 homolog (S. cerevisiae)	0.042530167	0.65700959	down
899	MALAT1	metastasis associated lung aden	0.000788614	1.82302866	up
900	MAML1	mastermind-like 1 (Drosophila)	0.006030807	1.50523533	up
901	MAML2	mastermind-like 2 (Drosophila)	0.003122272	1.55963919	up
902	MAML3	mastermind-like 3 (Drosophila)	0.006996922	1.62960815	up
903	MAN1A1	mannosidase, alpha, class 1A, n	0.010045172	0.39369001	down
904	MAN1C1	mannosidase, alpha, class 1C, n	9.14E-05	2.16225346	up
905	MAN2B1	mannosidase, alpha, class 2B, n	0.000201888	1.74506991	up
906	MANBA	mannosidase, beta A, lysosomal	0.007293145	1.68721396	up
907	MANEAL	mannosidase, endo-alpha-like	0.045993831	0.63222719	down
908	MANSC1	MANSC domain containing 1	0.022096876	1.63448825	up
909	MAP1LC3B	microtubule-associated protein	0.003418878	0.44534077	down
910	MAP2K4	mitogen-activated protein kinas	0.001129611	1.52269635	up
911	MAP3K1	mitogen-activated protein kinas	0.002967665	1.67517577	up
912	MAP3K2	mitogen-activated protein kinas	0.007288472	1.57204765	up
913	MAP3K3	mitogen-activated protein kinas	0.002424295	1.69804822	up
914	MAP3K5	mitogen-activated protein kinas	0.001221943	1.80478112	up
915	MAP3K7CL	MAP3K7 C-terminal like	0.010374489	1.80667436	up
916	MAP4K4	mitogen-activated protein kinas	0.006601611	1.53117494	up
917	MAP4K5	mitogen-activated protein kinas	0.000224333	0.42386218	down
918	MAP7D1	MAP7 domain containing 1	0.00064991	1.79195913	up
919	MAPK1	mitogen-activated protein kinas	0.001816912	0.47778125	down
920	MARS	methionyl-tRNA synthetase	0.008722647	1.5258459	up
921	MAST3	microtubule associated serine/th	4.06E-05	2.04525185	up
922	MAU2	MAU2 chromatid cohesion fact	0.014067801	1.58087884	up
923	MBD4	methyl-CpG binding domain pr	0.015937037	1.69414133	up
924	MBNL1	muscleblind-like splicing regula	0.044603595	1.52488425	up
925	MBNL3	muscleblind-like splicing regula	0.003178242	0.34114	down
926	MBOAT2	membrane bound O-acyltransfe	0.013825137	0.58792218	down
927	MBOAT7	membrane bound O-acyltransfe	0.004235386	1.89111071	up
928	MBP	myelin basic protein	0.000769932	1.54054028	up
929	MCL1	myeloid cell leukemia sequence	0.00171006	1.84441084	up
930	MCM10	minichromosome maintenance	0.017077807	0.61239648	down
931	MCOLN1	mucolipin 1	0.016435485	0.49517462	down
932	MCUR1	mitochondrial calcium uniporte	0.019625189	0.6135857	down
933	MDFIC	MyoD family inhibitor domain	0.04793363	1.70641951	up
934	MED29	mediator complex subunit 29	0.010713739	1.59919588	up
935	MEFV	Mediterranean fever	0.016911681	1.74926821	up
936	MEGF6	multiple EGF-like-domains 6	0.000185004	1.96053489	up
937	METTL21D	methyltransferase like 21D	0.020900174	1.63461923	up
938	METTL22	methyltransferase like 22	0.008500116	0.62780897	down
939	METTL9	methyltransferase like 9	0.00041816	0.52329078	down
940	MFAP3L	microfibrillar-associated protein	0.020597858	1.58518634	up
941	MFHAS1	malignant fibrous histiocytoma	0.018330275	0.62623247	down
942	MFSD2B	major facilitator superfamily do	0.020507769	0.62548893	down
943	MFSD8	major facilitator superfamily do	0.039797569	1.54939994	up
944	MGST1	microsomal glutathione S-trans	0.012342283	1.60701376	up
945	MICAL1	microtubule associated monoox	0.007967996	1.54337174	up
946	MICAL3	microtubule associated monoox	0.035500167	0.4581486	down
947	MID1IP1	MID1 interacting protein 1	0.005227868	1.53097605	up
948	MIDN	midnolin	0.003575005	1.59869019	up
949	MINPP1	multiple inositol-polyphosphate	0.001349807	0.54324501	down
950	MIR21	microRNA 21 /// vacuole memb	0.001890659	1.9876645	up
951	MIR22	microRNA 22 /// MIR22 host g	0.004741028	0.51108821	down
952	MIR4680	microRNA 4680 /// programme	0.017949011	1.51527954	up
953	MIR4800	microRNA 4800 /// MAX dime	6.42E-05	0.44070733	down
954	MKLN1	muskelin 1, intracellular media	0.009686641	1.54133116	up
955	MLF1IP	MLF1 interacting protein	0.037212898	0.37564321	down
956	MLLT6	myeloid/lymphoid or mixed-lin	0.000391466	1.76108685	up
957	MMP25	matrix metallopeptidase 25	0.006795014	1.97701482	up
958	MMP8	matrix metallopeptidase 8 (neut	0.031328107	0.19113148	down
959	MNDA	myeloid cell nuclear differentia	0.010330412	1.73533428	up
960	MOB1B	MOB kinase activator 1B	0.0009645	0.3132579	down
961	MOB3A	MOB kinase activator 3A	0.000280368	1.99469787	up
962	MORN4	MORN repeat containing 4	0.031512388	0.53886097	down
963	MOSPD1	motile sperm domain containin	0.000909496	0.25286836	down
964	MOSPD2	motile sperm domain containin	0.005233954	1.84820191	up
965	MPC2	mitochondrial pyruvate carrier 2	0.000928447	0.43614832	down
966	MPEG1	macrophage expressed 1	0.004011515	2.13540724	up
967	MPP1	membrane protein, palmitoylate	0.004798503	0.41596036	down
968	MPPE1	metallophosphoesterase 1	7.70E-05	2.13105764	up
969	MPZL3	myelin protein zero-like 3	0.002516014	1.97117274	up
970	MRVI1	murine retrovirus integration sit	0.004399081	1.92943342	up
971	MS4A7	membrane-spanning 4-domains	0.036906052	2.03774881	up
972	MSI2	musashi RNA-binding protein 2	0.000651084	0.30384032	down
973	MSL1	male-specific lethal 1 homolog	0.004720258	1.90584958	up
974	MSMO1	methylsterol monooxygenase 1	0.001455194	0.54325569	down
975	MSN	moesin	0.002186644	1.82975256	up
976	MSRB1	methionine sulfoxide reductase	0.04091773	1.53266385	up
977	MT1G	metallothionein 1G	0.012150712	0.58445249	down
978	MT2A	metallothionein 2A	0.02540754	0.59213415	down
979	MTMR11	myotubularin related protein 11	0.010925103	1.77815615	up
980	MTSS1	metastasis suppressor 1	0.036869924	1.54963812	up
981	MVP	major vault protein	0.012611396	1.54226497	up
982	MX2	myxovirus (influenza virus) res	0.046821201	1.69359522	up
983	MXD1	MAX dimerization protein 1	0.008476501	1.84485978	up
984	MXI1	MAX interactor 1, dimerization	0.000786172	0.20500005	down
985	MYCL1	v-myc myelocytomatosis viral c	0.002760701	2.03598592	up
986	MYD88	myeloid differentiation primary	0.026487295	1.50382288	up
987	MYH9	myosin, heavy chain 9, non-mu	8.04E-05	1.89914398	up
988	MYL6B	myosin, light chain 6B, alkali, s	0.007232269	0.31590146	down
989	MYO18B	myosin XVIIIB	0.041912878	0.54529798	down
990	MYO1F	myosin IF	0.001202595	2.10599405	up
991	MYO5A	myosin VA (heavy chain 12, my	0.006781519	1.84429877	up
992	MYO9B	myosin IXB	0.001516322	1.75468221	up
993	NAA16	N(alpha)-acetyltransferase 16, N	0.019975638	1.50621437	up
994	NAA30	N(alpha)-acetyltransferase 30, N	0.021042378	0.56923329	down
995	NAAA	N-acylethanolamine acid amida	0.001290484	1.76276928	up
996	NADK	NAD kinase	0.008226542	1.8618468	up
997	NAGA	N-acetylgalactosaminidase, alp	0.004177132	1.73197632	up
998	NAMPT	nicotinamide phosphoribosyltra	0.035974869	1.50893291	up
999	NAP1L2	nucleosome assembly protein 1	0.001274546	1.86246222	up
1000	NAP1L4	nucleosome assembly protein 1	5.70E-06	0.64262749	down
1001	NAPA	N-ethylmaleimide-sensitive fac	0.049123996	0.50355965	down
1002	NAPG	N-ethylmaleimide-sensitive fac	0.000266342	0.45520232	down
1003	NAPSB	napsin B aspartic peptidase, pse	0.018685177	1.61675715	up
1004	NBEAL2	neurobeachin-like 2	0.016170531	1.58440206	up
1005	NBPF1	neuroblastoma breakpoint famil	0.000510929	1.79544781	up
1006	NBPF8	neuroblastoma breakpoint famil	0.000857958	1.57123457	up
1007	NCAPG	non-SMC condensin I complex,	0.043958496	0.56877696	down
1008	NCF1	neutrophil cytosolic factor 1 ///	0.011028204	1.81881342	up
1009	NCF2	neutrophil cytosolic factor 2	0.013063068	1.81304413	up
1010	NCF4	neutrophil cytosolic factor 4, 40	0.031947042	1.61555144	up
1011	NCOA1	nuclear receptor coactivator 1	0.001122107	1.59103558	up
1012	NCOA3	nuclear receptor coactivator 3	0.005550649	1.70709545	up
1013	NCOA4	nuclear receptor coactivator 4	0.003728258	0.46638589	down
1014	ND1	NADH dehydrogenase, subunit	0.000389814	2.07349143	up
1015	NDE1	nudE nuclear distribution E hon	0.000566439	1.76760292	up
1016	NDEL1	nudE nuclear distribution E hon	0.002578001	1.50651812	up
1017	NDFIP1	Nedd4 family interacting protei	0.022307316	1.55059679	up
1018	NDUFAF3	NADH dehydrogenase (ubiquin	5.96E-05	0.6094365	down
1019	NEDD4L	neural precursor cell expressed,	0.043348899	0.42949908	down
1020	NEDD9	neural precursor cell expressed,	0.00174675	1.69852524	up
1021	NEIL2	nei endonuclease VIII-like 2 (E.	0.010112848	0.65244437	down
1022	NEK1	NIMA-related kinase 1	0.008504527	0.61091859	down
1023	NELL1	NEL-like 1 (chicken)	0.046582469	0.62549754	down
1024	NELL2	NEL-like 2 (chicken)	0.000128231	2.70570687	up
1025	NFAM1	NFAT activating protein with I	0.009183234	1.7857714	up
1026	NFATC2IP	nuclear factor of activated T-cel	0.012836148	1.53592719	up
1027	NFIC	nuclear factor I/C (CCAAT-bin	0.007696861	1.5075257	up
1028	NFIX	nuclear factor I/X (CCAAT-bin	0.000316689	0.21413968	down
1029	NFRKB	nuclear factor related to kappaB	0.009340717	1.52691531	up
1030	NHS	Nance-Horan syndrome (conge	0.002941576	1.73319325	up
1031	NID1	nidogen 1	0.02135944	1.7313787	up
1032	NINJ1	ninjurin 1	0.010992321	1.54851411	up
1033	NKTR	natural killer-tumor recognition	0.021614293	1.61039251	up
1034	NLRP1	NLR family, pyrin domain cont	0.00014098	2.32234827	up
1035	NLRP12	NLR family, pyrin domain cont	0.032632894	1.52982453	up
1036	NME4	NME/NM23 nucleoside diphos	0.00171577	0.49106078	down
1037	NNMT	nicotinamide N-methyltransfera	0.016967868	0.6600248	down
1038	NOP2	NOP2 nucleolar protein	0.0012622	1.53809928	up
1039	NOTCH1	notch 1	0.001077962	1.86397803	up
1040	NOTCH2	notch 2	0.00092487	1.88276396	up
1041	NPTN	neuroplastin	0.023583008	1.50515402	up
1042	NR1D2	nuclear receptor subfamily 1, gr	0.037649719	1.54131315	up
1043	NR3C2	nuclear receptor subfamily 3, gr	0.001911583	1.54533693	up
1044	NRBF2	nuclear receptor binding factor	0.003345712	2.11842819	up
1045	NRGN	neurogranin (protein kinase C s	0.008425517	2.19598442	up
1046	NRIP3	nuclear receptor interacting pro	0.024249534	0.45411042	down
1047	NRN1	neuritin 1	0.025420532	0.41763455	down
1048	NSD1	nuclear receptor binding SET d	0.012321443	1.88235327	up
1049	NSFL1C	NSFL1 (p97) cofactor (p47)	0.002333005	1.53560178	up
1050	NSUN3	NOP2/Sun domain family, mem	0.000258801	0.33967595	down
1051	NSUN5P1	NOP2/Sun domain family, mem	0.006150091	1.79110699	up
1052	NT5C2	5'-nucleotidase, cytosolic II	0.016301404	1.60922257	up
1053	NTAN1	N-terminal asparagine amidase	0.007635237	0.40791161	down
1054	NUDT1	nudix (nucleoside diphosphate l	0.002786076	0.52827555	down
1055	NUMB	numb homolog (Drosophila)	0.001633227	2.13872901	up
1056	NUP50	nucleoporin 50kDa	0.0031452	1.57505747	up
1057	NUP98	nucleoporin 98kDa	0.013769478	0.54644043	down
1058	NUSAP1	nucleolar and spindle associate	0.019919135	0.61292431	down
1059	OAT	ornithine aminotransferase	0.013085126	0.56580637	down
1060	OGFRL1	opioid growth factor receptor-li	0.008703872	1.85789774	up
1061	OLIG1	oligodendrocyte transcription fa	0.001181236	2.55990382	up
1062	OPA1	optic atrophy 1 (autosomal dom	0.004864432	0.50572328	down
1063	OPTN	optineurin	0.000458898	0.45946015	down
1064	OR2T12	olfactory receptor, family 2, sub	0.012076104	0.60245305	down
1065	OR2W3	olfactory receptor, family 2, sub	0.048267665	0.4845509	down
1066	ORMDL3	ORM1-like 3 (S. cerevisiae)	0.025422207	0.65134408	down
1067	OS9	osteosarcoma amplified 9, endo	0.008837455	1.58024294	up
1068	OSBP2	oxysterol binding protein 2	0.03345218	0.46956508	down
1069	OSBPL11	oxysterol binding protein-like 1	0.005215982	1.53890299	up
1070	OSBPL2	oxysterol binding protein-like 2	0.003311432	1.64779481	up
1071	OSBPL8	oxysterol binding protein-like 8	0.045218288	1.50441628	up
1072	OSGEPL1	O-sialoglycoprotein endopeptid	0.01460298	1.50715609	up
1073	OSTF1	osteoclast stimulating factor 1	0.022906916	1.50028896	up
1074	OXNAD1	oxidoreductase NAD-binding d	0.009776999	1.52420663	up
1075	P2RY10	purinergic receptor P2Y, G-pro	0.000919648	1.69996771	up
1076	P2RY13	purinergic receptor P2Y, G-pro	0.010594643	1.86312134	up
1077	PA2G4	proliferation-associated 2G4, 3	0.006509955	0.51590539	down
1078	PACSIN3	protein kinase C and casein kin	0.004302324	0.63773332	down
1079	PAGE2	P antigen family, member 2 (pr	0.020665719	0.32315075	down
1080	PAK1	p21 protein (Cdc42/Rac)-activa	0.007023324	1.77543738	up
1081	PAK2	p21 protein (Cdc42/Rac)-activa	0.001170336	1.74307923	up
1082	PANK3	pantothenate kinase 3	0.008654883	0.53500662	down
1083	PAQR8	progestin and adipoQ receptor f	0.00101305	1.67188423	up
1084	PAQR9	progestin and adipoQ receptor f	0.004767419	0.19000397	down
1085	PARP4	poly (ADP-ribose) polymerase	0.007714045	1.50058411	up
1086	PARP8	poly (ADP-ribose) polymerase	0.00405251	1.52503851	up
1087	PASK	PAS domain containing serine/t	0.011644361	2.14505681	up
1088	PBX1	pre-B-cell leukemia homeobox	0.010312142	0.32602893	down
1089	PCCB	propionyl CoA carboxylase, bet	0.017581013	0.65058387	down
1090	PCDH1	protocadherin 1	0.042712315	0.53227706	down
1091	PCED1B	PC-esterase domain containing	0.000885214	1.79946157	up
1092	PCGF5	polycomb group ring finger 5	0.003491445	0.32192817	down
1093	PCMTD1	protein-L-isoaspartate (D-aspar	0.003830519	0.5387294	down
1094	PCNX	pecanex homolog (Drosophila)	0.003109967	1.6083244	up
1095	PCTP	phosphatidylcholine transfer pr	0.005625036	0.31230413	down
1096	PCYOX1	prenylcysteine oxidase 1	0.000320309	1.59708454	up
1097	PCYOX1L	prenylcysteine oxidase 1 like	0.002067351	1.64365958	up
1098	PDCD10	programmed cell death 10	0.012611889	0.60675485	down
1099	PDE3B	phosphodiesterase 3B, cGMP-i	0.005371154	1.6779717	up
1100	PDE7A	phosphodiesterase 7A	0.011385721	1.5371622	up
1101	PDGFC	platelet derived growth factor C	0.020931641	1.75265677	up
1102	PDIA4	protein disulfide isomerase fam	0.033047549	1.83760291	up

1103	PDK1	pyruvate dehydrogenase kinase,	0.0032741	1.56553138	up
1104	PDK4	pyruvate dehydrogenase kinase,	0.026979548	1.91260686	up
1105	PDLIM2	PDZ and LIM domain 2 (mystic	0.001495678	1.51564366	up
1106	PDPK1	3-phosphoinositide dependent p	0.003815916	1.59056677	up
1107	PDZD8	PDZ domain containing 8	0.030812355	0.58952894	down
1108	PEA15	phosphoprotein enriched in astr	0.007535787	1.69060018	up
1109	PECAM1	platelet/endothelial cell adhesio	0.000509018	2.32869885	up
1110	PELI1	pellino E3 ubiquitin protein liga	0.002520983	2.20914653	up
1111	PFKFB4	6-phosphofructo-2-kinase/fructo	0.006203678	1.86576093	up
1112	PGK1	phosphoglycerate kinase 1	0.025381758	1.5042049	up
1113	PGM2L1	phosphoglucomutase 2-like 1	0.009404096	0.52856443	down
1114	PHC2	polyhomeotic homolog 2 (Dros	0.003915079	1.92613177	up
1115	PHF17	PHD finger protein 17	0.001915472	1.54699661	up
1116	PHF20	PHD finger protein 20	0.000430527	1.53263534	up
1117	PHF20L1	PHD finger protein 20-like 1	0.023870843	1.57734275	up
1118	PI3	peptidase inhibitor 3, skin-deriv	0.001466795	2.69257854	up
1119	PI4KA	phosphatidylinositol 4-kinase, c	0.016128961	1.50136323	up
1120	PICALM	phosphatidylinositol binding cla	0.003204214	1.74537583	up
1121	PID1	phosphotyrosine interaction dor	0.001756932	2.41969313	up
1122	PIGX	phosphatidylinositol glycan anc	0.003965569	1.83665295	up
1123	PIK3CD	phosphatidylinositol-4,5-bispho	0.000764453	1.86541989	up
1124	PIK3IP1	phosphoinositide-3-kinase inter	0.00138522	1.90820437	up
1125	PIK3R2	phosphoinositide-3-kinase, regu	0.026822971	0.61258962	down
1126	PIK3R5	phosphoinositide-3-kinase, regu	0.002714943	1.72653951	up
1127	PILRA	paired immunoglobin-like type	0.004186839	2.03400865	up
1128	PIM1	pim-1 oncogene	0.003555693	0.41765071	down
1129	PINK1	PTEN induced putative kinase	0.031245465	0.52807846	down
1130	PIP4K2A	phosphatidylinositol-5-phospha	0.04375561	0.61572972	down
1131	PIP5K1B	phosphatidylinositol-4-phospha	5.75E-05	0.22514201	down
1132	PISD	phosphatidylserine decarboxyla	0.013102389	1.52648747	up
1133	PITHD1	PITH (C-terminal proteasome-i	0.002211256	0.31958732	down
1134	PITPNA	phosphatidylinositol transfer pr	0.002861113	1.61791715	up
1135	PKN2	protein kinase N2	0.030648558	1.50493827	up
1136	PLA2G12A	phospholipase A2, group XIIA	4.65E-05	1.56327701	up
1137	PLA2G7	phospholipase A2, group VII (p	0.012240616	1.66351858	up
1138	PLAGL1	pleiomorphic adenoma gene-lik	0.01331938	1.8542815	up
1139	PLCG1	phospholipase C, gamma 1	0.001465986	1.83608173	up
1140	PLEC	plectin	0.000978973	1.60503164	up
1141	PLEK	pleckstrin	0.01402825	1.67710854	up
1142	PLEKHA1	pleckstrin homology domain co	0.005494937	1.77480259	up
1143	PLEKHA2	pleckstrin homology domain co	0.019725635	1.55876974	up
1144	PLEKHG3	pleckstrin homology domain co	0.004230227	1.52530361	up
1145	PLEKHO1	pleckstrin homology domain co	0.030559768	1.53581631	up
1146	PLEKHO2	pleckstrin homology domain co	0.010478887	1.69531122	up
1147	PLSCR4	phospholipid scramblase 4	0.010900453	0.44404538	down
1148	PLXDC1	plexin domain containing 1	0.000957374	1.65066903	up
1149	PLXNC1	plexin C1	0.00819836	1.66691928	up
1150	PNP	purine nucleoside phosphorylas	4.15E-05	0.36428912	down
1151	POC1B	POC1 centriolar protein homol	0.00506956	0.34901626	down
1152	POLR1D	polymerase (RNA) I polypeptid	0.000166968	0.39076412	down
1153	POLR2A	polymerase (RNA) II (DNA dir	0.001402156	1.62169368	up
1154	POM121	POM121 transmembrane nucle	0.001203618	1.51551192	up
1155	POP7	processing of precursor 7, ribon	0.017337806	0.58526097	down
1156	POU6F1	POU class 6 homeobox 1	0.00065269	1.54215915	up
1157	PPCDC	phosphopantothenoylcysteine d	0.014451885	1.6127475	up
1158	PPFIA1	protein tyrosine phosphatase, re	0.006676319	1.96015067	up
1159	PPIAL4G	peptidylprolyl isomerase A (cyc	0.005607286	1.86224117	up
1160	PPIF	peptidylprolyl isomerase F	0.008172443	1.67153163	up
1161	PPIL2	peptidylprolyl isomerase (cyclo	0.001306995	1.58198481	up
1162	PPM1F	protein phosphatase, Mg2+/Mn	0.00094618	1.91462398	up
1163	PPM1M	protein phosphatase, Mg2+/Mn	0.007172101	1.57939462	up
1164	PPME1	protein phosphatase methyleste	0.004414542	0.4898515	down
1165	PPOX	protoporphyrinogen oxidase	0.038531329	0.64123234	down
1166	PPP1CB	protein phosphatase 1, catalytic	0.015052177	0.65629768	down
1167	PPP1R12B	protein phosphatase 1, regulato	0.001090809	1.68801779	up
1168	PPP1R2	protein phosphatase 1, regulato	0.030251494	1.53659351	up
1169	PPP1R3B	protein phosphatase 1, regulato	0.016741834	1.62779081	up
1170	PPP3CB	protein phosphatase 3, catalytic	0.004781227	0.49123586	down
1171	PPP3R1	protein phosphatase 3, regulato	0.009023597	0.48360182	down
1172	PPP6R2	protein phosphatase 6, regulato	0.001553813	1.54122145	up
1173	PPT1	palmitoyl-protein thioesterase 1	0.007344119	1.66152993	up
1174	PPTC7	PTC7 protein phosphatase hom	0.003743776	1.77456916	up
1175	PRAM1	PML-RARA regulated adaptor	0.005063971	1.54845567	up
1176	PRDM1	PR domain containing 1, with Z	0.004499443	1.63358895	up
1177	PRDX1	peroxiredoxin 1	0.041950948	0.65904217	down
1178	PRDX2	peroxiredoxin 2	0.005327403	0.29666083	down
1179	PREX1	phosphatidylinositol-3,4,5-trisp	0.004044761	1.61347308	up
1180	PRF1	perforin 1 (pore forming protein	0.04022436	1.50812895	up
1181	PRKACA	protein kinase, cAMP-depender	0.000962054	1.73328527	up
1182	PRKAG2	protein kinase, AMP-activated,	0.006703012	1.59943905	up
1183	PRKAR1A	protein kinase, cAMP-depender	0.031830995	1.50204202	up
1184	PRKCA	protein kinase C, alpha	0.001510955	1.61330589	up
1185	PRKCB	protein kinase C, beta	0.006081343	1.55355794	up
1186	PRKCD	protein kinase C, delta	0.013997085	1.63444889	up
1187	PRKCZ	protein kinase C, zeta	0.005938257	1.58117343	up
1188	PRKD2	protein kinase D2	0.011076229	1.65005323	up
1189	PRKDC	protein kinase, DNA-activated,	0.008150036	1.5791491	up
1190	PRRC2C	proline-rich coiled-coil 2C	0.000443738	1.76890346	up
1191	PRSS23	protease, serine, 23	0.014590217	1.66410328	up
1192	PRTN3	proteinase 3	0.003616352	0.53791929	down
1193	PSAP	prosaposin	0.002372635	1.86042686	up
1194	PSD4	pleckstrin and Sec7 domain con	0.002083047	1.60370401	up
1195	PSEN1	presenilin 1	0.004421745	1.5432784	up
1196	PSMD9	proteasome (prosome, macropa	0.001726521	0.60600447	down
1197	PSME4	proteasome (prosome, macropa	0.001860716	0.41378157	down
1198	PSMF1	proteasome (prosome, macropa	0.012080478	0.3714686	down
1199	PSTPIP1	proline-serine-threonine phosph	0.005927818	1.54999121	up
1200	PTAFR	platelet-activating factor recepto	0.000847926	2.24167549	up
1201	PTCD3	pentatricopeptide repeat domain	0.008075862	1.79922407	up
1202	PTEN	phosphatase and tensin homolog	0.01659418	1.54066966	up
1203	PTGDR2	prostaglandin D2 receptor 2	0.00574577	1.51358285	up
1204	PTGR1	prostaglandin reductase 1	0.038684345	0.55482576	down
1205	PTGS2	prostaglandin-endoperoxide syn	0.009850569	1.91111308	up
1206	PTPLA	protein tyrosine phosphatase-lik	0.018118293	0.12210576	down
1207	PTPN12	protein tyrosine phosphatase, no	0.020555488	1.56304404	up
1208	PTPN6	protein tyrosine phosphatase, no	0.008756181	1.59886111	up
1209	PTPRC	protein tyrosine phosphatase, re	0.006059232	1.61040676	up
1210	PTPRE	protein tyrosine phosphatase, re	0.010647227	1.63351295	up
1211	PTPRF	protein tyrosine phosphatase, re	0.000536371	0.3974547	down
1212	PTPRJ	protein tyrosine phosphatase, re	0.000799531	1.97575367	up
1213	PTPRK	protein tyrosine phosphatase, re	0.034510277	1.55282319	up
1214	PTPRN2	protein tyrosine phosphatase, re	0.017728718	1.63871993	up
1215	PTTG1	pituitary tumor-transforming 1 /	0.005656563	0.52741006	down
1216	PWWP2A	PWWP domain containing 2A	0.014994124	1.51163584	up
1217	PXDN	peroxidasin homolog (Drosophi	0.046618961	0.4078999	down
1218	PXMP2	peroxisomal membrane protein	0.030376399	0.59313505	down
1219	PXN	paxillin	0.000838341	2.18114568	up
1220	QKI	QKI, KH domain containing, R	0.02070073	1.68685869	up
1221	QPRT	quinolinate phosphoribosyltrans	0.031992779	0.61476073	down
1222	RAB11FIP1	RAB11 family interacting prote	0.003380887	2.30753488	up
1223	RAB11FIP4	RAB11 family interacting prote	0.001264226	1.82322217	up
1224	RAB13	RAB13, member RAS oncogen	0.0364327	0.42394666	down
1225	RAB27A	RAB27A, member RAS oncoge	0.014008422	1.53348639	up
1226	RAB2B	RAB2B, member RAS oncogen	0.014224148	0.41501325	down
1227	RAB31	RAB31, member RAS oncogen	0.026209459	1.71220526	up
1228	RAB37	RAB37, member RAS oncogen	0.008129519	1.61265594	up
1229	RAB6A	RAB6A, member RAS oncogen	0.00612615	0.40588794	down
1230	RAB6B	RAB6B, member RAS oncogen	0.039314179	0.38200875	down
1231	RABL5	RAB, member RAS oncogene f	0.009376473	0.62052554	down
1232	RAC2	ras-related C3 botulinum toxin	0.007100101	1.52727591	up
1233	RAD23A	RAD23 homolog A (S. cerevisi	0.007471052	0.41309424	down
1234	RAD51L3-RFFL	RAD51L3-RFFL readthrough /	0.00043283	1.56730225	up
1235	RAF1	v-raf-1 murine leukemia viral o	0.003759086	1.59375934	up
1236	RALBP1	ralA binding protein 1	0.013714522	0.64564278	down
1237	RALGPS1	Ral GEF with PH domain and S	0.006744184	1.54935034	up
1238	RANBP10	RAN binding protein 10	0.010941499	0.5212485	down
1239	RANBP2	RAN binding protein 2 /// RAN	0.016273579	1.73555467	up
1240	RAP1GAP	RAP1 GTPase activating protei	0.00024379	0.12331514	down
1241	RAP2A	RAP2A, member of RAS oncog	0.002012834	0.55053567	down
1242	RAPGEF3	Rap guanine nucleotide exchang	0.014361071	0.65462523	down
1243	RARA	retinoic acid receptor, alpha	0.008337758	1.71925457	up
1244	RASA3	RAS p21 protein activator 3	0.000452776	1.68548361	up
1245	RASA4	RAS p21 protein activator 4 ///	0.018103531	1.62273997	up
1246	RASAL3	RAS protein activator like 3	0.000768431	1.50655463	up
1247	RASGRP1	RAS guanyl releasing protein 1	0.003629951	1.61828385	up
1248	RASGRP4	RAS guanyl releasing protein 4	0.034345035	1.56588141	up
1249	RASSF2	Ras association (RalGDS/AF-6	0.003113343	1.88947022	up
1250	RASSF4	Ras association (RalGDS/AF-6	0.005172627	1.89920258	up
1251	RASSF5	Ras association (RalGDS/AF-6	0.000402651	1.70932801	up
1252	RBFOX2	RNA binding protein, fox-1 hor	0.032699783	0.60490092	down
1253	RBL2	retinoblastoma-like 2 (p130)	0.002632821	1.63707589	up
1254	RBM26	RNA binding motif protein 26	0.019433294	1.50120315	up
1255	RBM38	RNA binding motif protein 38	0.014182756	0.51157764	down
1256	RBM47	RNA binding motif protein 47	0.022520451	1.6310921	up
1257	RBM6	RNA binding motif protein 6	0.02222828	1.56409729	up
1258	RBX1	ring-box 1, E3 ubiquitin protein	0.001452449	0.49308788	down
1259	RCAN3	RCAN family member 3	0.00472002	1.58170367	up
1260	RCBTB2	regulator of chromosome conde	0.012529225	1.70904073	up
1261	RCOR1	REST corepressor 1	0.014412531	1.55775331	up
1262	RCOR3	REST corepressor 3	0.016430871	0.64847412	down
1263	REC8	REC8 homolog (yeast)	0.007342147	1.53114786	up
1264	REEP5	receptor accessory protein 5	0.019610907	1.57776377	up
1265	RELL1	RELT-like 1	0.006083077	1.94130279	up
1266	REM2	RAS (RAD and GEM)-like GT	0.011111391	1.5027533	up
1267	REPS2	RALBP1 associated Eps domai	0.009683409	1.85528935	up
1268	RERE	arginine-glutamic acid dipeptid	0.000575487	1.9858909	up
1269	REXO2	RNA exonuclease 2	0.000148475	0.27586715	down
1270	RFESD	Rieske (Fe-S) domain containin	0.002876943	0.28829235	down
1271	RFNG	RFNG O-fucosylpeptide 3-beta	0.018555779	0.60252101	down
1272	RFWD2	ring finger and WD repeat dom	0.009056058	1.58451408	up
1273	RGCC	regulator of cell cycle	0.009170882	0.57974645	down
1274	RGL2	ral guanine nucleotide dissociat	0.006374174	1.75607409	up
1275	RGS18	regulator of G-protein signaling	0.027965928	1.51044974	up
1276	RGS2	regulator of G-protein signaling	0.007745201	1.84086957	up
1277	RHAG	Rh-associated glycoprotein	0.001684875	0.15765743	down
1278	RHCE	Rh blood group, CcEe antigens	0.002183334	0.1688678	down
1279	RHD	Rh blood group, D antigen	0.019896672	0.24743423	down
1280	RHOG	ras homolog family member G	0.014475802	1.52509205	up
1281	RHOQ	ras homolog family member Q	0.006291218	1.75400907	up
1282	RICTOR	RPTOR independent companio	0.005795786	1.74937402	up
1283	RILP	Rab interacting lysosomal prote	0.013909041	0.58861887	down
1284	RIMS3	regulating synaptic membrane e	0.016927151	0.65518649	down
1285	RIN2	Ras and Rab interactor 2	0.027279563	1.62016084	up
1286	RIOK3	RIO kinase 3	9.53E-05	0.28587895	down
1287	RIPK1	receptor (TNFRSF)-interacting	0.003647457	1.56126074	up
1288	RIT1	Ras-like without CAAX 1	0.002315208	1.76666436	up
1289	RMND5A	required for meiotic nuclear div	0.005452792	0.38830488	down
1290	RNASE6	ribonuclease, RNase A family,	0.008483385	1.67270668	up
1291	RNASEL	ribonuclease L (2',5'-oligoisoad	0.035352663	1.54731239	up
1292	RNASET2	ribonuclease T2	0.000123451	2.26545287	up
1293	RNF10	ring finger protein 10	0.029711745	0.55926404	down
1294	RNF11	ring finger protein 11	0.004568405	0.31658338	down
1295	RNF111	ring finger protein 111	0.035605128	1.59477202	up
1296	RNF130	ring finger protein 130	0.004640595	1.64784442	up
1297	RNF14	ring finger protein 14	0.000453984	0.31379024	down
1298	RNF144B	ring finger protein 144B	0.016354986	1.73295429	up
1299	RNF166	ring finger protein 166	0.003920641	1.94569456	up
1300	RNF182	ring finger protein 182	0.048918998	0.17674915	down
1301	RNF187	ring finger protein 187	0.043670613	0.58187565	down
1302	RNF213	ring finger protein 213	0.028243318	1.6613009	up
1303	RNF24	ring finger protein 24	0.014864258	1.72432507	up
1304	RNF4	ring finger protein 4	0.001095155	1.70712866	up
1305	RNF44	ring finger protein 44	0.000358543	1.72709833	up
1306	RPGRIP1	retinitis pigmentosa GTPase reg	0.001835686	1.52721305	up
1307	RPL41	ribosomal protein L41	0.041569678	0.57884972	down
1308	RPS14	ribosomal protein S14	0.011155185	0.59905827	down
1309	RPS6KA1	ribosomal protein S6 kinase, 90	0.004437012	1.64670743	up
1310	RPS6KA3	ribosomal protein S6 kinase, 90	0.022042058	1.69567221	up
1311	RPS6KA5	ribosomal protein S6 kinase, 90	0.000249907	2.19557145	up
1312	RPS6KB2	ribosomal protein S6 kinase, 70	0.036063186	0.66648939	down
1313	RRAGB	Ras-related GTP binding B	0.001851758	0.49472487	down
1314	RSBN1L	round spermatid basic protein 1	0.002680421	1.61347252	up
1315	RSRC1	arginine/serine-rich coiled-coil	0.013982976	0.611985	down
1316	RSU1	Ras suppressor protein 1	0.015200466	0.66100935	down
1317	RTN1	reticulon 1	0.000896019	2.09652905	up
1318	RTN3	reticulon 3	0.011828904	1.62137708	up
1319	RUNDC3A	RUN domain containing 3A	0.04088991	0.41890772	down
1320	RUNX2	runt-related transcription factor	0.000121526	2.14392332	up
1321	RUNX3	runt-related transcription factor	0.040664778	1.67800163	up
1322	RWDD1	RWD domain containing 1	0.021186167	0.64386121	down
1323	S100A11	S100 calcium binding protein A	0.025954483	1.56254358	up
1324	S1PR1	sphingosine-1-phosphate recept	0.001713455	1.79499402	up
1325	S1PR3	sphingosine-1-phosphate recept	0.020311435	1.66248889	up
1326	SAP30	Sin3A-associated protein, 30kD	3.36E-06	0.66641646	down
1327	SART1	squamous cell carcinoma antige	0.002012597	1.51122058	up
1328	SASH1	SAM and SH3 domain containi	0.010478051	1.63257984	up
1329	SASH3	SAM and SH3 domain containi	0.002120561	1.69310027	up
1330	SCCPDH	saccharopine dehydrogenase (pu	0.024263491	0.65434932	down
1331	SCLT1	sodium channel and clathrin lin	0.049854555	1.50264277	up
1332	SCML4	sex comb on midleg-like 4 (Dro	0.000556733	1.60782262	up
1333	SCRN1	secernin 1	0.0008104	1.81992188	up
1334	SDC2	syndecan 2	0.000399239	1.51889565	up
1335	SDCBP	syndecan binding protein (synte	0.00644158	1.66955247	up
1336	SDPR	serum deprivation response	0.009474635	1.97034332	up
1337	SEC14L1	SEC14-like 1 (S. cerevisiae)	9.56E-05	1.83475362	up
1338	SEC62	SEC62 homolog (S. cerevisiae)	0.000100077	0.20848607	down
1339	SECISBP2	SECIS binding protein 2	0.007639944	1.77311243	up
1340	SEL1L3	sel-1 suppressor of lin-12-like 3	0.019576505	1.73774555	up
1341	SELENBP1	selenium binding protein 1	0.012764969	0.40611387	down
1342	SELK	selenoprotein K	0.004732453	0.53226765	down
1343	SELL	selectin L	0.022808385	1.53549282	up
1344	SELPLG	selectin P ligand	0.010100848	1.52540295	up
1345	SEMA3C	sema domain, immunoglobulin	0.02014432	1.54614003	up
1346	SEMA4A	sema domain, immunoglobulin	0.021641876	1.61033691	up
1347	SEMA4D	sema domain, immunoglobulin	0.001411407	1.93913637	up
1348	SERF1A	small EDRK-rich factor 1A (tel	0.014229663	0.62054703	down
1349	SERPINA1	serpin peptidase inhibitor, clade	0.007186914	2.03542804	up
1350	SERPINB3	serpin peptidase inhibitor, clade	0.031814563	0.46901015	down
1351	SERPINE1	serpin peptidase inhibitor, clade	0.035363727	0.6020778	down
1352	SERPINF1	serpin peptidase inhibitor, clade	0.008338064	1.57185529	up
1353	SERPINI1	serpin peptidase inhibitor, clade	0.003329165	0.45122994	down
1354	SETD1B	SET domain containing 1B	0.000166524	1.62549992	up
1355	SF3A1	splicing factor 3a, subunit 1, 12	0.000925109	1.52989029	up
1356	SFT2D3	SFT2 domain containing 3 /// W	0.013159611	1.57935542	up
1357	SGK1	serum/glucocorticoid regulated	0.002080019	2.29073749	up
1358	SGMS1	sphingomyelin synthase 1	0.035774296	1.64646236	up
1359	SGTB	small glutamine-rich tetratricop	0.026131278	1.52124957	up
1360	SH2B3	SH2B adaptor protein 3	0.004496872	1.67880443	up
1361	SH3BGRL2	SH3 domain binding glutamic a	0.006680974	2.09841235	up
1362	SH3BP1	SH3-domain binding protein 1	0.000612207	1.64252701	up
1363	SH3BP2	SH3-domain binding protein 2	0.03686308	1.5066385	up
1364	SH3KBP1	SH3-domain kinase binding pro	0.007639759	1.50844945	up
1365	SH3YL1	SH3 domain containing, Ysc84	0.026821569	1.55354429	up
1366	SIAH2	siah E3 ubiquitin protein ligase	0.002658818	0.33018128	down
1367	SIGLEC10	sialic acid binding Ig-like lectin	0.0047162	1.9473975	up
1368	SIGLEC17P	sialic acid binding Ig-like lectin	0.002630115	1.8730898	up
1369	SIGLEC7	sialic acid binding Ig-like lectin	0.001333805	2.07554454	up
1370	SIN3A	SIN3 transcription regulator ho	0.003206078	1.78010219	up
1371	SIN3B	SIN3 transcription regulator ho	0.000172691	1.62426112	up
1372	SIPA1L1	signal-induced proliferation-ass	0.00958834	1.50446757	up
1373	SIRPA	signal-regulatory protein alpha	0.004041793	1.89979557	up
1374	SIRPB2	signal-regulatory protein beta 2	0.006108735	1.75885173	up
1375	SIRT3	sirtuin 3	0.031375931	0.5377416	down
1376	SKAP1	src kinase associated phospho	0.002809962	1.53644778	up
1377	SKAP2	src kinase associated phospho	0.005310305	1.82740289	up
1378	SKI	v-ski sarcoma viral oncogene h	0.000508764	1.81522598	up
1379	SKP1	S-phase kinase-associated prote	0.000932517	0.5733233	down
1380	SLA	Src-like-adaptor	0.036964399	1.50578406	up
1381	SLC12A6	solute carrier family 12 (potassi	0.001757959	1.63659411	up
1382	SLC14A1	solute carrier family 14 (urea tr	0.001260318	0.17728194	down
1383	SLC15A3	solute carrier family 15, membe	0.002407059	1.58297788	up
1384	SLC15A4	solute carrier family 15, membe	0.002229466	1.76733109	up
1385	SLC16A1	solute carrier family 16, membe	0.011640842	0.4707162	down
1386	SLC1A5	solute carrier family 1 (neutral a	0.008882231	0.39359993	down
1387	SLC22A23	solute carrier family 22, membe	0.041266004	0.6356077	down
1388	SLC22A4	solute carrier family 22 (organi	0.041390857	0.54621745	down
1389	SLC26A11	solute carrier family 26, membe	0.00166352	1.57640483	up
1390	SLC2A1	solute carrier family 2 (facilitat	0.002969902	0.26293587	down
1391	SLC36A1	solute carrier family 36 (proton/	0.001816529	1.6187466	up
1392	SLC37A2	solute carrier family 37 (glycero	0.003392212	1.50026405	up
1393	SLC40A1	solute carrier family 40 (iron-re	0.048278528	0.64149374	down
1394	SLC43A1	solute carrier family 43, membe	0.01936898	0.65918845	down
1395	SLC43A2	solute carrier family 43, membe	0.002115515	1.56936499	up
1396	SLC45A4	solute carrier family 45, membe	0.000301402	2.3210682	up
1397	SLC46A2	solute carrier family 46, membe	0.001470998	1.80099517	up
1398	SLC48A1	solute carrier family 48 (heme t	0.004562948	0.44847071	down
1399	SLC4A1	solute carrier family 4, anion ex	0.017895479	0.39977394	down
1400	SLC50A1	solute carrier family 50 (sugar t	0.007197743	0.6649535	down
1401	SLC6A19	solute carrier family 6 (neutral a	0.016192586	0.52025723	down
1402	SLC6A6	solute carrier family 6 (neurotra	0.002321783	1.89977902	up
1403	SLC7A1	solute carrier family 7 (cationic	0.022352328	0.62138867	down
1404	SLC7A5	solute carrier family 7 (amino a	0.001543491	0.29050816	down
1405	SLC7A6	solute carrier family 7 (amino a	0.009291619	1.51896936	up
1406	SLC7A7	solute carrier family 7 (amino a	0.025116039	1.53489793	up
1407	SLC8A1	solute carrier family 8 (sodium/	0.004015712	1.9277308	up
1408	SLCO3A1	solute carrier organic anion tran	0.013677919	1.55203744	up
1409	SLMAP	sarcolemma associated protein	0.032864372	1.5012073	up
1410	SMAP2	small ArfGAP2	0.000200459	1.96823775	up
1411	SMARCA2	SWI/SNF related, matrix associ	0.001417525	1.7572522	up
1412	SMC2	structural maintenance of chron	0.007875882	0.58562497	down
1413	SMCHD1	structural maintenance of chron	0.009864829	1.69084205	up
1414	SMCR8	Smith-Magenis syndrome chror	0.006117975	1.59858047	up
1415	SMIM5	small integral membrane protei	0.003505462	0.41711257	down
1416	SMIM6	small integral membrane protei	0.030081832	0.5588947	down
1417	SMOX	spermine oxidase	0.001947999	0.38036958	down
1418	SMPD3	sphingomyelin phosphodiestera	0.002225788	2.55146832	up
1419	SNAP23	synaptosomal-associated protei	0.005891738	1.70243813	up
1420	SNCA	synuclein, alpha (non A4 comp	0.000717364	0.16620121	down
1421	SNN	stannin	0.000931892	1.9619776	up
1422	SNX1	sorting nexin 1	0.004959223	1.61606824	up
1423	SNX10	sorting nexin 10	0.003803559	1.96170578	up
1424	SNX15	sorting nexin 15	0.008504457	0.621642	down
1425	SNX18	sorting nexin 18	0.003770766	1.97120154	up
1426	SNX3	sorting nexin 3	0.0004054	0.3714193	down
1427	SNX30	sorting nexin family member 3(	0.008785188	1.56107125	up
1428	SNX4	sorting nexin 4	0.023198024	0.6366495	down
1429	SNX5	sorting nexin 5	0.00617469	0.35063876	down
1430	SNX8	sorting nexin 8	0.012230989	0.627428	down
1431	SOAT1	sterol O-acyltransferase 1	0.001953442	1.70106421	up
1432	SORBS1	sorbin and SH3 domain contain	0.021234664	0.62687264	down
1433	SORL1	sortilin-related receptor, L(DLR	0.000455903	2.23927385	up
1434	SOX4	SRY (sex determining region Y	0.043247588	0.45866486	down
1435	SOX6	SRY (sex determining region Y	0.001303725	0.22192805	down
1436	SP1	Sp1 transcription factor	0.004573715	1.6495034	up
1437	SP110	SP110 nuclear body protein	0.020938118	1.54015274	up
1438	SPAG6	sperm associated antigen 6	0.029119381	0.60618888	down
1439	SPAG9	sperm associated antigen 9	0.01681931	1.55923322	up
1440	SPARC	secreted protein, acidic, cystein	0.021554226	1.74752488	up
1441	SPECC1	sperm antigen with calponin ho	0.023319088	0.59146941	down
1442	SPEN	spen homolog, transcriptional r	0.001197952	1.66741118	up
1443	SPIN3	spindlin family, member 3	0.000184374	1.87540003	up
1444	SPOCK2	sparc/osteonectin, cwcv and ka	5.20E-05	1.84500458	up
1445	SPOPL	speckle-type POZ protein-like	0.010577168	1.70862238	up
1446	SPTA1	spectrin, alpha, erythrocytic 1 (	0.005639379	0.2429316	down
1447	SPTB	spectrin, beta, erythrocytic	0.020295603	0.36891632	down
1448	SRGAP2	SLIT-ROBO Rho GTPase activ	0.003677368	0.47759494	down
1449	SRI	sorcin	0.036128234	1.52413753	up
1450	SRPK2	SRSF protein kinase 2	0.004801792	1.73672234	up
1451	SRPR	signal recognition particle recep	0.000547386	1.57096177	up
1452	SRRD	SRR1 domain containing	0.00166475	0.44143118	down
1453	SRRM1	serine/arginine repetitive matrix	0.010484668	1.51246635	up
1454	SRRM2	serine/arginine repetitive matrix	0.000651949	1.89748417	up
1455	SRSF5	serine/arginine-rich splicing fac	0.009942731	1.78540768	up
1456	SSH2	slingshot protein phosphatase 2	0.000427993	1.82131584	up
1457	SSPN	sarcospan	0.001052767	1.54687448	up
1458	SSX2IP	synovial sarcoma, X breakpoint	0.004580273	0.4877465	down
1459	ST13	suppression of tumorigenicity 1	0.000703317	0.44948739	down
1460	ST20	suppressor of tumorigenicity 20	0.004076233	2.02910856	up
1461	ST3GAL6	ST3 beta-galactoside alpha-2,3-	0.004636367	1.79104931	up
1462	ST6GAL1	ST6 beta-galactosamide alpha-2	7.02E-05	1.84287423	up
1463	ST6GALNAC2	ST6 (alpha-N-acetyl-neuraminy	0.004696329	1.91837054	up
1464	ST6GALNAC4	ST6 (alpha-N-acetyl-neuraminy	0.000308324	0.36995416	down
1465	ST8SIA4	ST8 alpha-N-acetyl-neuraminid	0.001328601	1.96536787	up
1466	STAT3	signal transducer and activator (	0.006196206	1.62982317	up
1467	STAT5A	signal transducer and activator (	0.002299215	1.55406616	up
1468	STAT5B	signal transducer and activator (	0.001547669	1.67397849	up
1469	STEAP4	STEAP family member 4	0.029769602	1.57901119	up
1470	STK10	serine/threonine kinase 10	0.000839165	1.5776964	up

1471	STK11	serine/threonine kinase 11	0.00077258	0.48443499	down
1472	STK32B	serine/threonine kinase 32B	0.035085471	0.63744649	down
1473	STK4	serine/threonine kinase 4	0.000361223	1.81004913	up
1474	STOM	stomatin	1.25E-05	0.32257856	down
1475	STOML2	stomatin (EPB72)-like 2	0.003454399	0.64717785	down
1476	STX18	syntaxin 18	0.013275175	0.64105275	down
1477	STXBP5	syntaxin binding protein 5 (tom	0.001104267	1.75365962	up
1478	SUCLG2	succinate-CoA ligase, GDP-for	0.007870959	1.71424323	up
1479	SUDS3	suppressor of defective silencin	0.016042479	0.45512477	down
1480	SUGT1	SGT1, suppressor of G2 allele c	0.024361628	0.63356699	down
1481	SULF2	sulfatase 2	0.013303674	1.87332828	up
1482	SUN2	Sad1 and UNC84 domain conta	0.002822607	1.54528915	up
1483	SVIL	supervillin	0.002741339	2.21993387	up
1484	SWT1	SWT1 RNA endoribonuclease l	0.000374787	0.54056064	down
1485	SYK	spleen tyrosine kinase	0.007656613	1.62191528	up
1486	SYNE2	spectrin repeat containing, nucl	0.005302121	1.62814037	up
1487	SZRD1	SUZ RNA binding domain cont	0.002647323	0.63365595	down
1488	TAB3	TGF-beta activated kinase 1/M.	0.013780963	0.51802037	down
1489	TACC1	transforming, acidic coiled-coil	0.023543441	1.5204697	up
1490	TACC3	transforming, acidic coiled-coil	0.000358436	1.82439218	up
1491	TAF1C	TATA box binding protein (TB	0.0162955	1.51188058	up
1492	TAGAP	T-cell activation RhoGTPase ac	0.00175035	1.66846941	up
1493	TAGLN2	transgelin 2	0.020938614	1.52491929	up
1494	TAL1	T-cell acute lymphocytic leuker	0.016592669	0.52114019	down
1495	TAOK3	TAO kinase 3	0.002274529	1.63603738	up
1496	TAP2	transporter 2, ATP-binding cass	0.012193682	1.71673841	up
1497	TAPBP	TAP binding protein (tapasin)	0.003616665	1.63098674	up
1498	TARDBP	TAR DNA binding protein	0.027172187	1.52786478	up
1499	TARS	threonyl-tRNA synthetase	0.022819787	0.56754768	down
1500	TAX1BP1	Tax1 (human T-cell leukemia v	0.003696585	0.55456325	down
1501	TBC1D10C	TBC1 domain family, member	0.016614831	1.50541064	up
1502	TBC1D22B	TBC1 domain family, member	0.005036976	0.57878655	down
1503	TBC1D9	TBC1 domain family, member	0.014224222	1.7058893	up
1504	TBCEL	tubulin folding cofactor E-like	0.000708316	0.28452857	down
1505	TBL1X	transducin (beta)-like 1X-linke	0.000349018	1.95915747	up
1506	TBL1XR1	transducin (beta)-like 1 X-linke	0.003121354	0.40897336	down
1507	TBPL1	TBP-like 1	0.000546397	0.5790148	down
1508	TBRG1	transforming growth factor beta	0.009520693	1.52427747	up
1509	TBXAS1	thromboxane A synthase 1 (plat	0.003909725	2.06533057	up
1510	TCEA1	transcription elongation factor	0.013722139	0.59187788	down
1511	TCF19	transcription factor 19	0.026487101	0.57168177	down
1512	TCF3	transcription factor 3	7.90E-06	0.37403331	down
1513	TCF4	transcription factor 4	0.012063093	0.65861352	down
1514	TCF7	transcription factor 7 (T-cell sp	0.000292802	1.96782597	up
1515	TCIRG1	T-cell, immune regulator 1, AT	0.014064776	1.87068253	up
1516	TCP11L2	t-complex 11, testis-specific-lik	0.014702512	0.3414109	down
1517	TEP1	telomerase-associated protein 1	0.028485017	1.50517104	up
1518	TERF2IP	telomeric repeat binding factor	0.001061095	0.39479141	down
1519	TET2	tet methylcytosine dioxygenase	0.010823564	1.7358883	up
1520	TFCP2	transcription factor CP2	0.024813644	1.54582683	up
1521	TFDP1	transcription factor Dp-1	0.000452101	0.23250006	down
1522	TFDP2	transcription factor Dp-2 (E2F	0.000456558	0.28195709	down
1523	TFR2	transferrin receptor 2	0.036171047	0.41643862	down
1524	TFRC	transferrin receptor (p90, CD71	0.008410006	0.46607309	down
1525	TGFBI	transforming growth factor, bet	0.006413123	2.25644665	up
1526	TGFBR2	transforming growth factor, bet	0.00304034	1.6646197	up
1527	TGM2	transglutaminase 2 (C polypepti	0.012086875	0.30700482	down
1528	TGOLN2	trans-golgi network protein 2	0.000211542	1.92762699	up
1529	THBD	thrombomodulin	0.003690641	1.86066594	up
1530	THEM4	thioesterase superfamily memb	0.004698462	1.6388761	up
1531	THEM5	thioesterase superfamily memb	0.004669623	0.17100264	down
1532	THEMIS2	thymocyte selection associated	0.023293187	1.56538236	up
1533	THOC7	THO complex 7 homolog (Dro	0.000267181	0.49381417	down
1534	TIAM1	T-cell lymphoma invasion and	0.000495591	2.05096714	up
1535	TIGD3	tigger transposable element deri	9.37E-05	2.00545153	up
1536	TIMM23	translocase of inner mitochondr	0.004254042	0.55841556	down
1537	TIMP2	TIMP metallopeptidase inhibito	0.014922923	1.60895425	up
1538	TK1	thymidine kinase 1, soluble	0.011042806	0.46829024	down
1539	TLE3	transducin-like enhancer of spli	0.000237839	2.1547509	up
1540	TLR1	toll-like receptor 1	0.03513777	1.50757575	up
1541	TLR2	toll-like receptor 2	0.016576898	1.80998763	up
1542	TLR4	toll-like receptor 4	0.025547392	1.6018563	up
1543	TLR8	toll-like receptor 8	0.018633913	1.6728405	up
1544	TM7SF2	transmembrane 7 superfamily m	0.013961875	0.65678625	down
1545	TMBIM4	transmembrane BAX inhibitor	0.012323192	1.69107519	up
1546	TMBIM6	transmembrane BAX inhibitor	0.001389615	1.6321022	up
1547	TMC5	transmembrane channel-like 5	0.045917517	0.64350748	down
1548	TMC8	transmembrane channel-like 8	0.001092108	1.83906095	up
1549	TMCC1	transmembrane and coiled-coil	0.00021354	2.1499212	up
1550	TMCC2	transmembrane and coiled-coil	0.000319436	0.16953682	down
1551	TMCC3	transmembrane and coiled-coil	0.002392663	2.06843761	up
1552	TMEM121	transmembrane protein 121	0.008151751	0.56470387	down
1553	TMEM127	transmembrane protein 127	0.004861263	1.50180234	up
1554	TMEM131	transmembrane protein 131	0.012553348	1.5185277	up
1555	TMEM144	transmembrane protein 144	0.041822316	1.72610884	up
1556	TMEM14B	transmembrane protein 14B	0.005155957	0.47740659	down
1557	TMEM14C	transmembrane protein 14C	0.028869494	0.47141256	down
1558	TMEM154	transmembrane protein 154	0.006206769	1.83957748	up
1559	TMEM183A	transmembrane protein 183A /	0.002241393	0.56344793	down
1560	TMEM189	transmembrane protein 189 ///	0.002024443	0.51573753	down
1561	TMEM200B	transmembrane protein 200B	0.005763406	0.48752074	down
1562	TMEM217	transmembrane protein 217	0.043047965	0.51967645	down
1563	TMEM245	transmembrane protein 245	0.013208698	0.66182353	down
1564	TMEM43	transmembrane protein 43	0.004993609	1.55832496	up
1565	TMEM54	transmembrane protein 54 /// u	0.042804675	0.65151254	down
1566	TMEM56	transmembrane protein 56	0.007685576	0.27781715	down
1567	TMEM57	transmembrane protein 57	0.000685342	0.43048673	down
1568	TMEM63B	transmembrane protein 63B	0.028398954	0.6134981	down
1569	TMEM71	transmembrane protein 71	0.012847094	1.61909425	up
1570	TMEM86B	transmembrane protein 86B	0.026696345	0.47486887	down
1571	TMEM9B	TMEM9 domain family, memb	0.001402182	0.65525055	down
1572	TMOD1	tropomodulin 1	0.016969416	0.36386491	down
1573	TMOD2	tropomodulin 2 (neuronal)	0.010530182	1.6815351	up
1574	TMSB4X	thymosin beta 4, X-linked	0.007853185	1.54753544	up
1575	TNFAIP2	tumor necrosis factor, alpha-ind	0.004453087	2.19965245	up
1576	TNFRSF10B	tumor necrosis factor receptor s	0.0015434	1.70249294	up
1577	TNFRSF10C	tumor necrosis factor receptor s	0.004885913	1.99066171	up
1578	TNFRSF1A	tumor necrosis factor receptor s	0.007343444	1.64063458	up
1579	TNFRSF1B	tumor necrosis factor receptor s	0.003145631	1.88398705	up
1580	TNFRSF25	tumor necrosis factor receptor s	0.002570069	1.79155722	up
1581	TNIK	TRAF2 and NCK interacting ki	0.00655712	1.68150948	up
1582	TNPO1	transportin 1	0.000497274	0.52755296	down
1583	TNRC18	trinucleotide repeat containing	0.001433796	1.70108085	up
1584	TNRC6A	trinucleotide repeat containing	0.004095437	1.66306077	up
1585	TNRC6B	trinucleotide repeat containing	0.003542301	1.6232344	up
1586	TNRC6C	trinucleotide repeat containing	0.000241096	1.60649511	up
1587	TNS1	tensin 1	0.035899404	0.48267836	down
1588	TOM1L2	target of myb1-like 2 (chicken)	2.81E-05	1.50969049	up
1589	TOP1	topoisomerase (DNA) I	0.004619454	0.58219525	down
1590	TOR1AIP1	torsin A interacting protein 1	0.011417962	1.5824157	up
1591	TPCN1	two pore segment channel 1	0.0005685	1.74054547	up
1592	TPGS2	tubulin polyglutamylase comple	0.049245519	0.46730342	down
1593	TPM1	tropomyosin 1 (alpha)	0.012628615	0.29033369	down
1594	TPR	translocated promoter region, n	0.000168182	1.63333781	up
1595	TPRG1L	tumor protein p63 regulated 1-l	0.004432612	0.59957607	down
1596	TPX2	TPX2, microtubule-associated,	0.048424965	0.44447241	down
1597	TRABD2A	TraB domain containing 2A	0.000973144	1.792744	up
1598	TRAC	T cell receptor alpha constant /	0.00140616	1.80893306	up
1599	TRAF1	TNF receptor-associated factor	0.000301513	1.70490499	up
1600	TRAF3IP3	TRAF3 interacting protein 3	0.004526362	1.69004515	up
1601	TRAF5	TNF receptor-associated factor	0.003740309	1.57501105	up
1602	TRAK2	trafficking protein, kinesin bind	0.000447822	0.31012108	down
1603	TRAV17	T cell receptor alpha variable 1	0.004077881	1.76587466	up
1604	TRAV21	T cell receptor alpha variable 2	0.010196918	2.01009346	up
1605	TRBC1	T cell receptor beta constant 1	0.002595436	1.88682579	up
1606	TRBC2	T cell receptor beta constant 2 /	0.002986548	1.67182261	up
1607	TREM1	triggering receptor expressed or	0.00721597	1.94113395	up
1608	TRERF1	transcriptional regulating factor	0.002227279	1.61831261	up
1609	TRIB1	tribbles homolog 1 (Drosophila	0.033459423	1.55234098	up
1610	TRIM10	tripartite motif containing 10	0.027001942	0.42632994	down
1611	TRIM23	tripartite motif containing 23	0.0424995	0.63911426	down
1612	TRIM33	tripartite motif containing 33	0.004240319	1.517501	up
1613	TRIM34	tripartite motif containing 34 ///	0.019175651	1.54441733	up
1614	TRIM4	tripartite motif containing 4	0.001259904	1.50464435	up
1615	TRIM58	tripartite motif containing 58	0.014087832	0.40118264	down
1616	TRIM8	tripartite motif containing 8	0.002209133	1.72871777	up
1617	TSC1	tuberous sclerosis 1	0.034021305	0.59239233	down
1618	TSC22D4	TSC22 domain family, member	0.018533265	1.55850854	up
1619	TSEN34	tRNA splicing endonuclease 34	0.005019073	1.74756935	up
1620	TSHZ2	teashirt zinc finger homeobox 2	0.020072873	1.60977635	up
1621	TSHZ3	teashirt zinc finger homeobox 3	0.00699096	1.75139736	up
1622	TSPAN32	tetraspanin 32	0.003150116	1.51989148	up
1623	TSPAN5	tetraspanin 5	0.020772548	0.47543456	down
1624	TSPAN7	tetraspanin 7	0.001053408	0.16258297	down
1625	TSPO2	translocator protein 2	0.01609448	0.46566258	down
1626	TSTA3	tissue specific transplantation a	7.87E-06	0.2614081	down
1627	TTC25	tetratricopeptide repeat domain	0.049872437	0.57709964	down
1628	TTC3	tetratricopeptide repeat domain	0.007307026	1.53654008	up
1629	TTC39A	tetratricopeptide repeat domain	0.018870801	0.53027183	down
1630	TTC9	tetratricopeptide repeat domain	0.000571424	1.90705764	up
1631	TUBA1A	tubulin, alpha 1a	0.022058592	1.57013984	up
1632	TUBB1	tubulin, beta 1 class VI	0.028006404	1.72641448	up
1633	TUBB4B	tubulin, beta 4B class IVb	0.004472999	0.62657062	down
1634	TUBG1	tubulin, gamma 1	0.005551672	0.47445322	down
1635	TXK	TXK tyrosine kinase	0.017459176	1.56660633	up
1636	TXN	thioredoxin	0.000496552	0.51084265	down
1637	TYK2	tyrosine kinase 2	0.000108014	1.88739918	up
1638	TYROBP	TYRO protein tyrosine kinase	0.018212408	1.63910677	up
1639	UBAC1	UBA domain containing 1	0.005997387	0.64082455	down
1640	UBALD1	UBA-like domain containing 1	0.042018768	0.58163738	down
1641	UBALD2	UBA-like domain containing 2	0.001292342	0.63816958	down
1642	UBAP1	ubiquitin associated protein 1	0.00682955	0.63345777	down
1643	UBASH3A	ubiquitin associated and SH3 d	0.000107135	1.63741406	up
1644	UBE2C	ubiquitin-conjugating enzyme E	0.048911014	0.58025429	down
1645	UBE2F	ubiquitin-conjugating enzyme E	0.000251713	0.38340562	down
1646	UBE2H	ubiquitin-conjugating enzyme E	0.003363125	0.16337576	down
1647	UBE2M	ubiquitin-conjugating enzyme E	0.002416622	0.54461222	down
1648	UBE2O	ubiquitin-conjugating enzyme E	0.017333985	0.53962477	down
1649	UBE2S	ubiquitin-conjugating enzyme E	0.001084103	0.59512756	down
1650	UBE2V1	ubiquitin-conjugating enzyme E	0.003863265	0.55041895	down
1651	UBL3	ubiquitin-like 3	0.009722673	1.63070079	up
1652	UBL5	ubiquitin-like 5	0.011847221	0.6484768	down
1653	UBN1	ubinuclein 1	0.00194844	1.7519802	up
1654	UBQLN1	ubiquilin 1	0.00175353	0.50920985	down
1655	UBXN10	UBX domain protein 10	0.032544893	0.59924028	down
1656	UGT2B7	UDP glucuronosyltransferase 2	0.009497465	0.66059273	down
1657	UGT3A2	UDP glycosyltransferase 3 fami	0.046493739	0.45463699	down
1658	UNC119	unc-119 homolog (C. elegans)	0.006248836	1.62834654	up
1659	UNKL	unkempt homolog (Drosophila)	0.003443629	1.68732559	up
1660	UPF1	UPF1 regulator of nonsense tra	0.002586318	1.54122512	up
1661	UROD	uroporphyrinogen decarboxylas	0.000708524	0.40689244	down
1662	USP12	ubiquitin specific peptidase 12	0.001022841	0.27327385	down
1663	USP15	ubiquitin specific peptidase 15	0.002337186	1.74783375	up
1664	USP32	ubiquitin specific peptidase 32	0.02590811	0.59394578	down
1665	USP34	ubiquitin specific peptidase 34	0.007444598	1.54890066	up
1666	USP36	ubiquitin specific peptidase 36	0.001109808	1.66211789	up
1667	USP44	ubiquitin specific peptidase 44	0.033447925	1.57026208	up
1668	UVSSA	UV-stimulated scaffold protein	0.007140157	1.70720744	up
1669	VCAN	versican	0.011696531	2.39973956	up
1670	VCL	vinculin	0.003920653	1.57715104	up
1671	VDR	vitamin D (1,25- dihydroxyvita	0.005122907	1.62175056	up
1672	VNN3	vanin 3	0.019486879	1.75487695	up
1673	VPS13D	vacuolar protein sorting 13 hon	0.024227857	1.6752052	up
1674	VPS4B	vacuolar protein sorting 4 homo	0.003977467	1.52035045	up
1675	VTI1B	vesicle transport through intera	0.000964182	0.37446587	down
1676	WASF2	WAS protein family, member 2	0.002216546	0.54647558	down
1677	WDFY3	WD repeat and FYVE domain c	0.030622189	1.60879025	up
1678	WDR26	WD repeat domain 26	0.013938558	0.35151938	down
1679	WDR59	WD repeat domain 59	0.005449995	0.56912828	down
1680	WDR82	WD repeat domain 82	0.002912486	1.60450979	up
1681	WHAMMP2	WAS protein homolog associat	0.009965841	1.63009226	up
1682	WHAMMP3	WAS protein homolog associat	0.013420609	1.53066592	up
1683	WIPF1	WAS/WASL interacting protei	0.002804217	1.96225428	up
1684	WIPI2	WD repeat domain, phosphoino	0.027656138	0.62202435	down
1685	WLS	wntless homolog (Drosophila)	0.000900028	2.75330977	up
1686	WNK1	WNK lysine deficient protein k	0.035054478	0.56170549	down
1687	XK	X-linked Kx blood group (McL	0.002084598	0.1533771	down
1688	XPC	xeroderma pigmentosum, comp	4.79E-06	1.61166177	up
1689	XPO6	exportin 6	0.00309736	1.79791926	up
1690	XPO7	exportin 7	0.013402313	0.44842932	down
1691	XRRA1	X-ray radiation resistance assoc	0.00295556	1.66418316	up
1692	YBX3	Y box binding protein 3	0.001331413	0.58223671	down
1693	YIPF2	Yip1 domain family, member 2	0.010976094	0.57251083	down
1694	YIPF6	Yip1 domain family, member 6	0.001982599	0.41336132	down
1695	YME1L1	YME1-like 1 ATPase	0.000917661	1.78612151	up
1696	YOD1	YOD1 deubiquitinase	0.000603317	0.17367329	down
1697	YPEL4	yippee-like 4 (Drosophila)	0.00522341	0.25223035	down
1698	YTHDC2	YTH domain containing 2	0.01117037	1.76157334	up
1699	YWHAB	tyrosine 3-monooxygenase/tryp	0.000510509	1.66803974	up
1700	ZAK	sterile alpha motif and leucine	0.003087159	0.53561595	down
1701	ZAP70	zeta-chain (TCR) associated pr	0.021916618	1.54858247	up
1702	ZBTB18	zinc finger and BTB domain co	0.001898844	1.8361584	up
1703	ZBTB38	zinc finger and BTB domain co	0.004076909	1.65326809	up
1704	ZBTB4	zinc finger and BTB domain co	0.00290946	1.52160133	up
1705	ZBTB44	zinc finger and BTB domain co	0.003380872	0.48838182	down
1706	ZC3H12D	zinc finger CCCH-type containi	0.025412175	1.5259045	up
1707	ZC3H18	zinc finger CCCH-type containi	0.000439752	1.52779601	up
1708	ZC3H4	zinc finger CCCH-type containi	0.00779417	1.62011755	up
1709	ZC3H6	zinc finger CCCH-type containi	0.008356111	1.54966842	up
1710	ZCCHC6	zinc finger, CCHC domain cont	0.00674839	1.57958704	up
1711	ZDHHC18	zinc finger, DHHC-type contair	0.003137385	1.78039235	up
1712	ZDHHC2	zinc finger, DHHC-type contair	0.037537059	0.54650991	down
1713	ZDHHC7	zinc finger, DHHC-type contair	0.000610855	1.69425195	up
1714	ZER1	zyg-11 related, cell cycle regula	0.044202653	0.51173588	down
1715	ZFAND3	zinc finger, AN1-type domain 3	0.017975772	0.59318465	down
1716	ZFAND5	zinc finger, AN1-type domain 5	0.005404975	0.43399889	down
1717	ZFAS1	ZNFX1 antisense RNA 1	0.015385121	1.65408626	up
1718	ZFP36	ZFP36 ring finger protein	0.00452043	1.58182199	up
1719	ZFP36L1	ZFP36 ring finger protein-like 1	0.007647624	1.58595808	up
1720	ZFP36L2	ZFP36 ring finger protein-like 2	0.001977893	1.93770082	up
1721	ZMAT2	zinc finger, matrin-type 2	0.013575348	0.49387707	down
1722	ZMIZ1	zinc finger, MIZ-type containin	0.005796695	1.64977672	up
1723	ZNF101	zinc finger protein 101	0.007122004	1.518347	up
1724	ZNF106	zinc finger protein 106	0.011070842	1.53420783	up
1725	ZNF169	zinc finger protein 169	0.017661082	1.5383029	up
1726	ZNF18	zinc finger protein 18	0.000187274	1.57352104	up
1727	ZNF182	zinc finger protein 182	0.045404924	1.50921192	up
1728	ZNF217	zinc finger protein 217	0.001322193	1.89841868	up
1729	ZNF23	zinc finger protein 23	0.032086298	0.38898411	down
1730	ZNF333	zinc finger protein 333	0.016903873	1.52552112	up
1731	ZNF33A	zinc finger protein 33A	0.016954081	1.62745161	up
1732	ZNF346	zinc finger protein 346	0.016985788	0.52112468	down
1733	ZNF362	zinc finger protein 362	0.005484642	1.61222294	up
1734	ZNF398	zinc finger protein 398	0.002644444	1.55343358	up
1735	ZNF428	zinc finger protein 428	0.007680841	0.60119877	down
1736	ZNF532	zinc finger protein 532	0.003450011	1.66637748	up
1737	ZNF573	zinc finger protein 573	0.002715033	1.82043419	up
1738	ZNF641	zinc finger protein 641	0.011577281	1.63433983	up
1739	ZNF652	zinc finger protein 652	5.90E-05	1.69120441	up
1740	ZNF653	zinc finger protein 653	0.01026177	0.60285113	down
1741	ZNF746	zinc finger protein 746	0.000783363	1.75159381	up
1742	ZNF815P	zinc finger protein 815, pseudo	0.016199125	0.63653176	down
1743	ZNRF1	zinc and ring finger 1, E3 ubiqu	0.000341115	0.38688925	down
1744	ZRANB1	zinc finger, RAN-binding dom	0.003101923	0.58497762	down
1745	ZSWIM6	zinc finger, SWIM-type contain	0.032252951	1.50060949	up
1746	ZWINT	ZW10 interacting kinetochore	0.044925225	0.63532735	down
1747	ZYG11B	zyg-11 family member B, cell c	0.024980105	1.51874477	up
1748	ZYX	zyxin	0.00275309	1.56488199	up

n

Table S4 Details of JHP putative targets

NO.	OFFICIAL_GENE_SYMBOL	Name	Species
1	EPHB2	EPH receptor B2(EPHB2)	Homo sapiens
2	RPL23A	ribosomal protein L23a(RPL23A)	Homo sapiens
3	EPOR	erythropoietin receptor(EPOR)	Homo sapiens
4	RFK	riboflavin kinase(RFK)	Homo sapiens
5	ABCC4	ATP binding cassette subfamily C	Homo sapiens
6	ABAT	4-aminobutyrate aminotransferase	Homo sapiens
7	ABCA1	ATP binding cassette subfamily A	Homo sapiens
8	RPL37	ribosomal protein L37(RPL37)	Homo sapiens
9	THNSL1	threonine synthase like 1(THNSL	Homo sapiens
10	ABL1	ABL proto-oncogene 1, non-recep	Homo sapiens
11	ABL2	ABL proto-oncogene 2, non-recep	Homo sapiens
12	MAN2A1	mannosidase alpha class 2A memb	Homo sapiens
13	GLTP	glycolipid transfer protein(GLTP)	Homo sapiens
14	ABO	ABO, alpha 1-3-N-acetylgalactosa	Homo sapiens
15	ACAA1	acetyl-CoA acyltransferase 1(AC	Homo sapiens
16	MAOA	monoamine oxidase A(MAOA)	Homo sapiens
17	ACACB	acetyl-CoA carboxylase beta(AC	Homo sapiens
18	MAOB	monoamine oxidase B(MAOB)	Homo sapiens
19	ACADM	acyl-CoA dehydrogenase medium	Homo sapiens
20	ACADS	acyl-CoA dehydrogenase short ch	Homo sapiens
21	ACADSB	acyl-CoA dehydrogenase short/br	Homo sapiens
22	MAP4	microtubule associated protein 4(	Homo sapiens
23	SIGMAR1	sigma non-opioid intracellular rec	Homo sapiens
24	ACHE	acetylcholinesterase (Cartwright b	Homo sapiens
25	MAT1A	methionine adenosyltransferase 1	Homo sapiens
26	MAT2A	methionine adenosyltransferase 2	Homo sapiens
27	RPS5	ribosomal protein S5(RPS5)	Homo sapiens
28	RPS6	ribosomal protein S6(RPS6)	Homo sapiens
29	ACO2	aconitase 2(ACO2)	Homo sapiens
30	ESR1	estrogen receptor 1(ESR1)	Homo sapiens
31	ACOX1	acyl-CoA oxidase 1(ACOX1)	Homo sapiens
32	ESR2	estrogen receptor 2(ESR2)	Homo sapiens
33	ESRRA	estrogen related receptor alpha(ES	Homo sapiens
34	RPS6KA3	ribosomal protein S6 kinase A3(R	Homo sapiens
35	BCKDK	branched chain keto acid dehydro	Homo sapiens
36	MB	myoglobin(MB)	Homo sapiens
37	ESRRB	estrogen related receptor beta(ES	Homo sapiens
38	NCF1	neutrophil cytosolic factor 1(NCF	Homo sapiens
39	ESRRG	estrogen related receptor gamma(	Homo sapiens
40	MBL2	mannose binding lectin 2(MBL2)	Homo sapiens
41	RPS8	ribosomal protein S8(RPS8)	Homo sapiens
42	ACTA1	actin alpha 1, skeletal muscle(AC	Homo sapiens
43	RPS9	ribosomal protein S9(RPS9)	Homo sapiens
44	ACTB	actin beta(ACTB)	Homo sapiens
45	MC1R	melanocortin 1 receptor(MC1R)	Homo sapiens
46	ETFDH	electron transfer flavoprotein deh	Homo sapiens
47	RPS13	ribosomal protein S13(RPS13)	Homo sapiens
48	AS3MT	arsenite methyltransferase(AS3M	Homo sapiens
49	ACTG1	actin gamma 1(ACTG1)	Homo sapiens
50	PTGR2	prostaglandin reductase 2(PTGR2	Homo sapiens
51	RPS17	ribosomal protein S17(RPS17)	Homo sapiens
52	RPS18	ribosomal protein S18(RPS18)	Homo sapiens
53	RPS19	ribosomal protein S19(RPS19)	Homo sapiens
54	TRAC	T cell receptor alpha constant(TR	Homo sapiens
55	TLR7	toll like receptor 7(TLR7)	Homo sapiens
56	AKR1A1	aldo-keto reductase family 1 mem	Homo sapiens
57	EXTL2	exostosin like glycosyltransferase	Homo sapiens
58	RPS28	ribosomal protein S28(RPS28)	Homo sapiens
59	ACVR1	activin A receptor type 1(ACVR1	Homo sapiens
60	ACVR1B	activin A receptor type 1B(ACVR	Homo sapiens
61	KDM5D	lysine demethylase 5D(KDM5D)	Homo sapiens
62	MDH1	malate dehydrogenase 1(MDH1)	Homo sapiens
63	PLA2G2E	phospholipase A2 group IIE(PLA	Homo sapiens
64	ACVRL1	activin A receptor like type 1(AC	Homo sapiens
65	NUDT9	nudix hydrolase 9(NUDT9)	Homo sapiens
66	MDH2	malate dehydrogenase 2(MDH2)	Homo sapiens
67	COMTD1	catechol-O-methyltransferase dom	Homo sapiens
68	RRM1	ribonucleotide reductase catalytic	Homo sapiens
69	RRM2	ribonucleotide reductase regulator	Homo sapiens
70	F2	coagulation factor II, thrombin(F2	Homo sapiens
71	ADA	adenosine deaminase(ADA)	Homo sapiens
72	ME1	malic enzyme 1(ME1)	Homo sapiens
73	ME2	malic enzyme 2(ME2)	Homo sapiens
74	ADCY1	adenylate cyclase 1(ADCY1)	Homo sapiens
75	ADCY2	adenylate cyclase 2(ADCY2)	Homo sapiens
76	F10	coagulation factor X(F10)	Homo sapiens
77	ADCY5	adenylate cyclase 5(ADCY5)	Homo sapiens
78	RXRA	retinoid X receptor alpha(RXRA)	Homo sapiens
79	RXRB	retinoid X receptor beta(RXRB)	Homo sapiens
80	RXRG	retinoid X receptor gamma(RXR	Homo sapiens
81	FAAH	fatty acid amide hydrolase(FAAH	Homo sapiens
82	FABP6	fatty acid binding protein 6(FABP	Homo sapiens
83	ADH1A	alcohol dehydrogenase 1A (class I	Homo sapiens
84	ADH1B	alcohol dehydrogenase 1B (class I	Homo sapiens
85	ADH1C	alcohol dehydrogenase 1C (class I	Homo sapiens
86	GNRHR2	gonadotropin releasing hormone r	Homo sapiens
87	ADH4	alcohol dehydrogenase 4 (class II)	Homo sapiens
88	ADH5	alcohol dehydrogenase 5 (class III	Homo sapiens
89	ADH7	alcohol dehydrogenase 7 (class IV	Homo sapiens
90	ACSL1	acyl-CoA synthetase long chain fa	Homo sapiens
91	ADK	adenosine kinase(ADK)	Homo sapiens
92	ACSL3	acyl-CoA synthetase long chain fa	Homo sapiens
93	ACSL4	acyl-CoA synthetase long chain fa	Homo sapiens
94	ADORA1	adenosine A1 receptor(ADORA1)	Homo sapiens
95	ADORA2A	adenosine A2a receptor(ADORA2	Homo sapiens
96	ADORA2B	adenosine A2b receptor(ADORA2	Homo sapiens
97	PTK2B	protein tyrosine kinase 2 beta(PT	Homo sapiens
98	ADORA3	adenosine A3 receptor(ADORA3)	Homo sapiens
99	TUBB4A	tubulin beta 4A class IVa(TUBB4	Homo sapiens
100	S100P	S100 calcium binding protein P(S	Homo sapiens
101	PARP1	poly(ADP-ribose) polymerase 1(P	Homo sapiens
102	FARSA	phenylalanyl-tRNA synthetase su	Homo sapiens
103	ADRA1D	adrenoceptor alpha 1D(ADRA1D)	Homo sapiens
104	ADRA1B	adrenoceptor alpha 1B(ADRA1B)	Homo sapiens
105	ADRA1A	adrenoceptor alpha 1A(ADRA1A)	Homo sapiens
106	MGAT1	alpha-1,3-mannosyl-glycoprotein	Homo sapiens
107	ADRA2A	adrenoceptor alpha 2A(ADRA2A	Homo sapiens
108	ADRA2B	adrenoceptor alpha 2B(ADRA2B)	Homo sapiens
109	ADRA2C	adrenoceptor alpha 2C(ADRA2C)	Homo sapiens
110	ADRB1	adrenoceptor beta 1(ADRB1)	Homo sapiens
111	ADRB2	adrenoceptor beta 2(ADRB2)	Homo sapiens
112	FBP1	fructose-bisphosphatase 1(FBP1)	Homo sapiens
113	ADRB3	adrenoceptor beta 3(ADRB3)	Homo sapiens
114	MAPK12	mitogen-activated protein kinase 1	Homo sapiens
115	AARS2	alanyl-tRNA synthetase 2, mitoch	Homo sapiens
116	MSMO1	methylsterol monooxygenase 1(M	Homo sapiens
117	CDIPT	CDP-diacylglycerol--inositol 3-ph	Homo sapiens
118	FDXR	ferredoxin reductase(FDXR)	Homo sapiens
119	AGTR1	angiotensin II receptor type 1(AG	Homo sapiens
120	MIF	macrophage migration inhibitory	Homo sapiens
121	FECH	ferrochelatase(FECH)	Homo sapiens
122	SCN5A	sodium voltage-gated channel alp	Homo sapiens
123	MIP	major intrinsic protein of lens fibe	Homo sapiens
124	AGXT	alanine--glyoxylate and serine--py	Homo sapiens
125	NR0B1	nuclear receptor subfamily 0 grou	Homo sapiens
126	AHCY	adenosylhomocysteinase(AHCY)	Homo sapiens
127	SCN10A	sodium voltage-gated channel alp	Homo sapiens
128	FGA	fibrinogen alpha chain(FGA)	Homo sapiens
129	FGB	fibrinogen beta chain(FGB)	Homo sapiens
130	AHR	aryl hydrocarbon receptor(AHR)	Homo sapiens
131	FGF1	fibroblast growth factor 1(FGF1)	Homo sapiens
132	FGF2	fibroblast growth factor 2(FGF2)	Homo sapiens
133	KRTAP5-3	keratin associated protein 5-3(KR	Homo sapiens
134	FGF4	fibroblast growth factor 4(FGF4)	Homo sapiens
135	AK1	adenylate kinase 1(AK1)	Homo sapiens
136	AK2	adenylate kinase 2(AK2)	Homo sapiens
137	AKT1	AKT serine/threonine kinase 1(A	Homo sapiens
138	L3HYPDH	trans-L-3-hydroxyproline dehydra	Homo sapiens
139	CCL5	C-C motif chemokine ligand 5(CC	Homo sapiens
140	NR3C2	nuclear receptor subfamily 3 grou	Homo sapiens
141	ALAD	aminolevulinate dehydratase(ALA	Homo sapiens
142	ALB	albumin(ALB)	Homo sapiens
143	ECI2	enoyl-CoA delta isomerase 2(ECI	Homo sapiens
144	TAGLN2	transgelin 2(TAGLN2)	Homo sapiens
145	FGFR2	fibroblast growth factor receptor 2	Homo sapiens
146	ALDH1A1	aldehyde dehydrogenase 1 family	Homo sapiens
147	ALDH2	aldehyde dehydrogenase 2 family	Homo sapiens
148	FGG	fibrinogen gamma chain(FGG)	Homo sapiens
149	ALDH3A1	aldehyde dehydrogenase 3 family	Homo sapiens
150	ALDH1B1	aldehyde dehydrogenase 1 family	Homo sapiens
151	ALDH1A3	aldehyde dehydrogenase 1 family	Homo sapiens
152	MMP8	matrix metallopeptidase 8(MMP8	Homo sapiens
153	ALDH3B1	aldehyde dehydrogenase 3 family	Homo sapiens
154	MMP9	matrix metallopeptidase 9(MMP9	Homo sapiens
155	PRKAG2	protein kinase AMP-activated non	Homo sapiens
156	ALDH3B2	aldehyde dehydrogenase 3 family	Homo sapiens
157	ALDH9A1	aldehyde dehydrogenase 9 family	Homo sapiens
158	FHIT	fragile histidine triad diadenosine	Homo sapiens
159	ALDH3A2	aldehyde dehydrogenase 3 family	Homo sapiens
160	PPIH	peptidylprolyl isomerase H(PPIH)	Homo sapiens
161	POLK	DNA polymerase kappa(POLK)	Homo sapiens
162	ALDOA	aldolase, fructose-bisphosphate A(	Homo sapiens
163	AKR1B1	aldo-keto reductase family 1 mem	Homo sapiens
164	BBOX1	gamma-butyrobetaine hydroxylase	Homo sapiens
165	FKBP1A	FKBP prolyl isomerase 1A(FKBP	Homo sapiens
166	ALDH6A1	aldehyde dehydrogenase 6 family	Homo sapiens
167	SEC14L4	SEC14 like lipid binding 4(SEC14	Homo sapiens
168	ALK	ALK receptor tyrosine kinase(AL	Homo sapiens
169	ALOX5	arachidonate 5-lipoxygenase(ALO	Homo sapiens
170	SOAT2	sterol O-acyltransferase 2(SOAT2	Homo sapiens
171	SDHA	succinate dehydrogenase complex	Homo sapiens
172	SDHB	succinate dehydrogenase complex	Homo sapiens
173	ALOX15	arachidonate 15-lipoxygenase(AL	Homo sapiens
174	IGKV2-30	immunoglobulin kappa variable 2-	Homo sapiens
175	SDHC	succinate dehydrogenase complex	Homo sapiens
176	SDHD	succinate dehydrogenase complex	Homo sapiens
177	LCMT1	leucine carboxyl methyltransferas	Homo sapiens
178	NCOA2	nuclear receptor coactivator 2(NC	Homo sapiens
179	AMD1	adenosylmethionine decarboxylas	Homo sapiens
180	ABCC1	ATP binding cassette subfamily C	Homo sapiens
181	FLNA	filamin A(FLNA)	Homo sapiens
182	AMHR2	anti-Mullerian hormone receptor t	Homo sapiens
183	SMARCA5	SWI/SNF related, matrix associate	Homo sapiens
184	AMT	aminomethyltransferase(AMT)	Homo sapiens
185	AMY1A	amylase alpha 1A(AMY1A)	Homo sapiens
186	SFPQ	splicing factor proline and glutami	Homo sapiens
187	HSD17B7	hydroxysteroid 17-beta dehydroge	Homo sapiens
188	AMY2A	amylase alpha 2A(AMY2A)	Homo sapiens
189	AMY2B	amylase alpha 2B(AMY2B)	Homo sapiens
190	SRSF4	serine and arginine rich splicing fa	Homo sapiens
191	DCPS	decapping enzyme, scavenger(DC	Homo sapiens
192	CHRNA9	cholinergic receptor nicotinic alph	Homo sapiens
193	ANPEP	alanyl aminopeptidase, membrane	Homo sapiens
194	SLC25A4	solute carrier family 25 member 4(	Homo sapiens
195	RTCB	RNA 2',3'-cyclic phosphate and 5'-	Homo sapiens
196	P3H3	prolyl 3-hydroxylase 3(P3H3)	Homo sapiens
197	SFTPD	surfactant protein D(SFTPD)	Homo sapiens
198	FOLR1	folate receptor alpha(FOLR1)	Homo sapiens
199	ANXA1	annexin A1(ANXA1)	Homo sapiens
200	FOLR2	folate receptor beta(FOLR2)	Homo sapiens
201	ANXA2	annexin A2(ANXA2)	Homo sapiens
202	FOLR3	folate receptor gamma(FOLR3)	Homo sapiens
203	DHRS4L2	dehydrogenase/reductase 4 like 2(	Homo sapiens
204	ANXA3	annexin A3(ANXA3)	Homo sapiens
205	ANXA4	annexin A4(ANXA4)	Homo sapiens
206	FPGS	folylpolyglutamate synthase(FPG	Homo sapiens
207	ANXA5	annexin A5(ANXA5)	Homo sapiens
208	APAF1	apoptotic peptidase activating fact	Homo sapiens
209	SHBG	sex hormone binding globulin(SH	Homo sapiens
210	IYD	iodotyrosine deiodinase(IYD)	Homo sapiens
211	TUBB	tubulin beta class I(TUBB)	Homo sapiens
212	LDHAL6B	lactate dehydrogenase A like 6B(	Homo sapiens
213	APCS	amyloid P component, serum(APC	Homo sapiens
214	SHMT1	serine hydroxymethyltransferase 1	Homo sapiens
215	SHMT2	serine hydroxymethyltransferase 2	Homo sapiens
216	SI	sucrase-isomaltase(SI)	Homo sapiens
217	BIRC5	baculoviral IAP repeat containing	Homo sapiens
218	TARS2	threonyl-tRNA synthetase 2, mito	Homo sapiens
219	KLK3	kallikrein related peptidase 3(KL	Homo sapiens
220	SKP1	S-phase kinase associated protein	Homo sapiens
221	AQP1	aquaporin 1 (Colton blood group)(	Homo sapiens
222	SLC1A4	solute carrier family 1 member 4(	Homo sapiens
223	AR	androgen receptor(AR)	Homo sapiens
224	ABCC6	ATP binding cassette subfamily C	Homo sapiens
225	AK8	adenylate kinase 8(AK8)	Homo sapiens
226	ARAF	A-Raf proto-oncogene, serine/thre	Homo sapiens
227	PDXK	pyridoxal kinase(PDXK)	Homo sapiens
228	ARF1	ADP ribosylation factor 1(ARF1)	Homo sapiens
229	KHSRP	KH-type splicing regulatory prote	Homo sapiens
230	ARF4	ADP ribosylation factor 4(ARF4)	Homo sapiens
231	SLC5A2	solute carrier family 5 member 2(	Homo sapiens
232	MTFMT	mitochondrial methionyl-tRNA fo	Homo sapiens
233	ARF6	ADP ribosylation factor 6(ARF6)	Homo sapiens
234	NCOA5	nuclear receptor coactivator 5(NC	Homo sapiens
235	ARG1	arginase 1(ARG1)	Homo sapiens
236	PRKAG3	protein kinase AMP-activated non	Homo sapiens
237	ARG2	arginase 2(ARG2)	Homo sapiens
238	SEC14L3	SEC14 like lipid binding 3(SEC14	Homo sapiens
239	SLC6A2	solute carrier family 6 member 2(	Homo sapiens
240	KANSL3	KAT8 regulatory NSL complex su	Homo sapiens
241	SLC6A3	solute carrier family 6 member 3(	Homo sapiens
242	RHOA	ras homolog family member A(R	Homo sapiens
243	SLC6A4	solute carrier family 6 member 4(	Homo sapiens
244	SLC6A7	solute carrier family 6 member 7(	Homo sapiens
245	RND3	Rho family GTPase 3(RND3)	Homo sapiens
246	SLC7A1	solute carrier family 7 member 1(	Homo sapiens
247	SLC7A2	solute carrier family 7 member 2(	Homo sapiens
248	ARL1	ADP ribosylation factor like GTP	Homo sapiens
249	SLC7A4	solute carrier family 7 member 4(	Homo sapiens
250	SLC8A1	solute carrier family 8 member A1	Homo sapiens
251	IARS2	isoleucyl-tRNA synthetase 2, mito	Homo sapiens
252	ARL3	ADP ribosylation factor like GTP	Homo sapiens
253	ACAD8	acyl-CoA dehydrogenase family m	Homo sapiens
254	MTAP	methylthioadenosine phosphoryla	Homo sapiens
255	DPH5	diphthamide biosynthesis 5(DPH5	Homo sapiens
256	LCTL	lactase like(LCTL)	Homo sapiens
257	MT-CO1	mitochondrially encoded cytochro	Homo sapiens
258	MT-CO2	mitochondrially encoded cytochro	Homo sapiens
259	SLC13A1	solute carrier family 13 member 1	Homo sapiens
260	MT-CO3	mitochondrially encoded cytochro	Homo sapiens
261	SLC15A1	solute carrier family 15 member 1	Homo sapiens
262	PTGR1	prostaglandin reductase 1(PTGR1	Homo sapiens
263	MT-CYB	mitochondrially encoded cytochro	Homo sapiens
264	MTHFD1	methylenetetrahydrofolate dehydr	Homo sapiens
265	SLC18A1	solute carrier family 18 member A	Homo sapiens
266	FARS2	phenylalanyl-tRNA synthetase 2,	Homo sapiens
267	MTOR	mechanistic target of rapamycin k	Homo sapiens
268	SLC18A2	solute carrier family 18 member A	Homo sapiens
269	MTHFR	methylenetetrahydrofolate reducta	Homo sapiens
270	ASL	argininosuccinate lyase(ASL)	Homo sapiens
271	HSD17B6	hydroxysteroid 17-beta dehydroge	Homo sapiens
272	MT-ND1	mitochondrially encoded NADH d	Homo sapiens
273	MT-ND2	mitochondrially encoded NADH d	Homo sapiens
274	ASNS	asparagine synthetase (glutamine-	Homo sapiens
275	MT-ND3	mitochondrially encoded NADH d	Homo sapiens
276	EBP	EBP cholestenol delta-isomerase(	Homo sapiens
277	MT-ND4	mitochondrially encoded NADH d	Homo sapiens
278	MT-ND4L	mitochondrially encoded NADH 4	Homo sapiens
279	MT-ND5	mitochondrially encoded NADH d	Homo sapiens
280	ASPH	aspartate beta-hydroxylase(ASPH	Homo sapiens
281	MT-ND6	mitochondrially encoded NADH d	Homo sapiens
282	ASS1	argininosuccinate synthase 1(ASS	Homo sapiens
283	AOC3	amine oxidase copper containing	Homo sapiens
284	NT5C2	5'-nucleotidase, cytosolic II(NT5C	Homo sapiens
285	MTTP	microsomal triglyceride transfer p	Homo sapiens
286	MTR	5-methyltetrahydrofolate-homocys	Homo sapiens
287	AKR1C3	aldo-keto reductase family 1 mem	Homo sapiens
288	ABCB11	ATP binding cassette subfamily B	Homo sapiens
289	NCOA1	nuclear receptor coactivator 1(NC	Homo sapiens
290	PDE5A	phosphodiesterase 5A(PDE5A)	Homo sapiens
291	SERPINC1	serpin family C member 1(SERPI	Homo sapiens
292	B3GAT1	beta-1,3-glucuronyltransferase 1(	Homo sapiens
293	FTL	ferritin light chain(FTL)	Homo sapiens
294	SMO	smoothened, frizzled class recepto	Homo sapiens
295	UQCRQ	ubiquinol-cytochrome c reductase	Homo sapiens
296	ALDH4A1	aldehyde dehydrogenase 4 family	Homo sapiens
297	SIGLEC1	sialic acid binding Ig like lectin 1(	Homo sapiens
298	TRAPPC3	trafficking protein particle comple	Homo sapiens
299	ATIC	5-aminoimidazole-4-carboxamide	Homo sapiens
300	EIF3F	eukaryotic translation initiation fa	Homo sapiens
301	ATOX1	antioxidant 1 copper chaperone(A	Homo sapiens
302	ATP1A1	ATPase Na+/K+ transporting sub	Homo sapiens
303	ATP1A2	ATPase Na+/K+ transporting sub	Homo sapiens
304	ATP1A3	ATPase Na+/K+ transporting sub	Homo sapiens
305	OAZ3	ornithine decarboxylase antizyme	Homo sapiens
306	BECN1	beclin 1(BECN1)	Homo sapiens
307	ATP2A1	ATPase sarcoplasmic/endoplasmi	Homo sapiens
308	SNRPD2	small nuclear ribonucleoprotein D	Homo sapiens
309	PDE7B	phosphodiesterase 7B(PDE7B)	Homo sapiens
310	G6PD	glucose-6-phosphate dehydrogena	Homo sapiens
311	GAA	alpha glucosidase(GAA)	Homo sapiens
312	ALDH7A1	aldehyde dehydrogenase 7 family	Homo sapiens
313	PADI3	peptidyl arginine deiminase 3(PA	Homo sapiens
314	SOAT1	sterol O-acyltransferase 1(SOAT1	Homo sapiens
315	CACNA2D3	calcium voltage-gated channel au	Homo sapiens
316	SOD1	superoxide dismutase 1(SOD1)	Homo sapiens
317	SOD2	superoxide dismutase 2(SOD2)	Homo sapiens
318	CYB5R1	cytochrome b5 reductase 1(CYB5	Homo sapiens
319	GABRA1	gamma-aminobutyric acid type A	Homo sapiens
320	MAP3K2	mitogen-activated protein kinase k	Homo sapiens
321	GABRA2	gamma-aminobutyric acid type A	Homo sapiens
322	GABRA3	gamma-aminobutyric acid type A	Homo sapiens
323	SORD	sorbitol dehydrogenase(SORD)	Homo sapiens
324	GABRA4	gamma-aminobutyric acid type A	Homo sapiens
325	GABRA5	gamma-aminobutyric acid type A	Homo sapiens
326	GABRA6	gamma-aminobutyric acid type A	Homo sapiens
327	GABRB1	gamma-aminobutyric acid type A	Homo sapiens
328	GABRB2	gamma-aminobutyric acid type A	Homo sapiens
329	GABRB3	gamma-aminobutyric acid type A	Homo sapiens
330	GABRD	gamma-aminobutyric acid type A	Homo sapiens
331	GABRE	gamma-aminobutyric acid type A	Homo sapiens
332	GABRG1	gamma-aminobutyric acid type A	Homo sapiens
333	GABRG2	gamma-aminobutyric acid type A	Homo sapiens
334	GABRG3	gamma-aminobutyric acid type A	Homo sapiens
335	GABRP	gamma-aminobutyric acid type A	Homo sapiens
336	GABRR1	gamma-aminobutyric acid type A	Homo sapiens
337	ATP6V1A	ATPase H+ transporting V1 subu	Homo sapiens
338	CMPK1	cytidine/uridine monophosphate k	Homo sapiens
339	UAP1	UDP-N-acetylglucosamine pyrop	Homo sapiens
340	MYH9	myosin heavy chain 9(MYH9)	Homo sapiens
341	GALE	UDP-galactose-4-epimerase(GAL	Homo sapiens
342	GALK1	galactokinase 1(GALK1)	Homo sapiens
343	LDHAL6A	lactate dehydrogenase A like 6A(	Homo sapiens
344	CMTR1	cap methyltransferase 1(CMTR1)	Homo sapiens
345	GAMT	guanidinoacetate N-methyltransfe	Homo sapiens
346	GANC	glucosidase alpha, neutral C(GAN	Homo sapiens
347	KIF1A	kinesin family member 1A(KIF1A	Homo sapiens
348	GAPDH	glyceraldehyde-3-phosphate dehy	Homo sapiens
349	SPR	sepiapterin reductase(SPR)	Homo sapiens
350	MTHFD2	methylenetetrahydrofolate dehydr	Homo sapiens
351	CYSLTR1	cysteinyl leukotriene receptor 1(C	Homo sapiens
352	RAB8B	RAB8B, member RAS oncogene	Homo sapiens
353	ACSS1	acyl-CoA synthetase short chain f	Homo sapiens
354	B2M	beta-2-microglobulin(B2M)	Homo sapiens
355	SQLE	squalene epoxidase(SQLE)	Homo sapiens
356	GART	phosphoribosylglycinamide formy	Homo sapiens
357	SRC	SRC proto-oncogene, non-recepto	Homo sapiens
358	UGT3A1	UDP glycosyltransferase family 3	Homo sapiens
359	SRD5A1	steroid 5 alpha-reductase 1(SRD5	Homo sapiens
360	SRD5A2	steroid 5 alpha-reductase 2(SRD5	Homo sapiens
361	BAG1	BAG cochaperone 1(BAG1)	Homo sapiens
362	RAB11A	RAB11A, member RAS oncogene	Homo sapiens
363	AKR1D1	aldo-keto reductase family 1 mem	Homo sapiens
364	OXCT2	3-oxoacid CoA-transferase 2(OX	Homo sapiens
365	GATM	glycine amidinotransferase(GAT	Homo sapiens
366	GBA	glucosylceramidase beta(GBA)	Homo sapiens
367	GABRQ	gamma-aminobutyric acid type A	Homo sapiens
368	SRP14	signal recognition particle 14(SR	Homo sapiens
369	BCAT1	branched chain amino acid transa	Homo sapiens
370	BCAT2	branched chain amino acid transa	Homo sapiens
371	SRPK2	SRSF protein kinase 2(SRPK2)	Homo sapiens
372	BCHE	butyrylcholinesterase(BCHE)	Homo sapiens
373	GCDH	glutaryl-CoA dehydrogenase(GC	Homo sapiens
374	NCF2	neutrophil cytosolic factor 2(NCF	Homo sapiens
375	NCF4	neutrophil cytosolic factor 4(NCF	Homo sapiens
376	BCL2	BCL2 apoptosis regulator(BCL2)	Homo sapiens
377	GCK	glucokinase(GCK)	Homo sapiens
378	BCL2L1	BCL2 like 1(BCL2L1)	Homo sapiens
379	NDUFA1	NADH:ubiquinone oxidoreductas	Homo sapiens
380	NDUFA2	NADH:ubiquinone oxidoreductas	Homo sapiens
381	ALDH1L1	aldehyde dehydrogenase 1 family	Homo sapiens
382	NDUFA3	NADH:ubiquinone oxidoreductas	Homo sapiens
383	FTCD	formimidoyltransferase cyclodea	Homo sapiens
384	NDUFA4	NDUFA4 mitochondrial complex	Homo sapiens
385	NDUFA5	NADH:ubiquinone oxidoreductas	Homo sapiens
386	NDUFA6	NADH:ubiquinone oxidoreductas	Homo sapiens
387	NDUFA7	NADH:ubiquinone oxidoreductas	Homo sapiens
388	ACSS2	acyl-CoA synthetase short chain f	Homo sapiens
389	PDE10A	phosphodiesterase 10A(PDE10A)	Homo sapiens
390	NDUFA8	NADH:ubiquinone oxidoreductas	Homo sapiens
391	SSTR1	somatostatin receptor 1(SSTR1)	Homo sapiens
392	GNMT	glycine N-methyltransferase(GN	Homo sapiens
393	SSTR2	somatostatin receptor 2(SSTR2)	Homo sapiens
394	NDUFA9	NADH:ubiquinone oxidoreductas	Homo sapiens
395	SUCLG2	succinate-CoA ligase GDP-formin	Homo sapiens
396	SSTR3	somatostatin receptor 3(SSTR3)	Homo sapiens
397	NDUFA10	NADH:ubiquinone oxidoreductas	Homo sapiens
398	SUCLG1	succinate-CoA ligase GDP/ADP-f	Homo sapiens
399	HCN2	hyperpolarization activated cyclic	Homo sapiens
400	NDUFAB1	NADH:ubiquinone oxidoreductas	Homo sapiens
401	SUCLA2	succinate-CoA ligase ADP-formin	Homo sapiens
402	CMAS	cytidine monophosphate N-acetyl	Homo sapiens
403	SSTR5	somatostatin receptor 5(SSTR5)	Homo sapiens
404	NDUFB1	NADH:ubiquinone oxidoreductas	Homo sapiens
405	NDUFB2	NADH:ubiquinone oxidoreductas	Homo sapiens
406	BCR	BCR activator of RhoGEF and GT	Homo sapiens
407	NDUFB3	NADH:ubiquinone oxidoreductas	Homo sapiens
408	NDUFB4	NADH:ubiquinone oxidoreductas	Homo sapiens
409	NDUFB5	NADH:ubiquinone oxidoreductas	Homo sapiens
410	RUVBL2	RuvB like AAA ATPase 2(RUVB	Homo sapiens
411	NDUFB6	NADH:ubiquinone oxidoreductas	Homo sapiens
412	PGRMC1	progesterone receptor membrane c	Homo sapiens
413	NDUFB7	NADH:ubiquinone oxidoreductas	Homo sapiens
414	NDUFB8	NADH:ubiquinone oxidoreductas	Homo sapiens
415	NDUFB9	NADH:ubiquinone oxidoreductas	Homo sapiens
416	NDUFB10	NADH:ubiquinone oxidoreductas	Homo sapiens
417	NDUFC1	NADH:ubiquinone oxidoreductas	Homo sapiens
418	BDH1	3-hydroxybutyrate dehydrogenase	Homo sapiens
419	NDUFC2	NADH:ubiquinone oxidoreductas	Homo sapiens
420	GFER	growth factor, augmenter of liver	Homo sapiens
421	NDUFS1	NADH:ubiquinone oxidoreductas	Homo sapiens
422	BDKRB2	bradykinin receptor B2(BDKRB2	Homo sapiens
423	NDUFS2	NADH:ubiquinone oxidoreductas	Homo sapiens
424	NDUFS3	NADH:ubiquinone oxidoreductas	Homo sapiens
425	NDUFV1	NADH:ubiquinone oxidoreductas	Homo sapiens
426	NDUFS4	NADH:ubiquinone oxidoreductas	Homo sapiens
427	GGCX	gamma-glutamyl carboxylase(GG	Homo sapiens
428	NDUFS5	NADH:ubiquinone oxidoreductas	Homo sapiens
429	NDUFS6	NADH:ubiquinone oxidoreductas	Homo sapiens
430	FFAR4	free fatty acid receptor 4(FFAR4)	Homo sapiens

431	NDUFS8	NADH:ubiquinone oxidoreductase	Homo sapiens
432	ME3	malic enzyme 3(ME3)	Homo sapiens
433	NDUFV2	NADH:ubiquinone oxidoreductase	Homo sapiens
434	B4GALT1	beta-1,4-galactosyltransferase 1(B	Homo sapiens
435	NDUFV3	NADH:ubiquinone oxidoreductase	Homo sapiens
436	SULT1E1	sulfotransferase family 1E membe	Homo sapiens
437	ELOVL4	ELOVL fatty acid elongase 4(EL	Homo sapiens
438	BLVRA	biliverdin reductase A(BLVRA)	Homo sapiens
439	BLVRB	biliverdin reductase B(BLVRB)	Homo sapiens
440	METAP1	methionyl aminopeptidase 1(MET	Homo sapiens
441	TUBB6	tubulin beta 6 class V(TUBB6)	Homo sapiens
442	SULT1B1	sulfotransferase family 1B membe	Homo sapiens
443	ALDH1A2	aldehyde dehydrogenase 1 family	Homo sapiens
444	NR1I2	nuclear receptor subfamily 1 grou	Homo sapiens
445	GANAB	glucosidase II alpha subunit(GAN	Homo sapiens
446	BPHL	biphenyl hydrolase like(BPHL)	Homo sapiens
447	NDUFA12	NADH:ubiquinone oxidoreductase	Homo sapiens
448	SULT1A1	sulfotransferase family 1A membe	Homo sapiens
449	PAPOLA	poly(A) polymerase alpha(PAPOL	Homo sapiens
450	SULT2B1	sulfotransferase family 2B membe	Homo sapiens
451	SULT2A1	sulfotransferase family 2A membe	Homo sapiens
452	BST1	bone marrow stromal cell antigen	Homo sapiens
453	P3H1	prolyl 3-hydroxylase 1(P3H1)	Homo sapiens
454	ABCC8	ATP binding cassette subfamily C	Homo sapiens
455	NAE1	NEDD8 activating enzyme E1 sub	Homo sapiens
456	GLO1	glyoxalase I(GLO1)	Homo sapiens
457	GLRA1	glycine receptor alpha 1(GLRA1)	Homo sapiens
458	AADACL2	arylacetamide deacetylase like 2(	Homo sapiens
459	GLRA2	glycine receptor alpha 2(GLRA2)	Homo sapiens
460	NFKB1	nuclear factor kappa B subunit 1(	Homo sapiens
461	NFKB2	nuclear factor kappa B subunit 2(	Homo sapiens
462	NFKBIA	NFKB inhibitor alpha(NFKBIA)	Homo sapiens
463	GLUD1	glutamate dehydrogenase 1(GLUD	Homo sapiens
464	GLUD2	glutamate dehydrogenase 2(GLUD	Homo sapiens
465	AFG3L2	AFG3 like matrix AAA peptidase	Homo sapiens
466	SYK	spleen associated tyrosine kinase(	Homo sapiens
467	SYN1	synapsin I(SYN1)	Homo sapiens
468	GM2A	ganglioside GM2 activator(GM2A	Homo sapiens
469	ACCS	1-aminocyclopropane-1-carboxyla	Homo sapiens
470	GMDS	GDP-mannose 4,6-dehydratase(G	Homo sapiens
471	GMPR	guanosine monophosphate reducta	Homo sapiens
472	CACNA1I	calcium voltage-gated channel sub	Homo sapiens
473	TRPV3	transient receptor potential cation	Homo sapiens
474	CACNA1H	calcium voltage-gated channel sub	Homo sapiens
475	CACNA1G	calcium voltage-gated channel sub	Homo sapiens
476	GNAI1	G protein subunit alpha i1(GNAI1	Homo sapiens
477	GNAS	GNAS complex locus(GNAS)	Homo sapiens
478	GNAT1	G protein subunit alpha transducin	Homo sapiens
479	TAGLN	transgelin(TAGLN)	Homo sapiens
480	C8G	complement C8 gamma chain(C8	Homo sapiens
481	NME1	NME/NM23 nucleoside diphosph	Homo sapiens
482	NME2	NME/NM23 nucleoside diphosph	Homo sapiens
483	GPT2	glutamic--pyruvic transaminase 2(	Homo sapiens
484	NQO2	N-ribosyldihydronicotinamide:qui	Homo sapiens
485	NMT1	N-myristoyltransferase 1(NMT1)	Homo sapiens
486	NOS1	nitric oxide synthase 1(NOS1)	Homo sapiens
487	NOS2	nitric oxide synthase 2(NOS2)	Homo sapiens
488	METAP2	methionyl aminopeptidase 2(MET	Homo sapiens
489	KYNU	kynureninase(KYNU)	Homo sapiens
490	GNRHR	gonadotropin releasing hormone r	Homo sapiens
491	NOS3	nitric oxide synthase 3(NOS3)	Homo sapiens
492	TAT	tyrosine aminotransferase(TAT)	Homo sapiens
493	NPY	neuropeptide Y(NPY)	Homo sapiens
494	CA1	carbonic anhydrase 1(CA1)	Homo sapiens
495	CA2	carbonic anhydrase 2(CA2)	Homo sapiens
496	CA3	carbonic anhydrase 3(CA3)	Homo sapiens
497	CA4	carbonic anhydrase 4(CA4)	Homo sapiens
498	CA5A	carbonic anhydrase 5A(CA5A)	Homo sapiens
499	KIF2C	kinesin family member 2C(KIF2C	Homo sapiens
500	PNP	purine nucleoside phosphorylase(	Homo sapiens
501	CA6	carbonic anhydrase 6(CA6)	Homo sapiens
502	CA7	carbonic anhydrase 7(CA7)	Homo sapiens
503	CA9	carbonic anhydrase 9(CA9)	Homo sapiens
504	GPD1	glycerol-3-phosphate dehydrogena	Homo sapiens
505	TBXA2R	thromboxane A2 receptor(TBXA2	Homo sapiens
506	CA12	carbonic anhydrase 12(CA12)	Homo sapiens
507	GPI	glucose-6-phosphate isomerase(G	Homo sapiens
508	CACNA1A	calcium voltage-gated channel sub	Homo sapiens
509	NPM1	nucleophosmin 1(NPM1)	Homo sapiens
510	CACNA1B	calcium voltage-gated channel sub	Homo sapiens
511	CACNA1C	calcium voltage-gated channel sub	Homo sapiens
512	CACNA1D	calcium voltage-gated channel sub	Homo sapiens
513	CACNA1F	calcium voltage-gated channel sub	Homo sapiens
514	CACNA1S	calcium voltage-gated channel sub	Homo sapiens
515	MGAM	maltase-glucoamylase(MGAM)	Homo sapiens
516	CHRNA6	cholinergic receptor nicotinic alph	Homo sapiens
517	CACNA2D1	calcium voltage-gated channel aux	Homo sapiens
518	P4HA2	prolyl 4-hydroxylase subunit alph	Homo sapiens
519	CACNB1	calcium voltage-gated channel aux	Homo sapiens
520	CACNB2	calcium voltage-gated channel aux	Homo sapiens
521	NPPB	natriuretic peptide B(NPPB)	Homo sapiens
522	CACNB3	calcium voltage-gated channel aux	Homo sapiens
523	CACNB4	calcium voltage-gated channel aux	Homo sapiens
524	CACNG1	calcium voltage-gated channel aux	Homo sapiens
525	PLOD3	procollagen-lysine,2-oxoglutarate	Homo sapiens
526	CALCA	calcitonin related polypeptide alp	Homo sapiens
527	TRPA1	transient receptor potential cation	Homo sapiens
528	CALM1	calmodulin 1(CALM1)	Homo sapiens
529	GPER1	G protein-coupled estrogen recept	Homo sapiens
530	AZIN2	antizyme inhibitor 2(AZIN2)	Homo sapiens
531	ALKBH2	alkB homolog 2, alpha-ketoglutar	Homo sapiens
532	FFAR1	free fatty acid receptor 1(FFAR1)	Homo sapiens
533	CAMK2G	calcium/calmodulin dependent pr	Homo sapiens
534	GPT	glutamic--pyruvic transaminase(G	Homo sapiens
535	CAST	calpastatin(CAST)	Homo sapiens
536	CASP1	caspase 1(CASP1)	Homo sapiens
537	CASP3	caspase 3(CASP3)	Homo sapiens
538	GRIA2	glutamate ionotropic receptor AM	Homo sapiens
539	OAT	ornithine aminotransferase(OAT)	Homo sapiens
540	OAZ1	ornithine decarboxylase antizyme	Homo sapiens
541	OAZ2	ornithine decarboxylase antizyme	Homo sapiens
542	RPL14	ribosomal protein L14(RPL14)	Homo sapiens
543	GRIN1	glutamate ionotropic receptor NM	Homo sapiens
544	GRIN2A	glutamate ionotropic receptor NM	Homo sapiens
545	GRIN2B	glutamate ionotropic receptor NM	Homo sapiens
546	GRIN2C	glutamate ionotropic receptor NM	Homo sapiens
547	GRIN2D	glutamate ionotropic receptor NM	Homo sapiens
548	POLE3	DNA polymerase epsilon 3, acces	Homo sapiens
549	NR3C1	nuclear receptor subfamily 3 grou	Homo sapiens
550	NFS1	NFS1 cysteine desulfurase(NFS1)	Homo sapiens
551	SLC13A2	solute carrier family 13 member 2	Homo sapiens
552	LARS2	leucyl-tRNA synthetase 2, mitoch	Homo sapiens
553	PAPSS1	3'-phosphoadenosine 5'-phosphos	Homo sapiens
554	TERT	telomerase reverse transcriptase(T	Homo sapiens
555	OGDH	oxoglutarate dehydrogenase(OGD	Homo sapiens
556	HIBADH	3-hydroxyisobutyrate dehydrogen	Homo sapiens
557	CBR1	carbonyl reductase 1(CBR1)	Homo sapiens
558	CBS	cystathionine beta-synthase(CBS)	Homo sapiens
559	SIRT5	sirtuin 5(SIRT5)	Homo sapiens
560	NR2F1	nuclear receptor subfamily 2 grou	Homo sapiens
561	SIRT3	sirtuin 3(SIRT3)	Homo sapiens
562	GSK3B	glycogen synthase kinase 3 beta(	Homo sapiens
563	GSPT1	G1 to S phase transition 1(GSPT1	Homo sapiens
564	GSR	glutathione-disulfide reductase(G	Homo sapiens
565	GSS	glutathione synthetase(GSS)	Homo sapiens
566	OPRD1	opioid receptor delta 1(OPRD1)	Homo sapiens
567	GSTA1	glutathione S-transferase alpha 1(	Homo sapiens
568	OPRK1	opioid receptor kappa 1(OPRK1)	Homo sapiens
569	OPRL1	opioid related nociceptin receptor	Homo sapiens
570	GSTA3	glutathione S-transferase alpha 3(	Homo sapiens
571	OPRM1	opioid receptor mu 1(OPRM1)	Homo sapiens
572	GSTM1	glutathione S-transferase mu 1(G	Homo sapiens
573	SLC7A8	solute carrier family 7 member 8(	Homo sapiens
574	GSTP1	glutathione S-transferase pi 1(GS	Homo sapiens
575	TGM2	transglutaminase 2(TGM2)	Homo sapiens
576	ORM1	orosomucoid 1(ORM1)	Homo sapiens
577	TGM3	transglutaminase 3(TGM3)	Homo sapiens
578	ORM2	orosomucoid 2(ORM2)	Homo sapiens
579	TH	tyrosine hydroxylase(TH)	Homo sapiens
580	OTC	ornithine transcarbamylase(OTC)	Homo sapiens
581	LSM6	LSM6 homolog, U6 small nuclear	Homo sapiens
582	SLC7A3	solute carrier family 7 member 3(	Homo sapiens
583	SF3B3	splicing factor 3b subunit 3(SF3B	Homo sapiens
584	THRA	thyroid hormone receptor alpha(T	Homo sapiens
585	OXCT1	3-oxoacid CoA-transferase 1(OX	Homo sapiens
586	THRB	thyroid hormone receptor beta(TH	Homo sapiens
587	GUCA1A	guanylate cyclase activator 1A(G	Homo sapiens
588	P4HA1	prolyl 4-hydroxylase subunit alph	Homo sapiens
589	P4HB	prolyl 4-hydroxylase subunit beta(	Homo sapiens
590	AIFM1	apoptosis inducing factor mitocho	Homo sapiens
591	GUK1	guanylate kinase 1(GUK1)	Homo sapiens
592	TK2	thymidine kinase 2(TK2)	Homo sapiens
593	GYG1	glycogenin 1(GYG1)	Homo sapiens
594	FURIN	furin, paired basic amino acid clea	Homo sapiens
595	PAEP	progestagen associated endometri	Homo sapiens
596	TLR4	toll like receptor 4(TLR4)	Homo sapiens
597	PRDX1	peroxiredoxin 1(PRDX1)	Homo sapiens
598	CYCS	cytochrome c, somatic(CYCS)	Homo sapiens
599	PAH	phenylalanine hydroxylase(PAH)	Homo sapiens
600	TAS1R2	taste 1 receptor member 2(TAS1R	Homo sapiens
601	PAM	peptidylglycine alpha-amidating m	Homo sapiens
602	IRGC	immunity related GTPase cinema(	Homo sapiens
603	CDA	cytidine deaminase(CDA)	Homo sapiens
604	PADI6	peptidyl arginine deiminase 6(PA	Homo sapiens
605	HSD17B10	hydroxysteroid 17-beta dehydroge	Homo sapiens
606	TNF	tumor necrosis factor(TNF)	Homo sapiens
607	HADHA	hydroxyacyl-CoA dehydrogenase	Homo sapiens
608	SETD7	SET domain containing 7, histone	Homo sapiens
609	RPL35	ribosomal protein L35(RPL35)	Homo sapiens
610	HADH	hydroxyacyl-CoA dehydrogenase(	Homo sapiens
611	HBA1	hemoglobin subunit alpha 1(HBA	Homo sapiens
612	RPL13A	ribosomal protein L13a(RPL13A)	Homo sapiens
613	HBB	hemoglobin subunit beta(HBB)	Homo sapiens
614	PCBD1	pterin-4 alpha-carbinolamine dehy	Homo sapiens
615	CDC42	cell division cycle 42(CDC42)	Homo sapiens
616	CA5B	carbonic anhydrase 5B(CA5B)	Homo sapiens
617	PADI2	peptidyl arginine deiminase 2(PA	Homo sapiens
618	PCCB	propionyl-CoA carboxylase subun	Homo sapiens
619	NNT	nicotinamide nucleotide transhydr	Homo sapiens
620	TOP1	DNA topoisomerase I(TOP1)	Homo sapiens
621	HCK	HCK proto-oncogene, Src family t	Homo sapiens
622	TOP2A	DNA topoisomerase II alpha(TOP	Homo sapiens
623	PCK1	phosphoenolpyruvate carboxykina	Homo sapiens
624	TOP2B	DNA topoisomerase II beta(TOP2	Homo sapiens
625	PTGDR2	prostaglandin D2 receptor 2(PTG	Homo sapiens
626	TP53	tumor protein p53(TP53)	Homo sapiens
627	SEC14L2	SEC14 like lipid binding 2(SEC14	Homo sapiens
628	MAN1B1	mannosidase alpha class 1B mem	Homo sapiens
629	SLC6A14	solute carrier family 6 member 14	Homo sapiens
630	PCMT1	protein-L-isoaspartate (D-aspartat	Homo sapiens
631	PCNA	proliferating cell nuclear antigen(	Homo sapiens
632	CDK2	cyclin dependent kinase 2(CDK2)	Homo sapiens
633	HDAC2	histone deacetylase 2(HDAC2)	Homo sapiens
634	AURKB	aurora kinase B(AURKB)	Homo sapiens
635	CDK6	cyclin dependent kinase 6(CDK6)	Homo sapiens
636	TPH1	tryptophan hydroxylase 1(TPH1)	Homo sapiens
637	TPI1	triosephosphate isomerase 1(TPI1	Homo sapiens
638	TPM1	tropomyosin 1(TPM1)	Homo sapiens
639	VAPA	VAMP associated protein A(VAP	Homo sapiens
640	TPO	thyroid peroxidase(TPO)	Homo sapiens
641	NOLC1	nucleolar and coiled-body phosph	Homo sapiens
642	TPR	translocated promoter region, nucl	Homo sapiens
643	HGF	hepatocyte growth factor(HGF)	Homo sapiens
644	CDO1	cysteine dioxygenase type 1(CDO	Homo sapiens
645	DDAH2	dimethylarginine dimethylaminoh	Homo sapiens
646	PHYKPL	5-phosphohydroxy-L-lysine phosp	Homo sapiens
647	HSP90B1	heat shock protein 90 beta family	Homo sapiens
648	SCN11A	sodium voltage-gated channel alp	Homo sapiens
649	PADI4	peptidyl arginine deiminase 4(PA	Homo sapiens
650	PDE2A	phosphodiesterase 2A(PDE2A)	Homo sapiens
651	PDE3A	phosphodiesterase 3A(PDE3A)	Homo sapiens
652	HIF1A	hypoxia inducible factor 1 subunit	Homo sapiens
653	PDE4A	phosphodiesterase 4A(PDE4A)	Homo sapiens
654	PDE4B	phosphodiesterase 4B(PDE4B)	Homo sapiens
655	HINT1	histidine triad nucleotide binding	Homo sapiens
656	PDE4C	phosphodiesterase 4C(PDE4C)	Homo sapiens
657	DDAH1	dimethylarginine dimethylaminoh	Homo sapiens
658	PDE4D	phosphodiesterase 4D(PDE4D)	Homo sapiens
659	HK1	hexokinase 1(HK1)	Homo sapiens
660	CEBPB	CCAAT enhancer binding protein	Homo sapiens
661	PDE7A	phosphodiesterase 7A(PDE7A)	Homo sapiens
662	HLA-A	major histocompatibility complex,	Homo sapiens
663	CCT3	chaperonin containing TCP1 subu	Homo sapiens
664	CACNA2D2	calcium voltage-gated channel aux	Homo sapiens
665	PDHA1	pyruvate dehydrogenase E1 subun	Homo sapiens
666	PDHA2	pyruvate dehydrogenase E1 subun	Homo sapiens
667	CES1	carboxylesterase 1(CES1)	Homo sapiens
668	PDHB	pyruvate dehydrogenase E1 subun	Homo sapiens
669	STK17B	serine/threonine kinase 17b(STK1	Homo sapiens
670	ENPP1	ectonucleotide pyrophosphatase/p	Homo sapiens
671	CFL1	cofilin 1(CFL1)	Homo sapiens
672	CYTH2	cytohesin 2(CYTH2)	Homo sapiens
673	CFTR	CF transmembrane conductance r	Homo sapiens
674	CA14	carbonic anhydrase 14(CA14)	Homo sapiens
675	CHD1	chromodomain helicase DNA bin	Homo sapiens
676	HMGCR	3-hydroxy-3-methylglutaryl-CoA	Homo sapiens
677	AKR1C4	aldo-keto reductase family 1 mem	Homo sapiens
678	CHEK1	checkpoint kinase 1(CHEK1)	Homo sapiens
679	PFKFB1	6-phosphofructo-2-kinase/fructose	Homo sapiens
680	PFKFB4	6-phosphofructo-2-kinase/fructose	Homo sapiens
681	HMOX1	heme oxygenase 1(HMOX1)	Homo sapiens
682	HAO1	hydroxyacid oxidase 1(HAO1)	Homo sapiens
683	LY96	lymphocyte antigen 96(LY96)	Homo sapiens
684	HMOX2	heme oxygenase 2(HMOX2)	Homo sapiens
685	LTB4R2	leukotriene B4 receptor 2(LTB4R	Homo sapiens
686	CHIT1	chitinase 1(CHIT1)	Homo sapiens
687	PFN1	profilin 1(PFN1)	Homo sapiens
688	UQCR10	ubiquinol-cytochrome c reductase,	Homo sapiens
689	HNF4A	hepatocyte nuclear factor 4 alpha(	Homo sapiens
690	HNF4G	hepatocyte nuclear factor 4 gamm	Homo sapiens
691	PGAM1	phosphoglycerate mutase 1(PGAM	Homo sapiens
692	CHRM1	cholinergic receptor muscarinic 1(	Homo sapiens
693	HNMT	histamine N-methyltransferase(H	Homo sapiens
694	CHRM2	cholinergic receptor muscarinic 2(	Homo sapiens
695	PGD	phosphogluconate dehydrogenase(	Homo sapiens
696	CHRM3	cholinergic receptor muscarinic 3(	Homo sapiens
697	CHRM4	cholinergic receptor muscarinic 4(	Homo sapiens
698	TUBA4A	tubulin alpha 4a(TUBA4A)	Homo sapiens
699	CHRM5	cholinergic receptor muscarinic 5(	Homo sapiens
700	HNRNPA2B1	heterogeneous nuclear ribonucleo	Homo sapiens
701	CHRNA1	cholinergic receptor nicotinic alph	Homo sapiens
702	PGK1	phosphoglycerate kinase 1(PGK1)	Homo sapiens
703	CHRNA2	cholinergic receptor nicotinic alph	Homo sapiens
704	CHRNA3	cholinergic receptor nicotinic alph	Homo sapiens
705	HNRNPD	heterogeneous nuclear ribonucleo	Homo sapiens
706	CHRNA4	cholinergic receptor nicotinic alph	Homo sapiens
707	CHRNA5	cholinergic receptor nicotinic alph	Homo sapiens
708	CHRNA7	cholinergic receptor nicotinic alph	Homo sapiens
709	TUFM	Tu translation elongation factor, m	Homo sapiens
710	CHRNB1	cholinergic receptor nicotinic beta	Homo sapiens
711	PGM1	phosphoglucomutase 1(PGM1)	Homo sapiens
712	CHRNB2	cholinergic receptor nicotinic beta	Homo sapiens
713	CHRNB3	cholinergic receptor nicotinic beta	Homo sapiens
714	HNRNPK	heterogeneous nuclear ribonucleo	Homo sapiens
715	CHRNB4	cholinergic receptor nicotinic beta	Homo sapiens
716	CHRND	cholinergic receptor nicotinic delt	Homo sapiens
717	CHRNE	cholinergic receptor nicotinic epsi	Homo sapiens
718	PGR	progesterone receptor(PGR)	Homo sapiens
719	CHRNG	cholinergic receptor nicotinic gam	Homo sapiens
720	ABCB1	ATP binding cassette subfamily B	Homo sapiens
721	TXNRD1	thioredoxin reductase 1(TXNRD1	Homo sapiens
722	TUBB1	tubulin beta 1 class VI(TUBB1)	Homo sapiens
723	TYMS	thymidylate synthetase(TYMS)	Homo sapiens
724	TYR	tyrosinase(TYR)	Homo sapiens
725	RPL23	ribosomal protein L23(RPL23)	Homo sapiens
726	CKM	creatine kinase, M-type(CKM)	Homo sapiens
727	HOXA10	homeobox A10(HOXA10)	Homo sapiens
728	PHYH	phytanoyl-CoA 2-hydroxylase(PH	Homo sapiens
729	PPIG	peptidylprolyl isomerase G(PPIG)	Homo sapiens
730	SERPINA1	serpin family A member 1(SERPI	Homo sapiens
731	UBA1	ubiquitin like modifier activating	Homo sapiens
732	RAB9A	RAB9A, member RAS oncogene f	Homo sapiens
733	VKORC1	vitamin K epoxide reductase comp	Homo sapiens
734	CLCN2	chloride voltage-gated channel 2(	Homo sapiens
735	COX5A	cytochrome c oxidase subunit 5A(	Homo sapiens
736	CLIC1	chloride intracellular channel 1(C	Homo sapiens
737	PIK3CA	phosphatidylinositol-4,5-bisphosp	Homo sapiens
738	PIM1	Pim-1 proto-oncogene, serine/thre	Homo sapiens
739	PIK3CD	phosphatidylinositol-4,5-bisphosp	Homo sapiens
740	PIK3CG	phosphatidylinositol-4,5-bisphosp	Homo sapiens
741	PIK3R1	phosphoinositide-3-kinase regulat	Homo sapiens
742	HPGD	15-hydroxyprostaglandin dehydro	Homo sapiens
743	HPRT1	hypoxanthine phosphoribosyltrans	Homo sapiens
744	PIN1	peptidylprolyl cis/trans isomerase,	Homo sapiens
745	NMT2	N-myristoyltransferase 2(NMT2)	Homo sapiens
746	CLPS	colipase(CLPS)	Homo sapiens
747	UGCG	UDP-glucose ceramide glucosyltr	Homo sapiens
748	UGDH	UDP-glucose 6-dehydrogenase(U	Homo sapiens
749	HRAS	HRas proto-oncogene, GTPase(H	Homo sapiens
750	PKM	pyruvate kinase M1/2(PKM)	Homo sapiens
751	HRH1	histamine receptor H1(HRH1)	Homo sapiens
752	PLA2G1B	phospholipase A2 group IB(PLA2	Homo sapiens
753	FADS2	fatty acid desaturase 2(FADS2)	Homo sapiens
754	PLA2G2A	phospholipase A2 group IIA(PLA	Homo sapiens
755	PLA2G4A	phospholipase A2 group IVA(PL	Homo sapiens
756	HRH2	histamine receptor H2(HRH2)	Homo sapiens
757	UCK2	uridine-cytidine kinase 2(UCK2)	Homo sapiens
758	UMPS	uridine monophosphate synthetase	Homo sapiens
759	PRMT1	protein arginine methyltransferase	Homo sapiens
760	TRPM8	transient receptor potential cation	Homo sapiens
761	PLAT	plasminogen activator, tissue type	Homo sapiens
762	NR1H2	nuclear receptor subfamily 1 grou	Homo sapiens
763	HSD3B1	hydroxy-delta-5-steroid dehydrog	Homo sapiens
764	HSD3B2	hydroxy-delta-5-steroid dehydrog	Homo sapiens
765	UQCRB	ubiquinol-cytochrome c reductase	Homo sapiens
766	UQCRC1	ubiquinol-cytochrome c reductase	Homo sapiens
767	UQCRC2	ubiquinol-cytochrome c reductase	Homo sapiens
768	LTB4R	leukotriene B4 receptor(LTB4R)	Homo sapiens
769	UQCRFS1	ubiquinol-cytochrome c reductase,	Homo sapiens
770	HSD11B1	hydroxysteroid 11-beta dehydroge	Homo sapiens
771	HSD11B2	hydroxysteroid 11-beta dehydroge	Homo sapiens
772	UQCRH	ubiquinol-cytochrome c reductase	Homo sapiens
773	ABCC2	ATP binding cassette subfamily C	Homo sapiens
774	HSD17B1	hydroxysteroid 17-beta dehydroge	Homo sapiens
775	FTO	FTO alpha-ketoglutarate depender	Homo sapiens
776	HSD17B3	hydroxysteroid 17-beta dehydroge	Homo sapiens
777	HSD17B2	hydroxysteroid 17-beta dehydroge	Homo sapiens
778	HSD17B4	hydroxysteroid 17-beta dehydroge	Homo sapiens
779	PRDX5	peroxiredoxin 5(PRDX5)	Homo sapiens
780	PYCR2	pyrroline-5-carboxylate reductase	Homo sapiens
781	PLK1	polo like kinase 1(PLK1)	Homo sapiens
782	PLOD1	procollagen-lysine,2-oxoglutarate	Homo sapiens
783	IL4I1	interleukin 4 induced 1(IL4I1)	Homo sapiens
784	PLOD2	procollagen-lysine,2-oxoglutarate	Homo sapiens
785	HSPA2	heat shock protein family A (Hsp7	Homo sapiens
786	HSPA5	heat shock protein family A (Hsp7	Homo sapiens
787	HSPA8	heat shock protein family A (Hsp7	Homo sapiens
788	HSPB1	heat shock protein family B (small	Homo sapiens
789	CNR1	cannabinoid receptor 1(CNR1)	Homo sapiens
790	CNR2	cannabinoid receptor 2(CNR2)	Homo sapiens
791	VCP	valosin containing protein(VCP)	Homo sapiens
792	PADI1	peptidyl arginine deiminase 1(PA	Homo sapiens
793	HSP90AA1	heat shock protein 90 alpha family	Homo sapiens
794	VDR	vitamin D receptor(VDR)	Homo sapiens
795	COL1A1	collagen type I alpha 1 chain(COL	Homo sapiens
796	HSP90AB1	heat shock protein 90 alpha family	Homo sapiens
797	PMP2	peripheral myelin protein 2(PMP2	Homo sapiens
798	VIM	vimentin(VIM)	Homo sapiens
799	MAPK8IP1	mitogen-activated protein kinase 8	Homo sapiens
800	VLDLR	very low density lipoprotein recep	Homo sapiens
801	NDST1	N-deacetylase and N-sulfotransfer	Homo sapiens
802	TRPV1	transient receptor potential cation	Homo sapiens
803	PMS2	PMS1 homolog 2, mismatch repai	Homo sapiens
804	HTR1A	5-hydroxytryptamine receptor 1A(	Homo sapiens
805	HTR1B	5-hydroxytryptamine receptor 1B(	Homo sapiens
806	HTR1D	5-hydroxytryptamine receptor 1D(	Homo sapiens
807	HTR2A	5-hydroxytryptamine receptor 2A(	Homo sapiens
808	ERO1B	endoplasmic reticulum oxidoreduc	Homo sapiens
809	HTR2B	5-hydroxytryptamine receptor 2B(	Homo sapiens
810	PNLIP	pancreatic lipase(PNLIP)	Homo sapiens
811	HTR2C	5-hydroxytryptamine receptor 2C(	Homo sapiens
812	HTR3A	5-hydroxytryptamine receptor 3A(	Homo sapiens
813	COMT	catechol-O-methyltransferase(CO	Homo sapiens
814	PNMT	phenylethanolamine N-methyltran	Homo sapiens
815	NMNAT1	nicotinamide nucleotide adenylylt	Homo sapiens
816	HTR6	5-hydroxytryptamine receptor 6(H	Homo sapiens
817	HTR7	5-hydroxytryptamine receptor 7(H	Homo sapiens
818	P2RY12	purinergic receptor P2Y12(P2RY1	Homo sapiens
819	POLA1	DNA polymerase alpha 1, catalyti	Homo sapiens
820	COX4I1	cytochrome c oxidase subunit 4I1(	Homo sapiens
821	POLB	DNA polymerase beta(POLB)	Homo sapiens
822	CYP3A43	cytochrome P450 family 3 subfam	Homo sapiens
823	NPEPPS	aminopeptidase puromycin sensiti	Homo sapiens
824	COX5B	cytochrome c oxidase subunit 5B(	Homo sapiens
825	POLE	DNA polymerase epsilon, catalyti	Homo sapiens
826	DHRS11	dehydrogenase/reductase 11(DHR	Homo sapiens
827	POLE2	DNA polymerase epsilon 2, acces	Homo sapiens
828	RRM2B	ribonucleotide reductase regulator	Homo sapiens
829	EGLN1	egl-9 family hypoxia inducible fac	Homo sapiens
830	COX6A2	cytochrome c oxidase subunit 6A2	Homo sapiens
831	COX6B1	cytochrome c oxidase subunit 6B1	Homo sapiens
832	COX6C	cytochrome c oxidase subunit 6C(	Homo sapiens
833	COX7A1	cytochrome c oxidase subunit 7A1	Homo sapiens
834	POMC	proopiomelanocortin(POMC)	Homo sapiens
835	COX7B	cytochrome c oxidase subunit 7B(	Homo sapiens
836	COX7C	cytochrome c oxidase subunit 7C(	Homo sapiens
837	COX8A	cytochrome c oxidase subunit 8A(	Homo sapiens
838	POR	cytochrome p450 oxidoreductase(	Homo sapiens
839	UGT1A9	UDP glucuronosyltransferase fami	Homo sapiens
840	XDH	xanthine dehydrogenase(XDH)	Homo sapiens
841	CPA1	carboxypeptidase A1(CPA1)	Homo sapiens
842	POLE4	DNA polymerase epsilon 4, acces	Homo sapiens
843	SLC13A3	solute carrier family 13 member 3	Homo sapiens
844	CPD	carboxypeptidase D(CPD)	Homo sapiens
845	PPARA	peroxisome proliferator activated	Homo sapiens
846	IDH1	isocitrate dehydrogenase (NADP(	Homo sapiens
847	PPARD	peroxisome proliferator activated	Homo sapiens
848	H6PD	hexose-6-phosphate dehydrogenas	Homo sapiens
849	IDH3A	isocitrate dehydrogenase (NAD(+	Homo sapiens
850	PPARG	peroxisome proliferator activated	Homo sapiens
851	IDH3B	isocitrate dehydrogenase (NAD(+	Homo sapiens
852	MED1	mediator complex subunit 1(MED	Homo sapiens
853	IDH3G	isocitrate dehydrogenase (NAD(+	Homo sapiens
854	SUCNR1	succinate receptor 1(SUCNR1)	Homo sapiens
855	PPIA	peptidylprolyl isomerase A(PPIA)	Homo sapiens
856	PPIB	peptidylprolyl isomerase B(PPIB)	Homo sapiens
857	PPIC	peptidylprolyl isomerase C(PPIC)	Homo sapiens
858	CREB1	cAMP responsive element binding	Homo sapiens
859	YWHAE	tyrosine 3-monooxygenase/trypto	Homo sapiens
860	SF1	splicing factor 1(SF1)	Homo sapiens
861	CRH	corticotropin releasing hormone(C	Homo sapiens
862	PARS2	prolyl-tRNA synthetase 2, mitoch	Homo sapiens

863	NMNAT3	nicotinamide nucleotide adenylylt	Homo sapiens
864	NDUFA11	NADH:ubiquinone oxidoreductas	Homo sapiens
865	PPP1CC	protein phosphatase 1 catalytic sul	Homo sapiens
866	IFNB1	interferon beta 1(IFNB1)	Homo sapiens
867	IFNG	interferon gamma(IFNG)	Homo sapiens
868	AGXT2	alanine--glyoxylate aminotransfer	Homo sapiens
869	PPP2CA	protein phosphatase 2 catalytic sul	Homo sapiens
870	PPP2CB	protein phosphatase 2 catalytic sul	Homo sapiens
871	ABCG1	ATP binding cassette subfamily G	Homo sapiens
872	CRYZ	crystallin zeta(CRYZ)	Homo sapiens
873	IGF1R	insulin like growth factor 1 recept	Homo sapiens
874	P4HTM	prolyl 4-hydroxylase, transmembr	Homo sapiens
875	PPP3CA	protein phosphatase 3 catalytic sul	Homo sapiens
876	IGF2R	insulin like growth factor 2 recept	Homo sapiens
877	PPP3R1	protein phosphatase 3 regulatory s	Homo sapiens
878	PPP5C	protein phosphatase 5 catalytic sul	Homo sapiens
879	PPT1	palmitoyl-protein thioesterase 1(P	Homo sapiens
880	IGHG1	immunoglobulin heavy constant g	Homo sapiens
881	IGHG2	immunoglobulin heavy constant g	Homo sapiens
882	CSNK1G2	casein kinase 1 gamma 2(CSNK1	Homo sapiens
883	CSNK2A1	casein kinase 2 alpha 1(CSNK2A1	Homo sapiens
884	CSNK2B	casein kinase 2 beta(CSNK2B)	Homo sapiens
885	NCAN	neurocan(NCAN)	Homo sapiens
886	CSRP1	cysteine and glycine rich protein 1	Homo sapiens
887	PRKAA1	protein kinase AMP-activated cata	Homo sapiens
888	IGKC	immunoglobulin kappa constant(I	Homo sapiens
889	PRKAA2	protein kinase AMP-activated cata	Homo sapiens
890	SLC25A10	solute carrier family 25 member 1	Homo sapiens
891	PRKAB1	protein kinase AMP-activated non	Homo sapiens
892	PRKAB2	protein kinase AMP-activated non	Homo sapiens
893	PRKACA	protein kinase cAMP-activated ca	Homo sapiens
894	PKIA	cAMP-dependent protein kinase in	Homo sapiens
895	PRKAG1	protein kinase AMP-activated non	Homo sapiens
896	PRKAR1A	protein kinase cAMP-dependent ty	Homo sapiens
897	CALY	calcyon neuron specific vesicular	Homo sapiens
898	PRKAR2B	protein kinase cAMP-dependent ty	Homo sapiens
899	PRKCA	protein kinase C alpha(PRKCA)	Homo sapiens
900	PRKCB	protein kinase C beta(PRKCB)	Homo sapiens
901	PRKCD	protein kinase C delta(PRKCD)	Homo sapiens
902	PRKCE	protein kinase C epsilon(PRKCE)	Homo sapiens
903	PRKCG	protein kinase C gamma(PRKCG)	Homo sapiens
904	PRKCI	protein kinase C iota(PRKCI)	Homo sapiens
905	CTH	cystathionine gamma-lyase(CTH)	Homo sapiens
906	PRKCQ	protein kinase C theta(PRKCQ)	Homo sapiens
907	PRKCZ	protein kinase C zeta(PRKCZ)	Homo sapiens
908	MAPK1	mitogen-activated protein kinase 1	Homo sapiens
909	MAPK3	mitogen-activated protein kinase 3	Homo sapiens
910	IKBKB	inhibitor of nuclear factor kappa B	Homo sapiens
911	CTRB1	chymotrypsinogen B1(CTRB1)	Homo sapiens
912	IL1B	interleukin 1 beta(IL1B)	Homo sapiens
913	MAPK10	mitogen-activated protein kinase 1	Homo sapiens
914	CTSB	cathepsin B(CTSB)	Homo sapiens
915	MAP2K1	mitogen-activated protein kinase k	Homo sapiens
916	MAP2K2	mitogen-activated protein kinase k	Homo sapiens
917	IL6	interleukin 6(IL6)	Homo sapiens
918	PRLR	prolactin receptor(PRLR)	Homo sapiens
919	PRODH	proline dehydrogenase 1(PRODH)	Homo sapiens
920	CYBA	cytochrome b-245 alpha chain(CY	Homo sapiens
921	CYBB	cytochrome b-245 beta chain(CYE	Homo sapiens
922	CYC1	cytochrome c1(CYC1)	Homo sapiens
923	RPL10L	ribosomal protein L10 like(RPL10	Homo sapiens
924	CYP1A1	cytochrome P450 family 1 subfam	Homo sapiens
925	CYP1A2	cytochrome P450 family 1 subfam	Homo sapiens
926	CYP1B1	cytochrome P450 family 1 subfam	Homo sapiens
927	PRSS1	serine protease 1(PRSS1)	Homo sapiens
928	CYP2A6	cytochrome P450 family 2 subfam	Homo sapiens
929	PRSS3	serine protease 3(PRSS3)	Homo sapiens
930	CYP3A7	cytochrome P450 family 3 subfam	Homo sapiens
931	CYP2B6	cytochrome P450 family 2 subfam	Homo sapiens
932	SLC36A1	solute carrier family 36 member 1	Homo sapiens
933	NDUFS7	NADH:ubiquinone oxidoreductas	Homo sapiens
934	CYP2C8	cytochrome P450 family 2 subfam	Homo sapiens
935	CYP2C9	cytochrome P450 family 2 subfam	Homo sapiens
936	CYP2D6	cytochrome P450 family 2 subfam	Homo sapiens
937	IMPDH1	inosine monophosphate dehydrog	Homo sapiens
938	IMPDH2	inosine monophosphate dehydrog	Homo sapiens
939	CYP2E1	cytochrome P450 family 2 subfam	Homo sapiens
940	CDK15	cyclin dependent kinase 15(CDK1	Homo sapiens
941	TRPM7	transient receptor potential cation	Homo sapiens
942	CYP3A4	cytochrome P450 family 3 subfam	Homo sapiens
943	CYP3A5	cytochrome P450 family 3 subfam	Homo sapiens
944	CXCL10	C-X-C motif chemokine ligand 10	Homo sapiens
945	CYP4A11	cytochrome P450 family 4 subfam	Homo sapiens
946	INS	insulin(INS)	Homo sapiens
947	DHRS4L1	dehydrogenase/reductase 4 like 1 (	Homo sapiens
948	CYP11B2	cytochrome P450 family 11 subfa	Homo sapiens
949	CYP17A1	cytochrome P450 family 17 subfa	Homo sapiens
950	CYP19A1	cytochrome P450 family 19 subfa	Homo sapiens
951	CYP27B1	cytochrome P450 family 27 subfa	Homo sapiens
952	INSR	insulin receptor(INSR)	Homo sapiens
953	NDUFA4L2	NDUFA4 mitochondrial complex	Homo sapiens
954	DGKA	diacylglycerol kinase alpha(DGK	Homo sapiens
955	SLCO1B3	solute carrier organic anion transp	Homo sapiens
956	DAO	D-amino acid oxidase(DAO)	Homo sapiens
957	DAPK1	death associated protein kinase 1(	Homo sapiens
958	ISG20	interferon stimulated exonuclease	Homo sapiens
959	DBH	dopamine beta-hydroxylase(DBH)	Homo sapiens
960	ECI1	enoyl-CoA delta isomerase 1(ECI	Homo sapiens
961	DCK	deoxycytidine kinase(DCK)	Homo sapiens
962	PTGER1	prostaglandin E receptor 1(PTGE	Homo sapiens
963	ITGAL	integrin subunit alpha L(ITGAL)	Homo sapiens
964	PTGER2	prostaglandin E receptor 2(PTGE	Homo sapiens
965	ACE	angiotensin I converting enzyme(	Homo sapiens
966	PTGER3	prostaglandin E receptor 3(PTGE	Homo sapiens
967	PTGER4	prostaglandin E receptor 4(PTGE	Homo sapiens
968	ITGB2	integrin subunit beta 2(ITGB2)	Homo sapiens
969	PTGIR	prostaglandin I2 receptor(PTGIR)	Homo sapiens
970	PTGIS	prostaglandin I2 synthase(PTGIS)	Homo sapiens
971	LCMT2	leucine carboxyl methyltransferas	Homo sapiens
972	DDC	dopa decarboxylase(DDC)	Homo sapiens
973	AKR1C1	aldo-keto reductase family 1 mem	Homo sapiens
974	PTGS1	prostaglandin-endoperoxide synth	Homo sapiens
975	AKR1C2	aldo-keto reductase family 1 mem	Homo sapiens
976	PTGS2	prostaglandin-endoperoxide synth	Homo sapiens
977	ZYX	zyxin(ZYX)	Homo sapiens
978	ARL5A	ADP ribosylation factor like GTP	Homo sapiens
979	PHGDH	phosphoglycerate dehydrogenase(	Homo sapiens
980	B3GAT3	beta-1,3-glucuronyltransferase 3(	Homo sapiens
981	DDX5	DEAD-box helicase 5(DDX5)	Homo sapiens
982	HP1BP3	heterochromatin protein 1 binding	Homo sapiens
983	ITPKA	inositol-trisphosphate 3-kinase A(	Homo sapiens
984	NSDHL	NAD(P) dependent steroid dehydr	Homo sapiens
985	IVD	isovaleryl-CoA dehydrogenase(IV	Homo sapiens
986	DECR1	2,4-dienoyl-CoA reductase 1(DEC	Homo sapiens
987	JAK1	Janus kinase 1(JAK1)	Homo sapiens
988	PTPN1	protein tyrosine phosphatase non-	Homo sapiens
989	CLEC4E	C-type lectin domain family 4 me	Homo sapiens
990	KCNA4	potassium voltage-gated channel s	Homo sapiens
991	HIBCH	3-hydroxyisobutyryl-CoA hydrola	Homo sapiens
992	CANT1	calcium activated nucleotidase 1(	Homo sapiens
993	TUBA1A	tubulin alpha 1a(TUBA1A)	Homo sapiens
994	PLA2G2D	phospholipase A2 group IID(PLA	Homo sapiens
995	KCNH2	potassium voltage-gated channel s	Homo sapiens
996	KCNJ3	potassium inwardly rectifying cha	Homo sapiens
997	UCKL1	uridine-cytidine kinase 1 like 1(U	Homo sapiens
998	DHCR7	7-dehydrocholesterol reductase(D	Homo sapiens
999	DHFR	dihydrofolate reductase(DHFR)	Homo sapiens
1000	AKR1B10	aldo-keto reductase family 1 mem	Homo sapiens
1001	PVR	PVR cell adhesion molecule(PVR	Homo sapiens
1002	DHODH	dihydroorotate dehydrogenase (qu	Homo sapiens
1003	CYB5R3	cytochrome b5 reductase 3(CYB5	Homo sapiens
1004	NQO1	NAD(P)H quinone dehydrogenase	Homo sapiens
1005	KCNMA1	potassium calcium-activated chan	Homo sapiens
1006	PYCR1	pyrroline-5-carboxylate reductase	Homo sapiens
1007	RAB7A	RAB7A, member RAS oncogene	Homo sapiens
1008	DLAT	dihydrolipoamide S-acetyltransfer	Homo sapiens
1009	DLD	dihydrolipoamide dehydrogenase(	Homo sapiens
1010	PYGL	glycogen phosphorylase L(PYGL)	Homo sapiens
1011	PYGM	glycogen phosphorylase, muscle a	Homo sapiens
1012	DLG4	discs large MAGUK scaffold prot	Homo sapiens
1013	KRTAP5-2	keratin associated protein 5-2(KR	Homo sapiens
1014	GRIN3A	glutamate ionotropic receptor NM	Homo sapiens
1015	GAPDHS	glyceraldehyde-3-phosphate dehy	Homo sapiens
1016	GRIN3B	glutamate ionotropic receptor NM	Homo sapiens
1017	CHRNA10	cholinergic receptor nicotinic alph	Homo sapiens
1018	TOP1MT	DNA topoisomerase I mitochondr	Homo sapiens
1019	HS3ST3A1	heparan sulfate-glucosamine 3-sul	Homo sapiens
1020	QDPR	quinoid dihydropteridine reductas	Homo sapiens
1021	HS3ST1	heparan sulfate-glucosamine 3-sul	Homo sapiens
1022	CHRFAM7A	CHRNA7 (exons 5-10) and FAM	Homo sapiens
1023	SLC23A1	solute carrier family 23 member 1	Homo sapiens
1024	ALDH5A1	aldehyde dehydrogenase 5 family	Homo sapiens
1025	RAB5A	RAB5A, member RAS oncogene	Homo sapiens
1026	DDX39B	DExD-box helicase 39B(DDX39E	Homo sapiens
1027	NR1I3	nuclear receptor subfamily 1 grou	Homo sapiens
1028	NR1H4	nuclear receptor subfamily 1 grou	Homo sapiens
1029	HSD17B8	hydroxysteroid 17-beta dehydroge	Homo sapiens
1030	RAC1	Rac family small GTPase 1(RAC1	Homo sapiens
1031	RAC2	Rac family small GTPase 2(RAC2	Homo sapiens
1032	DNMT1	DNA methyltransferase 1(DNMT	Homo sapiens
1033	TRDMT1	tRNA aspartic acid methyltransfer	Homo sapiens
1034	RAD51	RAD51 recombinase(RAD51)	Homo sapiens
1035	GPBAR1	G protein-coupled bile acid recept	Homo sapiens
1036	KRT2	keratin 2(KRT2)	Homo sapiens
1037	RALA	RAS like proto-oncogene A(RAL	Homo sapiens
1038	DPP4	dipeptidyl peptidase 4(DPP4)	Homo sapiens
1039	RAN	RAN, member RAS oncogene fan	Homo sapiens
1040	GLT6D1	glycosyltransferase 6 domain cont	Homo sapiens
1041	EGLN3	egl-9 family hypoxia inducible fac	Homo sapiens
1042	DPYD	dihydropyrimidine dehydrogenase	Homo sapiens
1043	EGLN2	egl-9 family hypoxia inducible fac	Homo sapiens
1044	DPYSL2	dihydropyrimidinase like 2(DPYS	Homo sapiens
1045	CYSLTR2	cysteinyl leukotriene receptor 2(C	Homo sapiens
1046	KRT12	keratin 12(KRT12)	Homo sapiens
1047	DRD1	dopamine receptor D1(DRD1)	Homo sapiens
1048	DRD2	dopamine receptor D2(DRD2)	Homo sapiens
1049	DRD3	dopamine receptor D3(DRD3)	Homo sapiens
1050	RAP2A	RAP2A, member of RAS oncogen	Homo sapiens
1051	GNPDA1	glucosamine-6-phosphate deamina	Homo sapiens
1052	DRD4	dopamine receptor D4(DRD4)	Homo sapiens
1053	DRD5	dopamine receptor D5(DRD5)	Homo sapiens
1054	RARA	retinoic acid receptor alpha(RAR	Homo sapiens
1055	RARB	retinoic acid receptor beta(RARB)	Homo sapiens
1056	RARG	retinoic acid receptor gamma(RAI	Homo sapiens
1057	SRD5A3	steroid 5 alpha-reductase 3(SRD5	Homo sapiens
1058	DSP	desmoplakin(DSP)	Homo sapiens
1059	DTYMK	deoxythymidylate kinase(DTYMK	Homo sapiens
1060	RBP1	retinol binding protein 1(RBP1)	Homo sapiens
1061	OGFOD2	2-oxoglutarate and iron dependent	Homo sapiens
1062	DUT	deoxyuridine triphosphatase(DUT	Homo sapiens
1063	GLRA3	glycine receptor alpha 3(GLRA3)	Homo sapiens
1064	LALBA	lactalbumin alpha(LALBA)	Homo sapiens
1065	RCVRN	recoverin(RCVRN)	Homo sapiens
1066	RDH5	retinol dehydrogenase 5(RDH5)	Homo sapiens
1067	FARSB	phenylalanyl-tRNA synthetase sul	Homo sapiens
1068	ABCC5	ATP binding cassette subfamily C	Homo sapiens
1069	ABCC9	ATP binding cassette subfamily C	Homo sapiens
1070	NR1H3	nuclear receptor subfamily 1 grou	Homo sapiens
1071	REN	renin(REN)	Homo sapiens
1072	LCK	LCK proto-oncogene, Src family t	Homo sapiens
1073	LCN2	lipocalin 2(LCN2)	Homo sapiens
1074	LCT	lactase(LCT)	Homo sapiens
1075	LDHA	lactate dehydrogenase A(LDHA)	Homo sapiens
1076	ECHS1	enoyl-CoA hydratase, short chain	Homo sapiens
1077	LDHB	lactate dehydrogenase B(LDHB)	Homo sapiens
1078	LDHC	lactate dehydrogenase C(LDHC)	Homo sapiens
1079	LGALS1	galectin 1(LGALS1)	Homo sapiens
1080	LGALS2	galectin 2(LGALS2)	Homo sapiens
1081	EDNRA	endothelin receptor type A(EDNR	Homo sapiens
1082	LGALS3	galectin 3(LGALS3)	Homo sapiens
1083	EDNRB	endothelin receptor type B(EDNR	Homo sapiens
1084	RHEB	Ras homolog, mTORC1 binding(	Homo sapiens
1085	PPIF	peptidylprolyl isomerase F(PPIF)	Homo sapiens
1086	RHO	rhodopsin(RHO)	Homo sapiens
1087	LGALS7	galectin 7(LGALS7)	Homo sapiens
1088	YARS2	tyrosyl-tRNA synthetase 2(YARS	Homo sapiens
1089	EEF1A1	eukaryotic translation elongation f	Homo sapiens
1090	NDUFA13	NADH:ubiquinone oxidoreductas	Homo sapiens
1091	EEF2	eukaryotic translation elongation f	Homo sapiens
1092	RNASE1	ribonuclease A family member 1,	Homo sapiens
1093	RNASE2	ribonuclease A family member 2(	Homo sapiens
1094	ARL5B	ADP ribosylation factor like GTP	Homo sapiens
1095	RNASE3	ribonuclease A family member 3(	Homo sapiens
1096	FADS1	fatty acid desaturase 1(FADS1)	Homo sapiens
1097	EGF	epidermal growth factor(EGF)	Homo sapiens
1098	EGFR	epidermal growth factor receptor(	Homo sapiens
1099	EHHADH	enoyl-CoA hydratase and 3-hydro	Homo sapiens
1100	SLC25A2	solute carrier family 25 member 2	Homo sapiens
1101	AASS	aminoadipate-semialdehyde synth	Homo sapiens
1102	P3H2	prolyl 3-hydroxylase 2(P3H2)	Homo sapiens
1103	EIF2S3	eukaryotic translation initiation fa	Homo sapiens
1104	TMLHE	trimethyllysine hydroxylase, epsil	Homo sapiens
1105	RPL26L1	ribosomal protein L26 like 1(RPL	Homo sapiens
1106	EPHA2	EPH receptor A2(EPHA2)	Homo sapiens
1107	SLC25A15	solute carrier family 25 member 1	Homo sapiens
1108	LPL	lipoprotein lipase(LPL)	Homo sapiens
1109	SAR1B	secretion associated Ras related G	Homo sapiens
1110	EIF4E	eukaryotic translation initiation fa	Homo sapiens
1111	ALKBH3	alkB homolog 3, alpha-ketoglutar	Homo sapiens
1112	OGFOD1	2-oxoglutarate and iron dependent	Homo sapiens
1113	HRH4	histamine receptor H4(HRH4)	Homo sapiens
1114	TNK2	tyrosine kinase non receptor 2(TN	Homo sapiens
1115	RORA	RAR related orphan receptor A(R	Homo sapiens
1116	LSS	lanosterol synthase(LSS)	Homo sapiens
1117	PRMT3	protein arginine methyltransferase	Homo sapiens
1118	LTF	lactotransferrin(LTF)	Homo sapiens
1119	CALCRL	calcitonin receptor like receptor(C	Homo sapiens
1120	TRIM13	tripartite motif containing 13(TRI	Homo sapiens
1121	TRBC1	T cell receptor beta constant 1(TR	Homo sapiens
1122	HSD17B11	hydroxysteroid 17-beta dehydroge	Homo sapiens
1123	LYN	LYN proto-oncogene, Src family t	Homo sapiens
1124	LYZ	lysozyme(LYZ)	Homo sapiens
1125	ENO1	enolase 1(ENO1)	Homo sapiens
1126	RPL3	ribosomal protein L3(RPL3)	Homo sapiens
1127	M6PR	mannose-6-phosphate receptor, ca	Homo sapiens
1128	RPL4	ribosomal protein L4(RPL4)	Homo sapiens
1129	DCXR	dicarbonyl and L-xylulose reducta	Homo sapiens
1130	RSL24D1	ribosomal L24 domain containing	Homo sapiens
1131	RPL8	ribosomal protein L8(RPL8)	Homo sapiens
1132	RPL10	ribosomal protein L10(RPL10)	Homo sapiens
1133	RPL11	ribosomal protein L11(RPL11)	Homo sapiens
1134	RPL15	ribosomal protein L15(RPL15)	Homo sapiens
1135	RPL18	ribosomal protein L18(RPL18)	Homo sapiens
1136	RPL19	ribosomal protein L19(RPL19)	Homo sapiens
1137	CCBL1	Kynurenine Aminotransferase 1(K	Homo sapiens
1138	DHFRL1	Dihydrofolate Reductase 2(DHFI	Homo sapiens
1139	TK	Transketolase(TKT)	Homo sapiens
1140	ATP5L	ATP Synthase Membrane Subunit	Homo sapiens
1141	TSTA3	GDP-L-Fucose Synthase(GFUS)	Homo sapiens
1142	WARS	Tryptophanyl-TRNA Synthetase 1	Homo sapiens
1143	ACS3	Twist Family BHLH Transcriptio	Homo sapiens
1144	TARS	Threonyl-TRNA Synthetase 1(TA	Homo sapiens
1145	ADRBK1	G Protein-Coupled Receptor Kina	Homo sapiens
1146	EFTUD1	Elongation Factor Like GTPase 1(	Homo sapiens
1147	LIP3	Zinc Finger Protein 521(ZNF521)	Homo sapiens
1148	AARS	Alanyl-TRNA Synthetase 1(AAR	Homo sapiens
1149	ADRBK2	G Protein-Coupled Receptor Kina	Homo sapiens
1150	ATP5C1	ATP Synthase F1 Subunit Gamma	Homo sapiens
1151	TPX1	Cysteine Rich Secretory Protein 2	Homo sapiens
1152	ADSSL1	Adenylosuccinate Synthase 1(AD	Homo sapiens
1153	ATP6	Mitochondrially Encoded ATP Sy	Homo sapiens
1154	PB1	Polybromo 1(PBRM1)	Homo sapiens
1155	P2	Ribosomal Protein Lateral Stalk S	Homo sapiens
1156	KARS	Lysyl-TRNA Synthetase 1(KARS	Homo sapiens
1157	ATP5O	ATP Synthase Peripheral Stalk Su	Homo sapiens
1158	PYCRL	Pyrroline-5-Carboxylate Reductas	Homo sapiens
1159	ATP5A1	ATP Synthase F1 Subunit Alpha(	Homo sapiens
1160	ATP5B	ATP Synthase F1 Subunit Beta(A	Homo sapiens
1161	LARS	Leucyl-TRNA Synthetase 1(LAR	Homo sapiens
1162	GSG2	Histone H3 Associated Protein Ki	Homo sapiens
1163	PROSC	Pyridoxal Phosphate Binding Prot	Homo sapiens
1164	YARS	Tyrosyl-TRNA Synthetase 1(YA	Homo sapiens
1165	IARS	Isoleucyl-TRNA Synthetase 1(IA	Homo sapiens
1166	P5CR2	Pyrroline-5-Carboxylate Reductas	Homo sapiens
1167	VARS	Valyl-TRNA Synthetase 1(VARS	Homo sapiens
1168	HRSP12	Reactive Intermediate Imine Dean	Homo sapiens
1169	ASS	argininosuccinate synthase 1(ASS	Homo sapiens
1170	EPRS	Glutamyl-Prolyl-TRNA Synthetas	Homo sapiens
1171	ASNA1	Guided Entry Of Tail-Anchored P	Homo sapiens
1172	PAPS	3'-Phosphoadenosine 5'-Phosphos	Homo sapiens

Table S4 early NONFH-related symptom genes

Database symptom entries	hip pain / Arthralgia of the hip	Episodic pain / paroxysmal pain	Joint swelling	Tongue_Edema	Ecchymosis	Chest tightness	Obesity/ Overweight	anorexia	Indigestion / Dyspepsia	Vertigo / dizziness	Xerostomia	Epigastric pain	Abdominal distention	Loose stool	Lethargy	Intermittent claudication
	BSCL2	ATRX	ACTC1	SERPING1	NABP1	COPD	ABCB4	ASXL1	COL4A5	H1R	ASXL1	CFI	BMPER	CORT	ADA2	CACNA1G-AS1
	COL2A1	CDKN1B	ALB		F13B		ARMC5	CBL	COL4A6	GRXCR1	CBL	ALMS1	COL11A1	ADGRV1	ATP5MK	AGXT2
	HNRNPA	CPT2	CITED2		GGCX		BAP1	CDKN1B	VMA21	TECRL	MAGEL2		COL11A2	TPH2	CDKN1B	ANGPTL6
	HNRNPA	DNMT3A	FLT4		NUMA1		BLM	MEN1	PRLHR	TPK1	RUNX1		CYP27B1	TFF3	CDON	EIF3E
	LMNA	ESR1	FN1		IRF2BP2		BPTF	PKHD1	MIR325	STRN4	SLC12A2		CYP2R1		COX5A	FURIN
	MATN3	LPL	GATA4		CHST14		CDH23	RUNX1	SYMPK	CKMT1B	TET2		DYNC2I1		CYTB	DHX40
	TRPV4	MAX	MYD88		GFI1B		CEP164	GBA	CELA3B	GYG1	ANG		DYNC2I2		DDC	KLRB1
	VCP	NF1	NKX2-5		TBXAS1		CFAP418	HCRT	PPY	CHRNA6	TBK1		EPCAM		DLL1	AGXT
	ZMPSTE2	RET	PRKAR1A		SBDS		COA3	PTGDS	SSTR3	NABP1	TREM2		G6PC1		DUOX2	XYLT1
	DMD	CDC73	CD247		TBXA2R		COL10A1	MPL	CYSLTR2	CHRNB3	HNRNPA1		GBA		DUOXA2	XYLT2
	FIP1L1	DLST	CHEK2		GP6		COPB1	MBP	KCNIP3	STRC	FGF10		GNE		FGF8	BAK1
	BEST1	EPAS1	COL1A1		CD109		DDB1	DBH	MCM5	SPRTN	SCN9A		GNPTAB		FGFR1	BAD
	AURKB	F12	COL5A1		USP8		DDX6	ZGLP1	HTR3A	TRDN	CHMP2B		HMBS		GATA1	ALDH7A1
	CCL22	FH	COL5A2		FIP1L1		DNAJC30	STAR	UGT1A9	CASQ2	OPTN		IFT122		GLI2	F8
	RHAG	FLI1	FGA		F13A1		DYNC2I2	NPPB	ATP12A	GCDH	FIG4		IFT52		HESX1	ABCC6
	PDGFRA	GNE	FGB		GP1BA		DYRK1B	CRHR1	ATP4A	PFN2	PFN1		INPPL1		IYD	BCAR1
	SLC45A2	HEXB	FGG		ZBTB16		EXOC6B	GHRL	AGA	TUBB6	PON2		KAT6A		LHX3	BBC3
	ACR	KIF1B	HLA-DRB1		PRF1		FMR1	UMOD	SCN10A	FOXI1	EPHA4		MTOR		LHX4	ZMPSTE24
	ATF3	MC2R	IL10		TBL1XR1		FXR1	TNFRSF1	SEMA4D	KLRC4	NEK1		NKX3-2		MAGEL2	FGF13
	ADAM11	MDH2	IL2RA		P2RY12		GNAS-AS1	ND1	CTBP1	ASIC3	NEFH		PLEC		MTHFR	SIRT2
	IFNG	MEFV	IL2RB		GP1BB		HDAC8	SLC4A1	TPH1	NOP56	AFG3L2		PSMB8		NDUFAF	MPL
	CRYBG1	MRAP	IL6		ITGA2		HERC2	NTRK3	CYBA	KCNJ13	DCTN1		PTH1R		NFS1	NLRP1
	COMP	NNT	RB1		AIP		IPW	MIR23B	GNB3	COCH	PRPH		PYGL		NODAL	
	AIM2	SDHA	STAT4		MPL		LARS2	GHSR	GGTLC4P	HRH4	CHCHD10		SI		PIGA	
	B2M	SDHAF2	TP53		COL1A1		MAN1B1	IFNGR1	GGTLC5P	CACNB4	CLDN10		SLC37A4		POU1F1	
	COL9A1	SDHB	PTPN2		COL3A1		MAPK8IP3	ND5	GGT2	NAGA	TAF15		SMARCAL1		PROP1	
	COL9A2	SDHC	APC2		COL5A1		MCM3AP	CCR1	GGTLC3	CHRNB4	GLE1		SMPD1		PTCH1	
	COL9A3	SDHD	EIF2AK4		COL5A2		MIA3	TRNL1	SLC26A4	NUMA1	MATR3		VDR		RPL11	
	NGF	SLC25A11	GATA6		GBA		MKRN3	MMUT	PTGDS	IRF2BP2	CFAP410		ACTG2		RPL15	
		STAR	GBA				MKRN3-AS1	TBL1XR1	SERPINB2	CNNM2	DAO		IRAK1		RPL18	
		TGFBI	GJC2				MTFMT	MC4R	FAP	CLCN1	EDARADD		SLC26A4		RPL26	
		TMEM127	KIF1A				NDNF	GLA	GGT1	SGSH	ANXA11		MYLK		RPL27	
		TXNRD2	MAGEL2				NIPBL	ACAT1	CHGA	UBAC2	UBQLN2		KRT8		RPL35	
		VHL	MEFV				NKAP	CD247	GAST	TRPM4	PON3		SLC4A1		RPL35A	
		SCN10A	MKKS				NPAP1	MNAT1	IL17F	IL12A-AS	VAPB		EPHB4		RPL5	
		SCN11A	MYH6				OCA2	NUCB2	CYP2C19	JUP	TUBB6		WDR35		RPS10	
		TRPA1	NDN				P4HTM	PTGES	PTGS1	TMEM94	CCNF		ITGB4		RPS15A	
		MSC-AS1	NSD1				PCNT	ARVCF	TRPV1	HINT1	UNC13A		RRM2B		RPS17	
			OCA2				PHF21A	OXTR	FHIT	CHRNB2	GLT8D1		MYH11		RPS19	
			PLAA				PIGT	PAM	CHD7	ADCY5			WAS		RPS24	
			RBM8A				PKDCC	ND6	CRH	SLC2A4RG			KRT18		RPS26	
			RNF13				PSMD12	DAXX	S100A8	TES			TNFSF15		RPS27	
			SCARB2				PWAR1	ZBTB16	SLC6A4	MYL2			PSAP		RPS28	
			SETD2				PWRN1	ND4	DECR1	KCNJ10			NKX2-5		RPS29	
			SNORD115-1				RAD21	COX3	MIF	ATP1A2			DUOX2		RPS7	
			SNORD116-1				RNPC3	ALPL	SLCO6A1	PRIMA1			FSHR		SHH	
			SNRPN				RP1L1	TXNRD2	GSTK1	FES			ITGA6		SIX3	
			TBX20				SATB1	HTR1B	HP	CLCNKB			ANK1		SLC19A3	
			TET2				SETD1A	TRNS1	HLA-DQB1	CACNA1G			POU1F1		SMC1A	
			TGM1				SLC10A7	RMC1	COMT	CHRNA3			DLL3		STAG2	
			TLL1				SLC25A4	ERAP1	CD14	AFG3L2			SLC26A2		SUGCT	
			VEGFC				SLC7A7	HMBS	GABPA	CCT			THRB		TG	
			ZNHIT3				SMARCE1	MAGEL2	IL1RN	MYO7A			SLC26A3		TGIF1	
			ADM				SMC1A	MIR340	FOXP3	CRY1			DYNC2H1		TPO	
			ACP5				SMC3	PRSS1	SERPINE1	SIL1			MNX1		TRHR	
			AIP				SNORD115-1	TRNW	NFE2L2	MVK			IL12RB1		TSHB	
			ANKH				SNORD116-1	C4A	IL17A	SCN1B			SPINT2		TSHR	
			ASAH1				SPEN	NTSR1	PTGS2	FIP1L1			POLG2		TSR2	
			ATP7B				STEEP1	AVPR2	TGFB1	SLC1A3			G6PC		YY1	
			BTNL2				TCF20	GIP	IL1B	KCNA1			HESX1		ZIC2	
			CCN6				TERT	RCBTB1	TP53	ERF			LIPA		MTRR	
			CD244				THOC2	GRHL3	TNF	LGI1			THRA		ATG7	
			CIITA				TOGARAM1	ARC	AChE	SLC25A4			FREM2		SLC1A2	
			DDR2				TRAF3IP1	FIP1L1	CCKBR	SPAG9			SLC2A2		GCH1	
			F8				TRAF7	CARTPT	HTR4	C4A			FOXE1		TBK1	
			GPR101				TRIP12	TRNS2	GLP1R	GFER			IFT80		TWNK	
			HFE				WAC	AQP2	CCKAR	PANK2			PROP1		DLD	
			HGD				XRCC4	TRNF	MLNR	DSPP			TCTN2		KRT8	
			HPGD				XYLT1	MC2R		ERAP1			SLC5A1		CYP24A1	
			IL1RN				ZBTB20	OR2AG1		ADRB1			WDR60		KCNMA1	
			LACC1				ZPR1	CRHR2		RABGEF1			TRIP11		SLC4A1	
			LMX1B				COX7C	SLC5A1		TERF2IP			WDR34		ND1	
			LPIN2				ZFR2	CCL28		ADA2			NPHS1		ASS1	
			MIF				PLSCR3	PYY		ZBTB16			INHBA		RRM2B	
			NFKBIL1				GHRLOS	TRNQ		RYR2			MMEL1		KCNJ11	
			NOD2				BCDIN3D-AS	GOLGA6A		CDH23			POU2AF1		KRT18	
			OCRL				CCDC77	UCN3		GLA			PIGQ		MMUT	
			OTULIN				LCE1C	TRNH		TBL1XR1			SLC9A3		NAA10	
			PTPN22				FAM71F1	NNT		NKX2-5			LHX4		CDH23	
			SLC22A4				ZSCAN30	NFAT5		CHRNA7			C1QA		CPT2	
			SLC40A1				WAGRO	SCARB2		CCR1			ALDOB		SIK1	
			SLCO2A1				MIR4777	SLC46A1		SH2B3			UNC80		SFRP2	
			TNFRSF11B				MIR4460	SHCBP1		EPOR			SPIB		CYP11B2	
			ANKRD55				MIR4517	MAPK10		GRM5			SPTA1		CPT1A	
							MACORIS	NPSR1		SCN2A			C1R		POLG2	
							HAUS1	PLCL1		AIP			TTC7A		H3-3A	
							CCDC171	HTR4		SCN5A			MYO5B		HMBS	
							CFAP61	NMU		FKRP			LHX3		SLC25A20	
							CCDC57	CDAN1		THPO			LMOD1		ASPA	
							FLJ33534	NPY2R		ZGLP1			SPTB		OTC	
							EEF1A1P11	C1QL1		CYP3A5			MESP2		NDUFB11	
							TMEM160	CD3E		DBH			ALG8		NBAS	
							PLAAT2	CCKAR		LRRK2			TNPO3		ATP13A2	
							RPL7P9	OXT		MPL			TSHB		SLC25A4	
							FN3KRP	UCP3		SLC26A4			PHKG2		HADHA	
							PERM1	CD200R1		CCHCR1			PRKCSH		FBP1	
							ZNF169	CD3D		NPPA			PHKA2		TCN2	
							GPR12	ECT		RAP1A			SAR1B		NDUFS7	
							LRRC53	OPRL1		ARHGEF2			DUOXA2		PDHA1	
							LRRN4	NPY1R		CYP3A4			IYD		NDUFS3	
							EML6	ABAT		CASP3			TRMU		NDUFS4	
							AP3S2	SLC39A4		TNF			EPB42		USP8	
							LINC00689	PALLD		SLC22A2			DDRGK1		CPS1	
							LINC01412	OGN		IL1B			SEC63		ASL	
							LINC00237	UCN2		IL6			STX3		PHIP	
							ABHD15	IL12A-AS1		BCL2					NDUFS1	
							ZNF543	SLC19A2		ABCB1					SLC25A22	
							GPAT4	UBAC2		FOS					NDUFS2	
							ATP8B3	IRF2BP2		CAT					USP18	
							PHETA2	LRIT1		MAPK1					ND2	
							GEMIN5	NUMA1		MAPK3					SLC7A7	
							ZZZ3	MMACHC		CYP2D6					ND3	
							GM2AP1	PTPN3		BAX					TES	
							HID1	NCOA6		KCNH2					NDE1	
							DNAJB3	HTR2B		BDNF					SLC22A5	
							GPCPD1	HLCS		IL10					NDUFV2	
							SEMA3G	CTRC		REN					BNC2	
							MCHR2-AS1	MLN		NOS2					GALT	
							POLR1B	PDE3B		RELA					SLAMF1	
							SCRN2	KLRC4		VEGFA					EIF2B5	
							ZDHHC24	MRAP		PRL					NDUFV1	
							AP4S1	HTR3B		IFNG					SLC25A13	
							AP4E1	HMGCL		CCL2					GABRB2	
							ACADVL	CARD16		CXCL8					HADHB	
							PAX4	MC3R		BRV2					DPYS	
							TRIP4	HCRTR2		BPPV					HJV	
							AP4M1	NABP1							ALDOB	
							AP4B1	MLX							ABAT	
							C8orf37	NUCB1							GLDC	
							KLF11	IK							SLC39A4	
							APPL1	DYRK3							BTD	
							IGSF1	MMADHC							FOXRED1	
							BBS1	NMUR2							NDUFS8	
							BLK	INSL5							SLC19A2	
							PPP3CA	GPR88							NDUFA1	
							NEUROD1	PYY3							DBT	
							PDX1	GFRAL							ACADM	
							KCNJ11								NDUFAF2	
							GCK								PURA	
							CEL								MMACHC	
							ABCC8								EIF2B2	
							CHD8								NDUFAF3	
							FOXP1								HLCS	
							HESX1								HADH	
							HNF1A								TIMMDC1	
							HNF4A								SLC25A19	
							INS								ACADVL	
							LHX3								CACNB4	
							LHX4								GK	
							POU1F1								EIF2B4	
							PROP1								EIF2B1	
							SPG11								ACADS	
							SPTBN1								NUBPL	
							TRHR								AMT	
							UNC45B								RMND1	
							USP7								ATP5F1D	
															NDUFAF5	
															MCCC2	
															PCCA	
															BCKDHB	
															NDUFS6	
															CA5A	
															NDUFAF4	
															GYS2	
															ACADSB	
															HMGCL	
															PCCB	
															BCKDHA	
															MMAB	
															NDUFB9	
															NDUFAF1	
															MCCC1	
															NDUFB3	
															NDUFA11	
															IVD	
															NDUFA6	
															SLC25A15	
															LMBRD1	
															TMEM126B	
															LYRM7	
															NAGS	
															NDUFB10	
															EIF2B3	
															TRNT	
															ACADL	
															MMAA	
															MMADHC	
															GCSH	
															BOLA3	
															ABCD4	
															NFU1	
															MRPS16	
															NAXD	

Table S5 Details of JHP effective targets

NO.	gene_id	gene_name	Descriptio	Group2_averag	Group1_averag	P.Value	FC	Regulation	Group2_C	Group2_C	Group2_C	Group2_C	Group1_C	Group1_C	Group1_C	Group1_Case4_Count
1	ENSG0000000	MSL3	male-speci	1192.529325	740.7977092	0.007616614	1.607327917	up	1644	842	1013	1284	681	219	909	2687
2	ENSG0000000	YBX2	Y-box binc	0	5.18517751	0.036163012	-27.99020317	down	0	0	0	0	3	0	17	8
3	ENSG0000000	TNFRSF12A	tumor necr	3.813910106	16.03212938	0.034187786	-4.149174867	down	4	0	5	6	13	8	25	20
4	ENSG0000000	SELE	selectin E	0	5.502411535	0.040651647	-28.25626081	down	0	0	0	0	0	7	4	5
5	ENSG0000000	MGST1	microsoma	53.82079015	150.3930985	0.011479236	-2.760770932	down	146	48	17	33	163	90	78	340
6	ENSG0000001	SEMA3G	semaphori	39.72461369	15.59936795	0.040137628	2.519104763	up	16	59	38	28	14	5	20	52
7	ENSG0000001	QPCTL	glutaminyl	73.16851675	33.39290603	0.048084518	2.109528328	up	71	62	81	66	10	8	44	234
8	ENSG0000002	CPS1	carbamoyl-	0	5.972816158	0.009880672	-30.6241748	down	0	0	0	0	5	5	4	5
9	ENSG0000003	GAB2	GRB2 assc	163.5717506	352.5091705	0.044192886	-2.156722626	down	364	184	70	88	413	38	546	1074
10	ENSG0000004	AIFM2	apoptosis i	61.72134298	29.94043933	0.016245185	2.058842979	up	115	34	55	56	18	11	50	95
11	ENSG0000004	PER3	period circ	102.0506664	40.86606752	0.008389	2.429523241	up	153	55	82	126	22	12	32	286
12	ENSG0000006	PIGV	phosphatid	99.46429388	57.95578602	0.042834531	1.696854247	up	135	90	65	107	34	24	52	306
13	ENSG0000007	ZCWPW1	zinc finger	35.39511861	69.53076013	0.004725635	-1.971923039	down	48	32	29	32	52	28	76	274
14	ENSG0000008	SESN1	sestrin 1	449.6942413	281.8218514	0.032665226	1.582640532	up	614	413	332	435	211	65	366	1371
15	ENSG0000008	DLG3	discs large	146.1493567	79.54936668	0.019756672	1.871289374	up	140	128	143	148	44	51	90	208
16	ENSG0000008	ZNF416	zinc finger	61.29582407	19.24997468	0.013868397	3.009224589	up	86	44	60	56	16	0	22	136
17	ENSG0000008	COL16A1	collagen ty	7.430626982	0	0.001242417	41.88914448	up	13	5	4	9	0	0	0	0
18	ENSG0000008	NCOA1	nuclear rec	464.6738956	742.8215211	0.019436154	-1.597011835	down	823	498	293	308	601	302	878	2459
19	ENSG0000008	PPP1R15A	protein phc	612.0619717	1110.420665	0.007363041	-1.812131409	down	902	523	550	492	1063	470	1480	2134
20	ENSG0000008	TMPRSS11E	transmemb	0	4.722415237	0.044147443	-25.28425515	down	0	0	0	0	9	0	4	12
21	ENSG0000008	KIZ	kizuna cen	273.260095	158.6532	0.011287762	1.714112358	up	278	217	261	298	86	64	219	645
22	ENSG0000009	TSPAN15	tetraspanin	54.34527565	18.75009976	0.012583097	2.855851187	up	78	21	44	78	9	5	44	48
23	ENSG0000009	GADD45B	growth arr	294.2943229	451.5046974	0.046477354	-1.527166355	down	512	246	267	192	337	230	572	1089
24	ENSG0000009	DERL3	derlin 3	85.81744127	20.27060722	0.007208725	4.168311678	up	115	25	106	99	36	0	14	75
25	ENSG0000009	GGT5	gamma-glu	0	6.696210945	0.022045183	-34.69609023	down	0	0	0	0	0	8	2	20
26	ENSG0000010	CABP7	calcium bi	0	5.688382415	0.027576414	-29.82681987	down	0	0	0	0	0	5	5	23
27	ENSG0000010	CSF2RB	colony stin	311.7989671	735.7029929	0.006208142	-2.35960497	down	779	280	198	126	586	151	1230	2697
28	ENSG0000010	PYGL	phosphoryl	417.4641555	723.6032445	0.045630494	-1.72969048	down	918	418	240	224	753	332	566	2117
29	ENSG0000010	NFKBIA	NFKB inhi	530.4912291	1075.27044	0.009281854	-2.026348648	down	983	504	302	430	1337	270	1315	2582
30	ENSG0000010	NTSR1	neurotensi	10.54512011	36.61535343	0.003914353	-3.461404396	down	20	12	8	4	45	9	38	114
31	ENSG0000010	RNF24	ring finger	448.9699277	753.8529341	0.022885839	-1.680929783	down	641	385	392	384	510	220	1452	2012
32	ENSG0000010	JAG1	jagged 1	6.49875216	23.7702666	0.018825238	-3.558107942	down	17	8	2	2	28	9	20	66
33	ENSG0000010	ZNF516	zinc finger	243.8778183	479.1112082	0.02005962	-1.966420039	down	533	193	149	180	506	78	624	1875
34	ENSG0000010	RBFA	ribosome b	134.6832721	74.4610087	0.023634928	1.810159539	up	202	75	104	168	36	37	77	343
35	ENSG0000010	USP14	ubiquitin s	222.1805373	346.4171356	0.038897583	-1.5608712	down	284	180	237	177	170	168	346	1660
36	ENSG0000010	RNMT	RNA (guai	273.2005436	519.9099634	0.015383607	-1.904831638	down	434	335	227	108	290	214	599	2411
37	ENSG0000010	ANKRD12	ankyrin rep	1410.426435	936.4453377	0.04991063	1.505309574	up	1741	1180	1122	1520	595	328	1669	2621
38	ENSG0000010	NXT2	nuclear tra	83.45639477	175.3920319	0.024976475	-2.079545283	down	119	43	90	85	120	124	191	259
39	ENSG0000010	SUV39H1	suppressor	94.49262555	44.45246085	0.017101066	2.090519402	up	148	69	117	50	27	16	39	255
40	ENSG0000010	XIAP	X-linked ir	482.4976852	201.7170784	0.035203373	2.394448932	up	806	177	594	421	377	36	142	311
41	ENSG0000010	SMARCA1	SWI/SNF r	0	7.900233969	0.02722439	-42.79525332	down	0	0	0	0	0	0	16	67
42	ENSG0000010	PORCN	porcupine	130.7936087	75.0218243	0.007048404	1.744177536	up	195	103	116	115	67	29	68	296
43	ENSG0000010	FA2H	fatty acid 2	8.441073052	0.853589518	0.037326943	9.037227795	up	12	6	7	9	0	0	4	0
44	ENSG0000010	CRISPLD2	cysteine ric	48.87664272	137.4877404	0.025348918	-2.803495054	down	131	63	16	9	184	32	133	399
45	ENSG0000010	MEFV	Mediterran	229.3587151	448.8744696	0.033594349	-1.963420298	down	363	288	160	116	344	65	721	2058
46	ENSG0000010	AQP9	aquaporin	254.1822434	503.1690144	0.047056865	-1.980730696	down	493	144	313	124	350	102	1158	1105
47	ENSG0000010	CEMIP	cell migrat	6.123529739	23.50470491	0.007762272	-3.906023298	down	5	8	2	8	12	12	19	119
48	ENSG0000010	BLOC1S6	biogenesis	187.906622	696.3940681	0.00021081	-3.704732327	down	198	137	306	82	627	405	281	2588
49	ENSG0000010	STX10	syntaxin 1(	257.1412048	439.953979	0.010241183	-1.703438727	down	402	244	230	167	341	226	513	1121
50	ENSG0000010	WDR83OS	WD repeat	94.59401917	241.0469851	0.022801605	-2.523419005	down	203	39	88	80	130	231	158	309
51	ENSG0000010	PIK3R2	phosphoin	14.82752935	79.44141533	0.01644733	-5.378877986	down	16	10	8	24	10	13	315	12
52	ENSG0000010	NAMPT	nicotinami	723.0026303	1612.970969	0.044795486	-2.231499401	down	1236	348	1015	392	1103	236	4350	2443
53	ENSG0000010	GLCCI1	glucocortic	282.7602456	173.7712132	0.042008916	1.642276906	up	304	200	347	245	143	97	166	446
54	ENSG0000010	FKTN	fukutin	116.3279637	56.45024038	0.018243413	2.027110375	up	107	125	134	74	33	16	96	222
55	ENSG0000010	SYNGR2	synaptogyr	653.411499	1083.054999	0.013110006	-1.655311437	down	914	382	662	671	955	526	883	3662
56	ENSG0000010	MAP2K6	mitogen-ac	64.16126207	182.3118301	0.001970669	-2.868277705	down	79	64	50	59	169	27	132	1194
57	ENSG0000011	PTPMT1	protein tyr	76.78872414	160.0622964	0.012235653	-2.062196169	down	93	54	88	67	91	116	128	452
58	ENSG0000011	CYP27B1	cytochrom	11.20405132	0.669533716	0.045483716	12.33298581	up	4	0	16	21	0	0	0	10
59	ENSG0000011	LIN7A	lin-7 homo	35.14821617	121.7236633	0.005826057	-3.427059278	down	112	25	21	8	107	41	226	176
60	ENSG0000011	FOXM1	forkhead b	69.62508616	31.57139546	0.00973125	2.287471277	up	98	32	76	75	21	20	30	85
61	ENSG0000011	SMIM8	small integ	50.20083334	95.0106095	0.021368741	-1.89562009	down	53	39	36	67	97	33	106	284
62	ENSG0000011	SLC26A8	solute carri	3.276606484	31.01254639	0.005527466	-9.190131171	down	12	4	0	0	22	3	89	40
63	ENSG0000011	PTP4A1	protein tyr	256.8307839	566.6595886	0.011347251	-2.208914756	down	206	269	305	180	658	149	842	1061
64	ENSG0000011	ALDH5A1	aldehyde d	463.3982632	176.9184917	5.6297E-05	2.608199083	up	467	540	430	332	87	70	259	730
65	ENSG0000011	FIG4	FIG4 phos	158.9037617	255.2740658	0.03589846	-1.603147534	down	336	120	133	93	221	92	265	997
66	ENSG0000011	THG1L	tRNA-histi	100.7461309	159.2140147	0.029265466	-1.570249218	down	147	94	84	80	148	69	160	480
67	ENSG0000011	PRLR	prolactin r	5.075445027	31.27093776	0.01814089	-6.029981059	down	16	4	0	4	63	4	4	97
68	ENSG0000011	DPYSL3	dihydropyr	4.619954956	0	0.046113961	25.413123	up	5	5	0	8	0	0	0	0
69	ENSG0000011	KNG1	kininogen	0.170748902	4.917443848	0.048816014	-16.11125911	down	1	0	0	0	8	0	4	20
70	ENSG0000011	CD86	CD86 mol	153.0469653	340.4557916	0.034524714	-2.215843749	down	461	142	50	58	346	156	229	1159
71	ENSG0000011	RTKN	rhotekin	19.81897868	4.072998802	0.013155609	4.723821326	up	13	17	31	12	6	0	7	8
72	ENSG0000011	TFCP2L1	transcriptic	0	9.605102538	0.004890578	-51.24172634	down	0	0	0	0	5	5	0	72
73	ENSG0000011	GCA	grancalcin	351.2904213	763.6539754	0.01086585	-2.171996422	down	899	256	232	185	891	171	994	2131
74	ENSG0000011	FN1	fibronectin	3.612323852	26.84127896	0.001005071	-7.500982534	down	4	5	0	5	32	10	11	111
75	ENSG0000011	TAF1B	TATA-box	60.36953313	115.4774118	0.01895346	-1.901515472	down	110	57	53	31	76	59	91	505
76	ENSG0000011	DCAF17	DDB1 and	78.79510725	260.2149754	0.000521826	-3.29549237	down	106	52	53	105	189	152	85	1255
77	ENSG0000011	MORN1	MORN rep	17.84417474	38.1636346	0.048491918	-2.098872228	down	41	16	6	15	26	18	43	135
78	ENSG0000011	ICMT	isoprenylc	382.6582377	240.1712744	0.043977175	1.586725661	up	418	446	340	270	188	87	208	1167
79	ENSG0000011	SLAMF1	signaling l	377.7934832	220.9073645	0.004090992	1.702997644	up	451	275	351	409	148	88	227	1013
80	ENSG0000011	PADI2	peptidyl ar	91.69360693	202.6280363	0.049276564	-2.212119126	down	224	95	24	62	98	39	480	639
81	ENSG0000011	CCDC181	coiled-coil	0	5.720551175	0.033614491	-30.07832726	down	0	0	0	0	0	4	14	4
82	ENSG0000011	PMFBP1	polyamine	18.43739422	1.272114061	0.01000138	11.05400036	up	16	20	29	4	0	0	0	19
83	ENSG0000011	CSF3R	colony stin	1980.625084	4561.956095	0.039748344	-2.303475177	down	5055	1556	918	1335	2585	384	11853	13673
84	ENSG0000011	FLVCR2	feline leuk	51.8622102	100.9699175	0.044692262	-1.921519804	down	148	32	25	34	64	48	121	355
85	ENSG0000011	HELLS	helicase, ly	106.4194213	45.25237288	0.009926257	2.349566643	up	90	44	134	138	29	21	43	198
86	ENSG0000011	AVPI1	arginine va	8.039175517	21.04421698	0.044067203	-2.553790963	down	16	8	5	5	21	10	13	74
87	ENSG0000012	DUSP1	dual specif	627.5879983	3533.195524	5.7843E-05	-5.628770517	down	1265	382	608	420	4879	522	6612	2072
88	ENSG0000012	INSL6	insulin like	1.534524575	10.63585804	0.049310731	-7.259281827	down	0	5	0	0	11	5	10	25
89	ENSG0000012	TSHZ3	teashirt zin	31.20829934	60.93766643	0.041609131	-1.930945596	down	62	24	34	12	48	28	60	217
90	ENSG0000012	ITPR2	inositol 1,4	269.7987868	421.4504078	0.024810458	-1.567500144	down	374	277	257	165	291	149	469	1948
91	ENSG0000012	ATP7B	ATPase co	7.928226921	27.94979407	0.038060018	-3.431311485	down	20	0	4	12	33	12	22	69
92	ENSG0000012	NR4A1	nuclear rec	45.38644992	121.9663305	0.020232696	-2.680094879	down	125	22	22	39	173	14	100	494
93	ENSG0000012	IKZF4	IKAROS f	82.69732164	27.81683084	0.037672707	2.983946123	up	31	22	185	60	39	8	29	51
94	ENSG0000012	G0S2	G0/G1 swi	5.646282622	163.9906951	0.034206962	-28.93148577	down	16	0	10	0	152	19	471	0
95	ENSG0000012	KCNJ2	potassium	45.12531365	116.5562467	0.040507034	-2.586483761	down	130	42	22	13	28	24	243	603
96	ENSG0000012	NCOA5	nuclear rec	538.7085883	335.6814628	0.034293173	1.593522724	up	787	472	428	483	175	92	457	1820
97	ENSG0000012	MPHOSPH10	M-phase pl	287.9760651	183.6528985	0.042607487	1.553003325	up	423	203	270	268	103	62	194	1030
98	ENSG0000012	TREM1	triggering r	101.9801936	244.6653201	0.014975441	-2.39594789	down	230	36	116	64	287	46	373	582
99	ENSG0000012	C3	complemer	12.69282293	38.46207401	0.041759952	-3.087961333	down	6	12	14	14	17	17	8	306
100	ENSG0000012	TNFSF14	tumor necr	133.0657634	61.99342037	0.045331669	2.121852527	up	98	166	143	85	38	12	145	160
101	ENSG0000012	FOSB	FBJ murin	35.22357498	129.0658456	0.00307187	-3.675277792	down	14	31	58	23	93	63	228	148
102	ENSG0000012	PRRG2	proline ricl	0	10.62014297	0.000662305	-54.92700604	down	0	0	0	0	12	8	5	8
103	ENSG0000012	AUNIP	aurora kina	7.599160341	0.334766858	0.01939918	13.039507	up	5	10	4	9	0	0	0	5
104	ENSG0000012	ADGRE2	adhesion G	242.8225508	421.6082132	0.049805654	-1.739620799	down	394	262	180	153	473	75	574	1371
105	ENSG0000012	PKMYT1	protein kin	28.85718107	5.023115748	0.030396213	5.987261127	up	30	4	60	18	0	5	0	29
106	ENSG0000012	HIP1	huntingtin	101.1643961	181.2122306	0.04068498	-1.797197924	down	134	141	56	67	166	52	264	542
107	ENSG0000012	ZFP36	ZFP36 ring	1195.254321	2409.401524	0.02147937	-2.015907397	down	2105	1035	907	910	3485	401	2668	6063
108	ENSG0000012	APOBEC3A	apolipopro	345.7779252	913.4764975	0.040655732	-2.641210724	down	997	206	305	84	801	78	2359	1332
109	ENSG0000012	DOCK4	dedicator c	6.372250465	35.61512567	0.033882709	-5.546158306	down	20	6	0	4	24	0	106	72
110	ENSG0000012	MKLN1	muskelin 1	198.8685403	405.4496317	0.033455775	-2.046803282	down	255	211	194	122	117	132	355	3114
111	ENSG0000012	MINDY2		222.1587216	108.307309	0.012730253	2.060219516	up	323	181	303	83	93	48	116	333
112	ENSG0000012	RNASE1	ribonuclea	7.057910538	56.29978185	0.004445211	-7.912756349	down	8	10	9	0	12	70	8	110
113	ENSG0000012	FGF13	fibroblast g	0	10.57258528	0.020006234	-55.35130235	down	0	0	0	0	0	13	12	0
114	ENSG0000012	TNNI3	troponin I3	0	7.530945908	0.00429207	-38.81701379	down	0	0	0	0	2	7	10	6
115	ENSG0000013	SAT1	spermidine	2930.516679	6620.28463	0.001431756	-2.258617368	down	6057	2292	1971	2217	6975	2290	9423	12284
116	ENSG0000013	FUT5	fucosyltrar	8.579294105	0	0.000661712	48.11257077	up	6	5	13	8	0	0	0	0
117	ENSG0000013	POMT1	protein O-r	291.8242449	166.7318327	0.034762767	1.730695517	up	316	223	254	342	79	44	253	877
118	ENSG0000013	ANGPTL6	angiopoieti	2.914681478	26.49274662	0.004852678	-8.864045404	down	4	0	0	8	17	23	8	72

119	ENSG0000013	HSD17B3	hydroxyste	1.115842935	18.70759982	0.030145396	-16.79320294	down	0	0	0	4	1	26	0	35
120	ENSG0000013	EPS8L1	EPS8 like	11.91325457	32.20706724	0.048490715	-2.608376651	down	24	3	16	8	18	24	35	57
121	ENSG0000013	EMC8	ER membr	109.9028653	168.2988933	0.043117097	-1.518604464	down	179	101	96	73	111	86	189	555
122	ENSG0000013	LRRC4B	leucine ricl	0	7.57355327	0.011327604	-40.74619613	down	0	0	0	0	5	0	25	8
123	ENSG0000013	NINJ1	ninjurin 1	308.9227652	468.1084329	0.047876678	-1.514961742	down	518	258	302	191	405	156	680	1328
124	ENSG0000013	MTSS1L	metastasis	10.42332043	33.07204658	0.043113456	-3.152678324	down	19	6	3	16	4	21	23	207
125	ENSG0000013	GIMAP4	GTPase, IM	3284.957829	1823.513583	0.000973372	1.799284	up	4987	2543	2986	2806	1551	510	1912	8557
126	ENSG0000013	E2F5	E2F transc	92.46668131	39.30878396	0.018489465	2.27731634	up	148	54	100	77	35	4	44	232
127	ENSG0000013	SORT1	sortilin 1	136.4988368	396.9482484	0.007252093	-2.91342634	down	121	267	56	63	455	135	216	1683
128	ENSG0000013	AP4B1	adaptor rel	319.5916369	188.2113361	0.00643214	1.68175227	up	425	225	335	288	126	60	234	872
129	ENSG0000013	DSC2	desmocolli	17.47837586	51.51197848	0.006518171	-2.944120077	down	41	12	8	16	28	12	104	185
130	ENSG0000013	SDS	serine dehy	6.887580986	0	0.041964342	37.93745696	up	24	0	0	10	0	0	0	0
131	ENSG0000013	PRR5L	proline ricl	218.0560157	121.5967795	0.002572881	1.791533819	up	257	199	211	185	89	53	115	509
132	ENSG0000013	DYSF	dysferlin	270.735226	589.2901615	0.00808298	-2.175743322	down	530	263	196	152	433	178	1198	1142
133	ENSG0000013	DRAM1	DNA dama	25.62398776	99.73909453	0.005360133	-3.888427003	down	30	13	49	8	168	29	70	145
134	ENSG0000013	KLF4	Kruppel-lil	162.5086484	424.74289	0.023866296	-2.613707986	down	405	159	61	96	603	30	471	1499
135	ENSG0000013	TLR4	toll like rec	176.1273688	450.2462277	0.007919632	-2.561014638	down	313	210	151	51	163	162	424	3053
136	ENSG0000013	TRMO	tRNA metl	200.7783874	132.1134046	0.03621257	1.53562906	up	339	122	157	214	99	65	135	441
137	ENSG0000013	SIT1	signaling tl	453.2465847	278.2523722	0.019068552	1.628700126	up	680	283	519	355	141	128	344	1164
138	ENSG0000013	IRF4	interferon i	528.4571328	314.4459953	0.029423166	1.672972966	up	920	358	501	414	267	64	492	1181
139	ENSG0000013	RNF144B	ring finger	172.1573576	386.9007231	0.023177009	-2.238339074	down	488	153	92	54	426	174	336	939
140	ENSG0000013	TLR2	toll like rec	669.9389316	1324.063231	0.037641814	-1.975803123	down	1373	779	383	304	1695	336	1722	2572
141	ENSG0000013	IL18BP	interleukin	150.5813921	228.3273332	0.049960183	-1.519502247	down	238	105	170	101	191	72	309	791
142	ENSG0000013	CGREF1	cell growth	0	8.264040203	0.009551566	-43.29181495	down	0	0	0	0	0	7	16	8
143	ENSG0000013	SLC3A1	solute carri	12.48628437	0	0.001370797	69.30449697	up	0	14	8	21	0	0	0	0
144	ENSG0000013	DBR1	debranchin	227.9034574	123.124549	0.040142696	1.824305349	up	221	155	250	250	60	27	188	686
145	ENSG0000013	INTS14		281.297142	165.7781107	0.009468188	1.68415147	up	512	186	254	225	114	55	186	797
146	ENSG0000013	RASGEF1B	RasGEF do	65.62286067	157.4676268	0.033487293	-2.394710094	down	142	45	63	33	269	22	144	322
147	ENSG0000013	FGD4	FYVE, Rh	62.02430879	156.1126816	0.009294226	-2.516319764	down	64	89	28	56	200	56	159	292
148	ENSG0000013	SYCP3	synaptoner	1.798838543	11.51966339	0.020610968	-6.158158945	down	4	0	0	4	9	5	12	42
149	ENSG0000013	ACVRL1	activin A r	0	7.759333175	0.01017873	-41.36095559	down	0	0	0	0	16	0	8	9
150	ENSG0000013	RB1	retinoblast	261.8654876	400.2498803	0.034827397	-1.529984387	down	476	234	222	158	292	149	401	1843
151	ENSG0000014	JDP2	Jun dimeri	68.07389846	153.6714071	0.034178989	-2.255525767	down	168	72	22	39	194	27	159	553
152	ENSG0000014	WARS	tryptophan	741.7830469	1338.217737	0.044278626	-1.802730943	down	1215	824	601	381	1693	580	780	3550
153	ENSG0000014	TCF12	transcriptic	226.9932947	379.3608233	0.028165018	-1.673240876	down	427	186	195	144	175	157	420	1992
154	ENSG0000014	SORD	sorbitol de	44.05861772	107.126931	0.024453103	-2.422620731	down	66	19	59	35	159	36	47	310
155	ENSG0000014	TLE3	transducin	329.1867485	528.8844077	0.049084855	-1.610691108	down	534	248	321	245	377	116	965	1838
156	ENSG0000014	ARRDC4	arrestin do	93.74186075	209.3765412	0.031836526	-2.228478184	down	186	114	62	32	308	60	166	479
157	ENSG0000014	IMPA2	inositol(my	170.9915684	318.5081565	0.031407351	-1.855520638	down	398	157	81	112	272	153	209	1299
158	ENSG0000014	ZNF750	zinc finger	7.023895486	0	0.046834732	38.38056253	up	0	0	4	21	0	0	0	0
159	ENSG0000014	PMAIP1	phorbol-12	65.32303855	172.1371202	0.002695131	-2.615785967	down	141	42	37	63	230	58	150	389
160	ENSG0000014	IGFBP4	insulin like	90.04928488	50.08776558	0.037992281	1.813062572	up	172	64	92	51	30	22	80	138
161	ENSG0000014	PRDM15	PR domain	176.4213158	88.78684077	0.011617632	1.966802038	up	190	152	132	211	66	32	63	495
162	ENSG0000014	SLC2A5	solute carri	41.50363295	3.956770629	3.33847E-06	9.058101084	up	58	32	24	53	4	0	4	26
163	ENSG0000014	FCGR2A	Fc fragmer	284.3475186	949.2297693	0.0001923	-3.339463831	down	445	429	178	89	817	299	1469	2587
164	ENSG0000014	S100A8	S100 calciu	2311.252148	4420.565373	0.033516047	-1.911993809	down	5858	1950	1751	725	3342	2070	6160	10336
165	ENSG0000014	C1orf35	chromoson	187.6751578	120.0193513	0.037306817	1.562199877	up	293	107	150	219	73	51	143	496
166	ENSG0000014	RHOB	ras homolo	360.1012886	976.2129571	0.00621246	-2.709431242	down	874	218	337	164	1482	150	1013	2432
167	ENSG0000014	POLR2D	polymerase	227.8897712	107.4312518	0.019731921	2.108014034	up	360	200	191	177	21	45	163	564
168	ENSG0000014	CSRNP1	cysteine an	196.8079259	456.2415914	0.017790488	-2.321269006	down	261	148	222	151	546	56	840	844
169	ENSG0000014	NFKBIZ	NFKB inhi	512.5485993	1175.943203	0.015900696	-2.294459064	down	811	409	514	354	1667	190	1749	1760
170	ENSG0000014	ADPRH	ADP-ribos	40.47246637	99.57054358	0.00847957	-2.460654475	down	69	59	21	16	94	36	94	378
171	ENSG0000014	ANKRD31	ankyrin rep	0	6.594511282	0.046072574	-35.56323054	down	0	0	0	0	0	0	24	22
172	ENSG0000014	TRIM41	tripartite m	453.0903033	292.4141884	0.042447417	1.556859327	up	752	366	386	358	130	173	308	1132
173	ENSG0000014	PHIP	pleckstrin l	1098.78988	1831.710701	0.035656191	-1.666501012	down	2338	1182	613	567	1478	690	2232	6374
174	ENSG0000014	RNF217	ring finger	36.07475948	112.1129422	6.59066E-05	-3.080280537	down	73	32	32	16	94	48	104	423
175	ENSG0000014	CREB5	cAMP resp	170.6153452	463.7184112	0.031305039	-2.71671887	down	497	46	152	98	84	101	1319	1365
176	ENSG0000014	DOCK5	dedicator c	336.369789	710.3341422	0.011542143	-2.113558126	down	736	358	144	211	561	146	1034	3116
177	ENSG0000014	PXDNL	peroxidasi	0	7.343965608	0.003414231	-37.76147997	down	0	0	0	0	6	6	5	8
178	ENSG0000014	ASTN2	astrotactin	13.36405904	3.324848706	0.030855017	3.876566168	up	25	12	9	10	4	0	7	7
179	ENSG0000014	TCF7L2	transcriptic	75.94828879	160.4368966	0.041652236	-2.123803223	down	88	103	24	80	187	32	125	752
180	ENSG0000014	ADM	adrenomed	16.31527802	71.04711416	0.045901941	-4.315942296	down	62	0	12	8	71	4	193	48
181	ENSG0000014	AASDHPPT	aminoadip	392.7378378	238.2710461	0.003747815	1.642806486	up	629	319	311	347	213	74	294	857
182	ENSG0000015	PTS	6-pyruvoyl	26.0980073	52.06339296	0.045837968	-2.016627439	down	21	38	20	18	40	19	76	157
183	ENSG0000015	ANK3	ankyrin 3,	182.4613947	104.7207222	0.046910581	1.730824422	up	134	206	166	172	50	44	132	484
184	ENSG0000015	ACSL1	acyl-CoA s	463.5641564	1123.856921	0.018483181	-2.423637881	down	1337	338	258	202	956	173	2419	2620
185	ENSG0000015	ZNF773	zinc finger	80.93780003	24.34624342	0.010823283	3.174888779	up	132	78	79	41	7	5	16	231
186	ENSG0000015	NR4A2	nuclear rec	24.3363898	61.73281171	0.019669112	-2.505735265	down	28	14	38	15	38	39	84	102
187	ENSG0000015	C4orf19	chromoson	8.725216877	0.267813486	0.007204009	17.09719474	up	8	12	4	9	0	0	0	4
188	ENSG0000015	EME1	essential m	7.566823136	0.267813486	0.041198766	15.67203424	up	0	9	5	12	0	0	0	4
189	ENSG0000015	KLF10	Kruppel-lil	329.2531024	989.1705688	0.004059985	-3.003574474	down	466	451	288	98	1543	314	744	1663
190	ENSG0000015	FCHO2	FCH doma	82.60427908	185.2887114	0.011355304	-2.22732852	down	157	103	38	47	140	100	239	373
191	ENSG0000015	PALM2-AKAP2	PALM2-A	30.29879189	0	0.029200647	164.3239316	up	0	76	0	25	0	0	0	0
192	ENSG0000015	GRHL3	grainyhead	0	9.151435843	0.011609239	-47.67480396	down	0	0	0	0	12	8	0	2
193	ENSG0000015	AHCYL2	adenosylho	56.71003467	121.4918226	0.031274231	-2.134365142	down	103	46	59	28	97	62	45	607
194	ENSG0000015	APOA2	apolipopro	0	5.913141074	0.022612042	-31.30751433	down	0	0	0	0	0	4	8	26
195	ENSG0000015	EPB41	erythrocyte	2340.816685	5365.745299	0.036722481	-2.291478722	down	2550	2079	1949	2507	4260	4976	2533	4594
196	ENSG0000015	CELF3	CUGBP, E	0	13.94710783	0.000199874	-71.74729665	down	0	0	0	0	4	16	9	12
197	ENSG0000015	TNFRSF13C	tumor necr	171.5488357	81.96186773	0.044929598	2.07009671	up	362	115	64	200	82	13	66	477
198	ENSG0000016	RSPH1	radial spok	2.914681478	16.689105	0.046702507	-5.538013008	down	4	0	0	8	4	17	13	31
199	ENSG0000016	ZNF208	zinc finger	22.84355658	6.562810817	0.041720769	3.20195124	up	36	34	10	12	6	0	8	42
200	ENSG0000016	FAM189B	family witl	111.9240068	174.4572458	0.045049394	-1.542520143	down	160	92	91	107	138	90	204	425
201	ENSG0000016	IER2	immediate	1272.643637	2382.00447	0.025396214	-1.872117836	down	1672	921	1374	1090	2560	508	4123	4737
202	ENSG0000016	ORAI2	ORAI calc	181.8125335	647.602087	0.0008936	-3.568971974	down	116	81	305	173	849	98	595	2555
203	ENSG0000016	BICDL2		0	7.845553981	0.033031762	-42.17186243	down	0	0	0	0	0	0	33	12
204	ENSG0000016	TMCO4	transmemb	193.6062639	124.990313	0.044718819	1.536977915	up	270	125	162	222	69	46	173	541
205	ENSG0000016	B3GALT2	Beta-1,3-g	55.72495917	22.39463225	0.044782796	2.418438962	up	126	32	32	54	4	8	24	164
206	ENSG0000016	ZNF326	zinc finger	126.8625228	268.7119481	0.001527355	-2.125771228	down	185	100	101	126	107	102	423	1182
207	ENSG0000016	ARPC5	actin relate	1371.338666	2848.790755	0.016177355	-2.077401116	down	1163	1927	492	1570	2400	1394	2485	9588
208	ENSG0000016	ATF3	activating t	11.59015907	57.11851718	0.001871718	-4.911153284	down	21	12	11	4	101	8	57	84
209	ENSG0000016	PQLC3	PQ loop re	235.453489	375.4098145	0.023610035	-1.588604309	down	364	198	188	207	321	185	268	1417
210	ENSG0000016	C2orf48	chromoson	7.427240083	0	0.011057537	41.08462042	up	9	4	16	0	0	0	0	0
211	ENSG0000016	S100A9	S100 calciu	6308.664101	12602.68496	0.037205389	-1.997548935	down	16304	5228	5183	1469	9605	4689	20068	32246
212	ENSG0000016	HIPK1	homeodom	994.817638	1731.096039	0.033924489	-1.740623292	down	853	691	1495	722	863	829	2127	7055
213	ENSG0000016	SLC22A15	solute carri	66.10069158	186.824779	0.011216564	-2.826385636	down	131	105	28	12	141	60	298	571
214	ENSG0000016	SPICE1	spindle anc	63.16868825	113.5505492	0.011147031	-1.804810856	down	99	38	68	53	99	36	128	453
215	ENSG0000016	CDS1	CDP-diacy	0.682995608	10.53103957	0.014749137	-12.63230073	down	4	0	0	0	8	9	4	21
216	ENSG0000016	CCNL1	cyclin L1	1408.767613	2895.082808	0.002090823	-2.054948822	down	1847	1083	1209	1465	3341	885	3701	6302
217	ENSG0000016	CXCL3	C-X-C mot	6.838112625	28.40179518	0.006961643	-3.977464256	down	11	6	4	7	13	26	20	55
218	ENSG0000016	KIF15	kinesin fan	3.28310469	23.23373397	0.019524391	-7.20526269	down	0	5	6	0	24	14	27	10
219	ENSG0000016	DNALI1	dynein axo	0	5.537671359	0.032804701	-28.87090653	down	0	0	0	0	0	5	9	8
220	ENSG0000016	PIGX	phosphatid	76.5124228	145.5498565	0.022892289	-1.887015292	down	118	88	50	53	69	93	138	527
221	ENSG0000016	MELTF	melanotrar	8.892400886	0.535626973	0.022262799	10.86230915	up	8	8	4	14	0	0	0	8
222	ENSG0000016	CEP44	centrosom	154.1238182	97.29054915	0.039471234	1.584690108	up	250	100	122	162	50	42	145	350
223	ENSG0000016	FAM198B	family witl	182.6149172	509.8381189	0.030161933	-2.786574328	down	663	158	22	52	599	145	443	1821
224	ENSG0000016	SHROOM1	shroom far	21.35415339	51.73602214	0.02448236	-2.431966264	down	20	15	18	29	69	16	29	182
225	ENSG0000016	UQCRQ	ubiquinol-c	210.3195556	380.0144559	0.024439363	-1.793947116	down	416	182	136	157	235	259	338	1019
226	ENSG0000016	FABP5	fatty acid b	49.1818288	92.76105607	0.048891638	-1.853417279	down	111	33	46	24	80	51	75	270
227	ENSG0000016	HDX	highly dive	3.406001349	25.35993725	0.005970168	-7.27907401	down	8	0	7	0	36	8	16	71
228	ENSG0000016	TRMT10B	tRNA metl	164.4772798	80.89388756	0.035978506	2.014125213	up	142	110	194	179	69	14	162	212
229	ENSG0000016	COMTD1	catechol-O	83.87448115	40.20117183	0.033691047	2.023338027	up	80	68	87	86	32	9	31	256
230	ENSG0000016	ZCCHC18	zinc finger	61.04817412	26.05075079	0.034170581	2.281755866	up	59	97	45	29	16	8	27	148
231	ENSG0000016	PIP4K2C	phosphatid	305.748138	172.0351083	0.013138381	1.756049149	up	363	296	295	240	117	48	184	946
232	ENSG0000016	CCDC103	coiled-coil	0	7.804709043	0.006244459	-42.28850834	down	0	0	0	0	8	0	10	44
233	ENSG0000016	PRRT2	proline ricl	7.575418539	31.63729052	0.003320952	-4.116157396	down	8	9	8	4	28	20	32	44
234	ENSG0000016	PROCA1	protein inte	59.82104223	21.75794001	0.004653687	2.669200248	up	58	37	50	86	16	5	35	86
235	ENSG0000016	ULK4	unc-51 like	22.06873627	58.26848172	0.007864515	-2.589165767	down	26	13	20	28	39	41	40	167
236	ENSG0000016	NT5DC2	5'-nucleoti	59.4367169	24.22126164	0.027558932	2.489576772	up	80	38	96	22	30	9	23	53
237	ENSG0000016	MGAT2	mannosyl (	484.9404021	310.1781958	0.02838169	1.553157362	up	698	416	458	375	211	98	306	1682
238	ENSG0000016	ADAM9	ADAM me	90.27339845	211.1249861	0.008691424	-2.318228856	down	202	94	57	37	166	131	155	609

239	ENSG0000016	AXIN2	axin 2	203.6813871	108.1246768	0.025628682	1.85794208	up	267	146	181	217	42	29	194	516
240	ENSG0000016	SEMA4C	semaphori	396.6984482	223.5333807	0.045565731	1.765054116	up	290	296	311	594	124	74	260	1198
241	ENSG0000016	SLC49A3		33.11264719	67.55070947	0.028201375	-2.037481036	down	68	30	24	19	77	17	68	244
242	ENSG0000016	CXCL10	C-X-C mot	8.743426456	37.57309237	0.035746421	-4.235768298	down	30	8	4	0	39	13	6	224
243	ENSG0000016	NPIPB3	nuclear po	228.4916139	120.313165	0.012502848	1.883079205	up	245	186	257	196	103	28	199	381
244	ENSG0000016	RNASE2	ribonuclea	95.84313939	217.7797304	0.017151417	-2.25042232	down	264	80	44	48	196	126	186	503
245	ENSG0000016	CXCL8	C-X-C mot	12.579606	115.3432426	0.002082511	-9.228776016	down	8	9	29	0	143	13	265	31
246	ENSG0000016	TM4SF1	transmemb	0.682995608	11.7731911	0.005225064	-14.13596497	down	4	0	0	0	13	5	9	35
247	ENSG0000016	NLGN2	neuroligin	77.77059911	42.14235776	0.027823244	1.824864327	up	120	42	70	86	22	16	60	179
248	ENSG0000017	ZNF212	zinc finger	188.2991925	100.2231483	0.008258218	1.871761161	up	180	120	197	227	66	40	128	385
249	ENSG0000017	CMTM8	CKLF like	16.50819275	35.14117234	0.047314938	-2.163107691	down	28	15	12	13	37	5	46	144
250	ENSG0000017	FOS	FBJ murin	508.1353624	2752.789773	0.000749948	-5.416790926	down	985	339	686	129	2570	609	6790	794
251	ENSG0000017	ZNF804A	zinc finger	10.2566957	28.87095791	0.026872641	-2.734512473	down	20	13	5	5	28	16	24	65
252	ENSG0000017	CD14	CD14 mol	801.3344422	1851.776514	0.026514349	-2.310072442	down	1854	1140	281	190	1726	723	2378	4643
253	ENSG0000017	MZB1	marginal z	290.777543	96.78069954	0.046416469	3.001835313	up	250	73	448	341	210	5	33	226
254	ENSG0000017	GPR27	G protein-	17.61295381	39.02677455	0.028090335	-2.191208139	down	33	14	12	15	16	19	62	129
255	ENSG0000017	TSEN34	tRNA splic	193.266415	366.3731583	0.011241244	-1.884446186	down	421	157	143	113	338	185	319	1033
256	ENSG0000017	S1PR1	sphingosin	1405.726189	662.1967144	0.007769425	2.124084712	up	2122	1729	1188	597	279	373	634	3017
257	ENSG0000017	ALK	anaplastic	8.186122967	0	0.028115937	44.97116171	up	12	0	0	22	0	0	0	0
258	ENSG0000017	JUNB	jun B prot	1299.028148	3907.198057	0.000535486	-3.007747784	down	2192	852	1401	914	3681	903	8093	5525
259	ENSG0000017	NPTX1	neuronal p	13.42933878	1.60261387	0.009794756	7.069939142	up	12	9	20	10	0	0	5	8
260	ENSG0000017	ANO5	anoctamin	4.239200312	27.05464827	0.021716338	-6.135793107	down	18	0	4	0	44	4	29	51
261	ENSG0000017	NHLH1	nescient he	3.231050068	16.1319662	0.026495588	-4.949406482	down	4	0	3	6	28	4	9	33
262	ENSG0000017	CXCR6	C-X-C mot	163.6731231	77.68121942	0.029690291	2.098185005	up	87	95	178	243	56	32	63	380
263	ENSG0000017	CLEC7A	C-type lect	461.6538368	1048.281219	0.022041271	-2.269772353	down	1249	440	164	235	1302	194	1275	3184
264	ENSG0000017	CHCHD1	coiled-coil	99.82898747	181.2931436	0.045439711	-1.793167832	down	228	53	67	90	107	121	153	562
265	ENSG0000017	SYT12	synaptotag	6.829885861	0	0.003793879	37.92453927	up	2	9	8	5	0	0	0	0
266	ENSG0000017	SFT2D3	SFT2 dom	0	12.32587188	0.039310862	-64.66151934	down	0	0	0	0	0	20	0	0
267	ENSG0000017	HOXB2	homeobox	234.276093	141.2633226	0.045094516	1.640106068	up	258	137	250	270	98	40	161	730
268	ENSG0000017	TLR1	toll like re	268.7727488	425.2089986	0.043358315	-1.585255102	down	507	267	188	163	332	115	600	1691
269	ENSG0000017	TLR6	toll like re	91.31207436	288.27662	0.000282155	-3.167734581	down	159	112	40	65	224	50	480	1176
270	ENSG0000017	PMS2P6	PMS1 hom	2.375049874	11.62941828	0.024119237	-4.759078163	down	2	2	2	3	6	10	6	32
271	ENSG0000017	GOLT1A	golgi trans	0	8.065056846	0.038150527	-41.89971899	down	0	0	0	0	0	12	0	10
272	ENSG0000017	SH3PXD2B	SH3 and P	7.494742108	26.18505944	0.043973202	-3.407123774	down	22	4	0	9	34	8	24	68
273	ENSG0000017	AMZ1	archaelysi	1.165720076	17.7789427	0.007174533	-15.5825034	down	0	0	4	0	8	20	4	28
274	ENSG0000017	UBE2C	ubiquitin c	2.048986824	21.34156255	0.015375662	-9.429081107	down	12	0	0	0	12	16	29	18
275	ENSG0000017	FAM131A	family wit	61.02227721	109.6597354	0.021798947	-1.791301672	down	72	53	53	61	118	42	112	294
276	ENSG0000017	PHLDB3	pleckstrin	98.75274292	43.33744073	0.029971533	2.233325268	up	146	30	126	100	30	7	76	188
277	ENSG0000017	KCNA2	potassium	81.1204613	24.59616932	0.020490896	3.347600017	up	26	64	100	100	4	16	52	34
278	ENSG0000017	NR2C2	nuclear re	197.1489375	654.5053559	0.005475519	-3.32725728	down	304	168	175	153	127	52	1206	4807
279	ENSG0000017	JUN	jun proto-	158.9969817	953.2671882	6.32743E-06	-5.989527176	down	310	142	134	84	1135	286	1652	566
280	ENSG0000017	CCDC184	coiled-coil	0	5.336811244	0.039324626	-27.76662223	down	0	0	0	0	0	5	9	5
281	ENSG0000017	AQP11	aquaporin	21.28965697	4.343435058	0.049746525	4.392846768	up	7	16	32	21	0	0	10	33
282	ENSG0000017	ZNF223	zinc finger	2.538590786	23.85641458	0.004747082	-9.69591821	down	0	1	0	8	28	4	27	91
283	ENSG0000017	ALOXE3	arachidona	8.118693241	0.736487088	0.047135276	7.822578302	up	12	4	8	9	0	0	0	11
284	ENSG0000017	LBX2	ladybird h	16.89825849	4.835434676	0.034057656	3.216403801	up	26	9	17	17	4	0	10	20
285	ENSG0000017	PSTK	phosphose	21.78613244	45.49469196	0.030577249	-2.073871773	down	24	16	18	27	22	27	43	182
286	ENSG0000018	SOCS4	suppressor	433.8108748	275.7443955	0.038120853	1.563588224	up	563	329	437	392	243	62	393	1059
287	ENSG0000018	HIGD1A	HIG1 hyp	355.2156771	575.8278743	0.010421266	-1.619126968	down	402	258	368	359	508	257	569	1837
288	ENSG0000018	AC105219.1		14.13724754	2.19265695	0.040513196	5.429805986	up	8	16	4	24	0	0	4	20
289	ENSG0000018	FRAT2	frequently	263.4401346	480.2298149	0.015350519	-1.820057103	down	622	204	206	124	350	178	623	1768
290	ENSG0000018	RELL1	RELT like	20.27931536	55.05095254	0.016016514	-2.643896524	down	46	20	12	10	51	35	30	145
291	ENSG0000018	IBA57	IBA57 hon	135.7371356	85.61940428	0.029062154	1.601424369	up	161	114	148	108	62	42	95	274
292	ENSG0000018	AC138028.1		0	6.422954424	0.023775478	-33.2679247	down	0	0	0	0	0	7	8	6
293	ENSG0000018	TMEM198B	transmemb	236.7852752	115.7581996	0.00657459	2.032214912	up	198	139	284	278	89	38	141	477
294	ENSG0000018	ADGRG3	adhesion G	64.17576952	161.1823018	0.036853823	-2.504938578	down	181	24	64	26	52	48	422	356
295	ENSG0000018	SPATA13	spermatog	685.3677857	1352.611367	0.008080052	-1.974606116	down	683	662	615	668	803	481	2712	3046
296	ENSG0000018	HIST2H3D	histone clu	0.512246706	16.78128198	0.001043632	-25.73684201	down	3	0	0	0	17	13	8	19
297	ENSG0000018	RP1L1	retinitis pi	0	7.503415175	0.016752238	-39.15952286	down	0	0	0	0	12	5	0	5
298	ENSG0000018	CCR4	C-C motif	388.351816	229.0146124	0.008799439	1.69433911	up	473	355	404	290	120	108	256	1000
299	ENSG0000018	NCF4-AS1		0	5.188570302	0.029452533	-28.05151545	down	0	0	0	0	5	0	11	17
300	ENSG0000018	BRD7P2	bromodom	12.48312028	31.00399511	0.039084206	-2.424976779	down	23	14	6	9	16	18	48	63
301	ENSG0000018	LRTOMT	leucine ric	27.19101477	51.20439539	0.043026198	-1.901020502	down	29	26	25	25	22	22	82	189
302	ENSG0000018	SNRNP35	small nucl	196.0364768	124.7383287	0.028366645	1.552823221	up	266	174	190	150	99	35	167	505
303	ENSG0000018	SOCS3	suppressor	175.0231762	887.2961918	0.000421927	-5.073993418	down	200	136	232	113	724	84	2543	691
304	ENSG0000018	PRKN		1.115842935	12.60410648	0.031725718	-11.27185193	down	0	0	0	4	12	12	4	4
305	ENSG0000018	TMLHE	trimethylly	46.88411152	101.6930245	0.036490723	-2.168154402	down	83	70	28	11	72	43	111	403
306	ENSG0000018	ZNF284	zinc finger	0	26.58894798	4.2283E-07	-142.6058387	down	0	0	0	0	10	5	76	58
307	ENSG0000018	BCL9L	B-cell CLL	2439.236673	1611.61901	0.044687793	1.511073817	up	2838	1974	2129	2611	1007	456	1878	8765
308	ENSG0000018	1-Mar	mitochond	31.16689925	99.25213357	0.031790695	-3.175423314	down	81	28	30	0	97	28	108	387
309	ENSG0000018	BLOC1S4	biogenesis	169.2243089	105.0991893	0.039843543	1.600881106	up	260	160	122	144	57	46	98	544
310	ENSG0000018	MIR22HG	MIR22 hos	45.27733507	112.1390216	0.04256607	-2.487231181	down	42	40	58	32	200	10	93	269
311	ENSG0000018	HS6ST1P1	heparan su	10.47676246	0.937347203	0.030622601	8.206493557	up	20	10	7	7	0	0	0	14
312	ENSG0000018	MORN2	MORN rep	4.365854589	22.7472747	0.040810262	-5.224782424	down	4	12	0	0	16	17	10	70
313	ENSG0000018	NPIPP1	nuclear po	76.89830888	18.89769248	0.002811841	3.967075927	up	56	94	44	92	4	9	11	144
314	ENSG0000018	LINC00910	long interg	57.27367214	17.50214813	0.004099093	3.152121158	up	65	44	25	91	9	4	31	80
315	ENSG0000018	RPSAP47	ribosomal	4.041297297	0.066953372	0.033137677	15.39532414	up	8	5	2	2	0	0	0	1
316	ENSG0000018	ZNF33A	zinc finger	568.2495772	340.2712807	0.011974807	1.671246279	up	665	526	454	577	358	119	372	980
317	ENSG0000019	ATP2A1	ATPase sa	0.836882201	11.89101513	0.024606846	-14.73356224	down	0	0	0	3	22	0	9	37
318	ENSG0000019	MME	membrane	30.09622459	117.4486112	0.002725054	-3.883840498	down	70	25	12	25	54	36	321	125
319	ENSG0000019	XRCC2	X-ray repa	26.21339289	10.84224793	0.030384031	2.416827964	up	23	25	31	20	10	4	12	36
320	ENSG0000019	SLC39A10	solute carr	316.2184293	202.83729	0.041491804	1.54682727	up	443	262	272	290	113	75	194	1146
321	ENSG0000019	GATA3-AS1	GATA3 an	8.989570987	0	0.005867937	49.66615298	up	0	10	5	16	0	0	0	0
322	ENSG0000019	PELI1	pellino E3	474.897607	872.7738539	0.037554298	-1.838758996	down	934	245	406	437	818	148	1570	2508
323	ENSG0000019	ZNF517	zinc finger	82.98242037	38.87209507	0.037960789	2.075829508	up	95	66	83	80	40	3	56	171
324	ENSG0000019	ZNF841	zinc finger	153.0421186	66.30997737	0.001965911	2.285925434	up	187	122	88	208	34	26	86	304
325	ENSG0000019	ZNF615	zinc finger	114.6155356	70.20832133	0.026129779	1.648506477	up	188	88	89	106	46	36	60	292
326	ENSG0000019	AL157838.1		5.170359444	24.55702781	0.044905535	-4.775015564	down	11	8	0	3	27	0	45	86
327	ENSG0000019	ZNF460	zinc finger	21.68899658	48.53147108	0.028251109	-2.245967388	down	32	28	8	19	28	18	84	149
328	ENSG0000019	ZNF544	zinc finger	189.171819	108.4594775	0.02689955	1.728085269	up	317	168	185	106	97	26	145	425
329	ENSG0000019	PIM3	Pim-3 prot	361.3799578	670.8201061	0.020821747	-1.857098679	down	565	207	333	374	755	145	992	1682
330	ENSG0000019	ZNF627	zinc finger	97.15634028	48.38267923	0.035041112	1.962906958	up	161	113	54	69	29	14	44	306
331	ENSG0000019	C6orf89	chromoson	258.5644031	495.231327	0.042552088	-1.922118351	down	280	373	182	155	406	109	363	3171
332	ENSG0000019	F5	coagulatio	160.2113833	247.6277834	0.038671752	-1.55419363	down	255	138	121	140	165	68	407	936
333	ENSG0000019	GK	glycerol ki	117.4245793	218.4184596	0.049089313	-1.861010721	down	263	71	86	92	219	37	377	606
334	ENSG0000019	CES1	carboxyles	152.6362156	333.7446096	0.025489222	-2.187445648	down	367	179	58	65	135	120	424	1842
335	ENSG0000019	CAPZA2	capping ac	550.6067606	1069.5283	0.007997305	-1.93822639	down	1128	494	310	416	991	580	791	3073
336	ENSG0000020	EFCAB2	EF-hand c	27.67802657	69.28207829	0.032399279	-2.457348429	down	46	27	8	33	27	58	51	201
337	ENSG0000020	LINC00862	long interg	0	6.007860994	0.022433891	-32.56474889	down	0	0	0	0	2	0	19	19
338	ENSG0000020	HIST2H3PS2	histone clu	0.341497804	5.899729553	0.032657033	-12.62948921	down	2	0	0	0	2	4	6	22
339	ENSG0000020	ARRDC1-AS1	ARRDC1	137.7654381	69.9731348	0.007838099	1.941043717	up	142	96	119	177	53	22	80	318
340	ENSG0000020	LINC01270	long interg	1.798838543	15.97988858	0.025886269	-8.78187591	down	4	0	0	4	10	0	47	38
341	ENSG0000020	SLC12A5-AS1		0	4.62116304	0.042563732	-24.89706827	down	0	0	0	0	6	0	8	13
342	ENSG0000020	CXorf65	chromoson	42.04275722	11.20014331	0.027935787	3.631875856	up	23	8	41	85	2	3	24	53
343	ENSG0000020	NOTCH4	notch 4	8.401146569	31.54124935	0.031920008	-3.840626595	down	0	4	16	9	29	8	37	132
344	ENSG0000020	INSYN2B		0.874290057	15.10518246	0.006136138	-18.03049181	down	0	0	3	0	16	9	18	4
345	ENSG0000020	AC134312.1		0	6.938398204	0.013195602	-37.07871873	down	0	0	0	0	13	0	8	12
346	ENSG0000020	SMN2	survival of	77.54415923	171.1003198	0.009118016	-2.197346496	down	138	48	38	101	159	76	102	722
347	ENSG0000020	RNU6-878P	RNA, U6 s	0.278960734	4.192938988	0.045859483	-13.02279567	down	0	0	0	1	3	3	4	7
348	ENSG0000021	IGKC	immunogl	6996.661769	2869.657633	0.042802419	2.438038587	up	8218	1420	9503	8561	5136	364	1926	7244
349	ENSG0000021	IGKV5-2	immunogl	36.45249502	4.666434675	0.011947724	7.469863534	up	26	20	84	5	8	0	0	29
350	ENSG0000021	IGKV6-21	immunogl	21.31169344	2.785908765	0.031239741	7.714494126	up	18	2	4	59	7	0	0	6
351	ENSG0000021	IGLV6-57	immunogl	81.17879566	29.68725127	0.034868323	2.756370012	up	88	14	106	111	45	10	13	81
352	ENSG0000021	IGLV1-40	immunogl	463.0855464	150.9004236	0.044102162	3.06784693	up	306	86	1099	230	279	14	169	167
353	ENSG0000021	IGLV1-36	immunogl	65.8479182	6.654734484	0.00277647	9.867259409	up	78	10	141	30	16	0	0	18
354	ENSG0000021	IGLV3-10	immunogl	31.9372398	8.241418298	0.042513243	3.799186207	up	12	11	68	24	14	0	10	20
355	ENSG0000021	IGLV3-1	immunogl	112.3683653	30.01836845	0.023254145	3.75843279	up	165	46	206	36	64	5	4	64
356	ENSG0000021	TRAV26-2	T cell rece	23.45057102	6.048962896	0.037320454	3.565325427	up	33	17	26	18	0	2	5	56
357	ENSG0000021	IGHV3-23	immunogl	541.4438387	200.4111551	0.028812358	2.701008333	up	886	153	633	569	387	18	149	384
358	ENSG0000021	IGHV3-49	immunogl	77.40381856	9.321206341	0.003157227	7.995104498	up	100	14	64	134	14	0	0	68

359	ENSG0000021	IGHV3-53	immunogl	46.25365843	6.643071476	0.010938577	6.845466565	up	72	18	88	10	14	0	0	28
360	ENSG0000021	IGHV4-61	immunogl	165.4630272	30.68194242	0.013051228	5.362004489	up	22	36	270	258	59	0	22	88
361	ENSG0000021	POU5F1B	POU class	16.14761709	1.021795087	0.002244257	18.21249872	up	12	20	12	16	3	0	0	0
362	ENSG0000021	NOC2LP1	NOC2 like	8.46390852	0.133906743	0.006003509	24.48322087	up	4	18	2	6	0	0	0	2
363	ENSG0000021	RPL12P38	ribosomal	8.962783432	27.97380695	0.029925771	-2.986431107	down	15	5	10	7	22	23	17	40
364	ENSG0000021	AC239799.1		0	14.67324306	0.008468399	-76.90152699	down	0	0	0	0	0	20	11	0
365	ENSG0000021	SULT1A4	sulfotransf	27.25304327	130.4718142	0.000776797	-4.73052756	down	48	10	7	50	119	96	73	227
366	ENSG0000021	AL356317.1		6.057987058	0	0.005410729	33.66923149	up	3	7	4	8	0	0	0	0
367	ENSG0000021	GNAQP1	G protein s	6.775884544	0	0.0237479	37.23059019	up	0	2	2	20	0	0	0	0
368	ENSG0000021	OR1X5P	olfactory r	5.920022203	0	0.023086817	32.65047734	up	7	4	12	0	0	0	0	0
369	ENSG0000021	NPIPA8	nuclear po	174.6680241	104.7851839	0.043458027	1.670482761	up	171	115	245	139	80	46	121	349
370	ENSG0000021	DNAJA1P3	DnaJ heat	0	12.32587188	0.003310862	-64.66151934	down	0	0	0	0	0	20	0	0
371	ENSG0000021	AL645728.1		0	4.560041172	0.048431825	-24.44377267	down	0	0	0	0	7	0	8	7
372	ENSG0000021	TNFRSF25	tumor necr	627.2824314	388.2248742	0.012776967	1.611463149	up	635	407	586	800	279	134	495	1568
373	ENSG0000021	SLC35E2A		688.1826894	448.3071368	0.045763923	1.538601049	up	1005	796	516	437	400	197	516	1203
374	ENSG0000021	MMP23A	matrix met	2.048986824	25.76256291	0.031262011	-11.70356733	down	12	0	0	0	0	31	13	58
375	ENSG0000021	AL158066.1		42.70714133	8.227926952	0.042081537	5.139680319	up	33	13	58	58	11	0	21	0
376	ENSG0000021	RSL24D1P1	ribosomal	0	7.166917296	0.018219989	-37.10485431	down	0	0	0	0	7	7	0	7
377	ENSG0000021	FAM228B	family wit	35.22899352	64.25109533	0.046852533	-1.86258118	down	42	35	24	37	54	12	88	294
378	ENSG0000022	HIST2H2BD	histone clu	75.70118429	161.202863	0.004890103	-2.133560423	down	91	93	75	35	138	63	191	517
379	ENSG0000022	ZNF844	zinc finger	94.72247285	44.23132428	0.043000182	2.071628926	up	104	64	78	124	18	8	61	301
380	ENSG0000022	AC034228.1		6.129619164	0	0.022576954	33.82505595	up	6	3	0	15	0	0	0	0
381	ENSG0000022	AC005076.1		0	11.23842089	0.000370605	-59.27681043	down	0	0	0	0	20	3	8	13
382	ENSG0000022	MTCO1P11	MT-CO1 p	0	8.835291061	0.00464341	-46.24498563	down	0	0	0	0	16	4	4	1
383	ENSG0000022	AL161785.1		0	16.56328489	3.67865E-06	-87.67696094	down	0	0	0	0	7	9	21	62
384	ENSG0000022	AP001476.1		0	7.663336616	0.047635002	-39.73595162	down	0	0	0	0	0	12	0	4
385	ENSG0000022	RPL32P1	ribosomal	1.045038959	9.05633437	0.04849016	-8.354395682	down	1	0	3	0	3	11	4	6
386	ENSG0000022	AC011447.1		10.84031157	1.096328475	0.014017405	7.591534807	up	11	5	14	12	0	0	2	10
387	ENSG0000022	MRPL20-AS1		133.5116017	71.53437263	0.010712133	1.868864939	up	156	74	150	145	43	33	85	275
388	ENSG0000022	RBMS1P1	RNA bindi	38.40443177	93.56857253	0.037475314	-2.408384335	down	93	52	12	11	64	56	104	225
389	ENSG0000022	BOLA3-AS1	BOLA3 an	14.24436032	30.61845133	0.04898357	-2.095252139	down	25	14	8	12	27	16	25	93
390	ENSG0000022	RPL35P5	ribosomal	6.492859284	0.40172023	0.044409158	10.04120527	up	6	2	9	8	0	0	0	6
391	ENSG0000022	AL139246.2		1.115842935	9.145087623	0.035450932	-8.443922831	down	0	0	0	4	6	4	12	31
392	ENSG0000022	NRIR	negative re	34.25395582	84.1614782	0.013143606	-2.442698595	down	44	32	37	22	89	35	118	106
393	ENSG0000022	PHF2P2	PHD finge	9.813450033	0.267813486	0.018469036	20.17966929	up	7	0	20	10	0	0	0	4
394	ENSG0000022	TEX22	testis expr	0	8.450375145	0.010743731	-44.36467328	down	0	0	0	0	16	4	0	8
395	ENSG0000022	LINC00381	long interg	13.44650417	1.255309747	0.003421213	8.892211668	up	20	16	8	10	0	0	4	6
396	ENSG0000022	CYP2D8P	ccytochron	0	7.514793946	0.002492424	-39.16197895	down	0	0	0	0	3	6	4	29
397	ENSG0000022	RPS2P36	ribosomal	0.341497804	8.719236371	0.020018271	-19.26552878	down	2	0	0	0	12	0	17	15
398	ENSG0000022	LINC01132	long interg	5.561215248	0	0.030793458	30.49798858	up	0	8	3	8	0	0	0	0
399	ENSG0000022	SLC9A7P1	solute carr	2.606908291	14.26954231	0.041002839	-5.089684183	down	12	0	0	2	8	7	16	57
400	ENSG0000022	TRIM60P18	tripartite m	26.11671444	4.469071585	0.024237422	5.095783162	up	61	17	13	24	0	0	4	54
401	ENSG0000022	AL096678.1		0	7.588588241	0.015678215	-39.26097685	down	0	0	0	0	0	9	8	5
402	ENSG0000022	AP001469.2		1.729652765	11.8795532	0.030845309	-6.908660108	down	0	2	0	4	8	10	9	16
403	ENSG0000022	MIR4432HG	MIR4432 l	5.045922789	45.7352549	4.73829E-05	-8.857172958	down	9	4	4	4	69	23	18	63
404	ENSG0000022	MRPL45P2	mitochond	0.874290057	19.6854073	0.000218989	-24.64514262	down	0	0	3	0	8	8	25	100
405	ENSG0000022	AC072022.1		0	5.570173251	0.02381919	-29.97203905	down	0	0	0	0	8	0	8	17
406	ENSG0000022	AL121985.1		7.112002708	22.57616547	0.048442765	-3.127900705	down	16	7	0	8	26	7	24	64
407	ENSG0000022	AC233976.1		0	9.96288311	0.002231228	-53.67764135	down	0	0	0	0	10	0	21	31
408	ENSG0000022	AL353194.1		4.613306248	0	0.049223474	25.30408331	up	3	7	0	7	0	0	0	0
409	ENSG0000022	LINC01150	long interg	0	7.239820317	0.037650003	-38.80695081	down	0	0	0	0	13	0	0	42
410	ENSG0000022	AC013460.1		6.461718848	0	0.020075601	35.60131838	up	4	0	16	4	0	0	0	0
411	ENSG0000023	PA2G4P4	proliferatic	14.21754061	54.7992457	0.005865666	-3.786511645	down	15	8	22	10	29	48	54	57
412	ENSG0000023	AC005722.1		0	8.652008862	0.03818742	-45.00394404	down	0	0	0	0	0	13	3	0
413	ENSG0000023	LINC01001	long interg	52.85049202	148.711831	0.015469993	-2.830590016	down	65	62	55	24	88	18	410	301
414	ENSG0000023	MEIS1-AS2	MEIS1 ant	1.677966782	13.04212736	0.016034133	-7.528911364	down	3	0	4	0	14	8	5	34
415	ENSG0000023	AC125232.1		75.35803007	18.90374231	0.008104301	3.874173806	up	65	28	57	140	26	1	10	109
416	ENSG0000023	HNRNPA1P9	heterogene	0	8.849323531	0.011300359	-46.05544918	down	0	0	0	0	9	8	4	0
417	ENSG0000023	AC007364.1		11.13098368	0.267813486	0.013632168	23.1044215	up	45	0	8	4	0	0	0	4
418	ENSG0000023	IGHV3-43	immunogl	51.55700996	8.279109916	0.028958523	6.182038703	up	26	4	56	106	19	0	0	27
419	ENSG0000023	DFFBP1	DNA fragr	12.59370614	2.163315372	0.039753708	5.101088064	up	8	8	10	21	0	0	7	10
420	ENSG0000023	AL683842.1		0	4.516899603	0.026640722	-23.87361134	down	0	0	0	0	8	1	3	8
421	ENSG0000023	AC112198.2		0	9.08078795	0.00147727	-46.92344008	down	0	0	0	0	8	7	8	5
422	ENSG0000023	LINC00945	long interg	0	6.495652558	0.032173264	-33.32037942	down	0	0	0	0	2	9	0	4
423	ENSG0000023	RERE-AS1		5.562602661	17.55730286	0.030892444	-3.148928222	down	5	7	4	5	11	9	29	31
424	ENSG0000023	SNHG15	small nucl	143.7969026	264.8480281	0.021394116	-1.844802462	down	150	115	115	177	307	73	326	683
425	ENSG0000023	LINC01271	long interg	0	9.702913049	0.007263008	-50.65790157	down	0	0	0	0	9	8	8	0
426	ENSG0000023	LINC00513		0	7.386552141	0.014688602	-38.32440538	down	0	0	0	0	0	8	9	8
427	ENSG0000023	HMGN2P5	high mobil	55.96131372	143.6151864	0.046191332	-2.545076652	down	58	47	76	34	48	140	161	99
428	ENSG0000023	AC098484.2		5.295998772	0	0.034357326	29.13347837	up	4	0	12	4	0	0	0	0
429	ENSG0000023	PPP1R3E	protein pho	146.8774327	76.46347591	0.027798908	1.911660267	up	111	92	187	162	77	24	77	284
430	ENSG0000023	LDHAP2	lactate deh	3.041742455	17.90144308	0.010755546	-6.022536489	down	2	5	4	0	27	5	16	33
431	ENSG0000023	AL450326.2		0	5.774967282	0.028648788	-30.39201093	down	0	0	0	0	0	4	13	8
432	ENSG0000023	C1DP2	C1D nucle	0	6.560555689	0.028084982	-33.74366612	down	0	0	0	0	4	8	0	4
433	ENSG0000023	SUCLG2P2	succinate-	6.406055366	0	0.005100549	35.62855893	up	6	12	2	4	0	0	0	0
434	ENSG0000023	AC108448.2		0	6.875847043	0.02261155	-36.13236948	down	0	0	0	0	11	3	6	0
435	ENSG0000023	AC007666.1		5.411162395	0	0.028080762	29.88879353	up	8	5	0	9	0	0	0	0
436	ENSG0000023	LINC00299	long interg	17.8996839	3.956770629	0.033852024	4.088487344	up	12	31	16	6	4	0	4	26
437	ENSG0000023	RPS3AP39	ribosomal	0	9.481699836	0.029988292	-49.38926244	down	0	0	0	0	0	14	4	0
438	ENSG0000023	LINC01684		15.66327443	3.617094944	0.042169734	5.191837253	up	21	18	11	12	0	5	0	8
439	ENSG0000023	MID1IP1-AS1	MID1IP1 a	0	6.244410907	0.009759799	-32.3018515	down	0	0	0	0	8	4	4	3
440	ENSG0000023	MAP3K20-AS1		0	5.435458163	0.043228866	-27.89347544	down	0	0	0	0	0	7	4	4
441	ENSG0000023	IGKV2D-30	immunogl	47.7176156	11.82745455	0.025218134	3.97394965	up	53	16	45	74	24	0	3	45
442	ENSG0000023	IGKV1-6	immunogl	57.40608246	11.18455982	0.021635054	5.140208488	up	60	5	102	57	25	0	10	8
443	ENSG0000023	IGKV3-20	immunogl	684.2671865	294.6651846	0.030656007	2.323897638	up	1093	250	668	811	513	70	123	755
444	ENSG0000023	IGKV1D-33	immunogl	116.8874895	27.36748056	0.003616358	4.175848458	up	133	62	143	120	41	0	17	146
445	ENSG0000023	LILRA2	leukocyte i	216.7570226	513.3233625	0.005845395	-2.364800687	down	573	194	131	76	498	159	507	2054
446	ENSG0000024	SMKR1	small lysin	16.39047918	1.539927547	0.021040214	8.341805749	up	16	26	8	12	0	0	0	23
447	ENSG0000024	RPS19P3	ribosomal	1.44468081	14.16325956	0.02311102	-9.934284721	down	0	0	4	1	20	10	4	5
448	ENSG0000024	AC092919.1		3.355933564	19.87908597	0.031393047	-5.7378632	down	6	0	8	0	11	19	11	31
449	ENSG0000024	AL645465.1		0.911888557	10.56004801	0.035819656	-10.83430722	down	2	0	1	1	0	13	11	3
450	ENSG0000024	IGKV2-24	immunogl	79.06498971	15.2692497	0.021604338	5.199406027	up	42	5	72	177	21	6	0	66
451	ENSG0000024	C8orf58	chromoson	80.41433472	39.64973939	0.034619059	1.976255976	up	73	72	53	109	31	8	58	176
452	ENSG0000024	AL627309.5		16.58537659	54.05254627	0.019697039	-3.291716285	down	26	20	12	9	23	6	166	106
453	ENSG0000024	TCP10L	t-complex	0	7.784941911	0.043502864	-40.81468317	down	0	0	0	0	12	6	0	0
454	ENSG0000024	AC005062.1		4910.594734	10295.57042	0.046270268	-2.096352079	down	3025	5699	5542	3692	7841	8351	7855	11979
455	ENSG0000024	IGKV2-30	immunogl	177.3133385	73.43062315	0.042554724	2.399487674	up	144	90	167	274	99	5	118	171
456	ENSG0000024	IGKV1-12	immunogl	150.6155393	33.18203704	0.010529599	4.484829009	up	168	28	230	166	55	0	15	168
457	ENSG0000024	MRPS31P5	mitochond	7.952824346	38.59606368	0.049324963	-4.864097868	down	4	3	17	5	0	34	8	238
458	ENSG0000024	RN7SL364P	RNA, 7SL	8.377036129	0.267813486	0.031139035	17.34363228	up	4	0	13	14	0	0	0	4
459	ENSG0000024	NPIPB5	nuclear po	1365.258091	764.1380546	0.043575635	1.7829541	up	1554	1463	1104	1180	437	138	1627	2734
460	ENSG0000024	IGKV2-28	immunogl	216.5135087	85.27949929	0.041860627	2.531577592	up	232	66	325	222	151	8	53	263
461	ENSG0000024	AL645922.1		9.144014782	0.066953372	0.004302649	34.89514856	up	22	0	7	12	0	0	0	1
462	ENSG0000024	RN7SL145P	RNA, 7SL	0	6.286289749	0.007751743	-32.57001575	down	0	0	0	0	8	4	2	10
463	ENSG0000024	IGKV3-15	immunogl	491.5063568	182.0841562	0.01032757	2.698206516	up	686	172	510	620	303	27	169	391
464	ENSG0000024	AL021707.5		9.982653035	0.414257494	0.00571837	16.79458193	up	26	2	15	2	0	0	1	3
465	ENSG0000024	IGKV1-27	immunogl	101.7220371	40.56051639	0.039268057	2.504648021	up	115	20	207	56	40	12	58	107
466	ENSG0000024	A2M-AS1	A2M antis	2.219735726	18.2383261	0.029152179	-7.466339975	down	13	0	0	0	15	13	13	35
467	ENSG0000024	AC105285.1		15.28976792	1.991796835	0.021980836	6.434843063	up	25	17	18	2	0	0	4	17
468	ENSG0000024	LINC00968	long interg	2.03655768	11.30246726	0.043460472	-5.724014761	down	0	3	0	4	15	4	9	27
469	ENSG0000024	MEF2C-AS1	MEF2C an	15.07751914	45.68978243	0.027528743	-2.920100458	down	47	11	4	9	37	33	29	98
470	ENSG0000024	AC106882.1		5.574959505	0	0.028999629	30.69278996	up	4	0	12	5	0	0	0	0
471	ENSG0000024	AC097103.2		6.57181182	0	0.014168078	36.46608236	up	13	6	0	9	0	0	0	0
472	ENSG0000025	AC139495.3		81.19948169	179.7513906	0.038732349	-2.214547176	down	177	70	61	42	153	29	419	304
473	ENSG0000025	ST3GAL1P1	ST3 beta-g	0	12.49033451	0.001683304	-65.14307808	down	0	0	0	0	0	14	9	29
474	ENSG0000025	AC106865.1		8.990880196	30.26776547	0.029935888	-3.453502184	down	4	16	4	8	20	12	10	208
475	ENSG0000025	BMS1P2		1.952725137	16.06330621	0.023776399	-8.40724966	down	0	0	0	7	8	6	32	42
476	ENSG0000025	AC037459.3		134.1206206	72.86228367	0.015047416	1.815308645	up	131	104	116	165	47	25	91	329
477	ENSG0000025	TRNP1	TMF1-regu	0	8.367977502	0.008182318	-43.96078152	down	0	0	0	0	11	5	0	23
478	ENSG0000025	MIR3142HG	MIR3142 l	33.01554646	4.693861174	0.023360074	7.29557643	up	85	0	52	12	8	2	0	11

479	ENSG0000025	CA3-AS1	CA3 antise	45.64632049	174.8144521	0.02741211	-3.812202091	down	43	80	29	19	45	208	136	34
480	ENSG0000025	ALG1L13P	asparagine	0	7.438082071	0.008920191	-40.280103	down	0	0	0	0	4	0	20	27
481	ENSG0000025	AP003097.1		5.254310333	0	0.036070768	28.79608074	up	0	7	3	8	0	0	0	0
482	ENSG0000025	STX16-NPEPL1	STX16-NF	124.0261692	37.45314185	0.03618291	3.321975395	up	130	156	9	184	3	26	31	206
483	ENSG0000025	AP003072.2		0.61380983	10.33665273	0.00646851	-19.10966679	down	0	2	0	0	13	4	13	10
484	ENSG0000025	AP002990.1		383.2086187	80.86323794	0.010122984	4.73542124	up	173	1002	144	15	49	27	176	149
485	ENSG0000025	AP001266.2		0	5.917397549	0.031390911	-31.84348658	down	0	0	0	0	3	0	22	3
486	ENSG0000025	AC010203.1		24.0421194	6.367092007	0.044956818	3.376193395	up	22	20	16	34	4	0	4	62
487	ENSG0000025	LBX2-AS1	LBX2 anti:	35.79389011	78.36777435	0.046060904	-2.141024917	down	80	20	30	26	83	48	61	112
488	ENSG0000025	AC073896.4		30.28358084	4.862257717	0.007480856	5.577558078	up	24	23	35	32	0	0	14	28
489	ENSG0000025	AC025034.1		24.39678953	63.872773	0.027261227	-2.605967713	down	22	12	40	19	46	33	108	72
490	ENSG0000025	TRIM6-TRIM34	TRIM6-TF	46.51801957	0	0.0180395	252.3630086	up	0	0	84	79	0	0	0	0
491	ENSG0000025	HSPE1P2	heat shock	0	5.752411289	0.007660632	-29.89911088	down	0	0	0	0	4	4	4	16
492	ENSG0000025	AL356019.2		47.5295115	11.500556	0.01159036	3.809664203	up	71	28	25	70	6	0	12	103
493	ENSG0000025	TUBB3	tubulin bet	5.587428791	0	0.02984725	30.7493828	up	4	0	13	4	0	0	0	0
494	ENSG0000025	AF111169.3		0	6.262342784	0.017057184	-33.83265702	down	0	0	0	0	9	0	4	35
495	ENSG0000025	AL136298.3		9.635184151	1.601739614	0.048855028	5.941952045	up	12	3	20	3	2	0	4	1
496	ENSG0000025	USP3-AS1	USP3 antis	18.74787668	39.97613491	0.048116963	-2.100744692	down	22	22	12	17	19	25	55	95
497	ENSG0000025	AC012170.2		5.764938841	18.94026439	0.041704245	-3.246003213	down	8	5	6	4	27	6	23	17
498	ENSG0000025	AC100830.1		0.477653817	7.753012547	0.033208277	-15.15356587	down	1	1	0	0	0	10	4	11
499	ENSG0000025	ST20-AS1	ST20 antis	61.36403205	106.8252845	0.031059707	-1.744867246	down	101	68	30	52	86	36	137	390
500	ENSG0000025	AC012236.1		23.51060212	3.90662157	0.018770945	5.380098908	up	28	12	21	32	4	0	0	38
501	ENSG0000026	AC104938.1		0	7.515397404	0.019322796	-38.87152869	down	0	0	0	0	7	8	0	3
502	ENSG0000026	SLX1B-SULT1A4	SLX1B-SU	258.9978541	106.7631586	0.019350129	2.389169616	up	334	176	232	288	32	13	193	697
503	ENSG0000026	AC138811.2		0	283.3678787	8.08292E-13	-19628621.95	down	0	0	0	0	691	0	225	0
504	ENSG0000026	AC022336.2		11.3430164	0	4.81011E-05	64.20996553	up	13	13	9	9	0	0	0	0
505	ENSG0000026	LINC02367		8.21965694	0	0.006322072	45.82124413	up	20	0	5	12	0	0	0	0
506	ENSG0000026	SCX	scleraxis b	15.34597117	0.602580345	0.005379968	17.05870682	up	4	4	26	21	0	0	0	9
507	ENSG0000026	FAM157C	family witl	95.02831141	262.3337252	0.031409446	-2.759684533	down	257	107	14	51	311	24	396	853
508	ENSG0000026	AC009133.3		81.27082573	40.89682735	0.0289503	1.957882892	up	76	45	74	118	30	13	51	176
509	ENSG0000026	AC092803.2		2.931543787	29.20844438	0.002363626	-9.344157915	down	9	0	0	5	20	26	17	41
510	ENSG0000026	AC020978.3		8.818591299	1.267847012	0.035193002	6.174847479	up	9	7	9	9	0	0	5	3
511	ENSG0000026	AL031717.1		0.557921468	14.64418953	0.005378055	-27.19545139	down	0	0	0	2	27	0	18	24
512	ENSG0000026	TEN1-CDK3	TEN1-CDI	138.6065197	67.077114	0.028656118	2.028435936	up	161	139	76	166	27	16	124	322
513	ENSG0000026	AC010542.4		8.272579834	0.267813486	0.008669249	16.26285184	up	8	4	8	12	0	0	0	4
514	ENSG0000026	AC012615.1		59.4770199	135.6453604	0.005395433	-2.285985727	down	56	46	52	74	152	41	178	308
515	ENSG0000026	AL031708.1		5.77677064	29.45673489	0.014218357	-5.258539067	down	6	9	3	4	24	0	26	235
516	ENSG0000026	MFSD1P1	major facil	0	4.734952502	0.044451365	-25.31869608	down	0	0	0	0	9	0	5	9
517	ENSG0000026	AC004771.3		6.582025421	24.77251694	0.024971101	-3.700655724	down	16	8	0	5	30	8	20	80
518	ENSG0000026	AC027796.3		7.363124956	0	0.041459365	40.20533289	up	0	5	20	0	0	0	0	0
519	ENSG0000026	MAPK8IP1P1		0	5.965642103	0.034911674	-31.00876294	down	0	0	0	0	0	6	10	2
520	ENSG0000026	AC124283.3		0	6.788238845	0.01913231	-35.11434339	down	0	0	0	0	0	8	4	15
521	ENSG0000026	OVCA2	ovarian tur	50.87531653	167.5736088	0.005951044	-3.259421049	down	90	26	60	36	170	133	79	162
522	ENSG0000026	AC133552.4		0	5.210331863	0.029861855	-28.0395607	down	0	0	0	0	8	0	6	18
523	ENSG0000026	SNORA4	small nucl	14.43565477	35.12685516	0.014545108	-2.439231824	down	21	14	11	12	35	10	51	92
524	ENSG0000026	AC112907.3		66.64509487	139.7258939	0.015129947	-2.096786319	down	57	47	74	75	134	53	205	264
525	ENSG0000026	AC100778.2		5.495632425	0.267813486	0.037970889	11.08976282	up	10	4	4	5	0	0	0	4
526	ENSG0000026	BAHCC1	BAH dom	36.42689439	68.77460323	0.044924512	-1.880011741	down	45	50	23	24	52	36	55	256
527	ENSG0000026	AC138150.2		4.884078279	39.96996988	0.001399254	-7.985047883	down	7	0	5	8	39	34	14	41
528	ENSG0000026	ARHGAP27P2		8.617005045	1.018013824	0.016010734	8.982500062	up	9	12	4	8	0	1	0	6
529	ENSG0000026	AC015802.4		0.341497804	6.579798714	0.04629115	-13.68361752	down	2	0	0	0	3	8	2	3
530	ENSG0000026	AC018766.1		0	6.043956754	0.026761572	-31.62203264	down	0	0	0	0	0	5	12	6
531	ENSG0000026	AC026304.1		0	7.056288148	0.004134484	-37.17479959	down	0	0	0	0	12	2	5	10
532	ENSG0000026	AC026202.3		1.195242314	13.18825906	0.026926449	-9.853587196	down	7	0	0	0	15	3	27	7
533	ENSG0000026	AC245128.3		9.476608195	28.30254862	0.027977121	-2.994775641	down	25	4	6	8	25	3	37	150
534	ENSG0000026	MAN1B1-DT		18.76624474	38.441352	0.049240423	-2.044896611	down	21	17	16	19	35	14	60	76
535	ENSG0000026	AP001160.3		21.823468	7.155205867	0.047373255	2.912086445	up	11	12	29	28	6	2	5	42
536	ENSG0000026	AC019171.1		0	6.810507022	0.012100625	-36.83825355	down	0	0	0	0	9	0	5	40
537	ENSG0000026	DMRTC1	DMRT lik	7.857041657	0	0.03041655	43.17985579	up	17	0	17	0	0	0	0	0
538	ENSG0000026	RPARP-AS1	RPARP an	217.0360099	140.4908745	0.041539492	1.531233097	up	277	166	153	266	100	46	172	618
539	ENSG0000026	AC004754.1		53.92565377	0.468673601	2.78633E-07	66.17619422	up	10	63	42	74	0	0	0	7
540	ENSG0000026	AC078802.1		0	7.491117306	0.045439636	-39.22972572	down	0	0	0	0	0	8	12	0
541	ENSG0000026	AL117336.2		0	5.208583351	0.041983927	-27.69721692	down	0	0	0	0	12	0	4	4
542	ENSG0000026	AL136115.2		0	4.347518049	0.049804377	-23.48077951	down	0	0	0	0	5	0	8	14
543	ENSG0000027	AC087203.3		9.830615426	1.082916954	0.010020671	8.295205284	up	15	7	8	10	2	0	0	6
544	ENSG0000027	HIST2H4B	histone clu	61.50015974	114.6743968	0.016447655	-1.849855871	down	88	44	49	67	96	50	161	251
545	ENSG0000027	BUD13P1	BUD13 ho	0.512246706	11.75871793	0.028898804	-19.26870461	down	3	0	0	0	4	16	0	8
546	ENSG0000027	AC016727.1		15.17105205	40.686378	0.036505419	-2.6057066	down	25	5	13	20	42	27	24	69
547	ENSG0000027	AC010680.3		0	7.890721383	0.008562338	-42.22107456	down	0	0	0	0	12	0	15	9
548	ENSG0000027	AL157791.2		0	12.40538335	0.002876006	-64.86934998	down	0	0	0	0	9	11	12	0
549	ENSG0000027	AL162258.2		0.682995608	12.72113966	0.015002369	-15.4712587	down	4	0	0	0	4	16	2	16
550	ENSG0000027	AC055822.1		32.84523372	13.15304813	0.038928542	2.393912464	up	56	13	26	42	12	2	16	66
551	ENSG0000027	AC114760.2		7.265467871	39.1549118	0.037049136	-5.360195124	down	20	4	9	0	44	0	96	55
552	ENSG0000027	AC004982.1		16.8581815	0.669533716	0.013517935	18.18450921	up	0	10	32	16	0	0	0	10
553	ENSG0000027	AC027644.3		23.21345722	6.501269075	0.04770913	3.826540614	up	14	9	39	24	0	6	10	10
554	ENSG0000027	AC004982.2		20.93786529	4.326630744	0.037943588	4.411391279	up	26	8	30	19	0	0	14	20
555	ENSG0000027	AC009570.1		0	8.895923805	0.034404891	-46.21826857	down	0	0	0	0	0	14	0	4
556	ENSG0000027	AL355512.1		0	7.151542275	0.019758368	-37.46927754	down	0	0	0	0	10	4	6	0
557	ENSG0000027	AC009275.1		0	4.979488393	0.048932212	-25.68086888	down	0	0	0	0	4	5	0	8
558	ENSG0000027	AC008033.3		14.8411954	2.238618784	0.011498538	6.556769191	up	19	9	15	16	5	0	0	8
559	ENSG0000027	AC024909.2		65.14863648	176.3462151	0.018601086	-2.690672133	down	200	30	25	52	275	40	116	497
560	ENSG0000027	IGHV2-70	immunogl	43.5740983	3.394320614	0.000705107	12.80836549	up	24	8	83	46	8	0	0	10
561	ENSG0000027	RIMBP3B	RIMS bind	36.31589891	7.248718398	0.031055	4.990758034	up	44	31	48	19	2	2	25	0
562	ENSG0000027	AL049757.1		4.434368644	0	0.049862106	24.43597253	up	6	0	5	7	0	0	0	0
563	ENSG0000027	HIST1H3E	histone clu	1.398368562	15.6888704	0.002902146	-11.74981193	down	1	4	0	0	16	7	8	63
564	ENSG0000027	NPHP3-ACAD11	NPHP3-AC	290.5734454	165.7471394	0.034202287	1.759357248	up	385	202	285	286	166	60	250	282
565	ENSG0000027	AC026356.1		0	5.219708814	0.036724154	-27.2065881	down	0	0	0	0	0	5	5	16
566	ENSG0000027	HIST1H3A	histone clu	0.682995608	10.57495116	0.046075424	-13.45602596	down	4	0	0	0	15	8	0	8
567	ENSG0000027	AC010327.5		35.77940183	75.35077809	0.038176873	-2.133412728	down	61	14	34	40	53	15	68	501
568	ENSG0000027	AC010654.1		2.202873417	16.77707074	0.008414233	-7.087684628	down	8	0	0	3	8	8	28	47
569	ENSG0000027	AL357033.4		84.95691874	45.73610228	0.027531434	1.866831527	up	172	72	46	72	33	21	37	204
570	ENSG0000027	RF00002		24.82588598	120.0373357	0.029301603	-4.778007767	down	57	3	18	32	38	165	20	17
571	ENSG0000027	RNA5-8SN1		29.82557473	164.8325478	0.017954114	-5.476641246	down	68	4	20	40	47	231	24	20
572	ENSG0000027	AC135068.2		6.842164502	0	0.04682646	37.33398887	up	0	9	14	0	0	0	0	0
573	ENSG0000027	AC027601.5		0	5.908874025	0.025708404	-31.13332338	down	0	0	0	0	0	4	13	10
574	ENSG0000027	AL928654.4		0	11.15386226	0.001651776	-60.42920764	down	0	0	0	0	4	0	28	57
575	ENSG0000027	AC093525.8		14.29943911	49.98569422	0.04013423	-3.510029196	down	14	6	2	34	64	5	96	69
576	ENSG0000027	AL162426.1		5.132201662	0	0.032753243	28.3357647	up	8	5	0	8	0	0	0	0
577	ENSG0000027	AL049780.3		16.06160707	2.473007701	0.017307059	5.391463556	up	16	12	13	21	0	0	5	21
578	ENSG0000027	AP003108.4		8.760196959	0.267813486	0.037041679	18.32532374	up	2	0	4	26	0	0	0	4
579	ENSG0000027	AC008764.9		9.096236919	0.267813486	0.020054989	18.70789817	up	17	12	0	9	0	0	0	4
580	ENSG0000027	AC010997.6		0	8.462510027	0.037739215	-44.05754812	down	0	0	0	0	0	12	5	0
581	ENSG0000028	MIR4697HG	MIR4697 l	178.9522168	94.57481275	0.025331808	1.880050779	up	156	122	219	183	62	29	172	282
582	ENSG0000028	AC006059.2		0	12.67311009	0.000483702	-65.30337935	down	0	0	0	0	10	13	4	6
583	ENSG0000028	AC005154.5		0	4.704365688	0.02239654	-24.97402944	down	0	0	0	0	1	3	3	28
584	ENSG0000028	AC105749.1		14.87653822	33.4726181	0.046702391	-2.238517627	down	24	11	12	14	9	17	57	116
585	ENSG0000028	RHOXF1P3	Rhox hom	11.21479879	0	0.012624821	61.56245098	up	0	12	0	27	0	0	0	0
586	ENSG0000028	KBTBD11-OT1	KBTBD11	21.78683424	2.778432981	0.012757163	6.994168015	up	76	14	4	12	0	0	8	16
587	ENSG0000028	LINC01260	long interg	17.30735727	0	0.000481124	96.30796272	up	8	0	48	7	0	0	0	0
588	ENSG0000028	AC092865.3		3.650481634	19.29627609	0.039432688	-5.314354876	down	7	8	0	0	25	2	30	47
589	ENSG0000028	AC005280.2		15.82741268	50.09891839	0.001667095	-3.090670168	down	33	10	12	13	50	21	62	103
590	ENSG0000028	AL590764.2		29.20296142	84.6010675	0.036631395	-2.887456073	down	13	11	14	70	80	54	35	248
591	ENSG0000028	ATXN7	ataxin 7	13.17116703	120.7036786	0.000296049	-9.164542757	down	2	9	2	34	61	92	169	107
592	ENSG0000028	Z83840.1		6.324247721	20.28872626	0.022258323	-3.084142667	down	13	4	7	3	10	13	24	56
593	ENSG0000028	AC069410.1		0	16.63992704	0.020435596	-87.50605542	down	0	0	0	0	0	27	0	0
594	ENSG0000028	AL135905.2		145.3804422	334.0231416	0.02668856	-2.288910701	down	299	28	164	136	377	156	337	561

Table S6 Target Type

NAME	type
PRKAG1	target
PRKAG2	target
PIK3CD	target
IRS2	target
PIK3R2	target
HMGCR	target
FOXO3	target
PRKAG3	target
FOXO1	target
IGF1R	target
INS	target
STK11	target
AKT2	target
HNF4A	target
LEPR	target
AKT1	target
CD36	target
CPT1A	target
PRKAB2	target
PDPK1	target
TSC1	target
PRKAB1	target
MTOR	target
CCNA1	target
CREB1	target
AKT1S1	target
RPS6KB2	target
STAT3	target
PRKAG1	target
SLC2A1	target
ACSL3	target
TNFRSF1E	target
TNF	target
RELA	target
TNFRSF1/	target
MAPK10	target
IKBKB	target
RNASEL	target
CXCL8	target
TXN	target
NOD2	target
CXCL3	target
MEFV	target
MAP1LC3I	target
TBK1	target
NAMPT	target
CASP1	target
CCL2	target
MAPK1	target
RIPK1	target
JAK1	target
MAPK3	target
IFNAR2	target
GABARAI	target
PRKCD	target
CYBB	target
CYBA	target
TYK2	target
IL6	target
IL1B	target
BCL2	target
TLR4	target
MYD88	target
BIRC2	target
IRF9	target
BIRC3	target
BCL2L1	target
ARPC1B	target
WAS	target
GATA4	target
CD1D	target
PRKCZ	target
RAP1A	target
SYMPK	target
MYH11	target
PRKACA	target
MAP3K5	target
ACTR2	target
MAP3K1	target
ACTN1	target
MSN	target
ARPC5	target
RUNX1	target
TIAM1	target
CTTN	target
MYL2	target
MYH9	target

Table S6 Protein-protein interaction network

NAME1	NAME2	NAME1	NAME2
PRKAG1	AMPK signaling pathway	PRKAG2	Vanillin acetate
PRKAG2	AMPK signaling pathway	HMGCR	guanosine
PIK3CD	AMPK signaling pathway	HMGCR	xiongterpene
IRS2	AMPK signaling pathway	HMGCR	3-O-p-Hydroxy-trans-cinnamoylmaslinic acid
PIK3R2	AMPK signaling pathway	HMGCR	biatractylenolide
HMGCR	AMPK signaling pathway	HMGCR	3-(2-hydroxyacetoxy)-5α,8α-peroxydehydrotumulosic a
FOXO3	AMPK signaling pathway	HMGCR	ganoderic acid B
PRKAG3	AMPK signaling pathway	HMGCR	Z-6-hydroxy-7-methoxy-dihydroligustilide
FOXO1	AMPK signaling pathway	HMGCR	Daturilin
IGF1R	AMPK signaling pathway	HMGCR	cinnacaside
INS	AMPK signaling pathway	HMGCR	phospholipid
STK11	AMPK signaling pathway	HMGCR	chuanxiongnolide R2
AKT2	AMPK signaling pathway	HMGCR	Rubrosterone
HNF4A	AMPK signaling pathway	HMGCR	1,2,3,4,6-Penta-O-galloyl-β-D-glucose
LEPR	AMPK signaling pathway	HMGCR	nomilin
AKT1	AMPK signaling pathway	HMGCR	(Z)-3-butylidenephthalide
CD36	AMPK signaling pathway	HMGCR	cinnacasol
CPT1A	AMPK signaling pathway	HMGCR	Di-(2-ethylhexyl)phthalate
PRKAB2	AMPK signaling pathway	HMGCR	ceanphytamic acid B
PDPK1	AMPK signaling pathway	HMGCR	stigmasterol-β-glucoside
TSC1	AMPK signaling pathway	PRKAG3	2-methoxybenzoic acid
PRKAB1	AMPK signaling pathway	PRKAG3	Vanillin acetate
MTOR	AMPK signaling pathway	IGF1R	guanosine
CCNA1	AMPK signaling pathway	INS	1-pentadecanol
CREB1	AMPK signaling pathway	INS	Phellatin
AKT1S1	AMPK signaling pathway	HNF4A	1-pentadecanol
RPS6KB2	AMPK signaling pathway	HNF4A	Phellatin
PRKAB2	Adipocytokine signaling pathway	AKT1	guanosine
CPT1A	Adipocytokine signaling pathway	AKT1	Cyanidin
STAT3	Adipocytokine signaling pathway	AKT1	5,6,4′-Trihydroxy-7,3′-dimethoxyflavone
PRKAG1	Adipocytokine signaling pathway	AKT1	(S)-naringenin
SLC2A1	Adipocytokine signaling pathway	AKT1	7-hydroxy coumarin
PRKAG2	Adipocytokine signaling pathway	AKT1	natsudaidain
IRS2	Adipocytokine signaling pathway	AKT1	Jionoside B2
ACSL3	Adipocytokine signaling pathway	AKT1	Eucalyptin
TNFRSF1B	Adipocytokine signaling pathway	AKT1	Liquiritin apioside
TNF	Adipocytokine signaling pathway	AKT1	5-hydroxy-3,3',4',7,8-pentamethoxyflavone
PRKAB1	Adipocytokine signaling pathway	AKT1	ledebouriellol
PRKAG3	Adipocytokine signaling pathway	AKT1	Isosinensetin
MTOR	Adipocytokine signaling pathway	PRKAB2	2-methoxybenzoic acid
RELA	Adipocytokine signaling pathway	PRKAB2	guanosine
TNFRSF1A	Adipocytokine signaling pathway	PRKAB2	Vanillin acetate
MAPK10	Adipocytokine signaling pathway	CREB1	guanosine
IKBKB	Adipocytokine signaling pathway	ACSL3	Phellatin
STK11	Adipocytokine signaling pathway	MAPK10	guanosine
AKT2	Adipocytokine signaling pathway	IKBKB	Vanillin acetate
LEPR	Adipocytokine signaling pathway	IKBKB	2-methoxybenzoic acid
AKT1	Adipocytokine signaling pathway	CASP1	tangshenoside I
CD36	Adipocytokine signaling pathway	MAPK1	5,6,4′-Trihydroxy-7,3′-dimethoxyflavone
RNASEL	NOD-like receptor signaling pathway	MAPK3	guanosine
CXCL8	NOD-like receptor signaling pathway	PRKCD	cinnacaside
TXN	NOD-like receptor signaling pathway	PRKCD	Spinoside A
NOD2	NOD-like receptor signaling pathway	PRKCD	stigmasterol-β-glucoside
CXCL3	NOD-like receptor signaling pathway	CYBB	L-Tetrandrine
MEFV	NOD-like receptor signaling pathway	CYBA	L-Tetrandrine
TNF	NOD-like receptor signaling pathway	IL6	9-Hydroxypaeonilactone A
RELA	NOD-like receptor signaling pathway	IL6	4-O-Ethylpaeoniflorin
IKBKB	NOD-like receptor signaling pathway	IL6	Paeonisuffrone
MAP1LC3B	NOD-like receptor signaling pathway	IL6	Oxypaeoniflorin
TBK1	NOD-like receptor signaling pathway	IL6	nomilin
NAMPT	NOD-like receptor signaling pathway	TLR4	phospholipid
CASP1	NOD-like receptor signaling pathway	TLR4	Phellatin
CCL2	NOD-like receptor signaling pathway	BCL2L1	natsudaidain
MAPK1	NOD-like receptor signaling pathway	BCL2L1	Liquiritin apioside
RIPK1	NOD-like receptor signaling pathway	BCL2L1	Betavulgarin
JAK1	NOD-like receptor signaling pathway	BCL2L1	ledebouriellol
MAPK3	NOD-like receptor signaling pathway	BCL2L1	Isosinensetin
IFNAR2	NOD-like receptor signaling pathway	PRKCZ	Spinoside A
GABARAPL2	NOD-like receptor signaling pathway	PRKACA	Cyanidin
PRKCD	NOD-like receptor signaling pathway	PRKACA	7-hydroxy coumarin
CYBB	NOD-like receptor signaling pathway	PRKACA	Jionoside B2
CYBA	NOD-like receptor signaling pathway	LYN	L-Tetrandrine
TYK2	NOD-like receptor signaling pathway	LYN	Epistephanine
MAPK10	NOD-like receptor signaling pathway	P2RY12	senkyunolide D
IL6	NOD-like receptor signaling pathway	P2RY12	Senkyunolide-R
IL1B	NOD-like receptor signaling pathway	P2RY12	chuanxiongoside A
BCL2	NOD-like receptor signaling pathway	P2RY12	chuanxiongnolide R2
TLR4	NOD-like receptor signaling pathway	P2RY12	Rehmapicrogenin
MYD88	NOD-like receptor signaling pathway	SYK	Cyanidin
BIRC2	NOD-like receptor signaling pathway	SYK	7-hydroxy coumarin
IRF9	NOD-like receptor signaling pathway	SYK	Jionoside B2
BIRC3	NOD-like receptor signaling pathway	2-methoxybenzoic ac	Prepared Rehmannia Root
BCL2L1	NOD-like receptor signaling pathway	Vanillin acetate	Angelica Sinensis
ARPC1B	Tight junction	guanosine	Prepared Rehmannia Root
PRKAG1	Tight junction	3-(2-hydroxyacetoxy	Deer-horn Glue
WAS	Tight junction	ganoderic acid B	Poria cocos
PRKAG2	Tight junction	Z-6-hydroxy-7-meth	Chenpi

HCLS1	target
GNAI2	target
MYLK	target
PIK3R5	target
PTGS1	target
FGB	target
LYN	target
P2RY12	target
FGA	target
SYK	target
ITGA2	target
FGG	target
GP1BA	target
GP6	target
APBB1IP	target
COL1A1	target
COL3A1	target
FCGR2A	target
GNAQ	target
LCP2	target
AMPK sig	pathway
Adipocyto	pathway
NOD-like r	pathway
Tight junct	pathway
Platelet act	pathway
2-methoxy	component
Vanillin ac	component
guanosine	component
3-(2-hydro	component
ganoderic a	component
Z-6-hydrox	component
Daturilin	component
cinnacaside	component
phospholip	component
chuanxiong	component
Rubrostero	component
1,2,3,4,6-P	component
nomilin	component
(Z)-3-butyl	component
cinnacasol	component
Di-(2-ethyl	component
ceanphytan	component
1-pentadec	component
Cyanidin	component
5,6,4′ -Tri	component
(S)-naringe	component
7-hydroxy	component
natsudaidai	component
Jionoside B	component
Eucalyptin	component
Liquiritin a	component
5-hydroxy-	component
ledebouriel	component
Isosinenset	component
tangshenos	component
Spinoside	component
L-Tetrandri	component
9-Hydroxy	component
4-O-Ethylp	component
Paeonisuffr	component
Oxypaeoni	component
Betavulgari	component
Epistephan	component
senkyunoli	component
Senkyunoli	component
chuanxiong	component
Prepared R	medicine
Angelica S	medicine
Deer-horn	medicine
Poria cocos	medicine
Chenpi	medicine
Red Peony	medicine
Ligusticum	medicine
Medicinal	medicine
Codonopsis	medicine
hip pain	symptom
Episodic p	symptom
Joint swelli	symptom
Ecchymosi	symptom
Obesity	symptom
Chest tight	symptom
Tongue_Ed	symptom
anorexia	symptom

GATA4	Tight junction
CD1D	Tight junction
PRKCZ	Tight junction
PRKAG3	Tight junction
STK11	Tight junction
RAP1A	Tight junction
SYMPK	Tight junction
MYH11	Tight junction
PRKACA	Tight junction
MAP3K5	Tight junction
ACTR2	Tight junction
PRKAB2	Tight junction
MAP3K1	Tight junction
ACTN1	Tight junction
MSN	Tight junction
ARPC5	Tight junction
PRKAB1	Tight junction
RUNX1	Tight junction
MAPK10	Tight junction
TIAM1	Tight junction
CTTN	Tight junction
MYL2	Tight junction
MYH9	Tight junction
HCLS1	Tight junction
PIK3CD	Platelet activation
PIK3R2	Platelet activation
PRKCZ	Platelet activation
GNAI2	Platelet activation
MYLK	Platelet activation
PIK3R5	Platelet activation
PTGS1	Platelet activation
RAP1A	Platelet activation
AKT2	Platelet activation
AKT1	Platelet activation
MAPK1	Platelet activation
PRKACA	Platelet activation
MAPK3	Platelet activation
FGB	Platelet activation
LYN	Platelet activation
P2RY12	Platelet activation
FGA	Platelet activation
SYK	Platelet activation
ITGA2	Platelet activation
FGG	Platelet activation
GP1BA	Platelet activation
GP6	Platelet activation
APBB1IP	Platelet activation
COL1A1	Platelet activation
COL3A1	Platelet activation
FCGR2A	Platelet activation
GNAQ	Platelet activation
LCP2	Platelet activation
PIK3CD	Platelet activation
PIK3R2	Platelet activation
PRKCZ	Platelet activation
GNAI2	Platelet activation
MYLK	Platelet activation
PIK3R5	Platelet activation
PTGS1	Platelet activation
RAP1A	Platelet activation
AKT2	Platelet activation
AKT1	Platelet activation
MAPK1	Platelet activation
PRKACA	Platelet activation
MAPK3	Platelet activation
FGB	Platelet activation
LYN	Platelet activation
P2RY12	Platelet activation
FGA	Platelet activation
SYK	Platelet activation
ITGA2	Platelet activation
FGG	Platelet activation
GP1BA	Platelet activation
GP6	Platelet activation
APBB1IP	Platelet activation
COL1A1	Platelet activation
COL3A1	Platelet activation
FCGR2A	Platelet activation
GNAQ	Platelet activation
LCP2	Platelet activation
PRKAG1	2-methoxybenzoic acid
PRKAG1	Vanillin acetate
PRKAG2	2-methoxybenzoic acid

Daturilin	Deer-horn Glue
cinnacaside	Deer-horn Glue
phospholipid	Red Peony Root
chuanxiongnolide R2	Ligusticum Wallichii
Rubrosterone	Red Peony root
1,2,3,4,6-Penta-O-ga	Red Peony root
nomilin	Chenpi
(Z)-3-butylidenephth	Ligusticum Wallichii
cinnacasol	Prepared Rehmannia Root
Di-(2-ethylhexyl)pht	Angelica Sinensis
ceanphytamic acid B	
1-pentadecanol	Chenpi
Cyanidin	Prepared Rehmannia Root
5,6,4′ -Trihydroxy-	Red Peony root
(S)-naringenin	Chenpi
7-hydroxy coumarin	Ligusticum Wallichii
natsudaidain	Chenpi
Jionoside B2	Prepared Rehmannia Root
Eucalyptin	Medicinal Cyathula Root
Liquiritin apioside	Chenpi
5-hydroxy-3,3',4',7,8-	Red Peony root
ledebouriellol	Chenpi
Isosinensetin	Chenpi
tangshenoside I	Chenpi
Spinoside A	Chenpi
L-Tetrandrine	Deer-horn Glue
9-Hydroxypaeonilact	Red Peony root
4-O-Ethylpaeoniflori	Red Peony root
Paeonisuffrone	Ligusticum Wallichii
Oxypaeoniflorin	Red Peony root
Betavulgarin	Medicinal Cyathula Root
Epistephanine	Deer-horn Glue
senkyunolide D	Codonopsis Root
Senkyunolide-R	Codonopsis Root
chuanxiongoside A	Ligusticum Wallichii
NOD-like receptor si	hip pain
NOD-like receptor si	Episodic pain
NOD-like receptor si	Joint swelling
Platelet activation	Ecchymosis
Adipocytokine signal	Obesity
Platelet activation	Ecchymosis
AMPK signaling pat	Chest tightness
AMPK signaling pat	hip pain
AMPK signaling pat	Obesity
AMPK signaling pat	Ecchymosis
NOD-like receptor si	Tongue_Edema
AMPK signaling pat	Tongue_Edema
Adipocytokine signal	anorexia
