# Supplementary material for: Jianpi‐Huogu Prescription Repairs Nontraumatic Osteonecrosis of the Femoral Head by Inhibiting NAMPT/STK11/HMGCR/ACAT1 Axis‐Mediated Lipid Production
Source: J Cell Mol Med. 2025 Sep 23;29(18):e70858. doi: 10.1111/jcmm.70858 (PMC12457209; doi:10.1111/jcmm.70858)
Supplement: Supplementary file 3 — Appendix S2: Supplementary Methods [file JCMM-29-e70858-s001.docx]

**Supplemental Methods**

**Section 1: Chemicals and materials**

*Citrus reticulata* Blanco (catalogu: 22022402), *Paeonia lac tiflor a* Pall. (catalogu: 22041701), *Codonopsis pilosula* (Franch.) Nannf. (catalogu: 22031002), *Rehmannia glutinosa* Libosch. (catalogu: 21123102), and *Cinnamomum cassia* Presl (catalogu: 21082002) were purchased from the Beijing Sifang Chinese Medicine decoction pieces company (Beijing, China). *Angelica sinensis* (Oliv.) Diels (catalogu: 2204021) and *Cyathula officinalis* Kuan (catalogu: 2203012) were purchased from the Beijing Shuangqiao Yanjing Traditional Chinese Medicine Slice Factory (Beijing, China). *Ligusticum chuanxiong* Hort. (catalogu: C1151511) and *Atractylodes macrocephala* Koidz. (catalogu: BB221341) were purchased from Beijing Jinchongguang Pharmaceutical Co., Ltd. (Beijing, China). *Poria* cocos (Schw.) Wolf (catalogu: 20220528) was purchased from Beijing Bencao Fangyuan Pharmaceutical Group Co., Ltd. (Beijing, China). Deerhorn glue (catalogu: 20210202) was purchased from Henan Furrentang Pharmaceutical Co., Ltd. (Zhengzhou, China). Lipopolysaccharide (catalogu: L6386) was supplied by Sigma-Aldrich (St. Louis, MO, USA). Methylprednisolone sodium succinate (Approval Number: H20170199) was purchased from Pfizer Manufacturing (NY, USA). 0.9% sodium chloride injection (catalogu: 2303212003) was supplied by Shijiazhuang Siyao Co., Ltd. (Shijiazhuang, China). Penicillin sodium injection (Approval Number: H13020655) was purchased from North China Pharmaceutical Group Co., Ltd. (Shijiazhuang, China). Methanol (catalogu: l1150907120) and acetonitrile (catalogu: SHBN7205) were supplied by Supelco (Darmstadt, Germany). Formic acid (catalogu: 214911) was purchased from Fisher Scientific (Waltham, USA). Pentobarbital sodium (catalogu: P3761) was supplied by Sigma-Aldrich (St. Louis, MO, USA).

**Section 2: Clinical Cohorts**

Clinical research is conducted in accordance with the principles expressed in the Helsinki Declaration, and all patients agree to participate in this study. Inclusion Criteria of our study included: (1) NONFH patients were diagnosed based on the Mont MA presented criteria ^[1]^; (2) Staging criteria for NONFH were based on ARCO ^[2]^; (3) No demonstrable history of direct trauma; (4) A demonstrable history of application of steroid therapy; (5) All NONFH patients were diagnosed for the first visit. Exclusion Criteria for our study included: Individuals with severe primary disease requiring alternative hormone therapy.

**Section 3: Preparation of JHP**

JHP (batch No. ZYS202208) was provided by the Third Affiliated Hospital of Beijing University of Chinese Medicine (Beijing, China). The Chinese medicinal materials were identified by Professor Ying Liu (Institute of Chinese Materia Medica, China Academy of Chinese Medical Sciences). A mixture of *Citrus reticulata* Blanco (5000 g), *Paeonia lac tiflor a* Pall. (5000 g), *Codonopsis pilosula* (Franch.) Nannf. (5000 g), *Rehmannia glutinosa* Libosch. (5000 g), *Cinnamomum cassia* Presl (5000 g), *Angelica sinensis* (Oliv.) Diels (5000 g), *Cyathula officinalis* Kuan (5000 g), *Ligusticum chuanxiong* Hort. (5000 g), *Atractylodes macrocephala* Koidz. (5000 g), *Poria* cocos (Schw.) Wolf (5000 g) were extracted two times by refluxing with 10-fold of water (volume/weight) for 1 h each time. And the supernatant was condensed and dried to yield 4000 g powder using the decompression drying method at 60°C. After pulverizing the Deerhorn glue (3000 g), mix it evenly with the aforementioned 4000 g of powder to obtain JHP.

**Section 4: Analysis of chemical constituents of JHP**

Take the decoction of JHP, centrifuge, filter with a microporous filter membrane, and set aside. Ultra-High Performance Liquid Chromatography Conditions: Chromatographic column (ACQUITY UPLC HSS T3 Column, 100Å, 1.8 µm, 2.1 mm × 100 mm), mobile phase 1% formic acid aqueous solution (A) -0.1% formic acid acetonitrile solution (B); flow rate 0.5 mL/min; detection wavelength 190-400 nm; Column temperature 40 ℃; injection volume 1 μ L. The gradient elutionwas set as follows：98%- 96% A at 0- 3 min; 96%- 93% A at 3- 7 min; 93%- 90% A at 7- 9 min; 90%- 86% A at 9- 11 min; 86%- 83% A at 11- 12 min; 83%- 80% A at 12- 15 min; 80%- 70%A at 15- 16 min; 70%- 58% A at 16- 18 min; 58%- 40% A at 18- 20 min; 40%- 30% A at 20- 22 min; 30%- 2% A at 22- 24 min; 2% A at 24- 27 min and finally 98% A at 27- 30 min. Mass spectrometry conditions: ionization mode: electric spray positive and negative ions; capillary voltage: (±) 0.5 KV; cone hole voltage: 40 V; Ion source temperature: 100 ℃; Solvent removal temperature: 450 ℃; solvent gas removal: 900 L/h; Collection range: 50-1500 Da; Collision energy: low energy 6 eV, high energy (-) 70-100 eV (+) 30-50 eV.

**Section 5: Establishment of early NONFH rat model**

Randomly divide rats into the following 5 groups, with 10 in each group: control group (Control), model group (Model), low-dose treatment group of JHP (JHP-L), medium-dose treatment group of JHP (JHP-M), and high-dose treatment group of JHP (JHP-H). The JHP-L, JHP-M and JHP-H groups were respectively given 0.50, 1.00 (clinically equivalent dose) and 2.00 g/kg/d. All treatments were carried out after the completion of modeling and lasted for 60 days.

**Section 6: Prediction of JHP Putative Targets & Collection of early NONFH-related genes**

JHP putative targets were predicted using TCMIP v2.0 database (http://www.tcmip.cn/TCMIP/index.php/Home) based on chemical structural similarity (Similar score > 0.70). In addition, our previous study identified a list of differentially expressed genes including 1124 upregulated genes and 624 downregulated genes between early NONFH patients and controls provided in National Center of Biotechnology Information Gene Expression Omnibus (https://www.ncbi.nlm.nih.gov/geo/query/acc.cgi?acc=GSE123568) ^[3]^. Then, we also collected the early NONFH-related genes from HPO database (https://hpo.jax.org/app/, June 6, 2023 updated).

**Section 7: Small molecule compounds information**

(Z)-3-butylidenephthalide (catalogu: T3S2072), Senkyunolide H (catalogu: T8159), Ferulic Acid (catalogu: T2215), Guanosine (catalogu: T6523) were purchased from the Topscience Biotechnology Co., Ltd. (Shanghai, China). 5,6,4'-Trihydroxy-7,3'-dimethoxyflavone (catalogu: BBP05813) was purchased from the BioBioPha Biotechnology Co., Ltd. (Yunnan, China). Riligustilide (catalogu: B32724) was purchased from the Shanghai yuanye Bio-Technology Co., Ltd. (Shanghai, China).

**References**

1. Mont MA, Hungerford DS. Non-traumatic avascular necrosis of the femoral head. J Bone Joint Surg Am. 1995 Mar;77(3):459-74. doi: 10.2106/00004623-199503000-00018.
2. Yoon BH, Mont MA, Koo KH, Chen CH, Cheng EY, Cui Q, Drescher W, Gangji V, Goodman SB, Ha YC, Hernigou P, Hungerford MW, Iorio R, Jo WL, Jones LC, Khanduja V, Kim HKW, Kim SY, Kim TY, Lee HY, Lee MS, Lee YK, Lee YJ, Nakamura J, Parvizi J, Sakai T, Sugano N, Takao M, Yamamoto T, Zhao DW. The 2019 Revised Version of Association Research Circulation Osseous Staging System of Osteonecrosis of the Femoral Head. J Arthroplasty. 2020 Apr;35(4):933-940. doi: 10.1016/j.arth.2019.11.029.
3. Li T, Zhang Y, Wang R, Xue Z, Li S, Cao Y, Liu D, Niu Y, Mao X, Wang X, Li W, Guo Q, Guo M, Lin N, Chen W. Discovery and validation an eight-biomarker serum gene signature for the diagnosis of steroid-induced osteonecrosis of the femoral head. Bone. 2019 May; 122:199-208. doi: 10.1016/j.bone.2019.03.008.
